# Phylogenetic tracing of midbrain-specific regulatory sequences suggests single origin of eubilaterian brains

**DOI:** 10.1126/sciadv.ade8259

**Published:** 2023-05-24

**Authors:** Helen C. Schuster, Frank Hirth

**Affiliations:** Department of Basic and Clinical Neuroscience, Maurice Wohl Clinical Neuroscience Institute, and Institute of Psychiatry, Psychology and Neuroscience, King’s College London, London, UK.

## Abstract

Conserved cis-regulatory elements (CREs) control *Engrailed*-, *Pax2*-, and *dachshund*-related gene expression networks directing the formation and function of corresponding midbrain circuits in arthropods and vertebrates. Polarized outgroup analyses of 31 sequenced metazoan genomes representing all animal clades reveal the emergence of *Pax2*- and *dachshund*-related CRE-like sequences in anthozoan Cnidaria. The full complement, including *Engrailed*-related CRE-like sequences, is only detectable in spiralians, ecdysozoans, and chordates that have a brain; they exhibit comparable genomic locations and extensive nucleotide identities that reveal the presence of a conserved core domain, all of which are absent in non-neural genes and, together, distinguish them from randomly assembled sequences. Their presence concurs with a genetic boundary separating the rostral from caudal nervous systems, demonstrated for the metameric brains of annelids, arthropods, and chordates and the asegmental cycloneuralian and urochordate brain. These findings suggest that gene regulatory networks for midbrain circuit formation evolved within the lineage that led to the common ancestor of protostomes and deuterostomes.

## Teaser

Phylogenetic tracing of midbrain-specific gene regulatory sequences suggests a common origin of eubilaterian brains.

## INTRODUCTION

The origin of brains in animal evolution is a major enigma that has remained unresolved. Fossil evidence suggests an emergence before the Paleozoic area 540 million years (My) ago, followed by a short period of 20 to 35 My during the Cambrian for which evidence of brains and nervous systems has been recovered in all three clades of bilateral symmetric animals (Bilateria). The fossilized neural tissues reveal correspondences to the nervous system organization of extant arthropods (*1*), annelids (*2*), and chordates (*3*), suggesting constraints in the development and evolution of species-specific morphologies (*4*). The observed correspondences between fossilized and extant taxa have facilitated the study of their evolution by comparative analysis of the developmental genetic mechanisms underlying character formation and the specification into character states (*5*). These studies revealed notable similarities in expression patterns of homologous genes and the underlying developmental mechanisms regulating brain formation and function in annelids, arthropods, and chordates (*6*–*8*).

However, the sudden and multiple appearance of brains in the fossil record and their presence among extant phyla that are related to brainless sister groups have been interpreted as suggesting independent origins of brains in animal phylogeny (*9*). These interpretations have been reinforced by the discovery of expression patterns of homologous genes showing corresponding domains along the body axes of extant Bilateria that exhibit very different nervous system architectures, ranging from a nerve net in hemichordates (*10*) to centralized nervous systems and neuromeric brains in arthropods (*1*) and vertebrates (*11*). Further genetic evidence revealed that the acquisition of positional identity along the rostrocaudal and dorsoventral axes can occur before neural fate specification and brain formation (*12*). These data suggest that the observed correspondences in gene expression patterns are the result of conserved mechanisms for axial allocation but not for subsequent neural patterning, implying that brain formation did not evolve in brainless Bilateria or was secondarily modified.

Both conjectures have been accounted for by contrasting hypotheses of brain evolution (*9*). These are guided by a consensus definition of the brain as part of the nervous system that consists of an anterior condensation of (inter)neurons and neuropil, which receives and processes sensory information and uses descending fibers assembled in one or more nerve cords to initiate a response. A single origin of the brain has been proposed on the basis of a multitude of similarities and minuteness of resemblances between arthropod and vertebrate brains, ranging from homologous gene expression patterns and functional attributes to corresponding pathologies (*13*). In contrast, it has been hypothesized that the observed similarities in brain formation and function are due to convergence, mediated by ancestral gene regulatory networks (*14*) involved in axial patterning that have been co-opted for the independent evolution of cephalic nervous systems in animal phylogeny (*9*).

Here, we tested the co-option hypothesis by using the recent discovery of highly conserved cis-regulatory elements (CREs) that direct gene regulatory networks mediating axial patterning (*5*, *9*, *10*, *14*) and the formation and function of corresponding midbrain circuits in arthropods and vertebrates (*8*). We carried out phylogenetic tracing of these regulatory sequences in an outgroup analysis across sequenced genomes of 31 eumetazoan species representing all clades of the animal kingdom. We then tested whether the detection of CRE-like sequences is congruent with the presence of a brain in the species examined. Last, we asked whether the occurrence of these CRE-like sequences relates to the developmental genetics and neural architecture underlying annelid, cycloneuralian, and urochordate brains when compared to those of arthropods and chordates.

## RESULTS

As templates for phylogenetic tracing, we used conserved CRE sequences that target the expression of homologous genes *shaven/PAX2 (sv/PAX2)*, *dachshund/DACH1 (dac/DACH1)*, and *invected/ENGRAILED-2 (inv/EN2)* to cells of the vertebrate midbrain-hindbrain boundary (MHB) and the *Drosophila* deutocerebral-tritocerebral boundary (DTB) region, respectively (fig. S1). Like for their discovery in arthropods and vertebrates (*8*), screening was restricted to sequences that are (i) linked to the same homologous genes in the different species. (ii) There is a minimum of 60% sequence identity over at least 55 base pairs (bp) with minimum 1 × 10^−1^ confidence level. (iii) The CRE-like sequences are noncoding and not un-annotated protein sequences, and (iv) the CRE-like sequences are not repetitive elements. We screened the genomes of 31 eumetazoans including Porifera, Ctenophora, Cnidaria, Xenacoelomorpha, Ambulacraria, Chordata, Spiralia, and Ecdysozoa (table S1), focusing on species for which annotated genome sequences are publicly available and can be examined using the Ensembl and Ensembl Metazoa servers of the European Bioinformatics Institute and the server of the National Center for Biotechnology Information (NCBI) of the U.S. National Library of Medicine.

To identify homologs of *sv/PAX2*, *dac/DACH1*, and *inv/EN2* in the genome sequences of the examined species, we applied the Basic Local Alignment Search Tool (BLAST) available at Ensembl and NCBI by using the sequences of homologous *Drosophila* and human genes as screening templates. The obtained results were used to determine relationships by using NGPhylogeny.fr with smart model selection for maximum likelihood phylogenies (*15*). For the identified gene homologs, the genome sequence annotations (and flanking regions for *engrailed* homologs) were extracted and aligned against the conserved CRE sequence identified in *Drosophila*, mouse, and human (fig. S1C) using the EMBOSS Matcher local alignment algorithm to screen for CRE-like sequences that match the selection criteria verified for the conserved regulatory sequences mediating midbrain circuit formation and function in arthropods and vertebrates (*8*).

### *sv/Pax2-* and *dac/DACH1-related* CRE-like sequences emerge in anthozoan Cnidaria

**Fig. 1.**
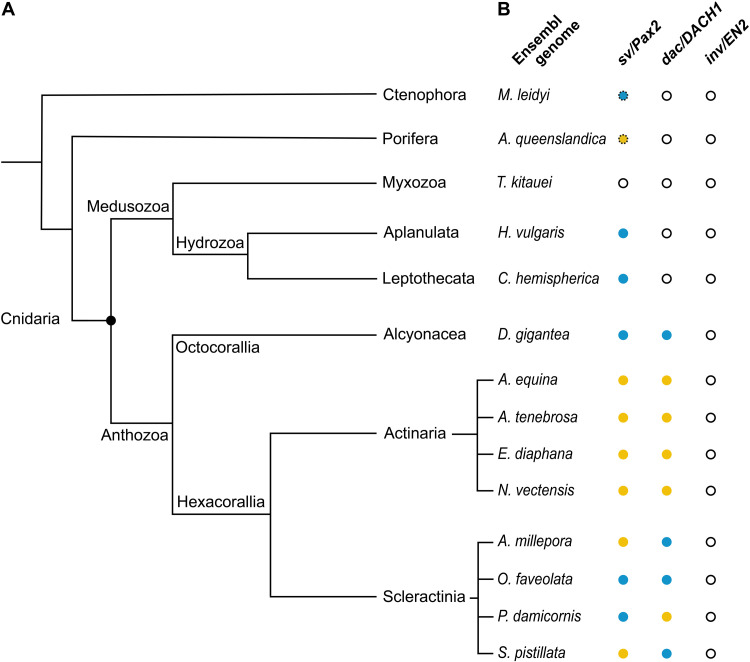
*sv/Pax2*- and *dac/DACH1*-*related* CRE-like sequences emerge in anthozoan Cnidaria. (**A**) Phylogenetic tree of nonbilaterian phyla and (**B**) select species examined, the genome sequences of which reveal the presence or absence of genes homologous to *shaven*/*PAX2* (*sv/PAX2*), *dachshund*/*DACH1* (*dac/DACH1*), and *invected/ENGRAILED-2* (*inv/EN2*) and related noncoding sequences homologous to CREs directing the formation and function of the DTB and the MHB that, respectively, give rise to adult midbrain circuitry in arthropods and vertebrates (*8*). Color code: yellow, CRE-like sequence absent; blue, CRE-like sequence present; black circle, no gene homolog; dotted circle, other Pax family member that is not a direct *sv/PAX2* or *Pax2/5/8* homolog.

To trace the phylogeny of the identified CRE-like sequences and to aid biological interpretation, we cataloged the resulting outcomes in an evolutionary tree based on genomic sampling of all metazoan phyla (*16*). However, for the phylogenetic tracing to be rooted and to apply character polarity necessary for the establishment of genealogical relationships (*17*), we first carried out an outgroup analysis investigating available nonbilaterian genomes. We focused on Cnidaria that have been recovered as the true outgroup to Bilateria by several recent phylogenomic analyses (*16*, *18*, *19*) and examined the genome sequences of 12 cnidarian species: the Medusozoa *Thelohanellus kitauei*, *Hydra vulgaris*, and *Clytia hemisphaerica* and the Anthozoa *Dendronephthya gigantea*, *Actinia equina*, *Actinia tenebrosa*, *Exaiptasia diaphana*, *Nematostella vectensis*, *Acropora millepora*, *Orbicella faveolata*, *Pocillopora damicornis*, and *Stylophora pistillata*. To further extend the outgroup analysis, we also investigated the genome sequences of the poriferan *Amphimedon queenslandica* and the ctenophore *Mnemiopsis leidyi* (Fig. 1 and table S1).

Our analyses did not recover bona fide homologs of *inv/EN2* nor *engrailed* gene homologs in any of the nonbilaterian species examined (Fig. 1 and dataset S1). For *dachshund/DACH1*, we did not detect homologs in *A. queenslandica* and *M. leidyi* and in none of the medusozoan but in all anthozoan cnidarian genomes investigated (Fig. 1, fig. S2, and table S2). However, local alignment algorithms using the conserved DTB/MHB-specific regulatory sequence as a template recovered a CRE-like motif matching the selection criteria only within the genomic sequence of the identified *D. gigantea*, *A. tenebrosa*, *A. millepora*, *O. faveolata*, and *S. pistillata* but not in the identified *A. equina*, *E. diaphana*, *N. vectensis*, and *P. damicornis dac/DACH1* homologs (dataset S1). Next, we searched for *sv/PAX2* and *Pax2/5/8* gene homologs, which, in nonbilaterian species, is considered to be *PaxB*, defined by the presence of a paired-like DNA binding domain, an octapeptide motif, and a paired-type homeobox DNA binding domain (*20*). We recovered *PaxB* gene homologs in all examined cnidarian genomes, except for *T. kitauei*, and identified *sv/PAX2* CRE-like sequences in *H. vulgaris*, *C. hemisphaerica*, *D. gigantea*, *O. faveolata*, and *P. damicornis* (Fig. 1). We also investigated the Porifera and Ctenophora *Pax* homolog as the predecessor of *PaxB* despite an absent octapeptide motif (table S2 and fig. S3) (*20*). We recovered a putative *sv/PAX2* CRE-like motif in the genomic region of the *Pax-like* gene *ML06935a* in the ctenophore *M. leidyi* but not for the *Pax-like* gene of *A. queenslandica* (Fig. 1 and dataset S1). Together, these data suggest that *sv/PAX2*- and *dac/DACH1*-related CRE-like sequences evolved in anthozoan Cnidaria that are devoid of *inv/EN2* and *engrailed* gene homologs.

### The midbrain-related gene regulatory network is absent in Xenacoelomorpha

Pre-bilaterian nervous systems are characterized by diffuse nerve nets that can exhibit elaborate condensations like the ctenophoran apical organ mediating gravitation and light sensation or a nerve ring innervating the single body opening in the cnidarian polyp *Hydra* (*21*); however, none of these resemble what could be considered a brain. Brain-like condensations of nerve cells and neuropil have been described in the head region of Acoela (*22*), which, together with Nemertodermatida and Xenoturbellida, constitute the phylum Xenacoelomorpha that either represent the sister group to all remaining Bilateria, the Eubilateria (*23*) also called Nephrozoa (*24*), or have been identified as relatives to echinoderms and hemichordates that together constitute the Xenambulacraria (*19*).

**Fig. 2.**
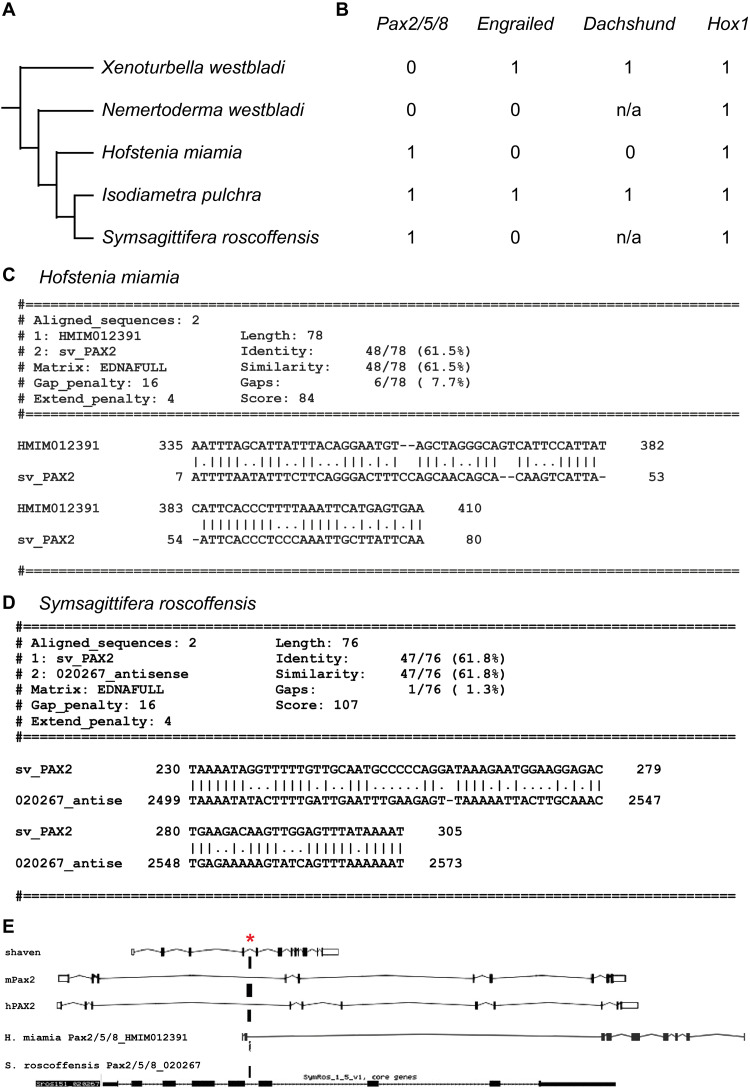
Absence of midbrain-related gene regulatory network in Xenacoelomorpha. (**A**) Phylogenetic tree of Xenacoelomorpha species, the genome sequences (*24*, *25*, *28*, *29*) of which were examined for (**B**) the presence or absence of gene homologs of *Pax2/5/8*, *engrailed*, and *dachshund* that are involved in the development and specification of the insect DTB and vertebrate MHB (*8*); *Hox1* homologs are included to illustrate presence call. Results shown for 0 = no homolog; 1 = one gene homolog; and n/a, no sequence information available. (**C**) EMBOSS Matcher pairwise alignment of *H. miamia Pax2/5/8* gene homolog, *HMIM012391*, revealing identified *shaven/PAX2* CRE-like sequence. (**D**) EMBOSS Matcher pairwise alignment of *S. roscoffensis Pax2/5/8* gene homolog, *020267*, showing identified *shaven/PAX2* CRE-like sequence. (**E**) Intragenic location (red asterisk) of *shaven/PAX2*-specific CRE element in *Drosophila shaven*, mouse *Pax2* (mPax2), and human *PAX2* (hPax2) and homologous CRE-like sequences in intronic region of *H. miamia Pax2/5/8* gene, *HMIM012391*, and *S. roscoffensis Pax2/5/8* gene homolog, *020267*.

To determine the presence or absence of DTB/MHB-related CRE-like sequences, we first examined the genome of the acoel *Hofstenia miamia* (*25*) that is available for navigation at Ensembl Metazoa. The cephalic nervous system of *H. miamia* is characterized by an anterior condensation containing neurite bundles together with a ring commissure and ventral lobe–like structures (*26*). BLAST searches using *Drosophila*, mouse, and human gene and protein sequences did not detect homologs of *engrailed/invected/EN* or homologs of *dac/DACH1* (Fig. 2, A and B). However, we detected a *Pax2/5/8* homolog (table S2) for which local alignment algorithms, using the conserved DTB/MHB-specific regulatory sequence of *sv/PAX2*, recovered a CRE-like motif within the intronic sequence of the identified *H. miamia HMIM012391* gene homolog (Fig. 2, C and E, and dataset S2).

Compared to *H. miamia*, the acoelomorph *Isodiametra pulchra* shows a more complex cephalic nervous system with a bilobed brain, cellular cortex, and dense internal neuropil with anterior commissures (*27*). Analysis of published genome sequences (*24*, *28*) revealed the presence of putative *engrailed*, *Pax2/5/8*, and *dac/DACH1* homologs in *I. pulchra* (Fig. 2B and dataset S2). In comparison, the phylogenetically older xenoturbellid *Xenoturbella westbladi* lacks a *Pax2/5/8* homolog but contains coding sequences indicative of *engrailed* and *dac/DACH1* homologs (Fig. 2, A and B, and dataset S2). However, except for *H. miamia*, the incomplete genome annotations of *I. pulchra* and *X. westbladi* are currently devoid of intronic and intergenic regions and thus preclude the further analysis of DTB/MHB-related CRE-like sequences. Further differences were found for the published coding sequences of the nemertodermatide *Nemertoderma westbladi* for which no *shaven/PAX2*, *dachshund/DACH1*, and *invected/ENGRAILED-2* homologs could be identified (Fig. 2B). Similarly, we could not identify *dachshund/DACH1* and *invected/ENGRAILED-2* or any bona fide *engrailed* homologs in the recently annotated genome of the acoelomorph *Symsagittifera roscoffensis* (*29*), except the *Pax2/5/8* homolog 020267 for which local alignment algorithms, using the conserved DTB/MHB-specific regulatory sequence of *sv/PAX2*, recovered a CRE-like motif within its intronic sequence (Fig. 2, D and E, and dataset S2). Together, these findings suggest the multiple gain and loss of *sv/PAX2*, *dac/DACH1*, and *inv/EN2* homologs and the absence of a DTB/MHB-related gene regulatory network in Xenacoelomorpha.

### *sv/PAX2*, *dac/DACH1*, and *inv/EN2* CRE-like sequences are present in Eubilateria that have a brain

The lack of a DTB/MHB-related gene regulatory network in Xenacoelomorpha could be a testimony of their phylogenetic position as either an early offshoot from the last common bilaterian ancestor or simplified relatives of echinoderms and hemichordates (Xenambulacraria) and, thus, the result of evolved loss, including morphological characters like a through-gut. We therefore investigated the presence or absence of a DTB/MHB-related gene regulatory network in protostomes and deuterostomes and examined whether its presence/absence relates to the occurrence of a brain. To do so, we screened the genome sequences of Chordata, Ambulacraria, Spiralia, and Ecdysozoa for gene homologs (figs. S4 to S6 and table S2) and CRE-like sequences of *sv/PAX2*, *dac/DACH1*, and *inv/EN2* (datasets S3 to S5) and related it to the organization of their cephalic nervous system (table S1). We found that all eubilaterian genomes examined contain at least one gene homolog of *sv/Pax2*, *dac/DACH1*, and *inv/EN2*, with two exceptions. The genome of the tardigrade *Hypsibius exemplaris* is devoid of a *dac/DACH1* gene homolog (*30*), and we were unable to identify a *dac/DACH1* homolog in the genome of the priapulid *Priapulus caudatus*.

**Fig. 3.**
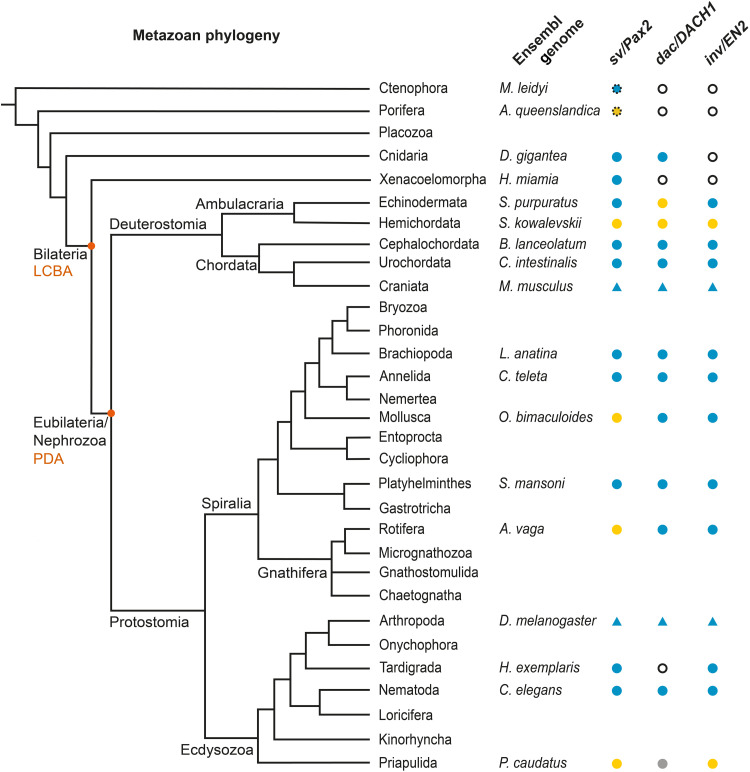
Phylogenetic tracing of *sv/PAX2*, *dac/DACH1*, and *inv/EN2* CRE-like sequences across sequenced metazoan genomes. Phylogenetic tree based on genomic sampling of all metazoan phyla (*16*), with Cnidaria as true outgroup to Bilateria and Xenacoelomorpha as sister group to the remaining Eubilateria/Nephrozoa; key nodes (orange dot) identify last common bilaterian ancestor (LCBA) and last common ancestor of Protostomia and Deuterostomia (PDA). Matrix on the right, presence/absence call within species-specific genomes (Ensembl genome; see table S1) for DTB- and MHB-specific CREs of *shaven* and *PAX2* (*sv/PAX2*), *dachshund* and *DACH1* (*dac/DACH1*), and *invected/ENGRAILED-2* (*inv/EN2*) that mediate midbrain circuit formation and function in insects and vertebrates, respectively (*8*). Symbol and color code: blue triangle, confirmed functional CRE (*8*); black circle, no gene homolog present; yellow circle, gene homolog identified but CRE-like sequence absent; blue circle, CRE-like sequence identified; gray circle, not accessible; dotted circle, other Pax family member that is not a direct *sv/Pax2* or *Pax2/5/8* homolog.

Analysis of the chordate sister group Ambulacraria revealed *sv/PAX2* and *inv/EN2* but not *dac/DACH1* CRE-like sequences in the genome of the echinoderm *Strongylocentrotus purpuratus* (Fig. 3 and table S2), whose ambulatory larval stage is characterized by a cephalic nervous system composed of an apical organ and its related ganglion together with two paired oral ganglia and neuropil surrounding the mouth region (*31*). In contrast, our analysis did not recover *sv/PAX2*, *dac/DACH1*, or *inv/EN2* CRE-like sequences in the genome of the Hemichordate *Saccoglossus kowalevskii* (Fig. 3 and table S2), the cephalic nervous system of which consists of a nerve net connected to a posterior collar chord and caudal condensations into a dorsal and ventral nerve cord (*32*). Further analysis recovered *sv/PAX2*, *dac/DACH1*, and *inv/EN2* CRE-like sequences in the genomes of the cephalochordate *Branchiostoma lanceolatum* and the urochordate *Ciona intestinalis* (Fig. 3 and table S2), the cephalic nervous systems of which are characterized by a tripartite brain in the motile tadpole larva of *C. intestinalis* (*33*) and a genoarchitectonically regionalized cerebral vesicle in *B. lanceolatum* (*34*). The identified CRE-like sequences exhibit homologies to the regulatory sequences identified in the mouse and human genome (*8*), suggesting their presence in all chordate lineages.

Within the spiralian clade, we detected a full complement of all three DTB/MHB-related CRE-like sequences in the brachiopod *Lingula anatina* (Fig. 3 and table S2), the motile, planktotrophic larva of which exhibits a cephalic nervous system including bilobed circumesophageal ganglia with nerves innervating an apical organ, a median tentacle, and surrounding lophophores (*35*). Similarly, a full complement of homologous *sv/PAX2*, *dac/DACH1*, and *inv/EN2* CRE-like sequences were recovered in both the annelid *Capitella teleta* (Fig. 3 and table S2), which is characterized by a dorsal brain comprising several fused cerebral domains and a segmented, ganglionated ventral nerve cord (*36*, *37*), and the platyhelminth *Schistosoma mansoni* with a central nervous system composed of a cephalic ganglion from which nerve trunks extend (*38*). The remaining two spiralian genomes examined all reveal the same pattern (Fig. 3 and table S2), with *dac/DACH1* and *inv/EN2* but not *sv/PAX2* CRE-like sequences present in the mollusk *Octopus bimaculoides* with its elaborate and highly organized cephalic nervous system comprising a circumesophageal brain, paired optic lobes, and axial nerve cords in each arm (*39*) and the rotifer *Adineta vaga* with its brain consisting of a paired ganglion located dorsal to the mastax and two main nerve cords elongating caudally from which neurites branch off that innervate the body musculature (*40*).

For the ecdysozoan genomes investigated, CRE-like sequences were detectable in the genomic region of *sv/Pax2* and *inv/EN2* homologs of the nematode *Caenorhabditis elegans* (Fig. 3 and table S2), the cephalic nervous system of which is characterized by a circumesophageal nerve ring from which a ventral and a less prominent dorsal nerve cord extend caudally (*41*). We also identified *sv/Pax2* and *inv/EN2* homologs of the tardigrade *H. exemplaris*, the brain of which is composed of lobed ganglia that are connected to the paired nerve cord with paired segmental ganglia via outer and inner connectives (*42*). We identified DTB/MHB-related CRE-like sequences for both gene homologs (Fig. 3 and table S2) but not for *dac/DACH1* for which no gene homologs have been detected in the tardigrade genomes examined so far. Furthermore, we did not recover CRE-like motifs in the genomic region of the *Pax2/5/8* and *engrailed* gene homologs of the priapulid *P. caudatus* (Fig. 3 and table S2), the cephalic nervous system of which consists of a circumpharyngeal nerve ring comprising neuropil and two somata aggregations and a caudal ventral nerve cord with neck and caudal ganglion (*43*).

### Conserved structural elements characterize CRE-like sequences

**Fig. 4.**
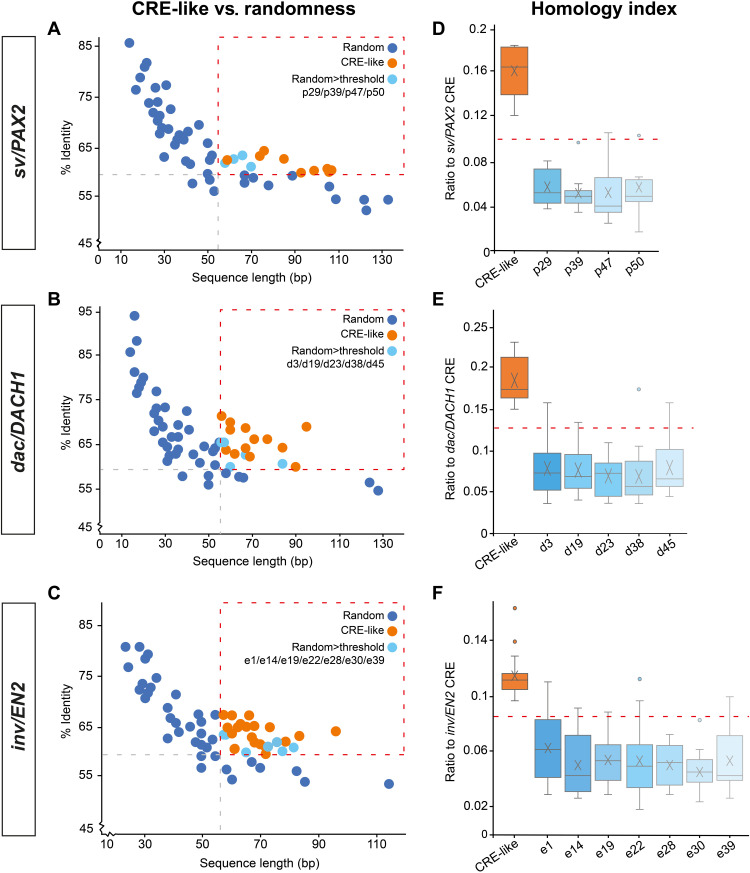
Conserved structural elements characterize *sv/PAX2*, *dac/DACH1*, and *inv/EN2* CRE-like sequences. (**A** to **C**) CRE-like sequences (orange; see datasets S3 to S5) and random sequences p1 to p50, d1 to d50, and e1 to d50 (blue; see dataset S6) plotted according to their sequence length and percentage identity when matched in local alignments against the *sv/Pax2*, *dac/DACH1*, and *inv/EN2* CRE, respectively; light blue dots indicate randomly generated sequences that meet the threshold of 60% sequence identity over 55 bp (indicated as red dashed box). (**D** to **F**) HI defined as a function of sequence length and percentage sequence identity related to the respective CRE sequence of *sv/PAX2*, *dac/DACH1*, and *inv/EN2*. Comparison of the average HI of the identified CRE-like sequences (orange) with the HI of each of the random sequences (blue), measured against the minimal HI (dashed red line) to be considered significant; *sv/PAX2* HI_min_ = 0.092; *dac/DACH1* HI_min_ = 0.133; *inv/EN2* HI_min_ = 0.088. Note that each HI of the random sequences is below the respective HI_min_ and significantly lower than the HI of CRE-like sequences.

The detected CRE-like sequences are positioned within noncoding regions of homologous genes. Hence, their common appearance, unique genomic location, and nucleotide identities could be interpreted as phylogenetic traces of genealogical relationships among Eubilateria that have a brain. Alternatively, the recovered sequences could be the mere result of stochastic events and thus evolved independently and de novo, with no relation to the conserved regulatory sequences mediating midbrain circuit formation and function in arthropods and vertebrates (*8*). To test this hypothesis, we developed code to generate a set of 50 random sequences with similar length for each of the three conserved DTB/MHB-specific CRE (356 bases for *sv/PAX2*, 247 bases for *dac/DACH1*, and 373 bases for *inv/EN2*), with a CG:AT ratio of 40:60 to mimic the average genomic distribution of nucleotides (dataset S6). Using EMBOSS Matcher, each random sequence was aligned with the respective consensus CRE sequence identified in *Drosophila*, mouse, and human (fig. S1C) to determine whether they match the threshold criteria of minimum 60% sequence identity over at least 55 bp with minimum 1 × 10^−1^ confidence level (data file S1). Of the 50 random sequences related to *sv/PAX2*, 4 were retrieved with EMBOSS matcher results above threshold (Fig. 4A); of the 50 random sequences related to *dac/DACH1*, 5 were retrieved above threshold (Fig. 4B), and of the 50 random sequences related to *inv/EN2*, 7 were retrieved above threshold (Fig. 4C). Thus, up to 14% of the random sequences return EMBOSS matcher results that resemble those of the identified CRE-like sequences.

Next, we tested whether the random sequences with EMBOSS matcher results above threshold have structural features corresponding to the CRE sequences identified in *Drosophila*, mouse, and human. We therefore determined their homology index (HI) as a function of sequence length and percentage sequence identity related to the respective CRE sequence of *sv/PAX2*, *dac/DACH1*, and *inv/EN2*, which was calculated as

HI=(sequence-length/length-of-conserved-CRE×%-sequence-identity)/100

For example, in the case of the conserved CRE sequence for *sv/PAX2*, the HI calculates as 356/356 × 100%/100, resulting in an HI of 1. Accordingly, we calculated the HI for all random sequences above threshold (data file S2) and compared them to the average HI of the identified CRE-like sequences. This revealed an HI for each of the random sequences that was substantially lower than the HI of the identified CRE-like sequences (Fig. 4, D to F, and data file S2). Each of the random sequence HIs was below the minimal HI to be considered significant, which, in the case of *sv/PAX2*, is HI_min_ = 0.092 (as calculated 55/356 × 60%/100), is HI_min_ = 0.133 (55/247 × 60%/100) for *dac/DACH1*, and is HI_min_ = 0.088 (as calculated 55/373 × 60%/100) for *inv/EN2*, whereas the HI of the identified CRE-like sequences was well above the respective HI_min_ in all three cases (dashed red lines in Fig. 4, D to F). These data distinguish the identified CRE-like sequences from random sequences, suggesting that their unique genomic location and nucleotide identities are unlikely the result of stochastic events.

To characterize the recovered CRE-like sequences in further detail, we compared them among each other to investigate whether they share a common or core domain, using as reference the consensus sequences identified for the *Drosophila*, mouse, and human DTB/MHB-specific CREs of *sv/Pax2*, *dac/Dach1*, and *inv/En2*. Our comparative analysis revealed that most of the identified CRE-like sequences cluster within a consensus region specific for *sv/Pax2*, *dac/Dach1*, and *inv/En2* CREs (fig. S7, A to C, red dashed box), which identifies the presence of a conserved core domain that characterizes midbrain-related CREs of eubilaterian species that have a brain. In addition, we applied the CiiiDER toolkit for the prediction and study of transcription factor binding sites (TFBSs) (*44*). Except for the *sv/PAX2* CRE-like sequences of *O. bimaculoides* and *A. vaga* and the *dac/DACH1* CRE-like sequence of *S. purpuratus*, we identified several putative TFBSs in all CRE-like sequences within the genomic loci of the eubilaterian genomes examined (table S3 and data file S3). Notably, the predicted TFBS include transcription factors, including *otd/Otx*, *unpg/Gbx*, *ems/Emx*, *vnd/Nkx*, *msh/Msx*, *inv-en/EN*, *Pax2*, and *Ptf1a* (table S3), that are all implicated in the development of the DTB/MHB and the specification of adult midbrain circuits in arthropods and vertebrates (*8*).

Note, however, that the CiiiDER algorithm also predicts a small number of putative TFBSs for the random sequences with EMBOSS matcher results above threshold, which is accountable for the short length of TFBS motifs (4 to 6 nucleotides) and the likelihood of their occurrence in sequences composed of A, T, G, and C with a minimum length of 55 bp. We therefore further corroborated the significance of the identified elements and investigated the presence or absence of *sv/PAX2* and *dac/DACH1* CRE-like sequences in the non-neural genes *Brachyury*, *GATA*, and *Twist* of the species *N. vectensis*, *S. kowalevskii*, *C. intestinalis*, *L. anatina*, *O. bimaculoides*, *S. mansoni*, *A. vaga*, *H. exemplaris*, and *P. caudatus* (datasets S3 and S4). We did not identify *sv/PAX2* or *dac/DACH1* CRE-like sequences in *Brachyury*, *GATA*, and *Twist* gene homologs in any of the 9 investigated species covering Cnidaria, Chordata, Spiralia, and Ecdysozoa (54 test cases in total), except for the *Brachyury* gene homolog of *O. bimaculoides*, which revealed a putative *sv/Pax2* CRE-like sequence (dataset S3).

Last, we investigated whether the identified CRE-like sequences are accessible chromatin regions. For this, we made use of available assay for transposase-accessible chromatin using sequencing (ATAC-seq), which can determine transcriptionally active sites in genomic DNA that are used as approximation for enhancer usage and CRE activity. We first examined ATAC-seq data of the larval stages of *C. intestinalis* that are available at the Aniseed browser (https://anis-server.aniseed.cnrs.fr/browser/) and the Ghost Database (http://ghost.zool.kyoto-u.ac.jp/default_ht.html). We focused on larval stage 10 hpf (hour post fertilization) during brain formation and examined the available ATAC-seq data directed by the genomic location of the identified *sv/PAX2*, *dac/DACH1*, and *inv/EN2* CRE-like sequences in *C. intestinalis.* Using the BLAST algorithm to locate the identified CRE-like sequences, we detected ATAC-seq signals for *sv/PAX2* (fig. S8, A and B), *dac/DACH1* (fig. S8, C and D), and *inv/EN2* (fig. S8, E and F) that, in each case, are distinguishable compared to background (see yellow column in fig. S8, B, D, and F, respectively). We also examined the available *C. elegans* regulatory atlas v0.5.4. (https://ahringerlab.com/RegAtlas/), focusing on neuron-specific ATAC-seq signals for the identified *sv/PAX2*, *dac/DACH1*, and *inv/EN2* CRE-like sequences (fig. S9, A to H). In contrast to the data observed in *Ciona*, the ATAC-seq signals spanning the *C. elegans* CRE-like sequences were less prominent (fig. S9, B, D, F, and H). However, note that only very few neurons express *sv/PAX2*, *dac/DACH1*, and *inv/EN2* homologs in *C. elegans* (see below; table S8), which may account for the low-profile ATAC-seq signals. Together, these findings reveal conserved structural elements with the identified CRE-like noncoding sequences of homologous *sv/PAX2*, *dac/DACH1*, and *inv/EN2* genes in eubilaterian species that have a brain.

### A conserved genetic boundary demarcates the anterior brain in Eubilateria

**Fig. 5.**
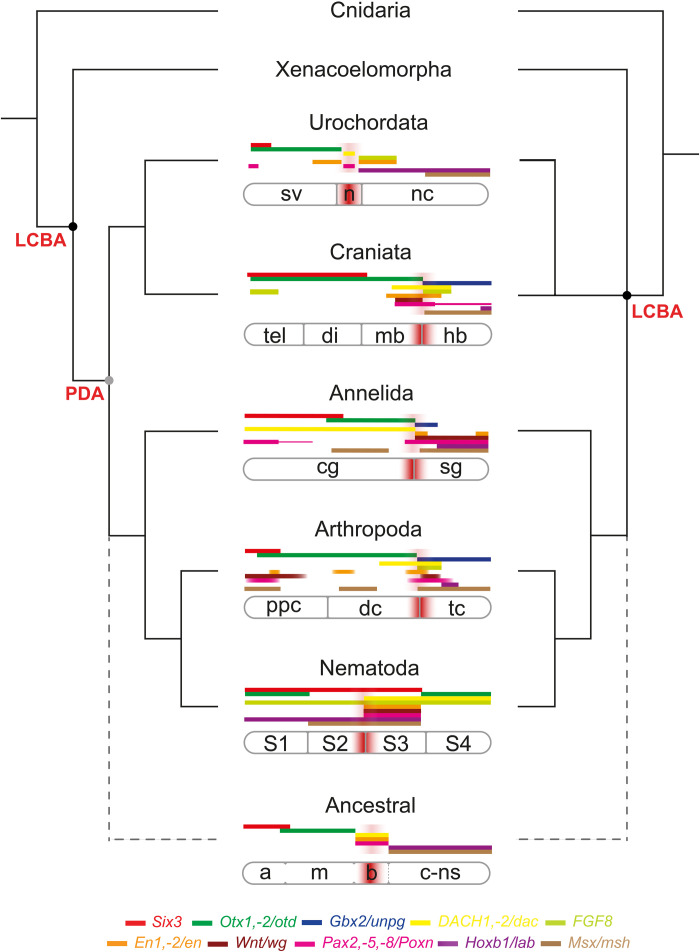
A conserved genetic boundary demarcates the anterior brain in Eubilateria. Brain-specific expression patterns of gene homologs for *Six3* (red), *Otx* (green), *unpg/Gbx* (blue), *Dachshund/Dach1* (yellow), *FGF 8/17/18* (light green), *Engrailed* (orange), *Wnt* (dark brown), *Pax2/5/8* (pink), *Hox1* (purple), *Msx* (light brown) shown for select phyla with identified DTB/MHB-related CRE-like sequences. Common to all is a rostral part defined by *Six3*, an intermediate region defined by *Otx*, and a caudal boundary defined by *sv/PAX2*, *inv/EN2*, and *dac/DACH1* gene expression domains, which together distinguish the anterior brain from the genetically distinct caudal nervous system. Polarized against Cnidaria as outgroup to Bilateria, phylogenetic interpretations depict Xenacoelomorpha either as sister group (left) to the remaining Bilateria (equal to Eubilateria, also called Nephrozoa), or as sister group (right) to Chordata (here, only Urochordata and Craniata are shown), suggesting that the gene regulatory network underlying anterior brain formation was already present in the last common ancestor of Protostomia and Deuterostomia (PDA, gray node) or the last common ancestor of Bilateria (LCBA, black node), the brain of which is hypothesized to consist of three genetically distinguishable domains; an anterior (a), a middle (m), and boundary (b) domain that together are distinct from the caudal nervous system (c-ns). n, neck; nc, nerve cord; tel., telencephalon; di, diencephalon; mb, midbrain; hb, hindbrain; cg, cerebral ganglion; sg, segmental ganglia; ppc, proso and protocerebrum; dc, deutocerebrum; tc, tritocerebrum; S1–S4, brain-specific stratum 1 to 4.

The insect DTB and vertebrate MHB delineate a boundary between two genetically distinct regions of the nervous system, the anterior brain, and its connected caudal nervous system (*8*). Given the above findings, we hypothesized that the presence of *sv/PAX2*, *dac/DACH1*, and *inv/EN2* CRE-like sequences concurs with a genetically defined boundary region that together characterize the organization of eubilaterian brains. To test this hypothesis, we compared their presence with the neural architecture of developing brains in select species of all three bilaterian clades. We focused on the urochordate *C. intestinalis* (*45*, *46*), the annelids *C. teleta* (*47*) and *Platynereis dumerilii* (*48*), and the cycloneuralian *C. elegans* (*41*, *49*) and analyzed the expression pattern of genes shown to regulate anterior brain and DTB/MHB formation in insects and vertebrates (*8*, *13*), including *Six3*, *Otx*, *Gbx*, *FGF8*, *Wnt1*, *En1/2*, *Pax2*, *Dach1*, *Msx*, and *Hoxb1* homologs (Fig. 5).

Analysis of single-cell RNA sequencing (RNA-seq) data available for the tadpole larva of *C. intestinalis* identified 9 different cell types (*46*) in the anterior sensory vesicle (SV), three clusters of cells of the posterior SV, one specific for the equivalent of the MHB (*33*), and one neck region cluster [neck neurons: ID 165 and 166; see (*50*)], as well as three motor neuron–specific clusters and two nerve cord clusters in each, the trunk and the tail (fig. S10, A to C). Gene expression patterns (*33*, *45*, *46*, *51*) mapped onto these cell clusters (table S4) recovered *Six3/6* expression exclusively in the pro–anterior SV (pro-aSV), *Otx* throughout the entire SV, *PAX2/5/8A* in the anterior neural boundary, as well as in the neck region, and *Pax2/5/8B* only in the pro–anterior vesicle. *Dachshund* was found to be expressed in only one cluster of the neck region, whereas *Msx* and *Hox1* expression are restricted to the nerve cord of the trunk. Together with cell type– and region-specific *engrailed* and *FGF8/17/18* expression (fig. S10D), the observed gene expression patterns identify cellular subdivisions that distinguish the anterior brain and its caudal boundary region in the tadpole larva of *C. intestinalis* (fig. S10).

Gene expression analysis of the larval annelid *C. teleta* (fig. S11A) identified a rostrolateral expression of the *Otx* gene homolog absent from the rostral-most part of the developing anterior brain (*52*), as is the case for the *FGF8/17/18* homolog (*53*). Expression of *Wnt* and *engrailed* gene homologs delineate the posterior domain of the anterior brain that is genetically distinct and develops decoupled from the caudal nerve cord (*37*), which is characterized by *Hox1* expression (*47*). However, gene expression patterns of *Six3*, *Gbx*, *Pax2*/5/*8*, *dachshund/Dach1*, and *Msx* homologs have not been described in *C. teleta* so far. We therefore explored gene expression patterns of the larval annelid *P. dumerilii* (*54*, *55*) together with single-cell RNA-seq data (*48*). These data identify staggered expression patterns of *Six3*, *Otx*, *Gbx*, *engrailed*, and *Hox* gene homologs in the developing anterior brain of *P. dumerilii* (fig. S11B). Furthermore, RNA-seq data recovered ubiquitous expression of *Dachshund* throughout the brain, localized especially to the mushroom bodies and the frontal ganglion, *Pax2/5/8* in the anterior ganglion and coexpressed with *Six4* and *Dachshund*, *Wnt5* in all ganglia, most notably in the frontal and palpal ganglia and the mushroom bodies, *Msx* with weak expression levels throughout the brain ganglia, and *Hox1* in the cirral ganglion, where it is the predominant gene (fig. S11B and tables S5 and S6). Together, these data identify gene expression territories that delineate domains and cellular compartments that distinguish the anterior brain and its caudal boundary region in the developing nervous system of *C. teleta* and *P. dumerilii*.

For the analysis of the larval and adult nervous system of the cycloneuralian *C.* elegans, we combined available RNA-seq data with connectome data at single-cell and circuit resolution (*41*, *56*–*58*). The nerve ring of *C. elegans* is organized into four functionally distinct strata (S1 to S4) and three distinct layers (*41*, *56*, *58*) that largely separate sensory and motor afferents and efferents (fig. S12, A to C, and table S7) (*56*, *58*). We used the CeNGEN project online tool (https://cengen.shinyapps.io/CengenApp/) to retrieve gene expression data at single-cell resolution (*57*) and combined them with cellular projection patterns covering the entire connectome. This resolved a brain-specific gene expression atlas for each of the investigated homologs (table S8 and fig. S12D) and recovered *Otx* expression in strata S1 and S4, *Six3* and *Hox1* covering strata S1 to S3, and expression of the *FGF* homolog throughout the entire ring neuropil from strata S1 to S4. *Msx* expression is detected in cells projecting to strata S2 and S3, while expression of the *dachshund* homolog is limited to strata S3 and S4, whereas the expression patterns of the *Pax2*, *Engrailed*, and *Wnt* homologs are restricted solely to stratum S3 (fig. S12). Together, these gene expression patterns reveal a strata- and layer-specific compartmentalization of the asegmental brain of *C. elegans*. When compared with data available for *Drosophila melanogaster* and *Mus musculus* (*8*) and concurrent with the presence of CRE-like sequences, these findings identify a genetic boundary that separates the anterior brain from its connected caudal nervous system in arthropods and vertebrates (*8*), as well as in the cycloneuralian *C. elegans*, the urochordate *C. intestinalis*, and the annelids *C. teleta* and *P. dumerilii* (Fig. 5).

## DISCUSSION

The present study established the phylogenetic tracing of highly conserved cis-regulatory sequences directing the expression of homologous developmental control genes to respective cells of the DTB region and the MHB that give rise to corresponding midbrain circuits in arthropods and vertebrates (*8*). We used the nucleotide sequences of these conserved CREs for a comparative phylogenomic analysis to trace their presence or absence in sequenced genomes of eumetazoan species representing all clades of the animal kingdom. A polarized outgroup analysis established that fully sequenced poriferan, ctenophoran, cnidarian, and Xenacoelomorpha genomes are devoid of the entire set of homologous *sv/PAX2*, *dac/DACH1*, and *inv/EN2* CRE-like sequences, which, together, were only detected in the genomes of eubilaterian/nephrozoan species that have a brain.

Our findings reveal the emergence of *sv/PAX2* and *dac/DACH1* CRE-like sequences in anthozoan Cnidaria, the gene homologs of which have been implicated in the formation of region-specific identity and patterning of the ectoderm (*59*). Cnidaria are characterized by an ectoderm-derived nerve net with varying degrees of organization and condensation along the anterior-posterior axis, the genetic determination of which resembles correspondences to axial patterning in Bilateria (*59*). These findings may indicate that *sv/PAX2* and *dac/DACH1* CRE-like sequences in Cnidaria have been co-opted for the independent evolution of cephalic nervous systems in Bilateria. However, recent cross-species comparison of single-cell RNA-seq data across *Homo sapiens*, *M. musculus*, *Danio rerio*, *C. intestinalis*, *C. elegans*, and *N. vectensis* trace neuron cell type evolution to a single origin (*60*). Instead of co-option, these data suggest a conserved regulatory logic for the region-specific determination of the bilaterian nervous system that likely evolved from a cnidarian ancestor or from an anthozoan-bilaterian ancestor. Consistent with this interpretation, comparative genomics analyses of 124 million sequences from 21 metazoan and three outgroup phyla reveal a common genetic basis for bilaterian nervous system patterning (*61*). This interpretation is independently supported by a comparative phylogenetic transcriptome analysis, which identified a conserved genetic program underlying head patterning (*35*) that originated in the lineage of the last common ancestor of Protostomia and Deuterostomia (PDA).

The alternative interpretation of convergent evolution suggests the independent origins of bilaterian brains in multiple lineages, including chordates, brachiopods, annelids, molluscs, panarthropods, and nematodes. Juxtaposition of both concepts, convergence versus common decent, applied to the acquired data and a phylogeny derived from genomic sampling of all metazoan phyla (Fig. 3) lead to predictions that a single origin implies at least two losses in hemichordates and priapulids, whereas convergent evolution within ecdysozoan, spiralian, and deuterostome lineages implies at least six independent gains of a midbrain-related gene regulatory network. A prerequisite of the latter hypothesis is the independent acquisition of brain-specific regulatory networks. However, our structural analysis revealed substantial differences to randomly assembled sequences and their homology index (Fig. 4), which makes the de novo acquisition of the identified *sv/Pax2*, *dac/DACH1*, and *inv/EN2* CRE-like sequences less likely. On the contrary, their genomic location and nucleotide identities, together with the presence of a conserved core domain and the observed ATAC-seq data in *C. intestinalis* and *C. elegans*, signify them as phylogenetic traces of genealogical relationships among Eubilateria that have a brain. Moreover, when polarized against nonbilaterian outgroups and Xenacoelomorpha, the available data identify the midbrain-related gene regulatory network as a eubilaterian innovation of which sequence homologs are detectable in spiralian, ecdysozoan, and chordate genomes. The principle of parsimony, which favors phylogenetic interpretations that require the fewest implied character state transformations, thus leads to the conclusion of a single origin within the lineage that became the PDA, that is, the crown ancestor of Eubilateria.

In support of this conclusion, we detected a full complement of CRE-like sequences across eubilaterian species, the comparative analysis of which identifies genetic traits common to all Eubilateria that have a brain (Fig. 5). These traits distinguish the anterior brain from the genetically distinct caudal nervous system, regulated by distinct developmental genetic mechanisms underlying brain versus nerve cord formation reported for annelids (*37*), arthropods (*42*), and vertebrates (*12*). Our conclusion is consistent with either phylogenetic position of Xenacoelomorpha, at the base of Bilateria (*24*) or as relatives of Hemichordata and Ambulacraria (*19*), but leads to different predictions: Their interpretation as part of Xenambulacraria implies the recurrent evolved loss of *Pax2/5/8*, *engrailed*, or *dachshund* genes (Fig. 2), whereas their phylogenetic position at the base of Bilateria denote the emergence of a DTB/MHB-related gene regulatory network within the lineage that became the PDA. Both interpretations of Xenacoelomorpha phylogeny imply secondary reduction and evolved loss of phenotype (*62*) that also characterizes the metamorphosis of developing urochordates during which the brain of the tadpole larva degenerates to give way to an adult nervous system that supports the sessile lifestyle of a filter-feeding animal (*21*).

Our analysis of the recovered CRE-like sequences suggests that secondary reduction and evolved loss also applies to hemichordates, irrespective of the phylogenetic position of Xenacoelomorpha. We retrieved genomic sequences associated with noncoding regions of *sv/Pax2*, *dac/DACH1*, and *inv/EN2* homologs in *S. kowalevskii* that are close but below the threshold criteria, suggesting their modification from an ancestral set of regulatory elements. This interpretation is consistent with an interphyletic outgroup analysis polarized either against chordates or against Protostomia, both of which reveal the presence of an entire set of regulatory elements at the base of each of the two branches (Fig. 3). Our interpretation is also compatible with earlier findings that reported mutually exclusive *Engrailed* and *Pax2/5/8* expression topologies in the collar of *S. kowalevskii* (*10*). In insects and vertebrates, however, *Engrailed* and *Pax2/5/8* homologs show overlapping and interdependent expression topologies that are required, respectively, for the formation and function of the DTB/MHB and their specification into adult midbrain circuits (*8*). These divergences in gene regulation and expression topologies also extend to *Otx* and *Gbx* homologs, the juxtaposition of which in hemichordates is part of the collar (*10*), whereas in arthropods and vertebrates, it positions the DTB/MHB, respectively (*8*). These differences together with an interphyletic outgroup analysis suggest an evolved modification of the DTB/MHB-related gene regulatory network in hemichordates that is no longer required for the stationary lifestyle of suspension-feeding animals like *S. kowalevskii* (*21*).

To corroborate not only the significance but also the limitations of our findings, future experiments will help determine whether the identified CRE-like sequences direct the expression of *sv/PAX2*, *dac/DACH1*, and *inv/EN2* homologs to, and are required for the formation and function of, the genetic boundary region between the anterior brain and caudal nerve cord in the eubilaterian species examined. While we cannot exclude the possibility that molecular divergences may obscure the function of the detected CRE-like sequences, the conclusions we have reached are in accord with the fossil record recovered from the early Cambrian. Analysis of the megacheiran arthropod *Alalcomenaeus* (Chengjiang and Burgess Shale, 515 My ago) revealed an organization of its anterior brain that corresponds to that of extant arthropods (*1*). In the polychaete annelid *Canadia spinosa* (Burgess Shale, 508 My ago), a terminal brain has been described that is connected via two circumoral nerves to a midventral ganglionated nerve cord (*2*), which together resemble the organization of the central nervous system seen in extant annelids (*36*). The brain of *Haikouella* (Maotianshan Shale, 520 My ago) reveals a diencephalon situated rostral to its hindbrain and neural cord (*3*), a relative order and neural arrangement also seen in the brain of extant vertebrates (*11*). All three fossil nervous systems share a common architecture characterized by an anterior brain in front of or above a mouth opening and connected to a caudal nerve cord that extends posteriorly from the “neck” region of the animal. In *Canadia* and *Alalcomenaeus*, the anterior brain is distinguishable from the caudal nerve cord by two circumoral connectives, while in *Haikouella*, this transition pertains to the hindbrain located dorsal to the branchial arches. Despite considerable differences between *Alalcomenaeus*, *Canadia*, and *Haikouella*, a common ground pattern can be recognized that corresponds to the genetic organization of extant spiralian, ecdysozoan, and chordate brains, suggesting that these traits are contingent on ancestral gene regulatory networks that evolved in the crown ancestor of Eubilateria.

## MATERIALS AND METHODS

### Experimental design

All analyses were based on the three recently identified, highly conserved CREs of *sv/Pax2*, *dac/Dach1*, and *inv/EN2* that direct DTB/MHB-related gene regulatory networks mediating the formation and function of corresponding midbrain circuits in arthropods and vertebrates (*8*). Phylogenetic screening for presence/absence calls was performed on the basis of criteria that have been used previously to identify transphyletic cis-regulatory DNA sequences (*63*). The applied criteria were as follows: (i) The sequences are linked to the same homologous genes in the different species. (ii) There is a minimum of 60% sequence identity over at least 55 bp with minimum 1 × 10^−1^ confidence level as the BLAST *e*-value. (iii) The CREs are noncoding and not un-annotated protein sequences. (iv) The CREs are not repetitive elements.

Because the BLAST algorithm does not support the first stated threshold criterion, phylogenetic screening was restricted to genomic regions of identified gene homologs, which could include more than one homolog (e.g., *DACH1* and *DACH2*). In the case of *inv/EN2*-related CRE-like sequences, intergenic regions 5 kb upstream and downstream of the genomic region of the respective gene homologs were analyzed (*8*).

### Identification and phylogeny of gene homologs

The respective *D. melanogaster–* and *H. sapiens*–specific protein sequences for *shaven/PAX2*, *dachshund/DACH1*, and *invected/ENGRAILED-2* were retrieved from NCBI and used as templates to apply BLAST searches to screen available genome sequences (table S1) annotated at Ensembl (www.ensembl.org/index.html), at Ensembl Metazoa (https://metazoa.ensembl.org/index.html), or at NCBI (www.ncbi.nlm.nih.gov/genome/) to determine the presence or absence of gene homologs in the species examined (table S2). In case of multiple hits, the significance of a potential gene homolog was scrutinized by protein sequence comparison using local alignment algorithms including Kalign (www.ebi.ac.uk/Tools/msa/kalign/) and Clustal Omega (www.ebi.ac.uk/Tools/msa/clustalo/). To further establish the correspondence of the identified candidates, a multiple sequence alignment was carried out using Multiple Sequence Comparison by Log-Expectation (www.ebi.ac.uk/Tools/msa/muscle/). Homologous proteins were then verified by manually identifying the characteristic protein domains of *sv/PAX2*, *dac/DACH1*, and *inv/EN2* in the respective homologs. To infer phylogenetic relationships between identified homologs, the NGPhylogeny.fr server (https://ngphylogeny.fr) was used (*15*). For identified homologs, smart model selection for maximum likelihood phylogenies was applied, and phylogenetic trees were established. All identified gene homologs are listed in table S2.

### Xenacoelomorpha genome sequences

At the time this study was conducted, the genome of *H. miamia* was fully sequenced (*25*) and available for navigation at Ensembl Metazoa, and the genome of *S. roscoffensis* (*29*) was available at the Acoela genome browser, https://gb.macgenome.org, for analysis of gene homologs and their exon intron structure. All other examined xenacoelomorphan genomes were only available as datasets in the Sequence Read Archive (SRA) at NCBI (https://trace.ncbi.nlm.nih.gov/Traces/sra/sra.cgi), which are listed in table S1. To determine the presence or absence of gene homologs of *sv/PAX2*, *dac/DACH1*, and *inv/EN2* in the genomes examined, the nucleotide sequences of *D. melanogaster shaven*, *dachshund*, and *engrailed*/*invected* were used to carry out BLAST searches (https://blast.ncbi.nlm.nih.gov/Blast.cgi) against the respective SRA datasets or against the genome sequence of *H. miamia* (assembly GCA_004352715.1; table S2) and the genome sequence of *S. roscoffensis* (assembly SymRos_1_5; table S2). In the case where more than one hit was identified, the results were manually assembled in the order of their percentage sequence match (highest top) with the input query using the NCBI Multiple Sequence Alignment Viewer (www.ncbi.nlm.nih.gov/projects/msaviewer/). Sequence homologies across key domains were considered as presence of the respective gene homolog in the species examined. However, because of the incomplete state of the genome sequence assemblies, the sequence homologies could not be further scrutinized to include intronic and intergenic regions but was compared to the supplementary material available in (*28*), except for the *H. miamia* (*25*) and *S. roscoffensis* (*29*) genomes.

### Local alignments and identification of DTB/MHB-related CRE-like sequences

To search for potential CRE-like sequences in gene homologs of *sv/PAX2* and *dac/DACH1*, their genomic sequences were manually extracted as text files from Ensembl, Ensembl Metazoa, or NCBI. In the case of *inv/EN2* homologs, 5-kb flanking sequences were manually extracted as text files from Ensembl, Ensembl Metazoa, or NCBI. The extracted genomic sequences were then compared against the conserved consensus sequences identified for the *Drosophila*, mouse, and human DTB/MHB-specific *sv/PAX2*, *dac/DACH1*, and *inv/EN2* CREs (fig. S1C). Local alignments were inferred using the EMBOSS Matcher algorithm that uses the eDNAfull matrix for DNA input sequences (www.ebi.ac.uk/Tools/psa/emboss_matcher/). Sequence alignments that revealed a minimum of 60% sequence identity over at least 55 bp with minimum 1 × 10^−1^ confidence level as the BLAST *e*-value were considered significant. To determine whether these CRE-like sequences are not un-annotated protein sequences and not repetitive elements, their genomic location was verified by a nucleotide BLAST search using Ensembl or Ensembl Metazoa. For those sequences that matched all selection criteria, their location was captured by screenshot and compared to the genomic location of the *Drosophila*, mouse, and human DTB/MHB-specific *sv/PAX2*, *dac/DACH1*, and *inv/EN2* CREs (*8*). All data are listed in datasets S1 to S5.

### Generation of random sequences

To generate random sequences of the same length as the CREs conserved in *Drosophila*, mouse, and human (fig. S1C), code was written for Python environment (www.python.org/downloads/). A set of 50 random sequences was generated for each of the three CREs and their specific length: for *sv/PAX2*, random sequences p1 to p50 of 356 nucleotide length; for *dac/DACH1*, random sequences d1 to d50 of 247 nucleotide length; for *inv/EN2*, random sequences e1 to e50 of 373 nucleotide length (dataset S6), each random sequence with a CG:AT ratio of 40:60 to mimic the average genomic distribution of nucleotides. The following code generates these random sequences and saves them into a .txt file (“sequences.txt”):

*import random*

*with open('sequences.txt', 'w') as f:*

*for length in [365, 247, 373]:*

*for i in range(50):*

*f.write("".join(random.choices(["G", "C", "A", "T"], weights=(0.2, 0.2, 0.4, 0.4), k=length)))*

*f.write("\n")*

### Homology index

The HI of a given sequence is defined as a function of sequence length and percentage sequence identity related to the respective DTB/MHB-specific CRE sequence of *sv/PAX2*, *dac/DACH1*, and *inv/EN2*, which was calculated as

HI=(sequence-length/length-of-conserved-CRE×%-sequence-identity)/100

The HI was determined for the identified CRE-like sequences and those random sequences that returned EMBOSS matcher results above the threshold criteria of minimum 60% sequence identity over at least 55 bp with minimum 1 × 10^−1^ confidence level (data file S1). For the identified CRE-like sequences, an average HI was determined in relation to the respective DTB/MHB-specific CRE in *Drosophila*, mouse, and human (data file S2) and compared to the HI of each of the random sequences. All resulting HIs were measured against the minimal HI to be considered significant, which, in the case of *sv/PAX2*, is HI_min_ = 0.092 (as calculated 55/356 × 60%/100), is HI_min_ = 0.133 (as calculated 55/247 × 60%/100) for *dac/DACH1*, and is HI_min_ = 0.088 (as calculated 55/373 × 60%/100) for *inv/EN2*.

### Transcription factor binding sites

To search for putative TFBSs, we focused on those initially identified within the regulatory sequences of the *H. sapiens*, *M. musculus*, and *D. melanogaster* CREs of *sv/PAX2*, *dac/DACH1*, and *inv/EN2* (*8*). In addition, we applied the CiiiDER toolkit for the prediction and study of TFBS (*44*). For scan analyses, we used the JASPAR2020_CORE_vertebrates matrix, which recovered additional, specific sites for both the DTB/MHB-specific CREs of *Drosophila*, mouse, and human and the CRE-like sequences identified in this study. Using Ensembl Protein Blast and Kalign multiple sequence alignments, we confirmed that homologs of the respective human genes are contained in the genomes investigated, supporting the presence of corresponding TFBS in the CRE-like sequences. All identified putative TFBS are listed in table S3 and data file S3.

### ATAC-seq data analysis

To investigate whether the identified CRE-like sequences are functionally active, we analyzed ATAC-seq data of the larval stages of *C. intestinalis* available at the Aniseed browser (https://anis-server.aniseed.cnrs.fr/browser/) (*64*) and the Ghost Database (http://ghost.zool.kyoto-u.ac.jp/default_ht.html). To unambiguously locate the position and extent of the identified CRE-like sequences, we used the BLAST algorithm for alignment of the respective CRE-like sequences. For our analysis, we focused on the annotation for *C. intestinalis* Roscoff type B and 10 hpf larva-stage data when brain formation is morphologically detectable.

To investigate *C. elegans* ATAC-seq data, we made use of a regulatory atlas generated and curated by the Ahringer Lab in Cambridge, UK (*65*). For our analysis, we focused on the neuron-specific ATAC-seq data registered with version 0.5.4. (https://ahringerlab.com/RegAtlas/).

### Brain-specific gene expression patterns

#### C. intestinalis

Reconstruction of cell type– and cluster-specific gene expression patterns was established using single-cell transcriptome data available for clusters of 41 neural subtypes, all of which are specific to the brain and nerve cord of the tadpole larva (*46*). The clusters were matched to the nervous system anatomy with reference to the description by Ryan *et al.* (*50*), and gene expression patterns were subsequently assigned to anatomy-specific nerve cell clusters. This was complemented with previously published gene expression patterns for homologs of *Otx*, *Pax2/5/8*, and *Hox1* (*33*, *45*, *46*, *51*), as well as for *FGF8*, *Wnt1*, and *Engrailed* (*45*, *46*, *51*).

#### C. teleta and P. dumerilii

For *C. teleta*, previously published gene expression patterns for homologs of *Otx*, *Hox1*, *Wnt*, and *Engrailed* (*37*), as well as for *FGF8* (*53*), were mapped onto the larval brain and nervous system anatomy elucidated by Seaver and colleagues (*36*, *47*). For *P. dumerilii*, reconstruction of brain area and cell type–specific gene expression patterns was established using the three-dimensional (3D) single-cell gene expression atlas via the 3D whole-body electron microscopy viewer made available as a Fiji plugin by Vergara *et al.* (*48*). With this tool, the cell-specific gene expression of *Dachshund*, *Pax2/5/8*, *Wnt*, and *Hox1* homologs was determined and visualized for each gene in each of the head ganglia. This single-cell and cluster-specific brain atlas was complemented with previously published gene expression patterns for homologs of *Six3*, *Otx*, *Engrailed*, and *Hox1* (*54*, *55*).

#### C. elegans

Reconstruction of cell type– and circuit-specific gene expression patterns was established using single-cell transcriptome and connectome data available that identify all cells of the brain (*41*, *56*–*58*). To establish single-cell resolution of gene expression patterns, we used the CeNGEN project (https://cengen.shinyapps.io/CengenApp/) (*49*, *57*) and determined each cell in which the gene homologs are expressed. The resulting cell type–specific gene expression atlas was mapped onto the available connectome, which allowed identification of each cell and its projections contributing to the recently identified four strata (S1 to S4) and three layers of the brain neuropil (*41*, *56*–*58*).

## Supplementary Materials

### This PDF file includes:

Figs. S1 to S12 Tables S1 to S8 Datasets S1 to S6 Legends for data files S1 to S3

## Notes

View/request a protocol for this paper from *Bio-protocol*.
