# Supplementary material for: Phylogenetic tracing of midbrain-specific regulatory sequences suggests single origin of eubilaterian brains
Source: Sci Adv. 2023 May 24;9(21):eade8259. doi: 10.1126/sciadv.ade8259 (PMC10208574; doi:10.1126/sciadv.ade8259)
Supplement: Supplementary file 1 — Figs. S1 to S12 Tables S1 to S8 Datasets S1 to S6 Legends for data files S1 to S3 [file sciadv.ade8259_sm.pdf]

Science Advances
AAAS

Supplementary Materials for

Phylogenetic tracing of midbrain-specific regulatory sequences suggests single origin of eubilaterian brains

Helen C. Schuster and Frank Hirth

Corresponding author: Frank Hirth, frank.hirth@kcl.ac.uk

Sci. Adv. **9**, eade8259 (2023)
DOI: 10.1126/sciadv.ade8259

The PDF file includes:

Figs. S1 to S12
Tables S1 to S8
Datasets S1 to S6
Legends for data files S1 to S3

Other Supplementary Material for this manuscript includes the following:

Data files S1 to S3

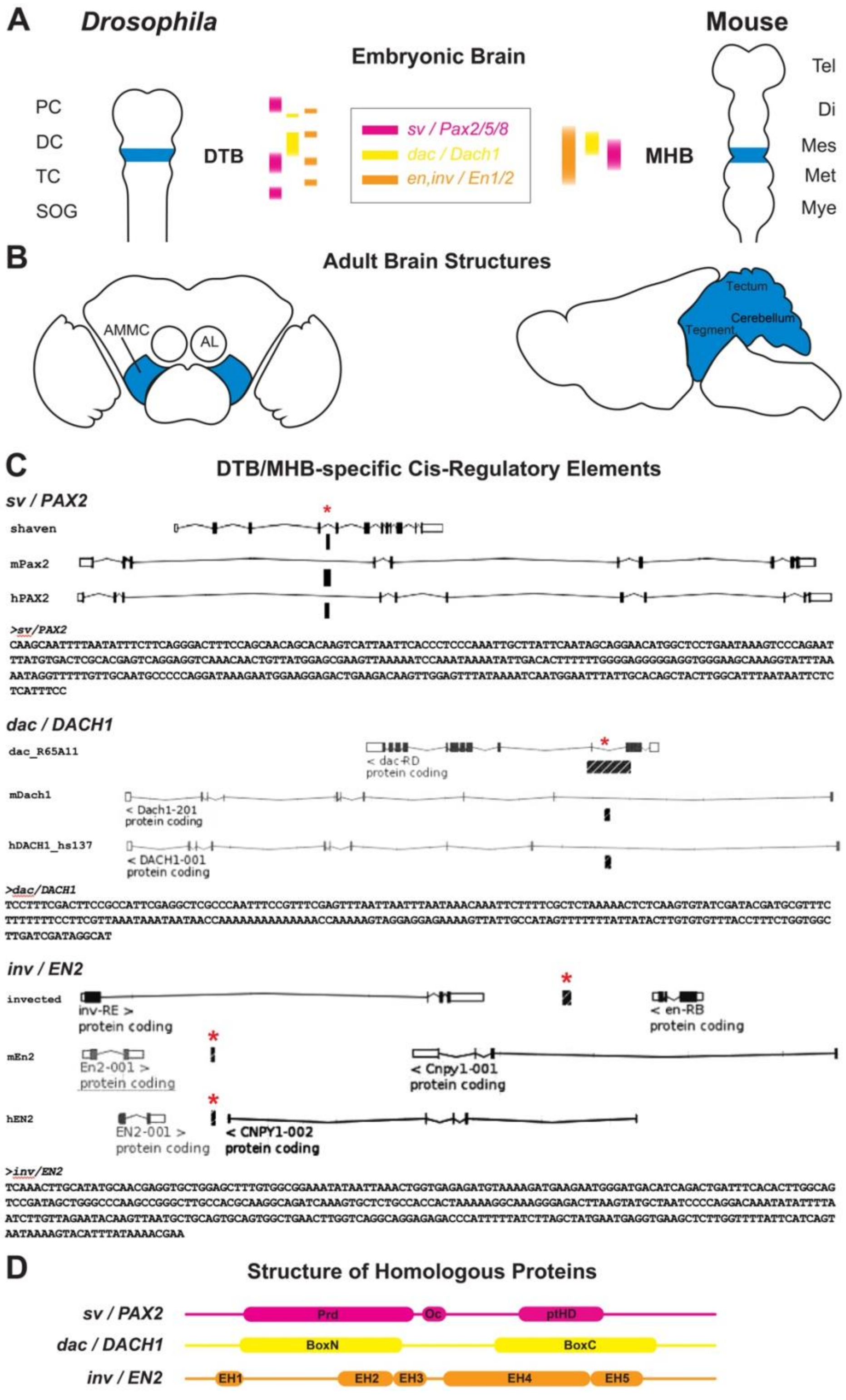

Fig. S1.

Conserved cis-regulatory elements regulate midbrain circuit formation in *Drosophila* and mouse. (**A**) Schematic of embryonic *Drosophila* and mouse brain showing expression patterns of *shaven/Pax2/5/8 (sv/PAX2)*, *dachshund/DACH1 (dac/DACH1)* and *engrailed*,

invected/ENGRAILED-1/2 (en, inv/EN1/2) gene homologs along the deutocerebral-tritocerebral boundary (DTB) and the midbrain hindbrain boundary (MHB). (**B**) Schematic of adult *Drosophila* and mouse brain; highlighted in blue are structures derived from, respectively, DTB and MHB regions. (**C**) Genomic location (asterisks) and consensus sequences of conserved DTB/MHB-related cis-regulatory elements of *sv/PAX2*, *dac/DACH1* and *inv/EN2*. (**D**) Protein domains of sv/PAX2, dac/DACH1 and inv/EN2 used for homology search. *Abbreviations*: AL, antennal lobe; AMMC, antennal mechanosensory motor center; dc, deutocerebrum; di, diencephalon; DTB, deutocerebral-tritocerebral boundary; EH, engrailed homology motif; hb, hindbrain; mb, midbrain; Mes, Mesencephalon; Met, Metencephalon; Mye, Myelencephalon; MHB, midbrain hindbrain boundary; Oc, octapeptide; pc, protocerebrum; prd, paired domain; ptHD, paired-type homeodomain; SOG, Subesophageal ganglion; tc, tritocerebrum; tel, telencephalon. Modified after ref. 8.

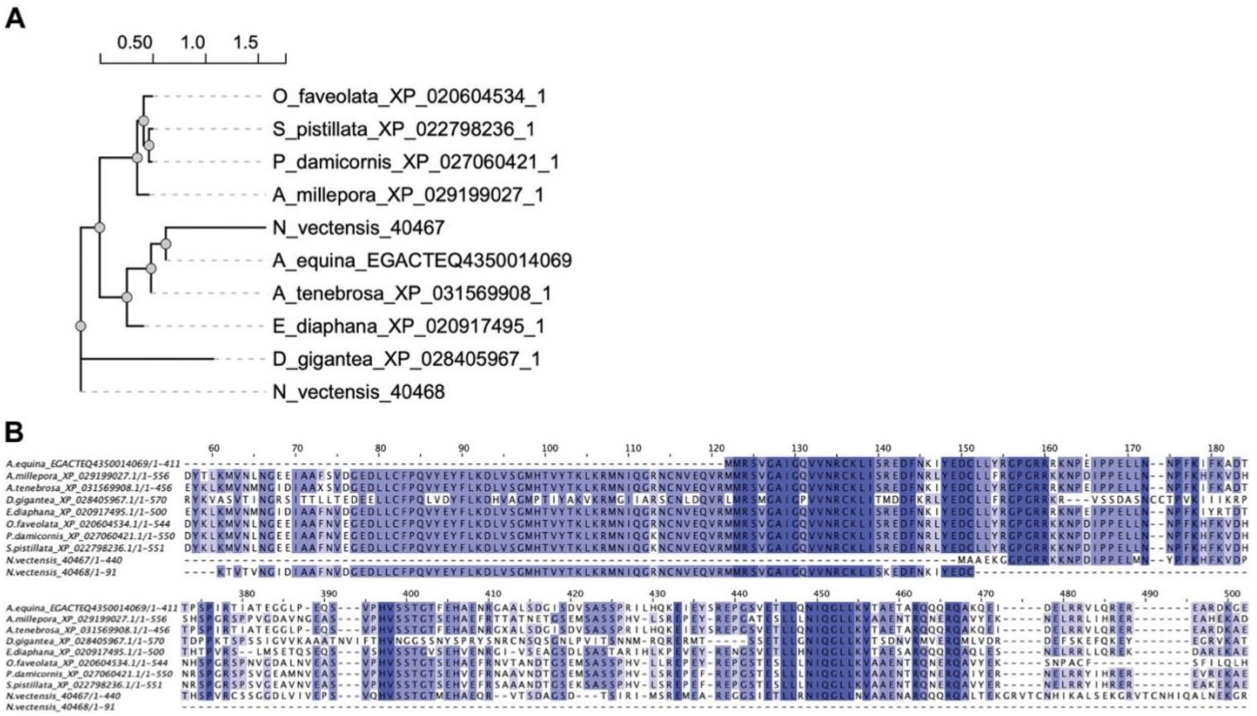

Fig. S2.

***dachshund*-like homologs in non-bilaterian genomes.** (**A**) Phylogenetic tree of *dachshund/DACH1* protein homologs detected in the sequenced genomes of the cnidarians *Orbicella faveolata* (XP_020604534.1); *Stylophora pistillata* (XP_022798236.1); *Pocillopora damicornis* (XP_027060421.1); *Acropora millepora* (XP_029199027.1); *Nematostella vectensis* (EDO40467; EDO40468); *Actinia equina* (EGACTEQ4350014069-PA); *Actinia tenebrosa* (XP_031569908.1); *Exaiptasia diaphana* (XP_020917495.1); *Dendronephthya gigantea* (XP_028405967.1). Tree inferred using the NGPhylogeny.fr server (https://ngphylogeny.fr) by applying smart model selection for maximum likelihood phylogenies. (**B**) Partial sequence comparison of named proteins using the Kalign neighbour joining algorithm available at the European Bioinformatics Institute (www.ebi.ac.uk./Tools/msa/kalign/); color code, percentage identity.

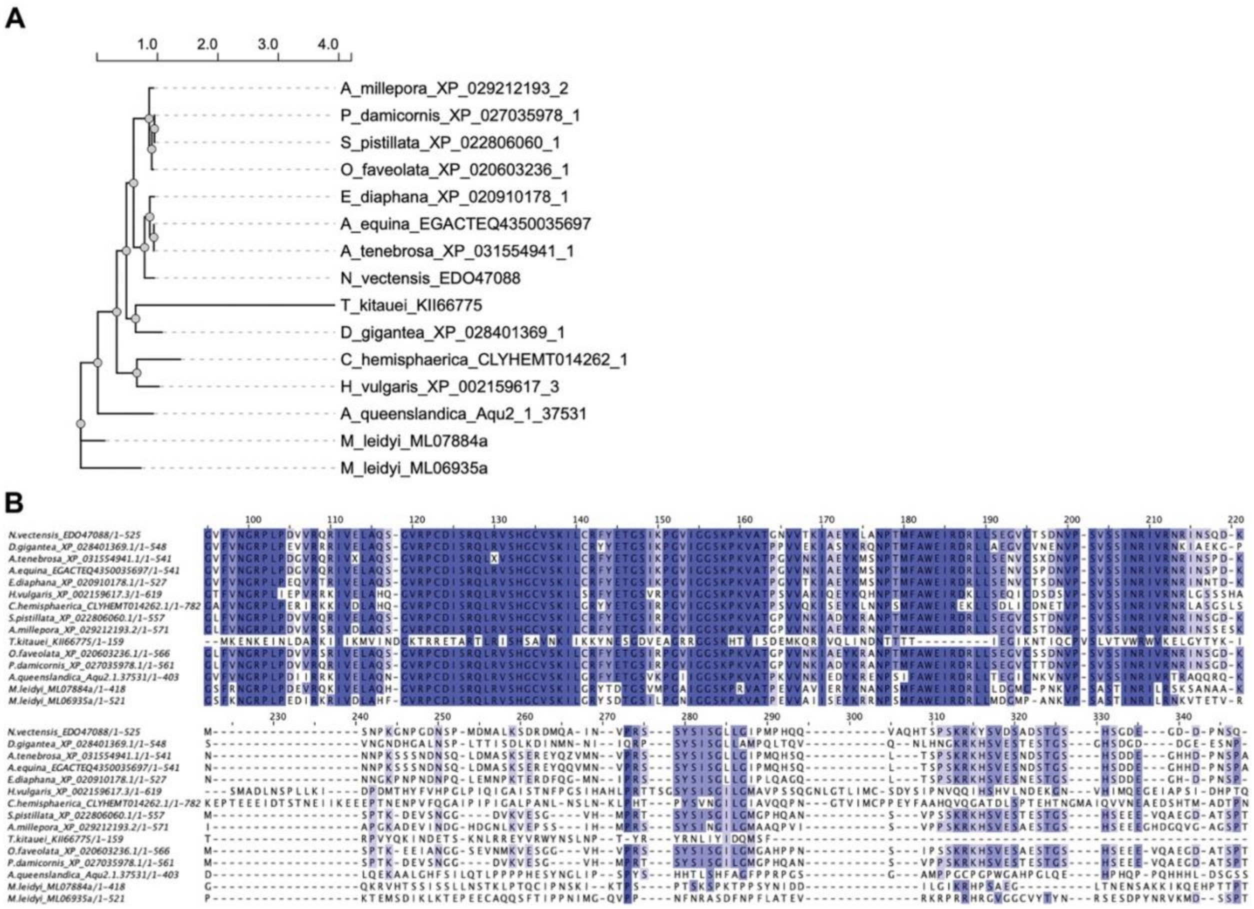

Fig. S3.

***Pax2/5/8*-like homologs in non-bilaterian genomes.** (**A**) Phylogenetic tree of *Pax2/5/8* protein homologs detected in the sequenced genomes of the cnidarians *Acropora millepora* (XP_029212193.2); *Pocillopora damicornis* (XP_027035978.1); *Stylophora pistillata* (XP_022806060.1); *Orbicella faveolata* (XP_020603236.1); *Exaiptasia diaphana* (XP_020910178.1); *Actinia equina* (EGACTEQ4350035697-PA); *Actinia tenebrosa* (XP_031554941.1); *Nematostella vectensis* (EDO47088); *Thelohanellus kitauei* (KII66775); *Dendronephthya gigantea* (XP_028401369.1); *Clytia hemisphaerica* (CLYHEMP014262.1); *Hydra vulgaris* (XP_002159617.3); *Amphimedon queenslandica* (Aqu2.1.37531_001); *Mnemiopsis leidyi* (ML07884a-PA; ML06935a-PA). Tree inferred using the NGPhylogeny.fr server (https://ngphylogeny.fr) by applying smart model selection for maximum likelihood phylogenies. (**B**) Partial sequence comparison of named proteins using the Kalign neighbour joining algorithm available at the European Bioinformatics Institute (www.ebi.ac.uk./Tools/msa/kalign/); color code, percentage identity.

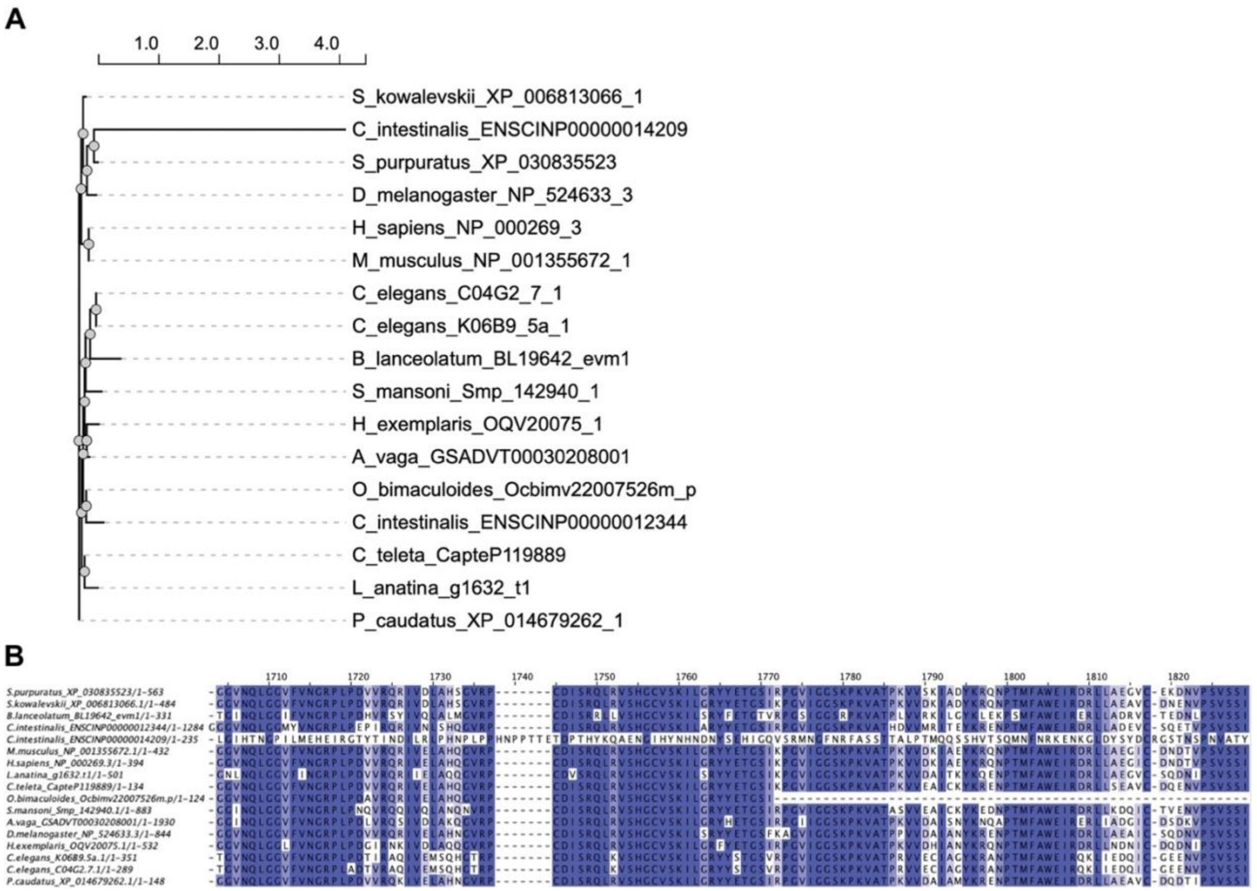

Fig. S4.

***shaven/PAX2* homologs in eubilaterian genomes.** (**A**) Phylogenetic tree of *shaven/PAX2 (sv/PAX2)* homologs detected in the sequenced genomes of *Saccoglossus kowalevskii* (XP_006813066.1); *Ciona intestinalis* (ENSCINP00000012344; ENSCINP00000014209); *Strongylocentrotus purpuratus* (XP_030835523); *Drosophila melanogaster* (NP_524633.3); *Homo sapiens* (NP_000269.3); *Mus musculus* (NP_001355672.1); *Caenorhabditis elegans* (K06B9.5a.1; C04G2.7.1); *Branchiostoma lanceolatum* (BL19642_evm1); *Schistosoma mansoni* (Smp_142940.1:pep); *Hypsibius exemplaris* (OQV20075.1); *Adineta vaga* (GSADVT00030208001); *Octopus bimaculoides* (Ocbimv22007526m.p); *Capitella teleta* (CapteP119889); *Lingula anatina* (g1632.t1); *Priapulus caudatus* (XP_014679262.1). Tree inferred using the NGPhylogeny.fr server (https://ngphylogeny.fr) by applying smart model selection for maximum likelihood phylogenies. (**B**) Partial sequence comparison of named proteins using the Kalign neighbour joining algorithm available at the European Bioinformatics Institute (www.ebi.ac.uk./Tools/msa/kalign/); color code, percentage identity.

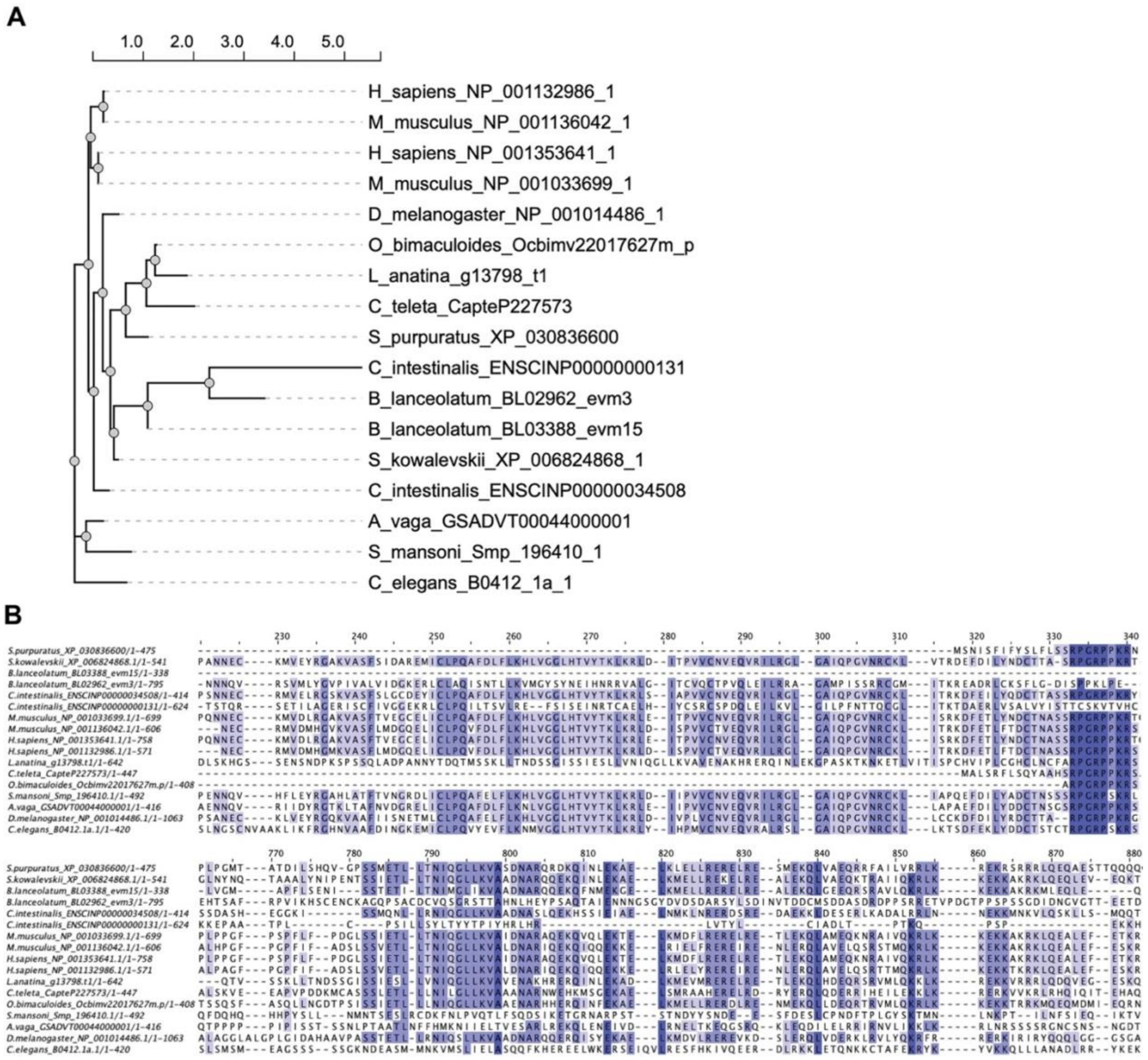

Fig. S5.

***dachshund/DACH1* homologs in eubilaterian genomes.** (**A**) Phylogenetic tree of *dachshund/DACH1 (dac/DACH1)* homologs detected in the sequenced genomes of *Homo sapiens* (NP_001353641.1; NP_001132986.1); *Mus musculus* (NP_001033699.1; NP_001136042.1); *Drosophila melanogaster* (NP_001014486.1); *Octopus bimaculoides* (Ocbimv22017627m.p); *Lingula anatina* (g13798.t1); *Capitella teleta* (CapteP227573); *Strongylocentrotus purpuratus* (XP_030836600); *Ciona intestinalis* (ENSCINP00000034508, ENSCINP00000000131); *Branchiostoma lanceolatum* (BL03388_evm15; BL02962_evm3); *Saccoglossus kowalevskii* (XP_006824868.1); *Schistosoma mansoni* (Smp_196410.1); *Adineta vaga* (GSADVT00044000001); *Caenorhabditis elegans* (B0412.1a.1). Tree inferred using the NGPhylogeny.fr server (https://ngphylogeny.fr) by applying smart model selection for maximum likelihood phylogenies. (**B**) Partial sequence comparison of named proteins using the Kalign neighbour joining algorithm available at the European Bioinformatics Institute (www.ebi.ac.uk./Tools/msa/kalign/); color code, percentage identity.

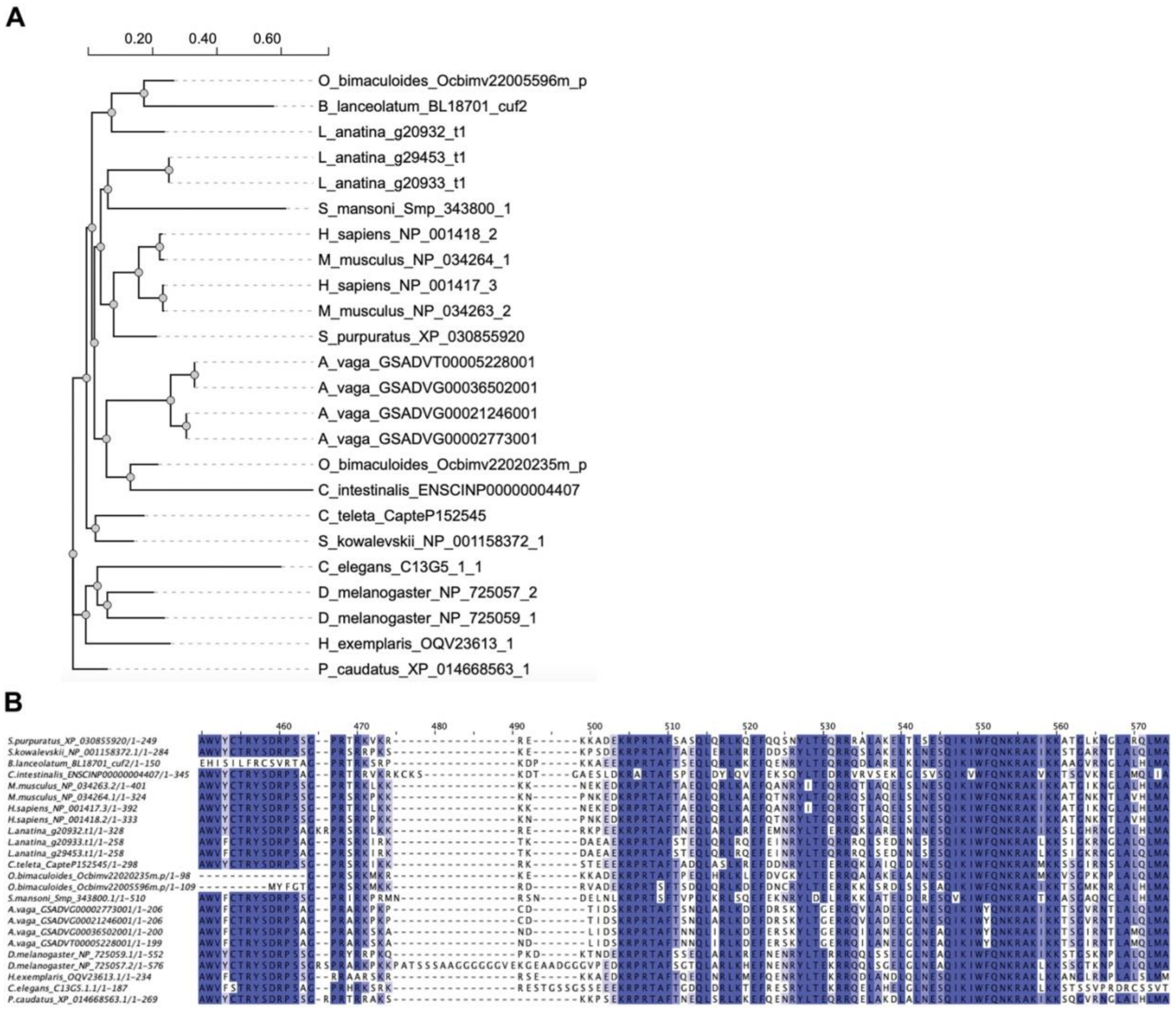

Fig. S6.

***invected/ENGRAILED2* homologs in eubilaterian genomes.** (**A**) Phylogenetic tree of *invected/ENGRAILED2 (inv/EN2)* homologs detected in the sequenced genomes of *Octopus bimaculoides* (Ocbimv22020235m.p; Ocbimv22005596m.p); *Branchiostoma lanceolatum* (BL18701_cuf2); *Lingula anatina* (g20932.t1; g20933.t1; g29453.t1); *Schistosoma mansoni* (Smp_343800.1); *Homo sapiens* (NP_001417.3; NP_001418.2); *Mus musculus* (NP_034263.2; NP_034264.1); *Strongylocentrotus purpuratus* (XP_030855920); *Adineta vaga* (GSADVT00002773001; GSADVT00021246001; GSADVT00005228001; GSADVT00036502001); *Ciona intestinalis* (ENSCINP00000004407); *Capitella teleta* (CapteP152545); *Saccoglossus kowalevskii* (NP_001158372.1); *Caenorhabditis elegans* (C13G5.1.1); *Hypsibius exemplaris* (OQV23613.1); *Drosophila melanogaster* (NP_725059.1; NP_725057.2); *Priapulus caudatus* (XP_014668563.1). Tree inferred using the NGPhylogeny.fr server (https://ngphylogeny.fr) by applying smart model selection for maximum likelihood phylogenies. (**B**) Partial sequence comparison of named proteins using the Kalign neighbour joining algorithm available at the European Bioinformatics Institute (www.ebi.ac.uk./Tools/msa/kalign/); color code, percentage identity.

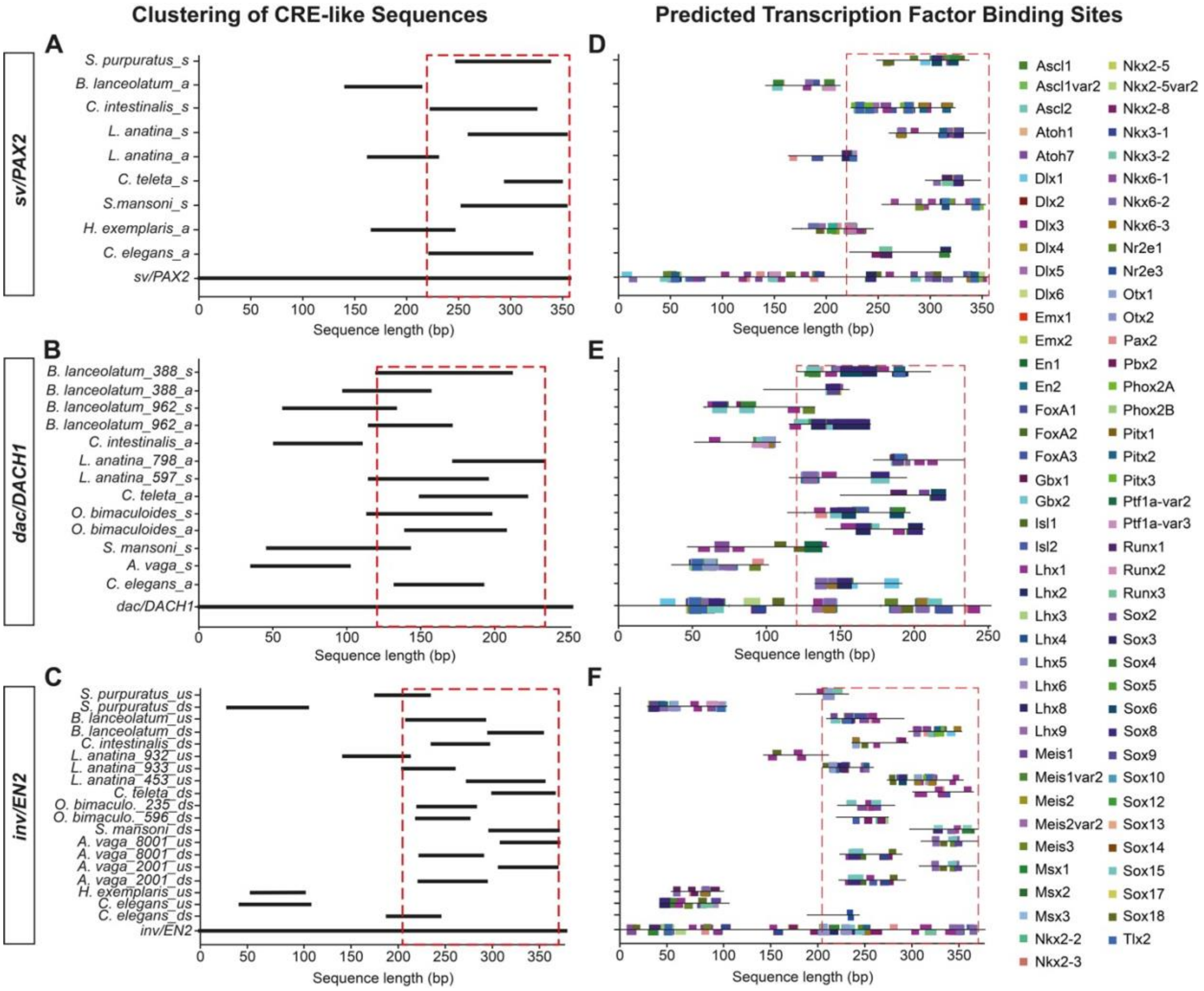

Fig. S7.

Clustering of DTB/MHB-related CRE-like sequences and their predicted transcription factor binding sites. Most cis-regulatory element (CRE)-like sequences identified for the shown species share a common domain outlined as red dashed box for (**A**) *shaven/PAX2* (*sv/PAX2*); (**B**) *dachshund/DACH1* (*dac/DACH1*), and (**C**) *invected/Engrailed-2* (*inv/EN2*). (**D-F**) Prediction and analysis of transcription factor binding sites (TFBS) as revealed by the CiiiDER algorithm (44). Note the enrichment of transcription factors known to be involved in the formation and function of the arthropod deutocerebral-tritocerebral boundary (DTB) and vertebrate midbrain hindbrain boundary (MHB) region (8).

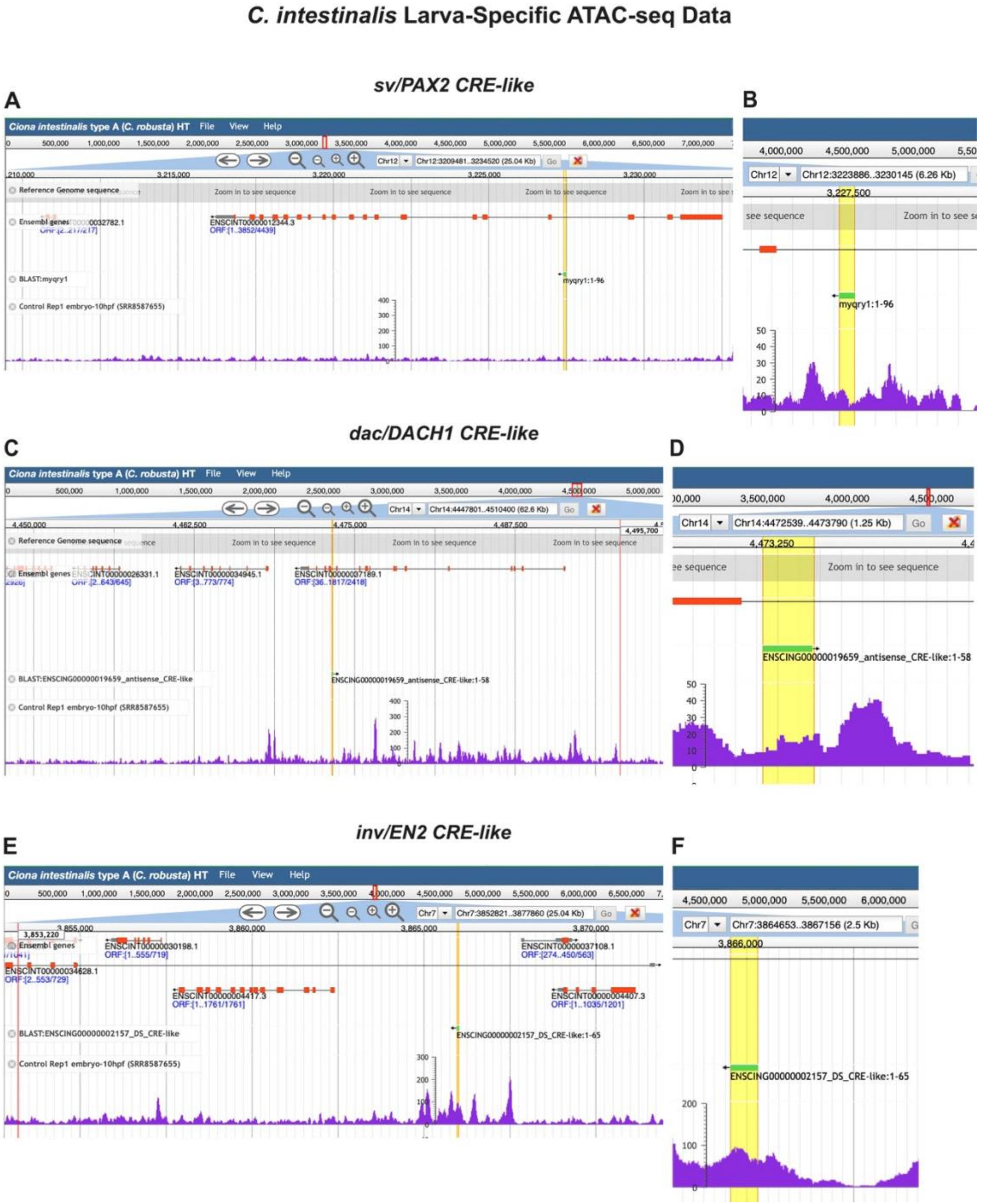

Fig. S8.

***Ciona intestinalis* ATAC-seq data.** ATAC-seq of 10hpf larvae reveals chromatin accessibility of genomic regions of (**A**) *shaven/PAX2*, (**C**) *dachshund/DACH1*, and (**E**) *invected/ENGRAILED2*, and respective CRE-like sequences highlighted as yellow bands in zoomed-in views (**B, D, F**). Note the inverted orientation of the *dac/DACH1* and *inv/EN2* genes compared to the ENSEMBL genome annotation. ATAC-seq data derived from the Ghost Database (http://ghost.zool.kyoto-u.ac.jp/default_ht.html) of the *Ciona intestinalis* type A (robusta) genome (64).

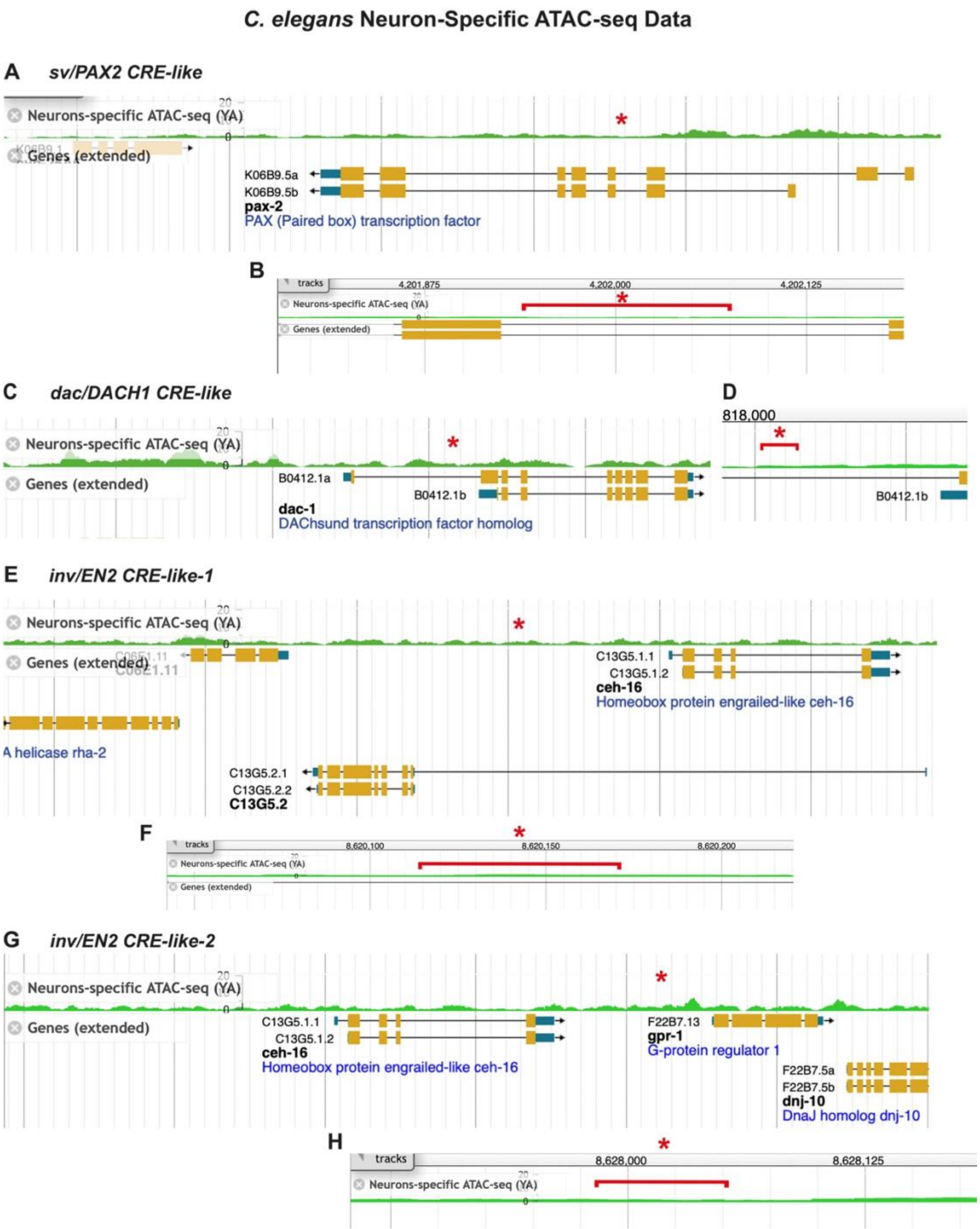

Fig. S9.

***Caenorhabditis elegans* neuron-specific ATAC-seq data.** ATAC-seq reveals chromatin accessibility of genomic regions and respective CRE-like sequences highlighted with red asterisks and brackets in zoomed-in views of (**A, B**) *shaven/PAX2*, (**C, D**) *dachshund/DACH1*, and (**E-H**) *invected/ENGRAILED2*. Note residual activity related to limited number of neurons expressing *sv/Pax2*, *dac/Dach1* and *inv/EN2* homologs (compare with Table S8). ATAC-seq data derived from the *C. elegans* regulatory atlas v0.5.4. (https://ahringerlab.com/RegAtlas/) (65).

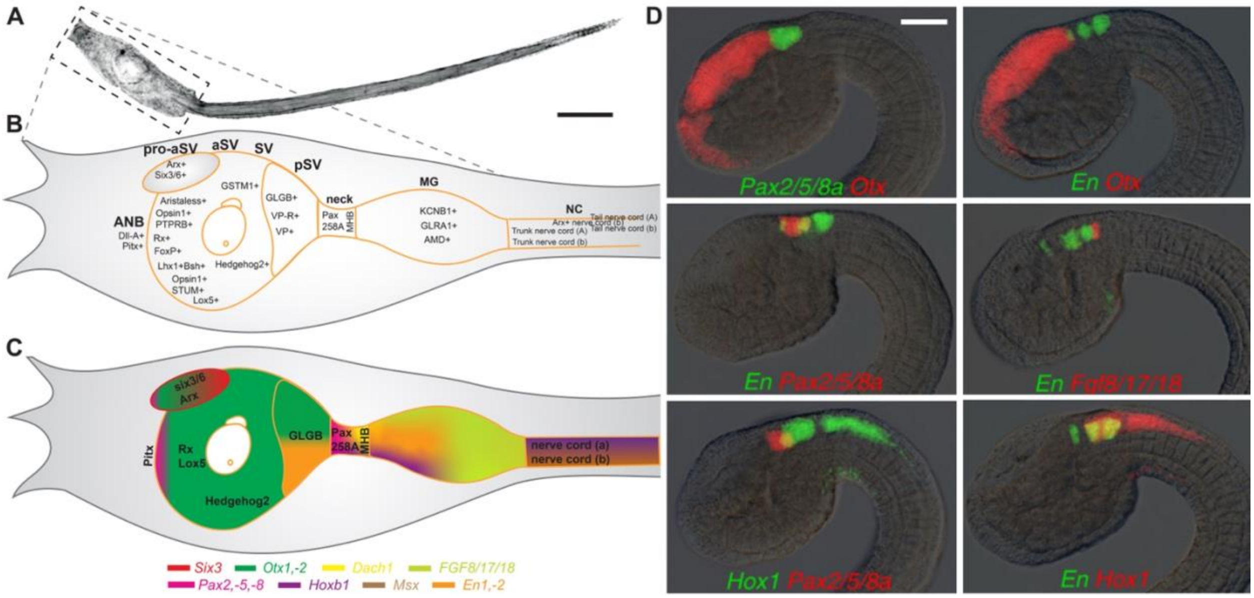

Fig. S10.

Cell type and cluster-specific gene expression in the brain of the tadpole larva of *Ciona intestinalis*. (**A**) *C. intestinalis* tadpole larva, anterior to the left. (**B**) Cell clusters in the central nervous system of the *C. intestinalis* tadpole larva are subdivided into anterior brain (ANB) consisting of pro-anterior (pro-aSV), anterior (aSV) and posterior sensory vesicles (pSV); neck region (neck) comprising midbrain hindbrain boundary (MHB); adjacent motor ganglion (MG), and the trunk and tail nerve cord (NC). (**C**) Cell cluster-specific gene expression in the different regions of the *C. intestinalis* larval CNS. (**D**) Brain-specific expression pattern of *Otx*, *Pax2/5/8a*, *Engrailed* (*En*), *Fgf8/17/18*, and nerve-cord specific expression of *Hox1* at mid tailbud stage; data from http://www.aniseed.cnrs.fr/aniseed/ originated from ref. 51. Sources: refs. 33, 45, 45, 50, 51. Scale bars: 100µm in A; 50µm in D.

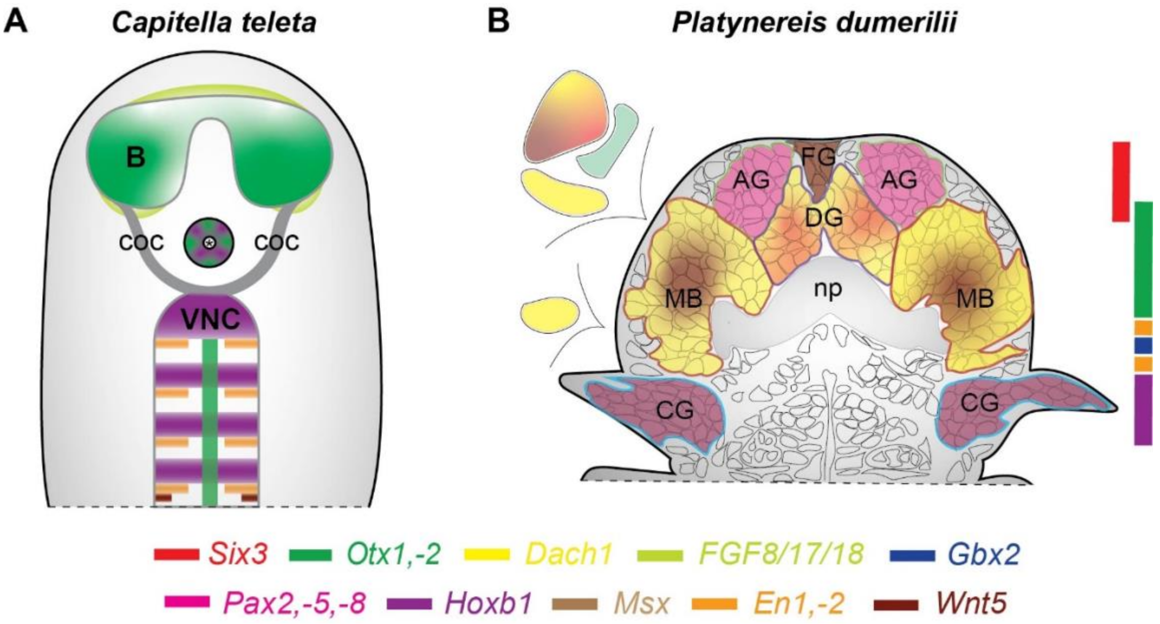

Fig. S11.

Region and cell cluster-specific gene expression in the larval brain of *Capitella teleta* and *Platynereis dumerilii.* (**A**) Expression domains of *Otx*, *FGF8*, *Engrailed* and *Hoxb1* genes subdivide the central nervous system of *Capitella teleta* into brain (B) and ventral nerve chord (VNC), both of which are separated by circumoral connectives (COC) surrounding the stomodeum (asterisk). (**B**) Ganglion and cell cluster-specific gene expression pattern in *Platynereis dumerilii* larva. *Abbreviations*: FG, frontal ganglion; AG, antennal ganglion; DG, dorsal ganglion; MB, mushroom bodies; CG, cirral ganglion; np, neuropil. Sources: refs. 47, 48, 53-55.

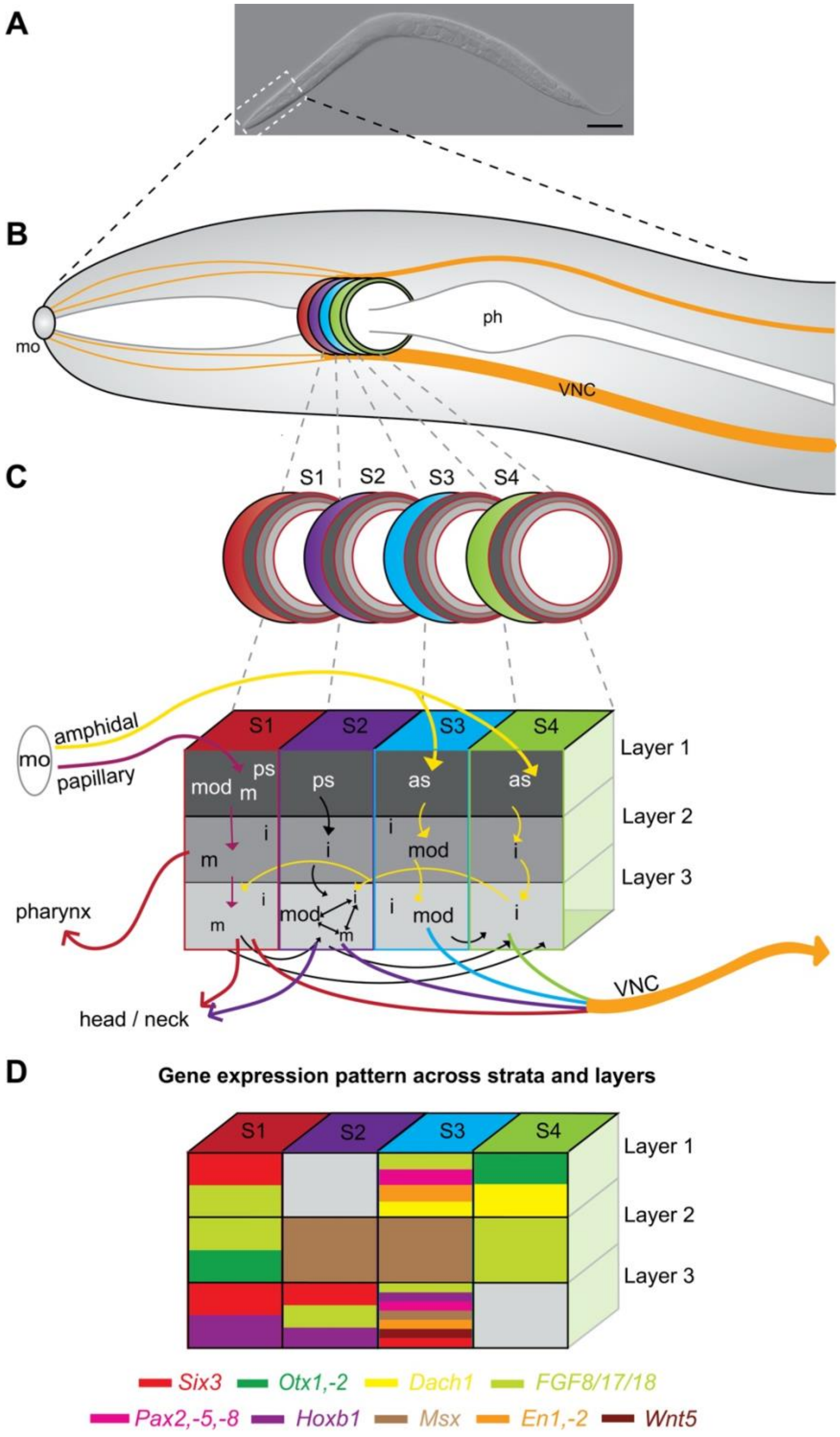

Fig. S12.

Region and cell cluster-specific gene expression in the larval brain of *Capitella teleta* and Strata and layer-specific gene expression in the brain of *C. elegans*. (A) *Caenorhabditis elegans*, anterior to the left. (**B**) Schematic of head region and brain, the neuropil of which surrounds pharynx (ph) and receives sensory input from mouth (mo) region; dorsal and ventral

nerve cord (VNC) indicated in orange. Four functionally distinct strata (S1-S4) subdivide the brain neuropil, each stratum comprising three distinct layers (L1-L3). (**C**) Strata S1 and S2 receive papillary sensory input, strata S3 and S4 receive amphidal sensory input from the mouth; motor neurons are situated in layers 2 and 3 of strata S1 and S2. (**D**) Strata and layer-specific gene expression patterns. Color code: Red, stratum 1; purple, stratum 2; blue, stratum 3; green, stratum 4. *Abbreviations*: mod, modulatory neuron; m, motor input; as, amphidal sensory input; ps, papillary sensory input; i, interneuron. Sources: refs. 41, 56-58 and https://cengen.shinyapps.io/CengenApp/. Scale bar in A, 100μm.

Table S1. Eumetazoan species, their nervous system, and sequenced genome examined.

Taxon	Nervous system	Description of Nervous System	Species & Genome Assembly
**Ctenophora**	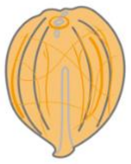	Diffuse nerve net with regional condensations, apical organ and juxtatentacular nerve cords.	***Mnemiopsis leidyi***<br>MneLei_Aug2011
**Porifera**	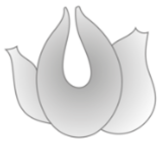	No nervous system, but cell-cell-interactions.	***Amphimedon queenslandica***<br>Aqu1
**Cnidaria**	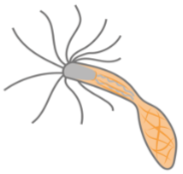	Diffuse nerve net with regional condensations. As larvae: anterior concentration of sensory cells into a ganglion.	***Clytia hemisphaerica*** (Z4C2)<br>GCA902728285v1<br>***Hydra vulgaris***<br>GCA000004095v1<br>***Thelohanellus kitauei***<br>ASM82789v1
**Xenacoelomorpha**	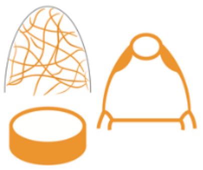	Xenoturbella: basiepidermal nerve net. Nemertodermatida: with neurite bundles and sometimes brain condensation. Acoela: With subepidermal brain or nerve ring.	***Hofstenia miamia***<br>HmiaM1<br>***Symsagittifera roscoffensis***<br>SymRos_1_5<br>***Isodiametra pulcha*** (SRA)<br>SRR12179258,SRR5760180<br>***Xenoturbella bocki*** (SRA)<br>SRR5760181,SRR8524532, SRR8524534
**Hemichordata**	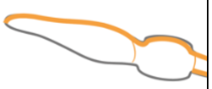	Nerve net with neurite bundles forming a dorsal nerve cord from proboscis to trunk and a ventral nerve cord in the trunk.	***Saccoglossus kowalevskii*** (NCBI)<br>GCA_000003605.1
**Echinodermata**	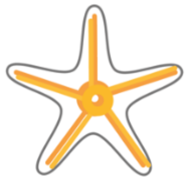	Circumoral nerve ring and five nerve cords, one for each radius, on ectoneural and hyponeural level. As larvae: Apical organ with central ganglion in two cell clusters.	***Strongylocentrotus purpuratus*** *(Spur 01)*<br>Spur_5.0
**Urochordata**	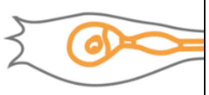	Larvae: anterior and posterior sensory vesicle, neck, motor ganglion, dorsal nerve cord.	***Ciona intestinalis***<br>KH
**Cephalochordata**	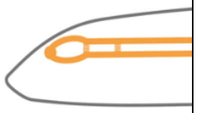	Cerebral vesicle and dorsal nerve cord.	***Branchiostoma lanceolatum***<br>BraLan2
**Craniata**	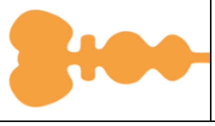	Dorsal nerve cord and fore-, mid- and hindbrain differentiating into tele-, di-, mes-, met- and myelencephalon.	***Homo sapiens***<br>GRCh38.p13<br>***Mus musculus***<br>GRCm39

**Brachiopoda**	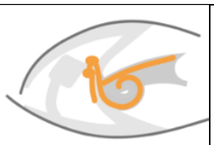	Supra- and subenteric ganglion with nerves emanating to innervate mantle and lophophore.	***Lingula anatina*** LinAna1.0
**Annelida**	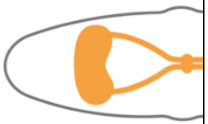	Dorsal brain, circumoral connectives, paired ventral nerve cord with segmental ganglia.	***Capitella teleta*** Capitella_teleta_v1.0
**Mollusca**	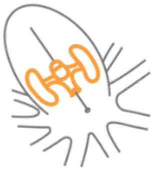	Circumferential brain, divided in three masses and numerous fused lobes. Nerve cords emanate to innervate each arm.	***Octopus bimaculoides*** PRJNA270931
**Platyhelminthes**	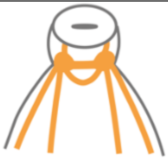	Paired ganglion with commissures and several paired nerve cords of which the ventral pair is most prominent.	***Schistosoma mansoni*** *(PRJEA36577)* Smansoni_v7
**Rotifera**	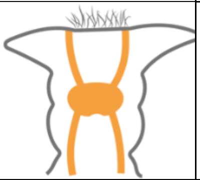	Paired ganglion with two main nerve cords fusing caudally.	***Adineta vaga*** AMS_PRJEB1171_v1
**Tardigrada**	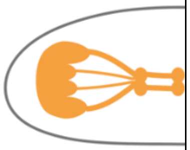	Four lobed ganglia connected to the paired nerve cord with paired segmental ganglia via outer and inner connectives.	***Hypsibius exemplaris*** nHd_3.1
**Arthropoda**	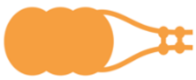	Anterior dorsal brain consisting generally of proto-, deuto- and tritocerebrum; paired ventral nerve cord with segmental ganglia.	***Drosophila melanogaster*** BDGP6.32
**Nematoda**	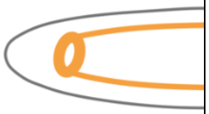	Circumesophageal nerve ring consisting of four functionally distinct strata and three layers; ventral and less prominent dorsal nerve cord.	***Caenorhabditis elegans*** WBcel235
**Priapulida**	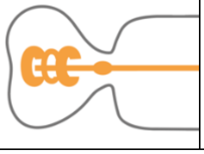	Circumpharyngeal nerve ring consisting of neuropil and two somata aggregations, single ventral nerve cord with neck and caudal ganglion.	***Priapulus caudatus*** (NCBI) GCA_000485595.2

Abbreviations: INSDC, *International Nucleotide Sequence Database Collaboration* where the genome sequences are publicly available.

Table S2. Genomes examined for homologs of *shaven/PAX2*, *dachshund/DACH1* and *invected/ENGRAILED-2*.

Species	Genome Assembly	*shaven/PAX2* homologs	*dachshund/DACH1* homologs	*invected/ENGRAILED-2* homologs
M. leidyi	Ensembl-genomes GCA_000226015.1	Description: --<br>Gene: **ML07884a**<br>Transcript: ML07884a-RA<br>Product: ML07884a-PA<br>NCBI: --<br>Uniprot: --<br><br>Description: --<br>Gene: **ML06935a**<br>Transcript: ML06935a-RA<br>Product: ML06935a-PA<br>NCBI: --<br>Uniprot: --	No homolog	No homolog
A. queens-landica	Ensembl-genomes GCA_000090795.1	Description: --<br>Gene: **Aqu2.1.37531**<br>Transcript: Aqu2.1.37531_001<br>Product: Aqu2.1.37531_001<br>NCBI: --<br>Uniprot: A0A1X7VBY1	No homolog	No homolog
T. kitauei	Ensembl-genomes GCA_000827895.1	No homolog	No homolog	No homolog
H. vulgaris	Ensembl-genomes GCA_000004095.1	Description: homeobox protein 10<br>Gene: **LOC100192231**<br>Transcript: XM_002159581.3<br>Product: XP_002159617.3<br>NCBI: XP_002159617.3<br>Uniprot: A0A8B6XLR5	No homolog	No homolog
C. hemi-sphaerica	Ensembl-genomes GCA_902728285.1	Description: --<br>Gene: **CLYHEMG014262**<br>Transcript: CLYHEMT014262.1<br>Product: CLYHEMP014262.1<br>NCBI: --<br>Uniprot: A0A0P0E5Y5	No homolog	No homolog
D. gigantea	Ensembl-genomes GCA_004324835.1	Description: paired box protein Pax-5-like<br>Gene: **LOC114524477**<br>Transcript: XM_028545568.1<br>Product: XP_028401369.1<br>NCBI: XP_028401369.1<br>Uniprot: --	Description: dachshund homolog 1-like<br>Gene: **LOC114528507**<br>Transcript: XM_028550166.1<br>Product: XP_028405967.1<br>NCBI: XP_028405967.1<br>Uniprot: --	No homolog
A. equina	Ensembl-genomes GCA_011057435.1	Description: --<br>Gene: **EGACTEQ4350035697**<br>Transcript: EGACTEQ4350035697-RA<br>Product: EGACTEQ4350035697-PA<br>NCBI: --<br>Uniprot: --	Description: --<br>Gene: EGACTEQ4350014069<br>Transcript: EGACTEQ4350014069-RA<br>Product: EGACTEQ4350014069-PA<br>NCBI: --<br>Uniprot: --	No homolog
A. tenebrosa	Ensembl-genomes GCA_009602425.1	Description: paired box protein Pax-5-like<br>Gene: **LOC116291861**<br>Transcript: XM_031699081.1<br>Product: XP_031554941.1<br>NCBI: XP_031554941.1<br>Uniprot: A0A6P8HGI4	Description: dachshund homolog 2-like<br>Gene: **LOC116304329**<br>Transcript: XM_031714048.1<br>Product: XP_031569908.1<br>NCBI: XP_031569908.1<br>Uniprot: A0A6P8IUT1	No homolog

E. diaphana	Ensembl-genomes<br>GCA_001417965.1	Description: paired box protein Pax-6<br>Gene: **LOC110248033**<br>Transcript: XM_021054519.2<br>Product: XP_020910178.1<br>NCBI: XP_020910178.1<br>Uniprot: --	Description: dachshund homolog 1<br>Gene: **LOC110254792**<br>Transcript: XM_021061836.1<br>Product: XP_020917495.1<br>NCBI: XP_020917495.1<br>Uniprot: --	No homolog
N. vectensis	Ensembl-genomes<br>GCA_000209225.1	Description: PAXB<br>Gene: **NEMVEDRAFT_v1g239535**<br>Transcript: EDO47088<br>Product: EDO47088<br>NCBI: XM_001639101.1<br>XP_001639151.1<br>Uniprot: A7RN74	Description: Predicted protein<br>Gene: **NEMVEDRAFT_v1g207853**<br>Transcript: EDO40467<br>Product: EDO40467<br>NCBI: --<br>Uniprot: A7S747<br><br>Description: Predicted Protein<br>Gene: **NEMVEDRAFT_v1g107148**<br>Transcript: EDO40468<br>Product: EDO40468<br>NCBI: --<br>Uniprot: A7S748	No homolog
A. millepora	Ensembl-genomes<br>GCA_013753865.1	Description: paired box protein Pax-6<br>Gene: **LOC114976020**<br>Transcript: XM_029356360.2<br>Product: XP_029212193.2<br>NCBI: XP_029212193.2<br>Uniprot: --	Description: dachshund homolog 1-like<br>Gene: **LOC114963920**<br>Transcript: XM_029343194.2<br>Product: XP_029199027.1<br>NCBI: XP_029199027.1<br>Uniprot: --	No homolog
O. faveolata	Ensembl-genomes<br>GCA_002042975.1	Description: paired box protein Pax-6-like<br>Gene: **LOC110042216**<br>Transcript: XM_020747577.1<br>Product: XP_020603236.1<br>NCBI: XP_020603236.1<br>Uniprot: --	Description: dachshund homolog 1-like<br>Gene: **LOC110043426**<br>Transcript: XM_020748875.1<br>Product: XP_020604534.1<br>NCBI: XP_020604534.1<br>Uniprot: --	No homolog
P. damicornis	Ensembl-genomes<br>GCA_003704095.1	Description: paired box protein Pax-6-like<br>Gene: **LOC113664562**<br>Transcript: XM_027180177.1<br>Product: XP_027035978.1<br>NCBI: XP_027035978.1<br>Uniprot: A0A3M6UGD1	Description: dachshund homolog 1-like<br>Gene: **LOC113686936**<br>Transcript: XM_027204620.1<br>Product: XP_027060421.1<br>NCBI: XP_027060421.1<br>Uniprot: A0A3M6UI48	No homolog
S. pistillata	Ensembl-genomes<br>GCA_002571385.1	Description: paired box protein Pax-6-like<br>Gene: **LOC111343163**<br>Transcript: XM_022950325.1<br>Product: XP_022806060.1<br>NCBI: XP_022806060.1<br>Uniprot:	Description: dachshund homolog 1-like<br>Gene: **LOC111336406**<br>Transcript: XM_022942501.1<br>Product: XP_022798236.1<br>NCBI: XP_022798236.1<br>Uniprot: A0A2B4RQU3	No homolog
H. miamia	Ensembl-genomes<br>GCA_004352715.1	Description: --<br>Gene: **HMIM012391**<br>Transcript: HMIM012391-RA<br>Product: HMIM012391-PA<br>NCBI: --<br>Uniprot: --	No homolog	No homolog
S. roscoffensis	UCSC genome-browser<br>SymRos_1_5	Description: `Sros151_020267`<br>Gene: Sros151_020267<br>Transcript: Sros151_020267<br>Product: Sros151_020267<br>NCBI: --<br>Uniprot: --	No homolog	No homolog
S. purpuratus	Ensembl-genomes<br>GCA_000002235.4	Description: Pax-2a-like<br>Gene: **LOC115918459**<br>Transcript: XM_030979663<br>Product: XP_030835523<br>NCBI: --<br>Uniprot: --	Description: dachshund homolog 1 like<br>Gene: **LOC115918252**<br>Transcript: XM_030981289<br>Product: XP_030837149<br>NCBI: --<br>Uniprot: --	Description: engrailed-like<br>Gene: **LOC115929921**<br>Transcript: XM_031000060<br>Product: XP_030855920<br>NCBI: XM_789660.3<br>XP_794753.1<br>Uniprot: --

			Description: Dachshund homolog 1, transcript variant X8<br>Gene: **LOC586986**<br>Transcript: XM_030980740<br>Product: XP_030836600<br>NCBI: --<br>Uniprot: --	
S. kowa-levskii	NCBI<br>GCA_000003605.1	Description: PREDICTED: Pax-2-B isoform X1<br>NCBI Gene: 100328996<br>Transcript: XM_006813003.1<br>Product: XP_006813066.1<br>Uniprot: --	Description:PREDICTED: dachshund homolog 1 isoform X3<br>NCBI Gene: 100303544<br>Transcript: XM_006824805.1<br>Product: XP_006824868.1<br>Uniprot: --	Description: en - engrailed<br>NCBI Gene: 100303466<br>Transcript: NM_001164900.1<br>Product: NP_001158372.1<br>Uniprot: Q7YTB6
B. lanceo-latum	Ensembl-genomes<br>GCA_900088365.1	Description: --<br>Gene: **BL19642**<br>Transcript: BL19642_evm1<br>Product: BL19642_evm1<br>NCBI: --<br>Uniprot: --	Description: --<br>Gene: **BL03388**<br>Transcript: BL03388_evm15<br>Product: BL03388_evm15<br>NCBI: --<br>Uniprot: --	Description: --<br>Gene: **BL18701**<br>Transcript: BL18701_cuf2<br>Product: BL18701_cuf2<br>NCBI: --<br>Uniprot: --
			Description: --<br>Gene: **BL02962**<br>Transcript: BL02962_evm3<br>Product: BL02962_evm3<br>NCBI: --<br>Uniprot: --	
C. intes-tinalis	Ensembl<br>GCA_000224145.1	Description: pax2/5/8-b<br>Gene: ENSCING00000005977<br>Transcript: ENSCINT00000012344.3<br>Product: ENSCINP00000012344<br>NCBI: --<br>Uniprot: F6VTF7	Description: novel gene<br>Gene: ENSCING00000019659<br>Transcript: ENSCINT00000037189.1<br>Product: ENSCINP00000034508<br>NCBI: --<br>Uniprot: H2XXX4	Description: en<br>Gene: ENSCING00000002157<br>Transcript: ENSCINT00000004407.3<br>Product: ENSCINP00000004407<br>NCBI: --<br>Uniprot: Q8IAC7
		Description: pax258<br>Gene: ENSCING00000006915<br>Transcript: ENSCINT00000014209.3<br>Product: ENSCINP00000014209<br>NCBI: --<br>Uniprot: F6WAV5	Description: novel gene<br>Gene: ENSCING00000000087<br>Transcript: ENSCINT00000000131.3<br>Product: ENSCINP00000000131<br>NCBI: --<br>Uniprot: F6TS32	
M. musculus	NCBI<br>GCA_000001635.9	Description: Pax-2 isoform 1<br>NCBI Gene: 18504<br>Transcript: NM_001368743.1<br>Product: NP_001355672.1<br>Uniprot: P32114	Description: dachshund 1 isoform 2<br>NCBI Gene: 13134<br>Transcript: NM_001038610.2<br>Product: NP_001033699.1<br>Uniprot: Q9QYB2	Description: engrailed-1<br>NCBI Gene: 13798<br>Transcript: NM_010133.3<br>Product: NP_034263.2<br>Uniprot: P09065
			Description: dachshund 2 isoform 1<br>NCBI Gene: 93837<br>Transcript: NM_001142570.1<br>Product: NP_001136042.1<br>Uniprot: Q925Q8	Description: engrailed-2 isoform 1<br>NCBI Gene: 13799<br>Transcript: NM_010134.4<br>Product: NP_034264.1<br>Uniprot: P09066
H. sapiens	NCBI<br>GCA_000001405.28	Description: Pax-2 isoform b<br>NCBI Gene: 5076<br>Transcript: NM_000278.5<br>Product: NP_000269.3<br>Uniprot: Q02962	Description: dachshund 1 isoform d<br>NCBI Gene: 1602<br>Transcript: NM_001366712.1<br>Product: NP_001353641.1<br>Uniprot: Q9UI36	Description: engrailed-1<br>NCBI Gene: 2019<br>Transcript: NM_001426.4<br>Product: NP_001417.3<br>Uniprot: Q05925
			Description: dachshund 2 isoform b<br>NCBI Gene: 117154<br>Transcript: NM_001139514.1<br>Product: NP_001132986.1<br>Uniprot: Q96NX9	Description: engrailed-2<br>NCBI Gene: 2020<br>Transcript: NM_001427.4<br>Product: NP_001418.2<br>Uniprot: P19622
L. anatina	Ensembl-genomes	Description: --<br>Gene: **g1632**	Description: --<br>Gene: **g13798**	Description: --<br>Gene: **g20932**

	GCA_001039355.1	Transcript: g1632.t1<br>Product: g1632.t1<br>NCBI: XP_013394332.1 and 10 other variants<br>Uniprot: --	Transcript: g13798.t1<br>Product: g13798.t1<br>NCBI: XP_013405870.1<br>Uniprot: --<br><br>Description: --<br>Gene: **g33597**<br>Transcript: g33597.t1<br>Product: g33597.t1<br>NCBI: --<br>Uniprot: --	Transcript: g20932.t1<br>Product: g20932.t1<br>NCBI: XP_013420680.1<br>Uniprot: --<br><br>Description: --<br>Gene: **g20933**<br>Transcript: g20933.t1<br>Product: g20933.t1<br>NCBI: XM_013535485.1<br>XM_024077645.1<br>XP_013390939.1<br>XP_023933413.1<br>Uniprot: --<br><br>Description: --<br>Gene: **g29453**<br>Transcript: g29453.t1<br>Product: g29453.t1<br>NCBI: XM_013535485.1<br>XM_024077645.1<br>XP_013390939.1<br>XP_023933413.1<br>Uniprot: --
C. teleta	Ensembl-genomes<br>GCA_000328365.1	Description: --<br>Gene: **CapteG119889**<br>Transcript: CapteT119889<br>Product: CapteP119889<br>NCBI: --<br>Uniprot: R7T7W6	Description: --<br>Gene: **CapteG137512**<br>Transcript: CapteT137512<br>Product: CapteP137512<br>NCBI: --<br>Uniprot: R7THV0<br><br>Description: --<br>Gene: **CapteG227573**<br>Transcript: CapteT227573<br>Product: CapteP227573<br>NCBI: --<br>Uniprot: R7TIT7	Description: EN<br>Gene: **CapteG152545**<br>Transcript: CapteT152545<br>Product: CapteP152545<br>NCBI: --<br>Uniprot: R7T5K2
O. bima-culoides	Ensembl-genomes<br>GCA_001194135.1	Description: --<br>Gene: **Ocbimv22007526m.g**<br>Transcript: Ocbimv22007526m<br>Product: Ocbimv22007526m.p<br>NCBI: XP_014769898.1<br>Uniprot: A0A0L8HRW6	Description: --<br>Gene: **Ocbimv22003564m.g**<br>Transcript: Ocbimv22003564m<br>Product: Ocbimv22003564m.p<br>NCBI: XP_014768677.1<br>Uniprot: A0A0L8HX76<br><br>Description: --<br>Gene: **Ocbimv22017627m.g**<br>Transcript: Ocbimv22017627m<br>Product: Ocbimv22017627m.p<br>NCBI: XP_014789304.1<br>Uniprot: A0A0L8FJA3	Description: --<br>Gene: **Ocbimv22020235m.g**<br>Transcript: Ocbimv22020235m<br>Product: Ocbimv22020235m.p<br>NCBI: XP_014774611.1<br>Uniprot: A0A0L8H8Q2<br><br>Description: --<br>Gene: **Ocbimv22005596m.g**<br>Transcript: Ocbimv22005596m<br>Product: Ocbimv22005596m.p<br>NCBI: XP_014786199.1<br>Uniprot: A0A0L8FWR3
S. mansoni	Ensembl-genomes<br>GCA_000237925.3	Description: Putative paired box protein pax-6<br>Gene: **Smp_142940**<br>Transcript: Smp_142940.1<br>Product: Smp_142940.1<br>NCBI: XM_018799904.1<br>XP_018653771.1<br>Uniprot: --	Description: Putative dachshund homolog<br>Gene: **Smp_196410**<br>Transcript: Smp_196410.1<br>Product: Smp_196410.1<br>NCBI: XM_018792936.1<br>XP_018647498.1<br>Uniprot: --	Description: --<br>Gene: **Smp_343800**<br>Transcript: Smp_343800.1<br>Product: Smp_343800.1<br>NCBI: XM_018789903.1<br>XP_018655299.1<br>Uniprot: --
A. vaga	Ensembl-genomes<br>GCA_000513175.1	Description: --<br>Gene: **GSADVG00030208001**<br>Transcript: GSADVT00030208001<br>Product: GSADVT00030208001<br>NCBI: --<br>Uniprot: --<br><br>Description: --<br>Gene: **GSADVG00000720001**<br>Transcript: GSADVT00000720001	Description: --<br>Gene: **GSADVG00044000001**<br>Transcript: GSADVT00044000001<br>Product: GSADVT00044000001<br>NCBI: --<br>Uniprot: --	Description: --<br>Gene: **GSADVG00002773001**<br>Transcript: GSADVT00002773001<br>Product: GSADVT00002773001<br>NCBI: --<br>Uniprot: --

		Product:<br>GSADVT00000720001<br>NCBI: --<br>Uniprot: --		
		Description: --<br>Gene:<br>**GSADVG00066437001**<br>Transcript:<br>GSADVT00066437001<br>Product:<br>GSADVT00066437001<br>NCBI: --<br>Uniprot: --	Description: --<br>Gene: **GSADVG00042435001**<br>Transcript:<br>GSADVT00042435001<br>Product: GSADVT00042435001<br>NCBI: --<br>Uniprot: --	Description: --<br>Gene: **GSADVG00021246001**<br>Transcript: GSADVT00021246001<br>Product: GSADVT00021246001<br>NCBI: --<br>Uniprot: --
		Description: --<br>Gene:<br>**GSADVG00003964001**<br>Transcript:<br>GSADVT00003964001<br>Product:<br>GSADVT00003964001<br>NCBI: --<br>Uniprot: --		
		Description: --<br>Gene:<br>**GSADVG00040493001**<br>Transcript:<br>GSADVT00040491001<br>Product:<br>GSADVT00040491001<br>NCBI: --<br>Uniprot: --	Description: --<br>Gene: **GSADVG00020231001**<br>Transcript:<br>GSADVT00020231001<br>Product: GSADVT00020231001<br>NCBI: --<br>Uniprot: --	Description: --<br>Gene: **GSADVG00005228001**<br>Transcript: GSADVT00005228001<br>Product: GSADVT00005228001<br>NCBI: --<br>Uniprot: --
		Description: --<br>Gene:<br>**GSADVG00040499001**<br>Transcript:<br>GSADVT00040499001<br>Product:<br>GSADVT00040499001<br>NCBI: --<br>Uniprot: --	Description: --<br>Gene: **GSADVG00018241001**<br>Transcript:<br>GSADVT00018241001<br>Product: GSADVT00018241001<br>NCBI: --<br>Uniprot: --	Description: --<br>Gene: **GSADVG00036502001**<br>Transcript: GSADVT00036502001<br>Product: GSADVT00036502001<br>NCBI: --<br>Uniprot: --
D. melano-gaster	NCBI<br>GCA_000<br>001215.4	Description: shaven, isoform A<br>NCBI Gene: 43825<br>Transcript: NM_079894.4<br>Product: NP_524633.3<br>Uniprot: O16117	Description: dachshund, isoform G<br>NCBI Gene: 34982<br>Transcript: NM_001014486.3<br>Product: NP_001014486.1<br>Uniprot: Q59DZ0	Description: engrailed, isoform B<br>NCBI Gene: 36240<br>Transcript: NM_165841.2<br>Product: NP_725059.1<br>Uniprot: P02836
				Description: invected, isoform D<br>NCBI Gene: 36239<br>Transcript: NM_165839.3<br>Product: NP_725057.2<br>Uniprot: P05527
H. exem-plaris	Ensembl-genomes<br>GCA_002<br>082055.1	Description: Paired box protein Pax-2<br>Gene: **BV898_05868**<br>Transcript:<br>mrna.BV898_05868.2<br>Product: OQV20075.1<br>NCBI: OQV20075.1<br>Uniprot: A0A1W0WXZ8	No homolog	Description: putative Homeobox protein engrailed<br>Gene: **BV898_02358**<br>Transcript: mrna.BV898_02358.1<br>Product: OQV23613.1<br>NCBI: OQV23613.1<br>Uniprot: A0A1W0X7Y3
C. elegans	Ensembl-genomes<br>GCA_000<br>002985.3	Description: pax-2<br>Gene: **WBGene00003938**<br>Transcript: K06B9.5a.1<br>Product: K06B9.5a.1<br>NCBI: NP_500513<br>Uniprot: Q21263	Description: dac-1<br>Gene: **WBGene00000895**<br>Transcript: B0412.1a.1<br>Product: B0412.1a.1<br>NCBI: NP_001021129<br>Uniprot: H2KY91	Description: ceh-16<br>Gene: **WBGene00000439**<br>Transcript: C13G5.1.1<br>Product: C13G5.1.1<br>NCBI: --<br>Uniprot: P34326
		Description: egl-38<br>Gene: **WBGene00001204**<br>Transcript: C04G2.7.1<br>Product: C04G2.7.1<br>NCBI: NP_501836		

		Uniprot: G5ED14		
P. caudatus	NCBI GCA_000485595.2	Description: paired box protein Pax-5-like<br>NCBI Gene: 106819112 (LOC106819112)<br>Transcript: XM_014823776.1<br>Product: XP_014679262.1<br>Uniprot: --	No homolog	Description: engrailed-1a-like<br>NCBI Gene: 106809848 (LOC106809848)<br>Transcript: XM_014813077.1<br>Product: XP_014668563.1<br>Uniprot: --

Table S3. Predicted transcription factor binding sites in CRE-like sequences.

	H. sapiens / M. musculus / D. melanogaster	*S. purpuratus*	*B. lanceolatum*	*C. intestinalis*	*L. anatina*	*C. teleta*	*O. bimaculoides*	*S. mansoni*	*A. vaga*	*H. exemplaris*	*C. elegans*
sv/PAX2	*Ascl1 Dlx1-6 Emx1,2 En1,2 Gbx1,2 Isl1,2 Lhx1-6, 8,9 Lmx1A Meis1-3 Msx1-3 Nkx2-3, 2-5, 2-8, 3-1, 6-1, 6-2, 6-3 Pax2 Phox2A Pitx2 Sox3,4, 8,9,15,18, Tlx2*	*Dlx Lhx Meis Nr2e1 Pax2 Sox*	*Ascl Atoh Dlx FoxA Lhx Nkx Sox*	*Atoh Dlx Emx En FoxA Gbx Isl Lhx Lmx Meis Msx Nkx Pbx Phox2 Pitx Tlx*	*Dlx Emx En FoxA Gbx Isl Lhx Msx Nkx Pax2 Pitx Sox Tlx*	*Lhx Nkx Runx Sox*	--	*Dlx En Gbx Lhx Meis Msx Nkx Phox2 Pitx Sox Tlx*	--	*Ascl Atoh Dlx En FoxA Isl Lhx Meis Nkx Pax2 Ptf1a Sox*	*Nkx Runx Sox*
dac/DACH1	*Emx1,2 En1,2 FoxA1-3 Gbx1,2 Lhx1-6,8,9 Meis2 Msx1-3 Nkx2-3, 2-5, 2-8, 3-1, 6-1, 6-2, 6-3 Nr2e1 Pax2 Pbx2 Phox2A, Phox2B Sox4,8, 15,18 Tlx2*	--	*Emx En FoxA Gbx Lhx Meis Msx Nkx Pax2 Pbx Pitx Sox Tlx*	*Ascl Emx En Lhx Nkx Otx Pitx Ptf1a Sox*	*Emx En Gbx Isl Lhx Msx Nkx Sox Tlx*	*Meis Sox*	*En FoxA Lhx Meis Pax2 Sox*	*Ascl Atoh Lhx Ptf1a Sox*	*Emx En Gbx Isl Lhx Meis Msx Nkx Otx Pax2 Pbx Phox Pitx Tlx*	--	*Emx En Lhx Nkx Sox*

inv/EN2	*Atoh1,7<br>Emx1,2<br>En1,2<br>Gbx1,2<br>Isl1,2<br>Lhx1,2,4<br>-6,8,9<br>Meis1-3<br>Msx1-3<br>Nkx2-3,<br>2-5, 2-8,<br>3-1, 6-1,<br>6-2, 6-3<br>Otx1,2<br>Pax2<br>Phox2A,<br>Phox2B<br>Pitx1-3<br>Runx1-3<br>Sox3,4,<br>6,8,10,1<br>5,18,<br>Tlx2*	*Ascl<br>Atoh<br>Emx<br>En<br>Gbx<br>Isl<br>Lhx<br>Msx<br>Nkx<br>Otx<br>Pax2<br>Pitx<br>Ptf1a<br>Sox<br>Tlx*	*Ascl<br>Atoh<br>Emx<br>En<br>FoxA<br>Gbx<br>Isl<br>Lhx<br>Meis1<br>Msx<br>Nkx<br>Pax2<br>Phox2<br>Pitx<br>Sox<br>Tlx*	*En<br>Lhx<br>Meis<br>Nkx<br>Otx<br>Pitx<br>Sox*	*Emx<br>En<br>FoxA<br>Gbx<br>Isl<br>Lhx<br>Meis<br>Msx<br>Nkx<br>Otx<br>Pax2<br>Phox2<br>Pitx<br>Runx<br>Sox<br>Tlx*	*FoxA<br>Lhx<br>Pax2*	*Atoh<br>Isl<br>Lhx<br>Meis<br>Nkx<br>Pax2<br>Sox*	*En<br>FoxA<br>Lhx<br>Msx<br>Nkx<br>Sox*	*FoxA<br>Isl<br>Lhx<br>Meis<br>Nkx<br>Pax2<br>Sox*	*Dlx<br>Emx<br>En<br>Isl<br>Lhx<br>Nkx<br>Pax2*	*Ascl<br>Lhx<br>Meis<br>Nr2e3<br>Ptf1a<br>Runx<br>Sox*

Putative transcription factor binding sites contained in *sv/PAX2*, *dac/DACH1* and *inv/EN2* CREs and CRE-like sequences. For number, position, core and matrix match, see supplementary file S3. Binding sites identified using Ciiider (http://www.ciiider.org).

Table S4: Cell cluster-specific gene expression in the nervous system of the *Ciona intestinalis* tadpole larva.

M. musculus	*D. melanogaster*	*C. intestinalis*	ANB	pro-aSV	aSV	SV	pSV	neck	MHB	MG	Trunk	Tail	Un-assigned
Six3	*optix*	KH.C10.367	-	Arx, Six3/6	-	-	-	-	-	-	-	-	-
Otx	*otd*	KH.C4.84	-	Arx, Six3/6	Rx, Lox5	Hedgehog2	GLGB	-	-	-	-	-	Pigment cells
Gbx	*unplugged*	n/a	-	-	-	-	-	-	-	-	-	-	-
FGF8/17/18	*pyr/ths/bnl*	KH.C5.5	-	-	-	-	-	-	-	✓	-	-	Ependymal
Wnt1	*wingless*	n/a	-	-	-	-	-	-	-	-	-	-	-
En1/2	*en/inv*	KH.C7.431	-	-	-	-	✓	-	-	✓	-	-	Ependymal
Pax2	*shaven*	A: KH.S1363.2	Pitx	-	-	-	-	Pax258A	-	-	-	-	-
		B: KH.C12.116	-	Six3/6	-	-	-	-	-	-	-	-	-
Dach1	*dac-1*	KH.C14.291	-	-	-	-	-	-	MHB	-	-	-	FoxD-b Collocytes aATENs
Hoxb1	*lab*	KH.L171.16	-	-	-	-	-	✓	-	✓	NC (A)	-	FoxD-b
Msx	*msh*	KH.C2.957	-	-	-	-	-	-	-	-	NC (b)	-	-

KH nomenclature refers to cell clusters established by single cell RNA sequencing (46). Abbreviations: Pro-aSV, pro-anterior sensory vesicle; aSV, anterior sensory vesicle; SV, sensory vesicle; pSV, posterior sensory vesicle; MHB, midbrain hindbrain boundary; Neck, Pax2/5/8-A-positive neck cells; MG, motor ganglion. Checkmark indicates expression data established by ref. 45.

Table S5: Cell cluster-specific gene expression in larval head ganglia of *Platynereis dumerilii*.

	FG	AG	DG	PG	VMG	CpG	MB	AE	CG
Six4	0	**46**	**116**	142	0	40	140	9	14
Wnt5	**33**	27	1	159	2	21	100	7	11
Pax2/5/8	0	**85**	0	31	0	26	36	0	20
Dac	1	**32**	**167**	245	6	**53**	**596**	10	14
Hox1	0	0	0	3	0	5	34	0	**154**
Msx	0	8	2	2	0	13	1	0	3

Abbreviations: FG, frontal ganglia; AG, antennal ganglion; DG, dorsal ganglia; PG, palpal ganglia; VMG, ventromedial ganglia; CpG, circumpalpal ganglia; MB, mushroom bodies; AE, adult eyes; CG, cirral ganglia. Indicated are number of cells of each ganglion expressing the respective gene with an expression level of >=0.1; numbers in bold indicate genes expression level of at least 0.7 in over 5 cells of the respective ganglion. Source: ref. 48.

Table S6: Six3, Otx, Engrailed, GBX, Hox1, Hox4 and Lox5 gene expression in *Platynereis dumerilii* head ganglia.

Steinmetz	Protostomium	Peristomium	Metastomium			
			I	II	III	IV
Six3	X					
Otx		X				
En			*Demarcating each segment*			
Gbx			X			
Hox1				X		
Hox4					X	
Lox5						X

Source: refs. 54, 55.

Table S7: Identified cell types, their modality and strata and layer-specific allocation in the brain of *Caenorhabditis elegans*.

	Stratum 1	Layer	Cell type	Stratum 2	Layer	Cell type	Stratum 3	Layer	Cell type	Stratum 4	Layer	Cell type	Un-assigned	Layer	Cell type
**LAYER 1**	**CEP**	s	Mod	ADE	L1, s	Mod	ALM	L1, s, i	Sens	ADF	L1, s	Sens	FLP	L1 M3, s	Sens
	**IL1**	L1, s	Mot				AQR	L1, s	Sens	AFD	L1, s	Sens	URX	L1 M4 n/a	Sens
	**IL2**	L1, s	Sens				ASH	L1, s	Sens	ASE	L1, s	Sens	AWA	L1 M4, s	Sens
	**OLL**	L1, s	Sens				AVM	L1, s	Sens	ASG	L1, s	Sens			
	**OLQ**	L1, s	Sens				ASJ	L1 (M4) s	Sens	ASI	L1, s	Sens			
	**URY**	L1, s	Sens				ASK	L1 (M4) s	Sens	AWB	L1, s	Sens			
							ADL	L1 (M4) s	Sens	AWC	L1, s	Sens			
										BAG	L1, s	Sens			
**LAYER 2**	**RIP**	L2, i	Int	ALN	L2, i	Sens	AVJ	L2, i	Mod	AIA	L2, i	Int	SDQ	L2 M2, i	Sens
	**URA**	L2, m	Mot	PVT	L2, i	Int	AIM	L2 (M4) i	Mod	AIN	L2, i	int			
							PVQ	L2 (M4) i	Mod	AIY	L2, i	Int			
							BDU	L2, i	Int						
**LAYER 3**	**RIA**	L3, i	Int	AVK	L3, i	Mod	DVA	L3 (M2) i	Mod	AUA	L3, i	Sens	AVE	L3 M1, i	Int
	**RIH**	L3, i	Int	DVC	L3, i	Int	ADA	L3, i	Int				RIG	L3 M2, i	Int
	**RMDD**	L3, m	Mot	RIC	L3, i	Mod	AVB	L3, i	Int				RIM	L3 M2, i	Int
	**RMDV**	L3, m	Mot	SAA	L3, i	Sens	AVH	L3, i	Mod				RIS	L3 M2, i	Mod
	**RME**	L3, m	Mot	RMF	L3, i	Mot	PVP	L3, i	Int				RMG	L3 M2, i	Mod
	**URB**	L3 (M2) i	Sens	RMH	L3, m	Mot	PVC	L3, i	Int				AVA	L3 M2 n/a	Int
				SIA	L3, m	mot	RIF	L3, i	Int				RIR	L3 M3, n/a	Int
				SIBD	L3, m	Mot	ALA	L3 (M4) i	Mod				AIB	L3 M4, i	Int
				SMB	L3, m	Mot	AVF	L3 (M4) i	Mod				AIZ	L3 M4, i	Int
				SMD	L3, m	Mot	HSN	-	Mod				RIB	L3 M4, i	Int
				RIV	L3, m	Mot	AVD	-	Int				SIBV	-	Mot
				RMD	L3, m	Mot	PVN	-	Mod				PVR	-	Int
				AVL	-	Mod	RID	-	Mod				VB01		-
				PLN	-	Sens									

Cell type/name of neuron according to wormatlas (https://www.wormatlas.org/index.html). Abbreviations: L, Layer; M, Module; Mod, modulatory neuron; m/Mot, motor neuron; s/Sens, sensory cell; i/Int, interneuron. Brackets indicate variability depending on source: refs. 41, 56-58.

Table S8: Layer and cell type-specific gene expression in the brain of *Caenorhabditis elegans*.

M. m.	***D. m.***	***C. e.***	**Expressing neurons**				
Six3	*optix*	*ceh-32*	RIA RME URY RMD IL1 OLL OLQ	RMD RMH	RID	-	RIB
Otx	*otd*	*ttx1*	RIP	-	-	AFD	RIB
Gbx	*unplugged*	*n/a*	n/a	n/a	n/a	n/a	n/a
FGF8/ 17/18	*pyr/ths/bnl*	*let-756*	IL2 OLQ URA	PLN SIB RMH SIA SMD	ALM AVF ALA DVA AVB AVH ASJ AVM	AIY	SIB RIG RIS AVE PVR
Wnt1	*wingless*	*wwn-1*	-	-	HSN	-	-
En1/2	*en/inv*	*ceh-16*	-	-	RIF ASJ	-	RIG AIZ
Pax2	*shaven*	*pax-2*	-	-	ASK PVC AQR	-	-
Dach1	*dac-1*	*dac-1*	-	-	ASK	AFD ASE ASI	-
Hoxb1	*lab*	*ceh-13*	RME	DVC	AVH	-	RMG
Msx	*msh*	*vab-15*	-	PVT	PVC PVQ	-	-
			**S1**	**S2**	**S3**	**S4**	*unassign.*
			**Brain-specific stratum**				

Gene expression in identified neurons, the names of which follow the wormatlas nomenclature (https://www.wormatlas.org/index.html). Individual neurons are further grouped according to their location within a brain-specific stratum, S1-S4, or if unclear designated as unassigned (*unassign.*). Abbreviations: *M. m., Mus musculus*; *D. m., Drosophila melanogaster*; *C. e., Caenorhabditis elegans*. Source: ref. 57; CeNGEN (https://cengen.shinyapps.io/CengenApp/).

Supplementary Data Set S1

Non-Bilateria

Shown are the results of EMBOSS matcher sequence comparisons between the superconserved CREs identified in ref. 8 and the respective homologs in each of the investigated non-bilaterian species. A match meeting the cutoff criteria of 60% sequence identity and 55bp length was blasted using Ensembl Blastn to identify its e-value (cutoff: 1e-1) and its position (criteria: intronic position). Each search was carried out with a) the sense and b) the antisense strand of the respective gene.

S1.1. *shaven/PAX2*

1. Mnemiopsis leidyi

1.a Gene ML07884a

Gene: **ML07884a**
Transcript: ML07884a-RA
Product: ML07884a-PA

A

```
# Aligned_sequences: 2
# 1: ML07884_sense
# 2: sv/PAX2
# Matrix: EDNAFULL
# Gap_penalty: 16
# Extend_penalty: 4
#
# Length: 148
# Identity:      86/148 (58.1%)
# Similarity:    86/148 (58.1%)
# Gaps:          19/148 (12.8%)
# Score: 98
#
#
#=======================================

ML07884_sense   1469 CCCAATTCGGTTCATCTATTTAAGCAGGAATAATTTTGTTTCTCTGCGCG   1518
                     |||||.|.|.||..||.||   ||||||||.|    ||..|| |||   .
sv/PAX2           63 CCCAAATTGCTTATTCAAT---AGCAGGAACA----TGGCTC-CTG---A    101

ML07884_sense   1519 ATCAATGACGAGAATTTTAAATGCGATTTTCTTGTTAGAGTGAA-ATGTC   1567
                     ||.||...|.|||||||   |||.||    ||.|...||||.|. |.|||
sv/PAX2          102 ATAAAGTCCCAGAATTT---ATGTGA----CTCGCACGAGTCAGGAGGTC    144

ML07884_sense   1568 ATTTAACTGCTAAGGAGCTGAATCGAGCACACGATTAAACCCTTGGCA   1615
                     |...|||||.||.|||||..|.|..|..|..|.|.||||...|||.||
sv/PAX2          145 AAACAACTGTTATGGAGCGAAGTTAAAAATCCAAATAAAATATTGACA    192
```

→ below the threshold criteria

B

```
# Aligned_sequences: 2
# 1: ML07884_antisense
# 2: sv/PAX2
# Matrix: EDNAFULL
# Gap_penalty: 16
# Extend_penalty: 4
#
# Length: 52
# Identity:      34/52 (65.4%)
# Similarity:    34/52 (65.4%)
# Gaps:           1/52 ( 1.9%)
# Score: 86
#
#
#=======================================

ML07884_anti   10931 CTTATAAACTAGCTATGCACATAACCAACTTATAAACTCCCCCAATCTAT  10980
                     |||||..|.|||| |.|.||||..|......|||||.||||..|||.|||
sv/PAX2             72 CTTATTCAATAGC-AGGAACATGGCTCCTGAATAAAGTCCCAGAATTTAT    120

ML07884_anti   10981 GT  10982
                     ||
sv/PAX2            121 GT    122
```

→ below the threshold criteria

1.b Gene ML06935a

Gene: **ML06935a**
Transcript: ML06935a-RA
Product: ML06935a-PA

A

```
# Aligned_sequences: 2
# 1: ML06935_sense
# 2: sv/PAX2
# Matrix: EDNAFULL
# Gap_penalty: 16
# Extend_penalty: 4
#
# Length: 141
# Identity:      81/141 (57.4%)
# Similarity:    81/141 (57.4%)
# Gaps:          12/141 ( 8.5%)
# Score: 93
#
#
#=======================================

ML06935_sense   5273 TGGAAAATAAATGTATTTGTGCTAAAAGTT-GTT-CATTACCTTAAGAAT   5320
                     |||.||..|||.||||||....||....|| ||| ||.|.||...||.||
sv/PAX2            213 TGGGAAGCAAAGGTATTTAAAATAGGTTTTTGTTGCAATGCCCCCAGGAT    262

ML06935_sense   5321 AATGAATTTAAGTACTATAGACCTCA-ACAACCTGGACTTTTTGATTTTT   5369
                     ||.||||..|||.|     |||...| ||||..||||.|||.|.|..|..
sv/PAX2            263 AAAGAATGGAAGGA-----GACTGAAGACAAGTTGGAGTTTATAAAATCA    307

ML06935_sense   5370 CTTG---TTATCACTATTACCACTGACCAGTTAATTATACT   5407
                     .|.|   ||||..| |...|.|||...||.|||||.||.||
sv/PAX2            308 ATGGAATTTATTGC-ACAGCTACTTGGCATTTAATAATTCT    347
```

→ below the threshold criteria

B

```
# Aligned_sequences: 2
# 1: ML06935_antisense
# 2: sv/PAX2
# Matrix: EDNAFULL
# Gap_penalty: 16
# Extend_penalty: 4
#
# Length: 95
# Identity:      58/95 (61.1%)
# Similarity:    58/95 (61.1%)
# Gaps:           8/95 ( 8.4%)
# Score: 94
#
#
#=======================================

ML06935_antis   8793 ATTGGTTTTTGTTGTGGTCAAAGAAACCAATTTAATAAATGGCGCGAAAA   8842
                     ||.|||||||||||    |||.|...|||...|||..|||||...||.|.
sv/PAX2          234 ATAGGTTTTGTTG----CAATGCCCCCAGGATAAAGAATGGAAGGAGAC    279

ML06935_antis   8843 GTTTTA-AATTTTCA--TTAAAAAA-CAATTGAATAAATCGCAGA   8883
                     .....| ||.||..|  |||.|||| ||||.||||..||.|||.|
sv/PAX2          280 TGAAGACAAGTTGGAGTTTATAAAATCAATGGAATTTATTGCACA    324
```

→ **Hit:**

>ML06935_antisense_CRE-like
ATTGGTTTTTGTTGTGGTCAAAGAAACCAATTTAATAAATGGCGCGAAAAGTTTTAAATTTTCATTAAAAAACAATTGAATAAATCGCAGA

→ **BLASTN SEARCH** for ML06935_antisense_CRE-like: e-value 4.1e-45

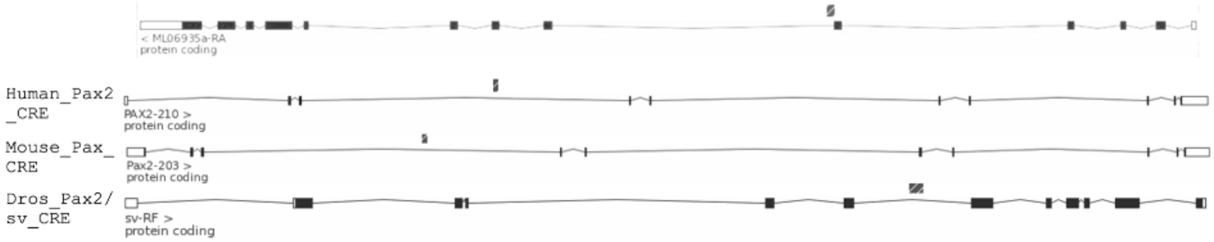

2. *Amphimedon queenslandica*

Gene: **Aqu2.1.37531**
Transcript: Aqu2.1.37531_001
Product: Aqu2.1.37531_001

A

```
# Aligned_sequences: 2
# 1: Aqu2.1.37531_sense
# 2: sv/PAX2
# Matrix: EDNAFULL
# Gap_penalty: 16
# Extend_penalty: 4
#
# Length: 177
# Identity:     100/177 (56.5%)
# Similarity:   100/177 (56.5%)
# Gaps:          16/177 ( 9.0%)
# Score: 96
#
#
#=======================================
Aqu2.1.37531_s   921 TCTTGACCGTTTTCCCAGCAGCTGCTACAACAACTCAGCAGTCCGCCACT    970
                     ||||.|..|..||.||||||.|.||    ||||.|||..|.|.|.|| ||
sv/PAX2           18 TCTTCAGGGACTTTCCAGCAACAGC----ACAAGTCATTAATTCACC-CT     62

Aqu2.1.37531_s   971 CCTAGCATGCCGTATTTTGTCTATCCAACAATTCCCCCGACTACCGGAGG   1020
                     ||.|. ||..|.||||...|......||||...|.||.||.||..|....
sv/PAX2           63 CCCAA-ATTGCTTATTCAATAGCAGGAACATGGCTCCTGAATAAAGTCCC    111

Aqu2.1.37531_s  1021 AGGCGATA-GTGTCTCTT----GTCACCAGGTTAGAAAAACTCGTTATTT   1065
                     ||....|| |||.|||..    ||||..||||.| ||.|||| |||||..
sv/PAX2          112 AGAATTTATGTGACTCGCACGAGTCAGGAGGTCA-AACAACT-GTTATGG    159

Aqu2.1.37531_s  1066 ATAATATTTGAATTAAATCTAATTAAA   1092
                     |....|.||.||   ||||.||.||||
sv/PAX2          160 AGCGAAGTTAAA---AATCCAAATAAA    183
```

→ below the threshold criteria

B

```
# Aligned_sequences: 2
# 1: Aqu2.1.37531_antisense
# 2: sv/PAX2
# Matrix: EDNAFULL
# Gap_penalty: 16
# Extend_penalty: 4
#
# Length: 108
# Identity:      62/108 (57.4%)
# Similarity:    62/108 (57.4%)
# Gaps:           7/108 ( 6.5%)
# Score: 78
#
#
#=======================================

Aqu2.1.37531_a   384 AGGCAGCTAGCATGGACAATGCAATTAATGTGTA-ATTTGTATGAATT-A    431
                     |.||..|.||.||..|.||||.||..|...||.| |...||..||.|| |
sv/PAX2          250 ATGCCCCCAGGATAAAGAATGGAAGGAGACTGAAGACAAGTTGGAGTTTA    299

Aqu2.1.37531_a   432 TAAGCTTAATGATAATGATGAAAGAGATACATTGCGAAAATAAAATAATC    481
                     |||..|.||||..|.|.||...|.||.|||.|.||    ||..||||||.
sv/PAX2          300 TAAAATCAATGGAATTTATTGCACAGCTACTTGGC----ATTTAATAATT    345

Aqu2.1.37531_a   482 ACTTTCAT    489
                      ||.||||
sv/PAX2          346 -CTCTCAT    352
```

→ below the threshold criteria

3. Thelohanellus kitauei

No *shaven/PAX2* gene found.

4. Hydra vulgaris

Gene: **LOC100192231**
Transcript: XM_002159581.3
Product: XP_002159617.3

A

```
# Aligned_sequences: 2
# 1: sv_PAX2
# 2: XM_002159581.3_sense
# Matrix: EDNAFULL
# Gap_penalty: 16
# Extend_penalty: 4
#
# Length: 52
# Identity:      35/52 (67.3%)
# Similarity:    35/52 (67.3%)
# Gaps:           0/52 ( 0.0%)
# Score: 107
#
#
#=======================================

sv_PAX2          146 AACAACTGTTATGGAGCGAAGTTAAAAATCCAAATAAAATATTGACACTT    195
                     |||||.|.|.||..|...||.|.|||||...|||.|||.||||||.||.|
XM_002159581.   6296 AACAAGTATCATATATATAACTAAAAAAAATAAAAAAATTATTGAAACCT   6345

sv_PAX2          196 TT    197
                     ||
XM_002159581.   6346 TT   6347
```

→ **below the threshold criteria**

B

```
# Aligned_sequences: 2
# 1: sv_PAX2
# 2: XM_002159581.3_antisense
# Matrix: EDNAFULL
# Gap_penalty: 16
# Extend_penalty: 4
#
# Length: 70
# Identity:      45/70 (64.3%)
# Similarity:    45/70 (64.3%)
# Gaps:           6/70 ( 8.6%)
# Score: 89
#
#
#=======================================

sv_PAX2          290 TTGGAGTTTATAAAATCAATGGAATTTATTGCA-CAG--CTACTTGGCAT    336
                     ||..|||||||..|||.||   |||||..|.|| ||.  |.||||.|..|
XM_002159581.   4354 TTCTAGTTTATTTAATAAA---AATTTGATACATCATTACAACTTTGTTT   4400

sv_PAX2          337 TTAATAATTCTCTCATTTCC    356
                     ||||||.|..|.|..|||||
XM_002159581.   4401 TTAATACTGATTTTTTTTCC   4420
```

→ **Hit:**

```
>XM_002159581.3_antisense_CRE-like
TTCTAGTTTATTTAATAAAAATTTGATACATCATTACAACTTTGTTTTTAATACTGATTTTTTTTCC
```

→ **BLASTN SEARCH** for XM_002159581.3_antisense_CRE-like: e-value 3.1e-30

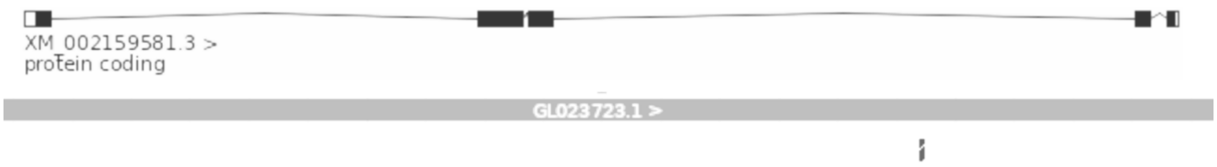

5. *Clytia hemisphaerica*

Gene: **CLYHEMG014262**
Transcript: CLYHEMT014262.1
Product: CLYHEMP014262.1

A

```
# Aligned_sequences: 2
# 1: CL YHEMT014262_sense
# 2: sv/PAX2
# Matrix: EDNAFULL
# Gap_penalty: 16
# Extend_penalty: 4
#
# Length: 71
# Identity:      44/71 (62.0%)
# Similarity:    44/71 (62.0%)
# Gaps:           2/71 ( 2.8%)
# Score: 88
#
#
#=======================================

CL YHEMT0142   10453 GGATGTTAAA-ATTTCTTTTAGAGACGAGTTTTTATATATAAATAGTATA  10501
                     |||.||.||| |..|.||.|.||| |||..||..|.||..|||||..|||
sv/PAX2          138 GGAGGTCAAACAACTGTTATGGAG-CGAAGTTAAAAATCCAAATAAAATA    186

CL YHEMT0142   10502 ATTTCGTTATTTTTGGTATGG  10522
                     .|..|..|.||||.||.|.||
sv/PAX2          187 TTGACACTTTTTTGGGGAGGG    207
```

→ **Hit:**
>CL YHEMT014262_sense_CRE-like
GGATGTTAAAATTTCTTTTAGAGACGAGTTTTTATATATAAATAGTATAATTTCGTTATTTTTGGTATGG

→ **BLASTN SEARCH** for CL YHEMT014262_sense_CRE-like: e-value 2.7e-32

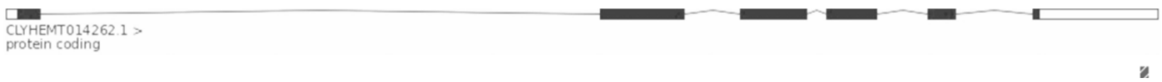

→ **does not meet criteria:** lies on UTR!

B

```
# Aligned_sequences: 2
# 1: CL YHEMT014262_antisense
# 2: sv/PAX2
# Matrix: EDNAFULL
# Gap_penalty: 16
# Extend_penalty: 4
#
# Length: 112
# Identity:      68/112 (60.7%)
# Similarity:    68/112 (60.7%)
# Gaps:          10/112 ( 8.9%)
# Score: 92
#
#
#=======================================

CL YHEMT01426   1367 ATTTTAATGATTTTG--GGGAATTTAGCCAAATATTCATCA-AAGGCA--   1411
                     ||||||||..||.|.  ||||.|||  |||...|  ||.|| |||.||
sv/PAX2            7 ATTTTAATATTTCTTCAGGGACTTT--CCAGCAA--CAGCACAAGTCATT     52

CL YHEMT01426   1412 AAATCACTCGTCAAATTTTCTACTTTAATCGGATTAACTTT-CGCGAGAT   1460
                     ||.||||.|..|.||.||.||..||.|||.|.|..|||.|. |.|..||.
sv/PAX2           53 AATTCACCCTCCCAAATTGCTTATTCAATAGCAGGAACATGGCTCCTGAA    102

CL YHEMT01426   1461 TAAATTTCGCGA   1472
                     ||||.|.|..||
sv/PAX2          103 TAAAGTCCCAGA    114
```

→ **Hit:**

>CL YHEMT014262_antisense_CRE-like
ATTTTAATGATTTTGGGGAATTTAGCCAAATATTCATCAAAGGCAAAATCACTCGTCAAATTTTCTACTTTAATCGGATTAACTTTCGCGAGATTAA
ATTTCGCGA

→ **BLASTN SEARCH** for CL YHEMT014262_antisense_CRE-like: e-value 1.5e-53

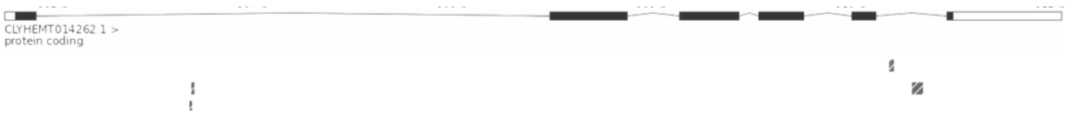

6. Dendronephthya gigantea

Gene: **LOC114524477**
Transcript: XM_028545568.1
Product: XP_028401369.1

A

```
# Aligned_sequences: 2
# 1: sv_PAX2
# 2: XM_028545568.1_sense
# Matrix: EDNAFULL
# Gap_penalty: 16
# Extend_penalty: 4
#
# Length: 107
# Identity:      64/107 (59.8%)
# Similarity:    64/107 (59.8%)
# Gaps:           5/107 ( 4.7%)
# Score: 100
#
#
#=======================================
sv_PAX2            7 ATTTTAATATTTCTTCAGGGACTTTCCAGCAACA-GCACAAGTCATTAAT     55
                     ||.||.|.||||.||.||....|.|.|||.|..| |.|..|.||||||.|
XM_028545568.   3329 ATCTTTAAATTTATTTAGACCATATTCAGTATTATGTATTATTCATTACT   3378

sv_PAX2           56 TCACCCTCCCAAATTGCTTATTCAATAGCAGGAACATGGCTCCTGAATAA    105
                       |..||.. |||...||.||||..||. |..|..|||||||.||.||||
XM_028545568.   3379 --ATGCTAA-AAAAGCCTAATTCTCTAT-ACTATGATGGCTCTTGCATAA   3424

sv_PAX2          106 AGTCCCA    112
                     .||||.|
XM_028545568.   3425 TGTCCTA   3431
```

→ below the threshold criteria

B

```
# Aligned_sequences: 2
# 1: sv_PAX2
# 2: XM_028545568.1_antisense
# Matrix: EDNAFULL
# Gap_penalty: 16
# Extend_penalty: 4
#
# Length: 92
# Identity:      58/92 (63.0%)
# Similarity:    58/92 (63.0%)
# Gaps:          12/92 (13.0%)
# Score: 94
#
#
#=======================================
sv_PAX2          214 GGGAAGCAAAGGTATTTAAAATAGGTTTTTGTTGCAATGCCCCCAGGATA    263
                     ||.|||.||   |||...|||.||||||||.|.|   |.|...|||.|||
XM_028545568   10097 GGTAAGAAA---TATGATAAAAAGGTTTTTATGG---TACAAACAGTATA  10140

sv_PAX2          264 A-AGAATGGAA--GGAGACTGAAGACA---AGTTGGAGTTTA    299
                     | |.||||..|  |||||.|.||.|||   |||.||..||||
XM_028545568   10141 ACACAATGACAATGGAGAATCAATACATTCAGTCGGGATTTA  10182
```

→ Hit:

```
>XM_028545568.1_antisense_CRE-like
GGTAAGAAATATGATAAAAAGGTTTTTATGGTACAAACAGTATAACACAATGACAATGGAGAATCAATACATTCAGTCGGGATTTA
```

→ BLASTN SEARCH for XM_028545568.1_antisense_CRE-like: e-value 6.7e-42

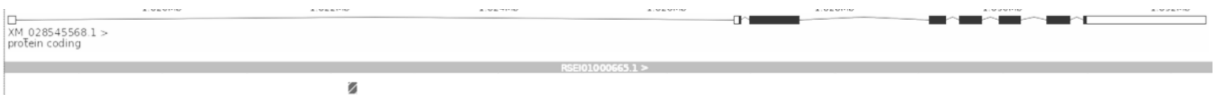

7. Actinia equina

Gene: **EGACTEQ4350035697**
Transcript: EGACTEQ4350035697-RA
Product: EGACTEQ4350035697-PA

A

```
# Aligned_sequences: 2
# 1: sv_PAX2
# 2: EGACTEQ4350035697-RA_sense
# Matrix: EDNAFULL
# Gap_penalty: 16
# Extend_penalty: 4
#
# Length: 99
# Identity:      60/99 (60.6%)
# Similarity:    60/99 (60.6%)
# Gaps:          11/99 (11.1%)
# Score: 96
#
#
#=======================================

sv_PAX2          101 AATAAAGTCCCAGAATTTATGTGACTCGCA-CGAGTCAGGAGGTCAAACA    149
                     |.||||  |.||.|..||.||||....||| |||......||||.|.|.|
EGACTEQ435003   5449 AGTAAA--CGCAAACATTCTGTGGAAAGCAACGACAGTACAGGTAAGAAA   5496

sv_PAX2          150 ACTGTTATGGAGCGAAGTTAAAAATCCAAATAAAATATTGACACTTTTT    198
                     |||.||      |.|..||||.||  |.||.||||.|||||||.|||||
EGACTEQ435003   5497 ACTTTT------CAACCTTAACAA--CGAAGAAAAAAATTGACAGTTTTT   5537
```

→ **Hit:**

```
>EGACTEQ4350035697-RA_sense_CRE-like
AGTAAACGCAAACATTCTGTGGAAAGCAACGACAGTACAGGTAAGAAAACTTTTCAACCTTAACAACGAAGAAAAAAATTGACAGTTTTT
```

→ **BLASTN SEARCH** for EGACTEQ4350035697-RA_sense_CRE-like: e-value 1.6e-43

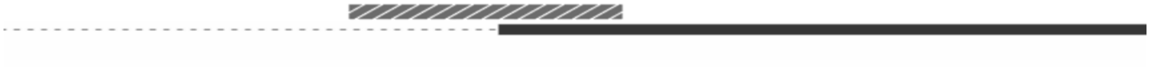

→ **does not meet criteria:** lies on exon!

B

```
# Aligned_sequences: 2
# 1: sv_PAX2
# 2: EGACTEQ4350035697-RA_antisense
# Matrix: EDNAFULL
# Gap_penalty: 16
# Extend_penalty: 4
#
# Length: 179
# Identity:      98/179 (54.7%)
# Similarity:    98/179 (54.7%)
# Gaps:          10/179 ( 5.6%)
# Score: 94
#
#
#=======================================

sv_PAX2          171 AAATCCAAATAAAATATTGACACTTTTTTGGGGAGGGGGAGGTGGGAAGC    220
                     ||||.|....|.|.|.|..|.|.|||..|.|.|..|..|||.|||.|| |
EGACTEQ4350035   396 AAATGCTGTCACATTGTGAAAAATTTAATAGTGCAGCAGAGATGGAAA-C    444

sv_PAX2          221 AAAGGTATTTAAAAT--AGGTTTTTGTTGCAATGCCCCCAGGATAAAGAA    268
                     |..|..|||.|||||  ||.||.|||.....||...|.||...||.|.||
EGACTEQ4350035   445 ATTGACATTGAAAATTGAGTTTCTTGGACACATTGTCACATTGTACAAAA    494

sv_PAX2          269 TGGAAGGAGACTGAAGACAAGTTGGAGTTTATAAAATCAATGGAATTTAT    318
                     |..||   .|.||.||...|.||  ||.|.|.|.|.|.|.|....|||.|
EGACTEQ4350035   495 TTTAA---TAGTGCAGCATAATT--AGATGAAACACTGATTTTTTTTTTT    539

sv_PAX2          319 TGCA-CAGCTACT-TGGCATTTAATAATT    345
                     ||.| |||.|||. |.||||..||||.||
EGACTEQ4350035   540 TGGAGCAGTTACCATTGCATACAATAGTT    568
```

→ below the threshold criteria

8. Actinia tenebrosa

Gene: **LOC116291861**
Transcript: XM_031699081.1
Product: XP_031554941.1

A

```
# Aligned_sequences: 2
# 1: sv_PAX2
# 2: XM_031699081.1_sense
# Matrix: EDNAFULL
# Gap_penalty: 16
# Extend_penalty: 4
#
# Length: 99
# Identity:      60/99 (60.6%)
# Similarity:    60/99 (60.6%)
# Gaps:          11/99 (11.1%)
# Score: 96
#
#
#=======================================

sv_PAX2          101 AATAAAGTCCCAGAATTTATGTGACTCGCA-CGAGTCAGGAGGTCAAACA    149
                     |.||||  |.||.|..||.||||....||| |||......||||.|.|.|
XM_031699081.1   745 AGTAAA--CGCAAACATTCTGTGGAAAGCAACGACAGTACAGGTAAGAAA    792

sv_PAX2          150 ACTGTTATGGAGCGAAGTTAAAAATCCAAATAAAATATTGACACTTTTT    198
                     |||.||      |.|..||||.||  |.||.||||.|||||||.|||||
XM_031699081.1   793 ACTTTT------CAACCTTAACAA--CGAAGAAAAAAATTGACAGTTTTT    833
```

→ **Hit:**
>XM_031699081.1_sense_CRE-like
AGTAAACGCAAACATTCTGTGGAAAGCAACGACAGTACAGGTAAGAAAACTTTTCAACCTTAACAACGAAGAAAAAAATTGACAGTTTTT

→ **BLASTN SEARCH** for XM_031699081.1_sense_CRE-like: e-value 9.4e-44

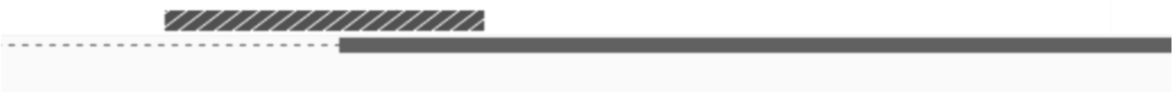

→ **does not meet criteria:** lies on exon!

B

```
# Aligned_sequences: 2
# 1: sv_PAX2
# 2: XM_031699081.1_antisense
# Matrix: EDNAFULL
# Gap_penalty: 16
# Extend_penalty: 4
#
# Length: 61
# Identity:      40/61 (65.6%)
# Similarity:    40/61 (65.6%)
# Gaps:           2/61 ( 3.3%)
# Score: 92
#
#
#=======================================

sv_PAX2          287 AAGTTGGAGTTTATAAAATCAATGGAATTTATTGC-ACAGCTACTTGGCA    335
                     ||||||||.||||. ||||||.||..|..||.||.| |.|||..||||.||
XM_031699081.   1762 AAGTTGGAATTTC-AAAATCCATAAATGTTGTTTCNATAGCAGCTTGTCA   1810

sv_PAX2          336 TTTAATAATTC    346
                     ...|..|.|||
XM_031699081.   1811 AGCACAAGTTC   1821
```

→ **Hit:**
>XM_031699081.1_antisense_CRE-like
AAGTTGGAATTTCAAAATCCATAAATGTTGTTTCNATAGCAGCTTGTCAAGCACAAGTTC

→ **BLASTN SEARCH** for XM_031699081.1_antisense_CRE-like: e-value 7E-25

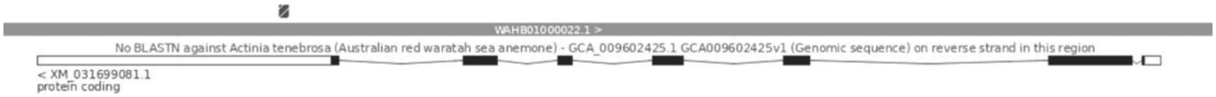

→ **does not meet criteria:** lies on UTR!

9. Exaiptasia diaphana

Gene: **LOC110248033**
Transcript: XM_021054519.2
Product: XP_020910178.1

A

```
# Aligned_sequences: 2
# 1: sv_PAX2
# 2: XM_021054519.2_sense
# Matrix: EDNAFULL
# Gap_penalty: 16
# Extend_penalty: 4
#
# Length: 125
# Identity:      73/125 (58.4%)
# Similarity:    73/125 (58.4%)
# Gaps:           8/125 ( 6.4%)
# Score: 97
#
#
#=======================================

sv_PAX2          226 TATTTAAAATAGGTTTTTGTTGCAATGCCCCCAGGATAAAGAATGGAAG-    274
                     ||||||..||.. |||||.|....||..|.|.|.|.|..|.||||||||
XM_021054519.   2534 TATTTACGATTC-TTTTTCTAAACATTACGCAATGTTTTACAATGGAAGC   2582

sv_PAX2          275 GAGACTGAAGACAAG--TTGGAGTTTATAAAATCAATG--GAATTTATTG    320
                     ||.||...|.||||.  ||.||||||..|..|..||||  ||.||...||
XM_021054519.   2583 GAAACCACACACAAACCTTTGAGTTTCGAGTAGGAATGCTGATTTGTCTG   2632

sv_PAX2          321 CACAGCTACTTGGCATTTAATAATT    345
                     .|||  ||.||...|.|.||.||.|
XM_021054519.   2633 AACA--TAGTTAAGAGTCAAAAAAT   2655
```

→ below the threshold criteria

B

```
# Aligned_sequences: 2
# 1: sv_PAX2
# 2: XM_021054519.2_antisense
# Matrix: EDNAFULL
# Gap_penalty: 16
# Extend_penalty: 4
#
# Length: 52
# Identity:      34/52 (65.4%)
# Similarity:    34/52 (65.4%)
# Gaps:           4/52 ( 7.7%)
# Score: 86
#
#
#=======================================

sv_PAX2          296 TTTATAAAATCAATGGAATTTATTGCACAGCTACTTGGCATTTAATAATT    345
                     ||||.|||||.||||.|||||    |.||...|.||||.||..||..|.|
XM_021054519.   6947 TTTACAAAATTAATGCAATTT----CTCATTGAATTGGTATAAAACCACT   6992

sv_PAX2          346 CT    347
                     ||
XM_021054519.   6993 CT   6994
```

→ below the threshold criteria

10. Nematostella vectensis

Gene: **NEMVEDRAFT_v1g239535**
Transcript: EDO47088
Product: EDO47088

A

```
# Aligned_sequences: 2
# 1: EDO47088_sense
# 2: sv/PAX2
# Matrix: EDNAFULL
# Gap_penalty: 16
# Extend_penalty: 4
#
# Length: 60
# Identity:      39/60 (65.0%)
# Similarity:    39/60 (65.0%)
# Gaps:           1/60 ( 1.7%)
# Score: 99
#
#
#=======================================

EDO47088_sens   5760 ATAAGATGGTGTTTATTAACACTTCATTATTTTTTGAAAATCTGACTAGA   5809
                     |.|||.|||.||||||.||..|......||||.|||.|.|.|| |||.|.
sv/PAX2          285 ACAAGTTGGAGTTTATAAAATCAATGGAATTTATTGCACAGCT-ACTTGG    333

EDO47088_sens   5810 CATTTAGTCA   5819
                     ||||||.|.|
sv/PAX2          334 CATTTAATAA    343
```

→ **Hit:**
>EDO47088_sense_CRE-like
ATAAGATGGTGTTTATTAACACTTCATTATTTTTTGAAAATCTGACTAGACATTTAGTCA

→ **BLASTN SEARCH** for EDO47088_sense_CRE-like: e-value 1.7e-26

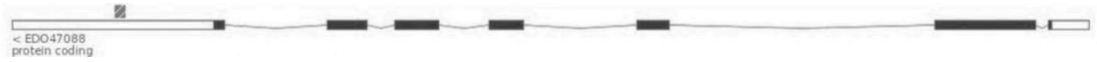

→ **does not meet criteria:** lies on UTR!

B

```
# Aligned_sequences: 2
# 1: EDO47088_antisense
# 2: sv/PAX2
# Matrix: EDNAFULL
# Gap_penalty: 16
# Extend_penalty: 4
#
# Length: 147
# Identity:      85/147 (57.8%)
# Similarity:    85/147 (57.8%)
# Gaps:          14/147 ( 9.5%)
# Score: 117
#
#
#=======================================

EDO47088_anti    564 ATGCAAGATTATTACAAGAAAATTTATGTCACAATCGCATCAGAATGTGG    613
                     |.||||...||||..||..|..|||.|||..||||..|..|||.||...|
sv/PAX2          217 AAGCAAAGGTATTTAAAATAGGTTTTTGTTGCAATGCCCCCAGGATAAAG    266

EDO47088_anti    614 GTGAATGAAAATATATTTGACTAAATGTCTAGTCAGATTTTCAAAAAATA    663
                        ||||.||..|     ||||.|| |.|.|||..||.|||..||||..|
sv/PAX2          267 ---AATGGAAGGA-----GACTGAA-GACAAGTTGGAGTTTATAAAATCA    307

EDO47088_anti    664 ATGAAGTGTTAATAAACACCATCTTA----TTGATAAATATTTAATT    706
                     |||.|.| |||.|..|||.|..|||.    ||.||||.|.|.|.|||
sv/PAX2          308 ATGGAAT-TTATTGCACAGCTACTTGGCATTTAATAATTCTCTCATT    353
```

→ below the threshold criteria

11. Acropora millepora

Gene: **LOC114976020**
Transcript: XM_029356360.2
Product: XP_029212193.2

A

```
# Aligned_sequences: 2
# 1: sv_PAX2
# 2: XM_029356360.2_sense
# Matrix: EDNAFULL
# Gap_penalty: 16
# Extend_penalty: 4
#
# Length: 83
# Identity:      48/83 (57.8%)
# Similarity:    48/83 (57.8%)
# Gaps:           1/83 ( 1.2%)
# Score: 88
#
#
#=======================================

sv_PAX2          222 AAGGTATTTAAAATAGGTTTTTGTTGCAATGCCCCCAGGATAAAGAATGG    271
                     |||.|.|....||||||.|||||.||...|....|.|..||.|| |.|||
XM_029356360.   3282 AAGTTTTGATGAATAGGCTTTTGCTGGCTTCATTCTACCATGAA-ATTGG   3330

sv_PAX2          272 AAGGAGACTGAAGACAAGTTGGAGTTTATAAAA    304
                     .||.||..|||..|.|..||...|||.|.||||
XM_029356360.   3331 CAGCAGTGTGACAATAGATTTCTGTTCAAAAAA   3363
```

→ below the threshold criteria

B

```
# Aligned_sequences: 2
# 1: sv_PAX2
# 2: XM_029356360.2_antisense
# Matrix: EDNAFULL
# Gap_penalty: 16
# Extend_penalty: 4
#
# Length: 50
# Identity:      34/50 (68.0%)
# Similarity:    34/50 (68.0%)
# Gaps:           2/50 ( 4.0%)
# Score: 94
#
#
#=======================================

sv_PAX2          140 AGGTCAAACAACTGTTATGGAGCG--AAGTTAAAAATCCAAATAAAATAT    187
                     ||||.|||.|..|...|.||||.|  ||||||.||||.||||.|||.|.|
XM_029356360.   4872 AGGTGAAATATATTAGAAGGAGAGGGAAGTTACAAATGCAAACAAATTCT   4921
```

→ **below the threshold criteria**

12. Orbicella faveolata

Gene: **LOC110042216**
Transcript: XM_020747577.1
Product: XP_020603236.1

A

```
# Aligned_sequences: 2
# 1: sv_PAX2
# 2: XM_020747577.1_sense
# Matrix: EDNAFULL
# Gap_penalty: 16
# Extend_penalty: 4
#
# Length: 69
# Identity:      43/69 (62.3%)
# Similarity:    43/69 (62.3%)
# Gaps:           3/69 ( 4.3%)
# Score: 87
#
#
#=======================================

sv_PAX2           11 TAATATTTCTTCAGGGACTTTCCAGCAACAGCACAAGTCA--TTAATTCA     58
                     |||.||.|||..|.|.|||.|.|||...|||.|.||...|  ||.||.||
XM_020747577   12606 TAAAATGTCTGAATGTACTGTTCAGTGTCAGTAAAACAAAAATTTATGCA  12655

sv_PAX2           59 CCCTCCCAAATTG-CTTAT     76
                     |..|||||...|| |||||
XM_020747577   12656 CAGTCCCAGTCTGGCTTAT  12674
```

→ **Hit:**

```
>XM_020747577.1_sense_CRE-like
TAAAATGTCTGAATGTACTGTTCAGTGTCAGTAAAACAAAAATTTATGCACAGTCCCAGTCTGGCTTAT
```

→ **BLASTN SEARCH** for XM_020747577.1_sense_CRE-like: e-value 1.2e-31

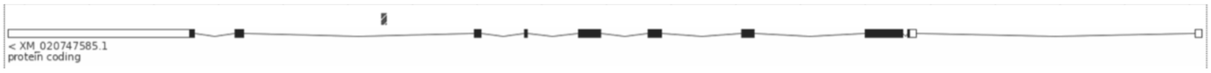

B

```
# Aligned_sequences: 2
# 1: sv_PAX2
# 2: XM_020747577.1_antisense
# Matrix: EDNAFULL
# Gap_penalty: 16
# Extend_penalty: 4
#
# Length: 136
# Identity:      81/136 (59.6%)
# Similarity:    81/136 (59.6%)
# Gaps:          12/136 ( 8.8%)
# Score: 101
#
#
#=======================================
sv_PAX2            70 TGCTTATTCAATAGC-AGGAACATGGCTCCTGAATAAA-GTCCCAGAATT    117
                      ||.|||.||..||.. ||||||   .||||.|.|.||| ||.|..|   |
XM_020747577.   1438 TGTTTACTCCGTACTGAGGAAC---TCTCCAGTAGAAACGTGCTGG---T   1481

sv_PAX2           118 TATGTGACTCGCACG-AGTC-AGGAGGTCAAAC--AACTGTTATGGAGCG    163
                      |||||.|.||.|.|. ||.| |.|||..||||.  |||..||.||.|..|
XM_020747577.   1482 TATGTAATTCACGCATAGCCTACGAGCGCAAAACAAACGATTGTGCACGG   1531

sv_PAX2           164 AAGTTAAAAATCCAAATAAAATATTGACACTTTTTT    199
                      ..||.||...|||||...||||.|||.||..|||.|
XM_020747577.   1532 TCGTGAACCTTCCAACATAAATCTTGGCATGTTTGT   1567
```

→ below the threshold criteria

13. Pocillopora damicornis

Gene: **LOC113664562**
Transcript: XM_027180177.1
Product: XP_027035978.1

A

```
# Aligned_sequences: 2
# 1: sv_PAX2
# 2: XM_027180177.1_sense
# Matrix: EDNAFULL
# Gap_penalty: 16
# Extend_penalty: 4
#
# Length: 94
# Identity:      57/94 (60.6%)
# Similarity:    57/94 (60.6%)
# Gaps:           6/94 ( 6.4%)
# Score: 101
#
#
#=======================================

sv_PAX2          216 GAAGCAAAGGTATTTAAAATAGGTTTTTGTTGCAATGCCCCCAGGATAAA    265
                     |||||..||..|...|..|.|.|||||||.||||.|||.||.||.| |..
XM_027180177.    810 GAAGCTGAGACAGCCACGAGAAGTTTTTGATGCACTGCTCCAAGAA-ACC    858

sv_PAX2          266 GAATGGAAGGAGACTGAAGACAAGTTGGAGTTTATA-AAATCAA    308
                     .|.|.|...||..||.|||..|||    ||.|||.| |||||||
XM_027180177.    859 CAGTCGCTAGAATCTCAAGGGAAG----AGATTAGATAAATCAA    898
```

→ **Hit:**

```
>XM_027180177.1_sense_CRE-like
GAAGCTGAGACAGCCACGAGAAGTTTTTGATGCACTGCTCCAAGAA-ACCCAGTCGCTAGAATCTCAAGGGAAGAGATTAGATAAATCAA
```

→ **BLASTN SEARCH** for XM_027180177.1_sense_CRE-like: e-value 9.3e-44

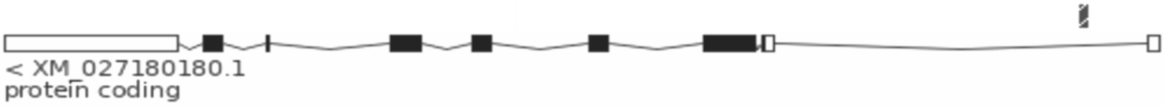

B

```
# Aligned_sequences: 2
# 1: sv_PAX2
# 2: XM_027180177.1_antisense
# Matrix: EBLOSUM62
# Gap_penalty: 14
# Extend_penalty: 4
#
# Length: 367
# Identity:     182/367 (49.6%)
# Similarity:   182/367 (49.6%)
# Gaps:          31/367 ( 8.4%)
# Score: 645
#
#
#=======================================

sv_PAX2            1 CAAGCAATTTTAATATTTCTTCAGGGACTTTCCAG----CAACAGCACAA     46
                     |...|...|..|.||.|||. |.|||..||||.||    ..||....||.
XM_027180177.   9155 CTTCCCGGTAAATTACTTCC-CGGGGGATTTCAAGGTTAAGACGAAGCAC   9203

sv_PAX2           47 GTCATTAATTCACCCTCCCAAATTGCTTAT---TCAATAGCAGGAACATG     93
                     ...|||...||..|.||.|||.||.|....   ||..||....|||.||.
XM_027180177.   9204 CGAATTTGGTCTTCTTCACAATTTTCGAGAATGTCGTTAAGCCGAAAATA   9253

sv_PAX2           94 GCTCCTGAATAAAGTCCCAGAATTTATGTGACTCGCACGAGTCAGGAGGT    143
                     . |.|.||.|.|. |.|.|.....|.|.||...||.||||...||.|.|.
XM_027180177.   9254 A-TGCAGAGTTAT-TTCAAACTGATCTTTGCTACGAACGAAATAGCATGC   9301

sv_PAX2          144 CAAACAACTGTTATGGAGCGAAGTTAAAAATCCAAATAAAATATTGACAC    193
                     ..|....||||| ||.||....||.||.|.|.....|||.|.|.||.||.
XM_027180177.   9302 GTATTTCCTGTT-TGTAGATGCGTCAAGATTGTTCGTAATACACTGGCAT   9350

sv_PAX2          194 TTTTTTGGGGAGGGGGAGGTGGGAAGCAAAGGTATTTAA-AATAGG--TT    240
                     ..||.   ||..|.|.||.||.    ||..|.|.|||.| ||||||  .|
XM_027180177.   9351 AATTA---GGTTGCGCAGTTGT----CATTGATTTTTACAATAGGGCCT   9393

sv_PAX2          241 TTTGTTGCAATGCCCCCAGGATAAAGAATGGAAGGAGACTGAAGACAAGT    290
                     |||.||.|..||.    ||||....|.||.|| |||||   |||||.|||
XM_027180177.   9394 TTTCTTACTGTGA----AGGAATCCGCATCGA-GGAGA---AAGACGAGT   9435

sv_PAX2          291 TGGAGTTTATAAAATCAA--TGGAATTTATTGCACAGCTACTTGGCATTT    338
                     .||.|||.|....|.|||  ||..|.|.|||....|||||.....|....
XM_027180177.   9436 AGGTGTTAAATGCAGCAAAGTGTGACTGATTTTTAGCTATAGACCTGAA   9485

sv_PAX2          339 AATAATTCTCTCATTTC    355
                     |.|.|.|||..|.|.||
XM_027180177.   9486 ATTCACTCTACCGTATC   9502
```

→ below the threshold criteria

14. Stylophora pistillata

Gene: **LOC111343163**
Transcript: XM_022950325.1
Product: XP_022806060.1

A

```
# Aligned_sequences: 2
# 1: sv_PAX2
# 2: XM_022950325.1_sense
# Matrix: EDNAFULL
# Gap_penalty: 16
# Extend_penalty: 4
#
# Length: 92
# Identity:      51/92 (55.4%)
# Similarity:    51/92 (55.4%)
# Gaps:           1/92 ( 1.1%)
# Score: 79
#
#
#=======================================

sv_PAX2          229 TTAAAATAGGTTTTTGTTGCAATGCCCCCAGGATAAAGAATGGAAGGAGA    278
                     |.||.||..|..|.||||..|.|.||.|.|||.||..||.||.. |.|||
XM_022950325   12210 TAAATATTTGCATCTGTTAGATTACCACAAGGTTATGGACTGCT-GTAGA  12258

sv_PAX2          279 CTGAAGACAAGTTGGAGTTTATAAAATCAATGGAATTTATTG    320
                     .||||...|.|...||.|..|.||..|||.||.|...|.|||
XM_022950325   12259 ATGAATGAATGAATGAATGAAGAATTTCATTGTACCCTTTTG  12300
```

→ **below the threshold criteria**

B

```
# Aligned_sequences: 2
# 1: sv_PAX2
# 2: XM_022950325.1_antisense
# Matrix: EDNAFULL
# Gap_penalty: 16
# Extend_penalty: 4
#
# Length: 120
# Identity:      72/120 (60.0%)
# Similarity:    72/120 (60.0%)
# Gaps:          14/120 (11.7%)
# Score: 96
#
#
#=======================================

sv_PAX2          187 TTGACACTTTTTTGGGGAGGGGGAGGTGG---GAAGCAAAGGTATTTAAA    233
                     |||||| ||.||...||||..|||||.|.   |||.||||||||..||.|
XM_022950325.   1969 TTGACA-TTCTTGCAGGAGCTGGAGGAGTCCTGAAACAAAGGTAAATACA   2017

sv_PAX2          234 ATAGGTTTTTGTTGCAATGCCCCCAGGATAAAGAATGGAAGGAGACTGAA    283
                     | ||.|..||..      |.||..|....|.|.|||.|.||.|.||| ||
XM_022950325.   2018 A-AGATAGTTTA------GACCTTAAACAACACAATTGTAGTATACT-AA   2059

sv_PAX2          284 GACAAGTTGGAGTTTATAAA    303
                     |..||.  |||.||||||||
XM_022950325.   2060 GTTAAA--GGAATTTATAAA   2077
```

→ **Hit:**
>XM_022950325.1_antisense_CRE-like
TTGACATTCTTGCAGGAGCTGGAGGAGTCCTGAAACAAAGGTAAATACAAAGATAGTTTAGACCTTAAACAACACAATTGTAGTATACTAAGTTAAA
GGAATTTATAAA

→ **BLASTN SEARCH** for XM_022950325.1_antisense_CRE-like: e-value 2.3e-55

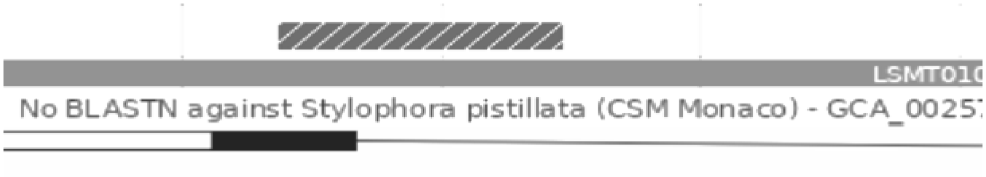

→ **does not meet criteria:** lies on exon!

S1.2. *dachshund/DACH1*

1. Mnemiopsis leidyi

No *dachshund/DACH1* homolog identified.

2. Amphimedon queenslandica

No *dachshund/DACH1* homolog identified.

3. Thelohanellus kitauei

No *dachshund/DACH1* homolog identified.

4. Hydra vulgaris

No *dachshund/DACH1* homolog identified.

5. Clytia hemisphaerica

No *dachshund/DACH1* homolog identified.

6. Dendronephthya gigantea

Gene: **LOC114528507**
Transcript: XM_028550166.1
Product: XP_028405967.1

A

```
# Aligned_sequences: 2
# 1: dac_DACH1
# 2: XM_028550166.1_sense
# Matrix: EDNAFULL
# Gap_penalty: 16
# Extend_penalty: 4
#
# Length: 73
# Identity:      45/73 (61.6%)
# Similarity:    45/73 (61.6%)
# Gaps:           2/73 ( 2.7%)
# Score: 89
#
#
#=======================================

dac_DACH1         80 TCTAAAAACTCTCAAGTGTATCGATACGATGCGTTTCTTTTTTTCCTTCG    129
                     |.|.||||||.||||.| |||...||..|..|.|...||||||.|.|||.
XM_028550166.   3588 TGTGAAAACTTTCAAAT-TATTCTTAGCAATCATGAATTTTTTCCTTTCC   3636

dac_DACH1        130 TTAA-ATAAATAATAACCAAAAA    151
                     ||.| |||.|.|.|..|.|||.|
XM_028550166.   3637 TTTATATACAAAGTTTCAAAATA   3659
```

→ **Hit:**

```
>XM_028550166.1_sense_CRE-like
TGTGAAAACTTTCAAATTATTCTTAGCAATCATGAATTTTTTCCTTTCCTTTATATACAAAGTTTCAAAATA
```

→ **BLASTN SEARCH** for XM_028550166.1_sense_CRE-like: e-value 1.2e-33

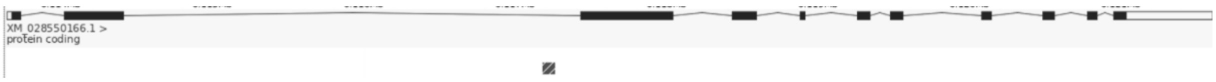

B

```
# Aligned_sequences: 2
# 1: dac_DACH1
# 2: XM_028550166.1_antisense
# Matrix: EDNAFULL
# Gap_penalty: 16
# Extend_penalty: 4
#
# Length: 110
# Identity:      66/110 (60.0%)
# Similarity:    66/110 (60.0%)
# Gaps:           9/110 ( 8.2%)
# Score: 118
#
#
#=======================================

dac_DACH1         70 TTCTTTTCGCTCTAAAAACTCTCAAGTGTATCGATACG-----ATGCGTT    114
                     |.||||.|..|.||||.|||.|||||...|.|||||.|     |||||.|
XM_028550166.   7639 TGCTTTACAATATAAATACTTTCAAGACAAACGATATGTGCACATGCGCT   7688

dac_DACH1        115 TCTT---TTTTTCCTTCGTTAAATAAATA-ATAACCAAAAAAAAAAAAAA    160
                     |.||   ||.||||||..|.|||..|.|. ||.|..|||.|.|..|||..
XM_028550166.   7689 TTTTAAATTATTCCTTATTGAAAGCATTTCATTATTAAACAGATTAAATG   7738

dac_DACH1        161 CCAAAAAGTA    170
                     |.|..|.|||
XM_028550166.   7739 CAATTATGTA   7748
```

→ **Hit:**

>XM_028550166.1_antisense_CRE-like
TGCTTTACAATATAAATACTTTCAAGACAAACGATATGTGCACATGCGCTTTTTAAATTATTCCTTATTGAAAGCATTTCATTATTAAACAGATTAA
ATGCAATTATGTA

→ **BLASTN SEARCH** for XM_028550166.1_antisense_CRE-like: e-value 4.3e-56

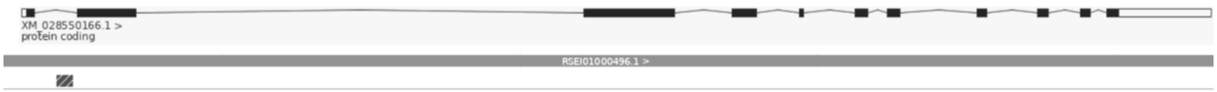

7. Actinia equina

Gene: EGACTEQ4350014069
Transcript: EGACTEQ4350014069-RA
Product: EGACTEQ4350014069-PA

A

```
# Aligned_sequences: 2
# 1: dac_DACH1
# 2: EGACTEQ4350014069-RA_sense
# Matrix: EDNAFULL
# Gap_penalty: 16
# Extend_penalty: 4
#
# Length: 117
# Identity:      69/117 (59.0%)
# Similarity:    69/117 (59.0%)
# Gaps:           5/117 ( 4.3%)
# Score: 117
#
#
#=======================================

dac_DACH1        112 GTTTCTTTTTTTCCTTCGTTAAATAAA---TAATAACCAAAAAAAAAAAA    158
                     ||||.|||||||..|.|..|..|||||   ||||.|...|..|||.||||
EGACTEQ4350014   929 GTTTTTTTTTTTTTTTGCCCTTGATAAAGTTTAATTAAGTATGAAATAAAA    978

dac_DACH1        159 AACCAAAAAGTAGGAG-GAGAAAAGTTATTGCCATAGTTTTTTTATTATA    207
                     |..|||.|.|.|..|. |..|..|.||.|||...||.|.|.||||||.||
EGACTEQ4350014   979 ATTCAATATGAATAAAAGCTATTACTTTTTGGATTACTGTATTTATTTTA   1028

dac_DACH1        208 CTTGTGTGTTTACCTTT    224
                     ...|..| |||.|.|||
EGACTEQ435001   1029 AAAGATT-TTTGCATTT   1044
```

→ below the threshold criteria

B

```
# Aligned_sequences: 2
# 1: dac_DACH1
# 2: EGACTEQ4350014069-RA_antisense
# Matrix: EDNAFULL
# Gap_penalty: 16
# Extend_penalty: 4
#
# Length: 125
# Identity:      74/125 (59.2%)
# Similarity:    74/125 (59.2%)
# Gaps:           7/125 ( 5.6%)
# Score: 106
#
#
#=======================================
dac_DACH1         42 TTCGAGTTTAATTAATTTAATAAACAAATTCTTTTCGCTCTAAAAACTCT     91
                     |||.|.|..||..|..||...|||.||||.|..|.  .||.|||||.|..
EGACTEQ435001   4607 TTCAAATGCAAAAATCTTTTAAAATAAATACAGTA--ATCCAAAAAGTAA   4654

dac_DACH1         92 CAAGTGT-ATCGATACGATGCGTTTCTTTTTT-TCCTTCGTTAAA-TAAA    138
                     .|..|.| ||..|||  .||..|||.|.|||. |.|||..||||| |..|
EGACTEQ435001   4655 TAGCTTTTATTCATA--TTGAATTTTTATTTCATACTTAATTAAACTTTA   4702

dac_DACH1        139 TAATAACCAAAAAAAAAAAAAAAACCA    163
                     |.|....||||||||||||||||.|
EGACTEQ435001   4703 TCAAGGGCAAAAAAAAAAAAAAAACAA   4727
```

→ below the threshold criteria

8. *Actinia tenebrosa*

Gene: **LOC116304329**
Transcript: XM_031714048.1
Product: XP_031569908.1

A

```
# Aligned_sequences: 2
# 1: dac_DACH1
# 2: XM_031714048.1_sense
# Matrix: EDNAFULL
# Gap_penalty: 16
# Extend_penalty: 4
#
# Length: 99
# Identity:      58/99 (58.6%)
# Similarity:    58/99 (58.6%)
# Gaps:           9/99 ( 9.1%)
# Score: 94
#
#
#=======================================
dac_DACH1        130 TTAAATAAATAA--TAACCAAAAAAAAAAAAAACCAAAAAGTAGGAGGAG    177
                     |.||||||||.  |||.|.....||||.|||.|.|||||.||.
XM_031714048.   4888 TAAAATAAATACGGTAATCCNTTCAAAATAAACCAAAAAATTAT------   4931

dac_DACH1        178 AAAAGTTATTGCCATAGTTTTTTTATTATACTTGTGTGTTTACCTTTCT    226
                     |....|||||...||.|..|||||||||..| |.||...||.|.||||.|
XM_031714048.   4932 AGCTTTTATTCATATTGAATTTTTATTTCA-TNGTTAATTAAACTTTAT   4979
```

→ below the threshold criteria

B

```
# Aligned_sequences: 2
# 1: dac_DACH1
# 2: XM_031714048.1_antisense
# Matrix: EDNAFULL
# Gap_penalty: 16
# Extend_penalty: 4
#
# Length: 91
# Identity:      55/91 (60.4%)
# Similarity:    55/91 (60.4%)
# Gaps:           2/91 ( 2.2%)
# Score: 109
#
#
#=======================================
dac_DACH1        108 ATGCGTTTCTTTTTTTCCTTCGTTAAATAAATAATAACCAAAAAAAAAAA    157
                     |.|.|||..||||||.||.|.|.||||... ||||.|.|.|..|||.|||
XM_031714048.   1003 AAGTGTTGTTTTTTTGCCCTTGATAAAGTT-TAATTAACNATGAAATAAA   1051

dac_DACH1        158 AAACCAAAAAGTAGGAG-GAGAAAAGTTATTGCCATAGTTT    197
                     ||..|||.|.|.|..|. |..|.||.||.|||...||.|||
XM_031714048.   1052 AATTCAATATGAATAAAAGCTATAATTTTTTGGTTTATTTT   1092
```

→ Hit:
>XM_031714048.1_antisense_CRE-like
AAGTGTTGTTTTTTTGCCCTTGATAAAGTTTAATTAACNATGAAATAAAAATTCAATATGAATAAAAGCTATAATTTTTTGGTTTATTTT

→ BLASTN SEARCH for XM_031714048.1_antisense_CRE-like: e-value 1.5e-42

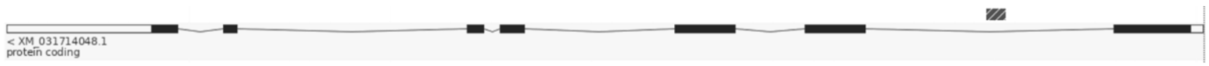

9. Exaiptasia diaphana

Gene: **LOC110254792**
Transcript: XM_021061836.1
Product: XP_020917495.1

A

```
# Aligned_sequences: 2
# 1: dac_DACH1
# 2: XM_021061836.1_sense
# Matrix: EDNAFULL
# Gap_penalty: 16
# Extend_penalty: 4
#
# Length: 101
# Identity:      60/101 (59.4%)
# Similarity:    60/101 (59.4%)
# Gaps:           7/101 ( 6.9%)
# Score: 100
#
#
#=======================================
dac_DACH1        136 AAATAATAACC--AAAAAAAAAAAAAACCAAAAAGTAGGAGGAGAAAAGT    183
                     ||||||...||  |.||.|||.||.|.|||||..|||...|.|    |..
XM_021061836.   4060 AAATAAAGTCCTTATAATAAAGAAGAGCCAAATTGTACTCGTA----ACC   4105

dac_DACH1        184 TATTGCCATAGTTTTTTTATTATACTTGTGTGTTTACCTTTCTGGTGGCT    233
                     |||..|.||| ||||||.|...|||.|.|.|..||..|.|||.||||..|
XM_021061836.   4106 TATATCAATA-TTTTTTCAGGCTACATATCTTCTTCTCATTCCGGTGTTT   4154

dac_DACH1        234 T    234
                     |
XM_021061836.   4155 T   4155
```

→ below the threshold criteria

B

```
# Aligned_sequences: 2
# 1: dac_DACH1
# 2: XM_021061836.1_antisense
# Matrix: EDNAFULL
# Gap_penalty: 16
# Extend_penalty: 4
#
# Length: 86
# Identity:      53/86 (61.6%)
# Similarity:    53/86 (61.6%)
# Gaps:           4/86 ( 4.7%)
# Score: 85
#
#
#=======================================

dac_DACH1         31 CCAATTTCCGTTTCG-AGTTTAATTAATTTAATAAACAAATTCTTTTCGC     79
                     |.||.|||..|.||| |..|||.|||..|.|.||||.|||.|||||.| |
XM_021061836.   1151 CTAAGTTCTTTATCGTACATTAGTTATGTAAGTAAATAAAATCTTTAC-C   1199

dac_DACH1         80 TCTAAAAACTCTCAAGTGTATCGATACGATGCGTTT    115
                     ..|.|....|.|||.| |.|||. |.|||||..|||
XM_021061836.   1200 ATTCATTTGTTTCATG-GCATCT-TGCGATGTATTT   1233
```

→ **Hit:**
>XM_021061836.1_antisense_CRE-like
CTAAGTTCTTTATCGTACATTAGTTATGTAAGTAAATAAAATCTTTACCATTCATTTGTTTCATGGCATCTTGCGATGTATTT

→ **BLASTN SEARCH** for XM_021061836.1_antisense_CRE-like: e-value 3.5e-40

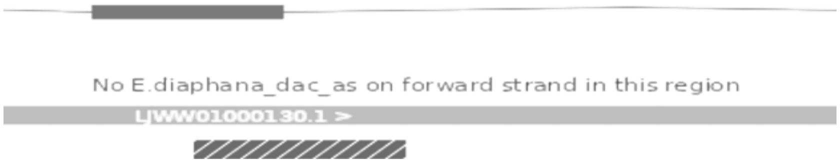

→ **does not meet criteria:** lies on exon!

10. *Nematostella vectensis*

10.a Gene NEMVEDRAFT_v1g207853 (EDO40467)

Gene: **NEMVEDRAFT_v1g207853**
Transcript: EDO40467
Product: EDO40467

A

```
# Aligned_sequences: 2
# 1: EDO40467_sense
# 2: dac/DACH1
# Matrix: EDNAFULL
# Gap_penalty: 16
# Extend_penalty: 4
#
# Length: 73
# Identity:      43/73 (58.9%)
# Similarity:    43/73 (58.9%)
# Gaps:           1/73 ( 1.4%)
# Score: 83
#
#
#=======================================

EDO40467_sens   1660 CATTATATTAAGAA-AACCTTTAAAAGAAAAAGTCGACAACAACGCAGAT   1708
                     |.|||.||.||.|| ||||...||||.|||||..|.|.||..|.|..||.
dac/DACH1        128 CGTTAAATAAATAATAACCAAAAAAAAAAAAAACCAAAAAGTAGGAGGAG    177

EDO40467_sens   1709 TTATGTTTTTTAGAACGATGTTT   1731
                     ..|.|||.||...|..|.|.|||
dac/DACH1        178 AAAAGTTATTGCCATAGTTTTTT    200
```

→ below the threshold criteria

B

```
# Aligned_sequences: 2
# 1: EDO40467_antisense
# 2: dac/DACH1
# Matrix: EDNAFULL
# Gap_penalty: 16
# Extend_penalty: 4
#
# Length: 37
# Identity:      26/37 (70.3%)
# Similarity:    26/37 (70.3%)
# Gaps:           0/37 ( 0.0%)
# Score: 86
#
#
#=======================================

EDO40467_anti   6655 TAACCCTTAATATAATTAACAAAAACGTAGGAAGAAA   6691
                     |||||...||.|.||..|||.||||.||||||.||.|
dac/DACH1        142 TAACCAAAAAAAAAAAAAACCAAAAAGTAGGAGGAGA    178
```

→ below the threshold criteria

10.b Gene [NEMVEDRAFT_v1g107148](#) (EDO40468)

Gene: **NEMVEDRAFT_v1g107148**
Transcript: [EDO40468](#)
Product: [EDO40468](#)

A

```
# Aligned_sequences: 2
# 1: EDO40468_sense
# 2: dac/DACH1
# Matrix: EDNAFULL
# Gap_penalty: 16
# Extend_penalty: 4
#
# Length: 41
# Identity:      24/41 (58.5%)
# Similarity:    24/41 (58.5%)
# Gaps:           0/41 ( 0.0%)
# Score: 52
#
#
#=======================================

EDO40468_sens    221 AATCGATGCAAGCTCATCTCCAAAGAAGACTTTAATAAAAT    261
                     |.||||.||..||.||..|.|......||.||||||.||.|
dac/DACH1         18 ATTCGAGGCTCGCCCAATTTCCGTTTCGAGTTTAATTAATT     58
```

→ below the threshold criteria

B

```
# Aligned_sequences: 2
# 1: EDO40468_antisense
# 2: dac/DACH1
# Matrix: EDNAFULL
# Gap_penalty: 16
# Extend_penalty: 4
#
# Length: 20
# Identity:      14/20 (70.0%)
# Similarity:    14/20 (70.0%)
# Gaps:           0/20 ( 0.0%)
# Score: 46
#
#
#=======================================

EDO40468_sens     15 ATTTTATTAAAGTCTTCTTT     34
                     ||||.||.||....||||||
dac/DACH1         56 ATTTAATAAACAAATTCTTT     75
```

→ below the threshold criteria

11. Acropora millepora

Gene: **LOC114963920**
Transcript: XM_029343194.2
Product: XP_029199027.1

A

```
# Aligned_sequences: 2
# 1: dac_DACH1
# 2: XM_029343194.2_sense
# Matrix: EDNAFULL
# Gap_penalty: 16
# Extend_penalty: 4
#
# Length: 97
# Identity:      59/97 (60.8%)
# Similarity:    59/97 (60.8%)
# Gaps:           5/97 ( 5.2%)
# Score: 107
#
#
#=======================================

dac_DACH1         49 TTAATTAATTTAATAAACAAATTCTT---TTCGCTCTAAAAACTCTCAAG     95
                     |||||.|.||||..||| ||||..|.   |||..|.|.||||||| |.|.
XM_029343194.   2962 TTAATCATTTTAGCAAA-AAATGGTCGGGTTCTATTTGAAAACTC-CTAC   3009

dac_DACH1         96 TGTATCGATACGATGCGTTTCTTTTTTTCCTTCGTTAAATAAATAAT    142
                     |.|.||.|...|.|.|.||||..|...|||.|.|||.|..||||.||
XM_029343194.   3010 TATGTCTAATTGCTTCCTTTCAATGAATCCATTGTTTATGAAATGAT   3056
```

→ **Hit:**
>XM_029343194.2_sense_CRE-like
TTAATCATTTTAGCAAAAAATGGTCGGGTTCTATTTGAAAACTCCTACTGTATCGATACGATGCGTTTCTTTTTTTCCTTCGTTAAATAAATAAT

→ **BLASTN SEARCH** for XM_029343194.2_sense_CRE-like: e-value 1.5e-19

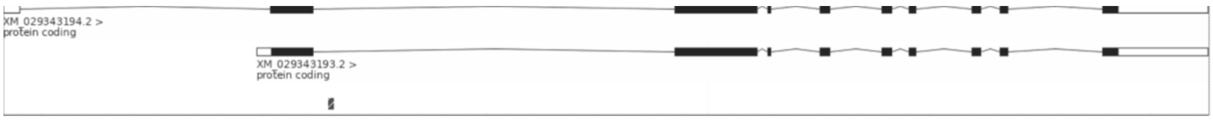

B

```
# Aligned_sequences: 2
# 1: dac_DACH1
# 2: XM_029343194.2_antisense
# Matrix: EDNAFULL
# Gap_penalty: 16
# Extend_penalty: 4
#
# Length: 55
# Identity:      38/55 (69.1%)
# Similarity:    38/55 (69.1%)
# Gaps:           2/55 ( 3.6%)
# Score: 110
#
#
#=======================================

dac_DACH1        113 TTTCTTTTTTTCCTTCGTTAAATAAATAATAACCAAAAAAAAAAAAAACC    162
                     ||||||...||.|||  ||..|..||||.|||.|...|||||||||||.|
XM_029343194.   2696 TTTCTTGAGTTTCTT--TTGGAGGAATACTAAACGGGAAAAAAAAAAAAC   2743

dac_DACH1        163 AAAAA    167
                     |||.|
XM_029343194.   2744 AAACA   2748
```

→ **Hit:**

>XM_029343194.2_antisense_CRE-like
TTTCTTGAGTTTCTTTTGGAGGAATACTAAACGGGAAAAAAAAAAAAACAAACA

→ **BLASTN SEARCH** for XM_029343194.2_antisense_CRE-like: e-value 2.9e-22

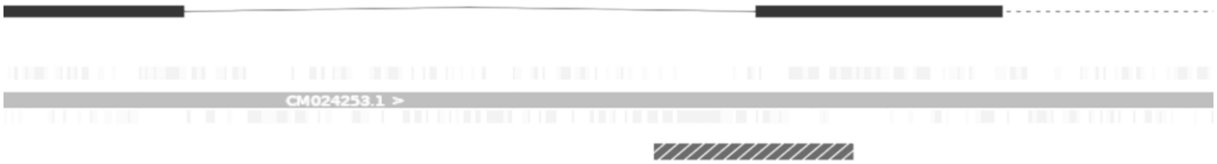

→ **does not meet criteria:** lies on exon!

12. Orbicella faveolata

Gene: **LOC110043426**
Transcript: XM_020748875.1
Product: XP_020604534.1

A

```
# Aligned_sequences: 2
# 1: dac_DACH1
# 2: XM_020748875.1_sense
# Matrix: EDNAFULL
# Gap_penalty: 16
# Extend_penalty: 4
#
# Length: 99
# Identity:      62/99 (62.6%)
# Similarity:    62/99 (62.6%)
# Gaps:          12/99 (12.1%)
# Score: 102
#
#
#=======================================
dac_DACH1         16 CCATTCGAGGCTCGCCCAATTTCCGTTTCGAGTTTAAT--TAATTTAATA     63
                     ||||||||   ||||  ||||..|.||.||.|.|.|||  |..|||.|.|
XM_020748875.   3006 CCATTCGA---TCGC--AATTATCTTTCCGTGATAAATATTGTTTTCAGA   3050

dac_DACH1         64 AACAAATTCTTTTC---GCTCTAAAAACTCTCAAGTGTATCGATACGAT    109
                     ||||  |||.|.||   |.|.||..||...|.||.|||||||.|.|.||
XM_020748875.   3051 AACA--TTCCTATCATTGGTATATCAAAATTTAATTGTATCGGTTCAAT   3097
```

→ Hit:

```
>XM_020748875.1_sense_CRE-like
CCATTCGATCGCAATTATCTTTCCGTGATAAATATTGTTTTCAGAAACATTCCTATCATTGGTATATCAAAATTTAATTGTATCGGTTCAAT
```

→ BLASTN SEARCH for XM_020748875.1_sense_CRE-like: e-value 3.2e-45

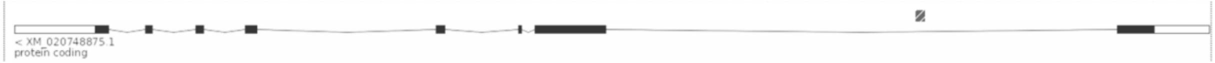

B

```
# Aligned_sequences: 2
# 1: dac_DACH1
# 2: XM_020748875.1_antisense
# Matrix: EDNAFULL
# Gap_penalty: 16
# Extend_penalty: 4
#
# Length: 114
# Identity:      65/114 (57.0%)
# Similarity:    65/114 (57.0%)
# Gaps:           4/114 ( 3.5%)
# Score: 93
#
#
#=======================================
dac_DACH1         54 TAATTTAATAAACAA-ATTCTTTTCGCTCTAAAAACTCTCAAGTGTATCG    102
                     |||.||||.|.||.. ||.|||||..||..|.|..||||..|.|..|||.
XM_020748875.   7726 TAAATTAAGACACTGCATACTTTTAACTTGACATTCTCTATATTTAATCT   7775

dac_DACH1        103 ATACGATGCGTTTCTTTTTTTCCTTCGTTAAATAAAT-AATAACCAAAAA    151
                     |.|..||.|.||.|...|..|.  |||..||.|.|.| ||||..|||||.
XM_020748875.   7776 AAAGAATTCATTACCAATGCTG--TCGGAAATTTAGTCAATATTCAAAAT   7823

dac_DACH1        152 AAAAAAAAACCAAA    165
                     |.|..|.|||.|.|
XM_020748875.   7824 ATACCAGAACGATA   7837
```

→ below the threshold criteria

13. Pocillopora damicornis

Gene: **LOC113686936**
Transcript: XM_027204620.1
Product: XP_027060421.1

A

```
# Aligned_sequences: 2
# 1: dac_DACH1
# 2: XM_027204620.1_sense
# Matrix: EDNAFULL
# Gap_penalty: 16
# Extend_penalty: 4
#
# Length: 187
# Identity:     104/187 (55.6%)
# Similarity:   104/187 (55.6%)
# Gaps:          17/187 ( 9.1%)
# Score: 104
#
#
#=======================================

dac_DACH1         53 TTAATTTAATAAACAAATTCTTTTC---GCTCTAAAAACTCTCAAGTGTA     99
                     ||.|||||..||..|.|..||||||   ||| ||....||.||.|||...
XM_027204620.   6645 TTGATTTAGCAACAACAGACTTTTCAATGCT-TATCTTCTGTCCAGTCGT   6693

dac_DACH1        100 TCGATACGATGCGTTTCTTTTTTTCCTTCGTTAAATAAATAATAACCAAA    149
                     ..|||.||  ||||.|.|.||...|....|.||||.|.|.|.|...|.||
XM_027204620.   6694 GGGATTCG--GCGTGTGTGTTCGCCACAGGATAAAAAGAAATTCGGCTAA   6741

dac_DACH1        150 AAAAAAAAAAACCAAAAAGTAGGAGGAGAAAAGTTATTGCCATA-GTTTT    198
                     |.|||.||...|    |.||  .|.||..|||||.    |.||| ||..|
XM_027204620.   6742 AGAAACAATCGC----ATGT--CATGAATAAAGTA----CAATATGTAAT   6781

dac_DACH1        199 TTTATTATACTTGTGTGTTTACCTTTCTGGTGGCTTG    235
                     |||||.|||..|.|...|..|.|||.||.||||||.|
XM_027204620.   6782 TTTATGATATCTTTCACTGCAGCTTCCTTGTGGCTAG   6818
```

→ below the threshold criteria

B

```
# Aligned_sequences: 2
# 1: dac_DACH1
# 2: XM_027204620.1_antisense
# Matrix: EDNAFULL
# Gap_penalty: 16
# Extend_penalty: 4
#
# Length: 43
# Identity:      33/43 (76.7%)
# Similarity:    33/43 (76.7%)
# Gaps:           0/43 ( 0.0%)
# Score: 125
#
#
#=======================================

dac_DACH1        125 CTTCGTTAAATAAATAATAACCAAAAAAAAAAAAAAACCAAAAA    167
                     ||.|..||||.|||.||.||..||||||||||||||..|||||
XM_027204620.   1295 CTCCACTAAAAAAAAAAAAAAAAAAAAAAAAAAAAAAAAAAAA   1337
```

→ below the threshold criteria

14. Stylophora pistillata

Gene: **LOC111336406**
Transcript: XM_022942501.1
Product: XP_022798236.1

A

```
# Aligned_sequences: 2
# 1: dac_DACH1
# 2: XM_022942501.1_sense
# Matrix: EDNAFULL
# Gap_penalty: 16
# Extend_penalty: 4
#
# Length: 222
# Identity:     121/222 (54.5%)
# Similarity:   121/222 (54.5%)
# Gaps:          12/222 ( 5.4%)
# Score: 117
#
#
#=======================================

dac_DACH1         27 TCGCCCAATTTCCGTTTCGAGTTTAATTAATTTAATAAACAAAT--TCTT     74
                     ||||....||..||.||.|..|||.||...||...|....||||  ||||
XM_022942501.   1159 TCGCATTTTTCACGGTTAGCCTTTGATACTTTGGTTTTTAAAATCATCTT   1208

dac_DACH1         75 TTC--GCTCTAAAAACT-CTCAAGTGTATCG--ATACGATGCGT---TTC    116
                     |||  |||.|.|||||| ||...||.|..||  ||...|..|.|   ||.
XM_022942501.   1209 TTCAGGCTATGAAAACTTCTTTTGTCTTCCGCCATCTCACTCATCAATTA   1258

dac_DACH1        117 TTTTTTT-CCTTCGTTAAATAAATAATAACCAAAAAAAAAAAA-AACCAA    164
                     ||||||| ||..|.||||..|||..|||..||....||||..| ||..||
XM_022942501.   1259 TTTTTTTGCCCCCCTTAAGCAAAAGATAGACACGGGAAAATCAGAAGGAA   1308

dac_DACH1        165 AAAGTAGGAGGAGAAAAGTTATTGCCATAGTTTTTTTATTATACTTGTGT    214
                     |||..|..||||...|....||.....||||.|...|.|.||.....|||
XM_022942501.   1309 AAAAAAAAAGGAACGATCCAATCCATCTAGTGTCAATCTCATGAAATTGT   1358

dac_DACH1        215 GTTTACCTTTCTGGTGGCTTGA    236
                     ||||...|..|.|||..|||.|
XM_022942501.   1359 GTTTTGGTGCCCGGTCTCTTAA   1380
```

→ below the threshold criteria

B

```
# Aligned_sequences: 2
# 1: dac_DACH1
# 2: XM_022942501.1_antisense
# Matrix: EDNAFULL
# Gap_penalty: 16
# Extend_penalty: 4
#
# Length: 66
# Identity:      42/66 (63.6%)
# Similarity:    42/66 (63.6%)
# Gaps:           2/66 ( 3.0%)
# Score: 102
#
#
#=======================================

dac_DACH1         62 TAAACAAATTCTTTTCGCTCTAAAAACTCT--CAAGTGTATCGATACGAT    109
                     ||||..||||.||||....||..|..||||  |||.|.|.||||.|.|||
XM_022942501.   1270 TAAAAGAATTTTTTTTTTCCTCGAGCCTCTAACAATTTTCTCGAGATGAT   1319

dac_DACH1        110 GCGTTTCTTTTTTTCC    125
                     .|.||||.|.|..|||
XM_022942501.   1320 TCTTTTCATGTCATCC   1335
```

→ **Hit:**
>XM_022942501.1_antisense
TAAAAGAATTTTTTTTTTCCTCGAGCCTCTAACAATTTTCTCGAGATGATTCTTTTCATGTCATCC

→ **BLASTN SEARCH** for XM_022942501.1_antisense: e-value 5.7e-30

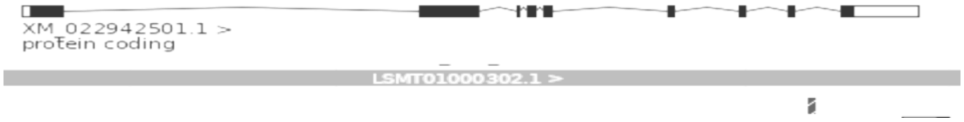

S1.3. *invected/ENGRAILED-2*

No homologs identified in any of the sequenced genomes of non-bilaterian species examined.

Supplementary Data Set S2

Xenacoelomorpha

Shown are the results of EMBOSS matcher sequence comparisons between the superconserved CREs identified in ref. 8 and the respective homologs in each of the investigated Xenacoelomorpha species. A match meeting the cutoff criteria of 60% sequence identity and 55bp length was blasted using Ensembl Blastn to identify its e-value (cutoff: 1e-1) and its position (criteria: intronic position). Each search was carried out with a) the sense and b) the antisense strand of the respective gene.

1. Hofstenia miamia

<u>HMIM012391</u>

Gene: **HMIM012391**
Transcript: HMIM012391-RA
Product: HMIM012391-PA

A

```
H.miamia_HMIM012391         --------MS----KGSPPNLIRP-----------NIP--TAVVCSGGNSSTSAGHGGVN      35
D.melanogaster_shaven       YSSRTLSNISDANTTPSANNFITQSQGIEWITAMNDIQNGAEDSHSSQGSISGDGHGGVN      180
M.musculus_Pax2             ----------------------------------MDM-----HCKADPFSAMHPGHGGVN      21
H.sapiens_PAX2              ----------------------------------MDM-----HCKADPFSAMHPGHGGVN      21
                                                               ::        :.  *    ******

H.miamia_HMIM012391         QLGGVYVNGRPLPDIVRQRIVDLAQSGVRPCDISRQLRVSHGCVSKILARFYETGSIRPG      95
D.melanogaster_shaven       QLGGVFVNGRPLPDVVRQRIVELAHNGVRPCDISRQLRVSHGCVSKILSRYYETGSFKAG      240
M.musculus_Pax2             QLGGVFVNGRPLPDVVRQRIVELAHQGVRPCDISRQLRVSHGCVSKILGRYYETGSIKPG      81
H.sapiens_PAX2              QLGGVFVNGRPLPDVVRQRIVELAHQGVRPCDISRQLRVSHGCVSKILGRYYETGSIKPG      81
                            *****:********:******:**:.**********************.*:*****:: *

H.miamia_HMIM012391         VIGGSKPKVATQQVVEKISDYKRQNPTMFAWEIRDRLLSETVCSQDNVPSVSSINRIVRN      155
D.melanogaster_shaven       VIGGSKPKVATPPVVDAIANYKRENPTMFAWEIRDRLLAEAICSQDNVPSVSSINRIVRN      300
M.musculus_Pax2             VIGGSKPKVATPKVVDKIAEYKRQNPTMFAWEIRDRLLAEGICDNDTVPSVSSINRIIRT      141
H.sapiens_PAX2              VIGGSKPKVATPKVVDKIAEYKRQNPTMFAWEIRDRLLAEGICDNDTVPSVSSINRIIRT      141
                            ***********  **: *::***:**************:* :*.:*.**********:*.

H.miamia_HMIM012391         KISEKAKEDETNA-----------------------------------------------      168
D.melanogaster_shaven       KAAEKAKHVHHHQQHHVSQSLGGGHIATESVDSSTGTIGEPQPPTSNSSANSVNTNVSAS      360
M.musculus_Pax2             KVQQPFHPTP--------DGAGTGVTAP----------G---------------------      162
H.sapiens_PAX2              KVQQPFHPTP--------DGAGTGVTAP----------G---------------------      162
                            *  :  :
```

B

```
H.miamia_HMIM012391         -------------------------------TNDCLDKTSQSYSINGILGMSRLEGAPDL      197
D.melanogaster_shaven       ASVHASIPTSGTDSVQVSVGHINANSNETTHINSTAEQRTTGYSINGILGIQHGHHSHNN      420
M.musculus_Pax2             ---HTIVP-----------------STASPPVSSASNDPVGSYSINGILGIPRSNGEKRK      202
H.sapiens_PAX2              ---HTIVP-----------------STASPPVSSASNDPVGSYSINGILGIPRSNGEKRK      202
                                                            ..  :.   .********: : .
```

C

```
H.miamia_HMIM012391         CRKRSYYNINFSDTKLKREDDSFTFAQPGYGAYPP-------------------------      232
D.melanogaster_shaven       NNNSSVNNNNNTESSCKRKR--IEAHDENHDTNIHSDNDDG-----KRQRMSTYSG----      469
M.musculus_Pax2             REEVEVYTDPAHIRGGGGLHLVWTLRDVSEGSVPNGDSQSGVDSLRKHLRADTFTQQQLE      262
H.sapiens_PAX2              RDE-----------------------DVSEGSVPNGDSQSGVDSLRKHLRADTFTQQQLE      239
                              :                       : . .:

H.miamia_HMIM012391         ------TAGCYPQIHHSKHFP--------DRTQMHPMVSAPLHSYYEKMSYFSAAAAAAS      278
D.melanogaster_shaven       -------DQLYTNIWSGKWCIKDDHKLLAELGNLTASTGNCPATYYEASNGFSTTPISGS      522
M.musculus_Pax2             ALDRVFERPSYPDVFQASEHIKSEQGNEYSLPALTPGLDEV-------KSSLSASANPEL      315
H.sapiens_PAX2              ALDRVFERPSYPDVFQASEHIKSEQGNEYSLPALTPGLDEV-------KSSLSASTNPEL      292
                                      * ::  ..           .   :    .          . :*::
```

D

```
# Aligned_sequences: 2
# 1: HMIM012391_sense
# 2: sv/PAX2
# Matrix: EDNAFULL
# Gap_penalty: 16
# Extend_penalty: 4
#
# Length: 78
# Identity:      48/78 (61.5%)
# Similarity:    48/78 (61.5%)
# Gaps:           6/78 ( 7.7%)
# Score: 84

#=======================================

SCFE01000039.    335 AATTTAGCATTATTTACAGGAATGT--AGCTAGGGCAGTCATTCCATTAT    382
                     |.||||..|||..||...|||.|.|  |||.|..|||  ||...|||||
sv/PAX2            7 ATTTTAATATTTCTTCAGGGACTTTCCAGCAACAGCA--CAAGTCATTA-     53

SCFE01000039.    383 CATTCACCCTTTTAAATTCATGAGTGAA    410
                      |||||||||...|||||..|.|.|.||
sv/PAX2           54 -ATTCACCCTCCCAAATTGCTTATTCAA     80
```

E

Hit:

```
>HMIM012391_sense_CRE-like
AATTTAGCATTATTTACAGGAATGTAGCTAGGGCAGTCATTCCATTATCATTCACCCTTTTAAATTCATGAGTGAA
```

→ **BLASTN SEARCH** for HMIM012391_sense_CRE-like: e-value 1.7e-35

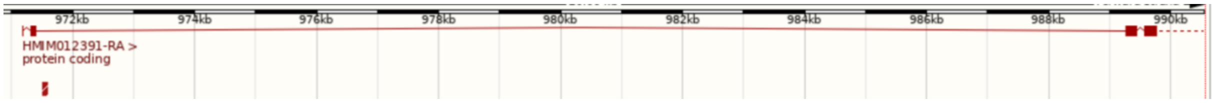

Shaven/PAX2 homolog and putative CRE-like element in *Hofstenia miamia HMIM012391 (Transcript: HMIM012391-PA)*. Panel A-C show Clustal sequence comparisons of *shaven/PAX2* specific motives in *H. miamia* (HMIM012391), *D. melanogaster shaven* (NP_524633.3), *M. musculus Pax2* (NP_001355672.1), and *H. sapiens PAX2* (NP_000269.39). (**A**) in bold, *shaven/PAX2*-Paired motive (**B**) in bold, *shaven/PAX2*-Octapeptide (**C**) in bold, *shaven/PAX2* partial Homeodomain. (**D**) shows the emboss matcher alignment of *H. miamia HMIM012391* and *sv/PAX2* CRE, see Fig. S1C. (**E**) shows the location of the putative CRE-like element in *H. miamia* on ensemblgenomes.org. Sequence comparisons in A-C were generated using Clustal Omega Multiple Sequence Comparison tool https://www.ebi.ac.uk/Tools/msa/clustalo/, local alignment in D was generated using https://www.ebi.ac.uk/Tools/psa/emboss_matcher/. The protein sequences of human, mouse and fly were retrieved from NCBI, *H. miamia* HMIM012391 was retrieved from ensemblgenome.org, genome assembly HmiaM1.

2. *Symsagittifera roscoffensis*

<u>2.1 sv/Pax2: Sros151_020267</u>

A

```
D.melanogaster_shaven     YSSRTLSNISDANTTPSANNFITQSQGIEWITAMNDIQNGAEDSHSSQGSISG--D-GHG
M.musculus_Pax2           -----------------------------------------MDMHCKADPFSAMHP-GHG
H.sapiens_PAX2            -----------------------------------------MDMHCKADPFSAMHP-GHG
S.roscoffensis_020267     ------------------------------------MDFEMFSDRCKQLKFFPDSDLGHG


D.melanogaster_shaven     GVNQLGGVFVNGRPLPDVVRQRIVELAHNGVRPCDISRQLRVSHGCVSKILSRYYETGSF
M.musculus_Pax2           GVNQLGGVFVNGRPLPDVVRQRIVELAHQGVRPCDISRQLRVSHGCVSKILGRYYETGSI
H.sapiens_PAX2            GVNQLGGVFVNGRPLPDVVRQRIVELAHQGVRPCDISRQLRVSHGCVSKILGRYYETGSI
S.roscoffensis_020267     GVNQLGGVYVNGRPLPDMVRQRIVDLAQSGVRPCDISRQLRVSHGCVSKILARFYETGSI


D.melanogaster_shaven     KAGVIGGSKPKVATPPVVDAIANYKRENPTMFAWEIRDRLLAEAICSQDNVPSVSSINRI
M.musculus_Pax2           KPGVIGGSKPKVATPKVVDKIAEYKRQNPTMFAWEIRDRLLAEGICDNDTVPSVSSINRI
H.sapiens_PAX2            KPGVIGGSKPKVATPKVVDKIAEYKRQNPTMFAWEIRDRLLAEGICDNDTVPSVSSINRI
S.roscoffensis_020267     RPGVIGGSKPKVATPGVVDKIGEYKRLNPTMFAWEIRDRLLQDNVCSSETVPSVSSINRI


D.melanogaster_shaven     VRNKAAEKAKHVHHHQQHHVSQSLGGGHIATESVDSSTGTIGEPQP--PTSNSSANSVNT
M.musculus_Pax2           IRTK------------------------------------VQQPFH--PTPDGAGTGV--
H.sapiens_PAX2            IRTK------------------------------------VQQPFH--PTPDGAGTGV--
S.roscoffensis_020267     VRNK------------------------------------IGDKLDGDGKRDSLGDSMS-
```

B

```
D.melanogaster_shaven     NVSASASVHASIPTSGTDSVQVSVGHINANSNETTHINSTAEQRTTGYSINGILGIQHGH
M.musculus_Pax2           ----TAPGHTIVPSTASPPV-----------------SSASNDPVGSYSINGILGIPR--
H.sapiens_PAX2            ----TAPGHTIVPSTASPPV-----------------SSASNDPVGSYSINGILGIPR--
S.roscoffensis_020267     ----TPGGHDCMDKDPQSHL----------------VSSSVTSNGSSYSINGILGMRDA-
```

C

```
D.melanogaster_shaven     IWSGKWCIKDD--HKLLAELGNLTASTGNCPATYYEASNGFS-TTPISGSGATASGNDTS
M.musculus_Pax2           VDSLRKHLRAD--TFTQQQLEALD--------RVFERPSYPD-VFQASEHIKSEQGNEYS
H.sapiens_PAX2            VDSLRKHLRAD--TFTQQQLEALD--------RVFERPSYPD-VFQASEHIKSEQGNEYS
S.roscoffensis_020267     HMHPSPHPMGPFAAFATAPQGHMGAA-GVGTYGATVGTGYPAYVHQHHNHAAHFNSGDTR
```

D

```
# Aligned_sequences: 2
# 1: sv_PAX2
# 2: 020267_antisense
# Matrix: EDNAFULL
# Gap_penalty: 16
# Extend_penalty: 4
#
# Length: 76
# Identity:      47/76 (61.8%)
# Similarity:    47/76 (61.8%)
# Gaps:           1/76 ( 1.3%)
# Score: 107
#
#
#=======================================

sv_PAX2          230 TAAAATAGGTTTTTGTTGCAATGCCCCCAGGATAAAGAATGGAAGGAGAC    279
                     |||||||...|||||.|..|||......||. ||||.|.|....|.|.||
020267_antise   2499 TAAAATATACTTTTGATTGAATTTGAAGAGT-TAAAAATTACTTGCAAAC   2547

sv_PAX2          280 TGAAGACAAGTTGGAGTTTATAAAAT    305
                     |||..|.||||...||||||.|||||
020267_antise   2548 TGAGAAAAAGTATCAGTTTAAAAAAT   2573
```

E

Hit:

>020267_antisense_CRE-like

TAAAATATACTTTTGATTGAATTTGAAGAGTTAAAAATTACTTGCAAACTGAGAAAAAGTATCAGTTTAAAAAAT

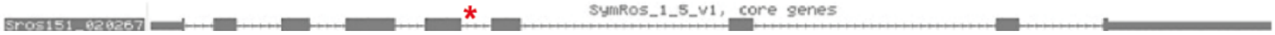

Shaven/PAX2 homolog and putative CRE-like element in *Symsagittifera roscoffensis Sros151_020267*. Panel A-C show Clustal sequence comparisons of *shaven/PAX2* specific motives in *S. roscoffensis* (*020267*), *D. melanogaster shaven* (NP_524633.3), *M. musculus Pax2* (NP_001355672.1), and *H. sapiens PAX2* (NP_000269.39). (**A**) in bold, *shaven/PAX2*-Paired motive (**B**) in bold, *shaven/PAX2*-Octapeptide (**C**) in bold, *shaven/PAX2* partial Homeodomain. (**D**) shows the emboss matcher alignment of *S. roscoffensis 020267* and *sv/PAX2* CRE, see Fig. S1C. (**E**) shows the location of the putative CRE-like element in *S. roscoffensis 020267* on macgenome.org. Sequence comparisons in A-C were generated using Clustal Omega Multiple Sequence Comparison tool https://www.ebi.ac.uk/Tools/msa/clustalo/, local alignment in D was generated using https://www.ebi.ac.uk/Tools/psa/emboss_matcher/. The protein sequences of human, mouse and fly were retrieved from NCBI, *S. roscoffensis 020267* was retrieved from UCSC Genome Browser (https://gb.macgenome.org, genome SymRos_1_5).

2.2 inv/EN2

A

S.roscoffensis_009290 --
S.roscoffensis_009293 --
D.melanogaster_invected GAVHPHQQLLLQRDQVHHHHMQNHLNNNENLHERALKFSIDNILKADFGSRLPKIGALS
H.sapiens_EN2 NHQHP**HRITNFFIDNI-----LRPEFG**RRKDAGTCCAGAG-----GGRGGGAGGEGGASG
M.musculus_En2 NHQHP**HRITNFFIDNI-----LRPEFG**RRKDAGTCCAGAG-----GAR----GGEGGAGT

B

S.roscoffensis_009290 ----------------------------------KSSCIPHNDYYKTTFPG
S.roscoffensis_009293 ---
D.melanogaster_invected VDLVKSPPPAAGAGATGASGKSGEDSGTPIV**WPAWVYCTRYSD-RPSSGRS**
H.sapiens_EN2 DLSVSSDSDSSQAGAN--------LGAQPML**WPAWVYCTRYSD-RPSS--G**
M.musculus_En2 DLSVSSDSDSSQASAT--------LGAQPML**WPAWVYCTRYSD-RPSS--G**

C

S.roscoffensis_009290 FLNIKPAASFGSKSILHATEMNTTSGKSR
S.roscoffensis_009293 --------------------MNTTSGKSR
D.melanogaster_invected **PRARKPKKP**ATSSSAAGGGGGGVEKGEAA
H.sapiens_EN2 **PRSRKP--------------------KKK**
M.musculus_En2 **PRSRKP--------------------KKK**

D

S.roscoffensis_009290 KFH**PGRRKRASFSKFQVDQLKKEFTHNCFIREENRVELAARLGMGENQVKVWFQNKRAKLRKKA**
S.roscoffensis_009293 KFH**PGRRKRASFSKFQVDQLKKEFTHNCFIREENRVELAARLGMGENQVKVWFQNKRAKLRKKA**
D.melanogaster_invected GVP**EDKRPRTAFSGTQLARLKHEFNENRYLTEKRRQQLSGELGLNEAQIKIWFQNKRAKLKKSS**
H.sapiens_EN2 PNK**EDKRPRTAFTAEQLQRLKAEFQTNRYLTEQRRQSLAQELSLNESQIKIWFQNKRAKIKKAT**
M.musculus_En2 PNK**EDKRPRTAFTAEQLQRLKAEFQTNRYLTEQRRQSLAQELSLNESQIKIWFQNKRAKIKKAT**

E

S.roscoffensis_009290 IHSQQSHCQHSYTN-TNHANTILSRSNDT-SMFQKP----------
S.roscoffensis_009293 IHSQQTHCQHSYTN-TNHANTILSRSNDT-SMFQKP----------
D.melanogaster_invected **GTKNPLALQLMAQGLYNHST**IPLTREEEELQELQEAASAAAAKEPC
H.sapiens_EN2 **GNKNTLAVHLMAQGLYNHST**TAKEGKSDS-E---------------
M.musculus_En2 **GNKNTLAVHLMAQGLYNHST**TAKEGKSDS-E---------------

Multiple sequence alignment of putative *S. roscoffensis inv/EN2* homologs Sros151_009290 and Sros151_009293 and *D. melanogaster invected* (NP_725057.2), *Homo sapiens EN2* (NP_001418.2) and *Mus musculus En2* (NP_034264.1) showing absence of *engrailed*-defining boxes EH1-EH3 and EH5 in *S. roscoffensis 009290* and *009293*, only homeodomain EH4 is present in all genes. Bold letters indicate *engrailed*-defining boxes: (**A**) EH1; (**B**) EH2; (**C**) EH3; (**D**) EH4; (**E**) EH5. Sequence alignment generated using the Kalign neighbour joining algorithm available at the European Bioinformatics Institute (www.ebi.ac.uk./Tools/msa/kalign/). The protein sequences of human, mouse and fly were retrieved from NCBI, *S. roscoffensis 009290* and *009293* were retrieved from UCSC Genome Browser (https://gb.macgenome.org, genome SymRos_1_5).

3. *Isodiametra pulchra*

3.1 *I. pulchra unplugged/Gbx*

```
>IP_SRR12179258_unpg_Gbx
GTTAAGGCTGGAATCACCTCGCACGGACTGGGTCGCAACGGGACCACTAGTGGCACCAAGATCTGGTGCCCATTCCCGTGCACGTGAACCGGCAGCA
CCAGCAGCTGGAGAAGATGTGCCTCAGCGGACCGAAGCCGGATCTGCGCAAGAAGCTCTCGGCGGAGGCGATCGGAGGCTTCGAGATTGGGCGTGGG
CGTGGGCGTTGGCGTGGGAGTCGGCCTGGGGGTATCCACGCCCCTCTCCCTCGCCAGGAGCATCTATTGATGGAGTTGGGCTCCTGAAGC
```

BlastN results generated with the NCBI SRA-nucleotide-Blast tool available under the accession numbers of the SRA dataset SRR12179258, input nucleotide sequence retrieved from ensemblgenomes.org, *Drosophila melanogaster* genome assembly BDGP6.32, *unplugged* FBgn0015561. NCBI BlastN shows two results that have been manually assembled in the order of input query coverage.

3.2 *I. pulchra engrailed*

```
>IP_SRR12179258_En
AACTAAAATACTCTTTCATAGCAATCCTTAAGTGTGTAAACTAGGAATTAAGCTAGGGAACTGTTAAGTTACTTAATTTACAGAGCGGTTGATTTAT
AGGGAGTAATAACTATAGATAGTGTTTTGTGTAGGAATAAACGTCTGAAGGTCGAAGCTTTTGAAGTGCGGCGATAGCGATAGGC
```

BlastN results generated with the NCBI SRA-nucleotide-Blast tool available under the accession numbers of the SRA dataset SRR12179258, input nucleotide sequence retrieved from ensemblgenomes.org, *Drosophila melanogaster* genome assembly BDGP6.32, *engrailed* FBgn0000577 (NCBI: NP_725057.2). NCBI BlastN shows two results that have been manually assembled in the order of input query coverage.

3.3 *I. pulchra optix/Six3*

A

```
>gnl|SRA|SRR12179258.247390287.2:1-91 247390287
GTTACAAGACCATTATTTGACGTATTTTTTGCGCGCTCGGTTGGCCGTTTGGTTGGTCGCTCTCTCGCTC
GCTGGATCGTATCGCACCACA
>gnl|SRA|SRR12179258.217183835.2:1-91 217183835
GAACCGCATCGGTTATATCCATTGCCAAAGGCCCCCTTTATCGCCTCGATGTGGAGCAGCAGTTGCGCTC
CGTTCGTGGTCCGGGCACGGG
>gnl|SRA|SRR12179258.244010364.2:1-91 244010364
ATCGCATTCATGAGCTGGCTCATTGGCAATCATTTGGATTCGTACGCCAGTCGAACCGGTGGCCACCTCA
CACATCCAGACGGCCAGATGC---
>gnl|SRA|SRR12179258.172502021.2:1-91 172502021
---ACGCCAGTCGAACCGGTGGCCACCTCACACATCCAGACGGCCAGATGC---CCTCGATCGCGCGTGCAAAGTG
CTAACCCCAAACCAATCAGTA
>gnl|SRA|SRR12179257.338083707.2:1-91 338083707
CTCGAGGACTCCGGCGACATCGAGCGGTTGGCCCGCTTCCTCTGGAGCCTGCCGGTGGCCCTGCCCAACA
TGCACGAGATCCTCAACTGCG
>gnl|SRA|SRR12179258.341798204.2:1-91 341798204
---CAACTGCG---AGGCGGTGCTCCGTGCCCGCGCAGTGGTCGCCTACCATGTGGGCAACTTCAGGTAGGAATCC
GCCACACACAAAAGCCCCCTG
>gnl|SRA|SRR12179258.273392242.2:1-91 273392242
ATTTGCGCATCTGCAGGAGCAGCCAAACACTTGAGTGGACTACGATTTCGACTCCAGATCTGCGACTCCA
GTCCAGTCCAGTGCAGCTCCG
>gnl|SRA|SRR12179258.272059081.2:1-91 272059081
CCTATACCTATACCTATACCGTTATCCAGCAGCAGCAAATCGCAAGCGTCGCACACTCACTCTTCAAAGA
TAGCCATTTAAGCCGGATGGA
>gnl|SRA|SRR12179258.270146834.2:1-91 270146834
---GCCGGATGGA---AACTGAGATACAGATTTAGAGCTAATACAGATAGCGGTCCCAGTGGCCCCAAAACTGAAA
TTGAATTCGAGACGTTTTCAT
```

B

```
>IP_SRR12179258_Optix_Six3
GTTACAAGACCATTATTTGACGTATTTTTTGCGCGCTCGGTTGGCCGTTTGGTTGGTCGCTCTCTCGCTCGCTGGATCGTATCGCACCACAGAACCG
CATCGGTTATATCCATTGCCAAAGGCCCCCTTTATCGCCTCGATGTGGAGCAGCAGTTGCGCTCCGTTCGTGGTCCGGGCACGGGATCGCATTCATG
AGCTGGCTCATTGGCAATCATTTGGATTCGTACGCCAGTCGAACCGGTGGCCACCTCACACATCCAGACGGCCAGATGCCCTCGATCGCGCGTGCAA
AGTGCTAACCCCAAACCAATCAGTACTCGAGGACTCCGGCGACATCGAGCGGTTGGCCCGCTTCCTCTGGAGCCTGCCGGTGGCCCTGCCCAACATG
CACGAGATCCTCAACTGCGAGGCGGTGCTCCGTGCCCGCGCAGTGGTCGCCTACCATGTGGGCAACTTCAGGTAGGAATCCGCCACACACAAAAGCC
CCCTGATTTGCGCATCTGCAGGAGCAGCCAAACACTTGAGTGGACTACGATTTCGACTCCAGATCTGCGACTCCAGTCCAGTCCAGTGCAGCTCCGC
CTATACCTATACCTATACCGTTATCCAGCAGCAGCAAATCGCAAGCGTCGCACACTCACTCTTCAAAGATAGCCATTTAAGCCGGATGGAAACTGAG
ATACAGATTTAGAGCTAATACAGATAGCGGTCCCAGTGGCCCCAAAACTGAAATTGAATTCGAGACGTTTTCAT
```

BlastN results generated with the NCBI SRA-nucleotide-Blast tool available under the accession numbers of the SRA dataset SRR12179258, input nucleotide sequence retrieved from ensemblgenomes.org, *Drosophila melanogaster* genome assembly BDGP6.32, *optix* FBgn0025360. NCBI BlastN reveals nine results (panel A) that have been manually assembled in the order of input query coverage (panel B). Dashed lines in A show manually detected overlap that has been cut in the assembly (panel B) to avoid doubling.

3.4 *I. pulchra labial/Hox1*

A

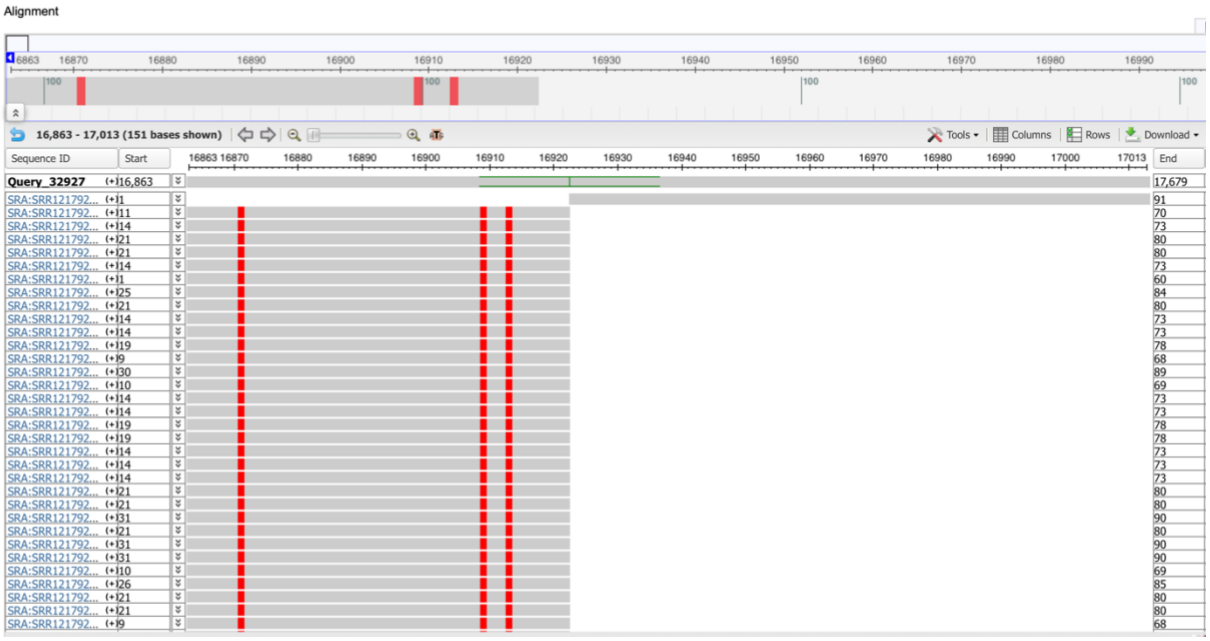

B

```
>IP_SRR12179258_lab_hox1
CAGGTCAAGATCTGGTTCCAGAACCGCCGCATGAAGCAGAAGAAGCTCGTCAAGGAGGGCGTATTATTAGCTAAGTTCTAGAATGCCCAAGTTATTC
GTTTTGTGCTTAAAAGTAATCGGTAAATCTTGATGTAAATCAACGTCTGTATAT
```

BlastN results generated with the NCBI SRA-nucleotide-Blast tool available under the accession numbers of the SRA dataset SRR12179258, input nucleotide sequence retrieved from ensemblgenomes.org, *Drosophila melanogaster* genome assembly BDGP6.32, *labial* FBgn0002522. NCBI BlastN reveals 34 results viewed along the input query via NCBI MSA viewer (panel A; red mark: nucleotide mismatch), of which 33 cover the same query sequence. Results have been manually assembled in the order of input query coverage (panel B).

3.4 *I. pulchra shaven/Pax2*

A

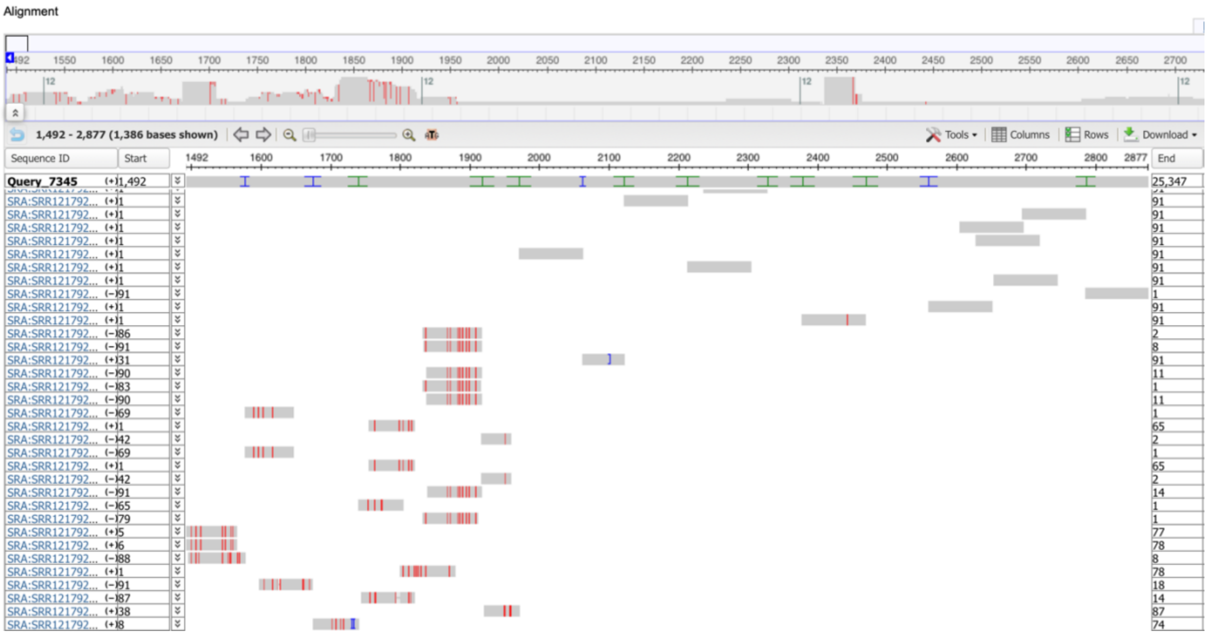

B

```
>IP_SRR12179258_sv_pax2
ACGGACAGTGCCAGATCGACTGGACTATTGATCCTGATCAAGAATATATATACTTTATATGGTCGCAAACGCTTCCTTCTAGCTTTTATAGTTCCTG
AGAATTCGACCTTCATATGGACGGACAGACTTCTTATGACATAGACGCAGCGCAAGGACAGATGGATAGGGTCAGATCGGCTCGGCTATTGATCCTG
ATCAAGAATATATATACTTAGGATGGTCGGAAACTCTTCCTGTCTATCCTTATGAACGTCGATTTAGGAACTATAAAAGCTAGAAGGTTGAGACTAA
GCATTCAGATTCTAGAGAGTATGTAACAGGCAGGAGGAAACGTTTTCGACTATATAAAGTATATATATTCTTGATCAGGATCAATAGCCGAGTCGAT
GTCTATCCTTATGAACGTCGATTTAGGAACTATAAAAGCTAGAAGGTTGAGACTAAGCATTCAGATTCTAGAGAAAAATAAGCCACAGCAACGAGAG
TGCTCGGTCCACTTTACTTATGCAAAGAAGTAGACAAACGAAAATCAAACTTCATCTTCAACAGCCTGAGTGCTTTTCTAGAACGTCAATCAGAACA
TCGGAGTTGCAAATCGGAGCACTTCTAGCCGTACTGAAAAAAGCAAATTAGTGAAAAACTGAGAATCATATAGTCATGTATTTTAAATTTAAATGAC
ACGTGAATTTTTTTTGTGTTCCTTTGACAATTAGTGCTCTCAAATTTTTTTAACTCCCTTCTATCTGTTACGTACTTTTAAACGCATCTGTTATGGC
CTTTTACCTAATAGTAACGGAAAGGGTTGTTAAAAAAATGATAATTTTAATCGAAAAGCAACGTTGCATTTTTGGGTTATGTTCTCGTGTTTTTTGG
TAATAATAAAAAAGAGAACAAACATCACTACATGTGAAAAACAAAATCTGTGCAAAAAGATTCGCATTTATTAATAAATTTAGGTTACAATATAAGC
AGGTATATAGCAATTCTTCTTTTCTAAAATAACTTTAGCTGAAAATATAGCTTGGCATTTTTTTATTCTTCAAAAAATATCTAAGTGGCATGGTAATT
CGACCGTAGAAGCCTTGTGCGTACACAATGCGTGGTTTTCTTGGCCTTTGTAAGATACACATCAAAAATCAAAAACCTGTAACATTGTTTCAATTTT
GTGTCGAAACATGATTTCGCGTGTACACTATAAAAATGTAATCATTCAGCCTAAAAATAGGCCTGGAGAAATCGAATTCAAAGGGAA
```

BlastN results generated with the NCBI SRA-nucleotide-Blast tool available under the accession numbers of the SRA dataset SRR12179258, input nucleotide sequence retrieved from ensemblgenomes.org, *Drosophila melanogaster* genome assembly BDGP6.32, *shaven* FBgn0005561 (NP_524633.3). NCBI BlastN reveals 52 results viewed along the input query via NCBI MSA viewer (panel A; red mark: nucleotide mismatch), that have been manually assembled in the order of input query coverage (panel B).

3.5 *I. pulchra dac/DACH1*

A

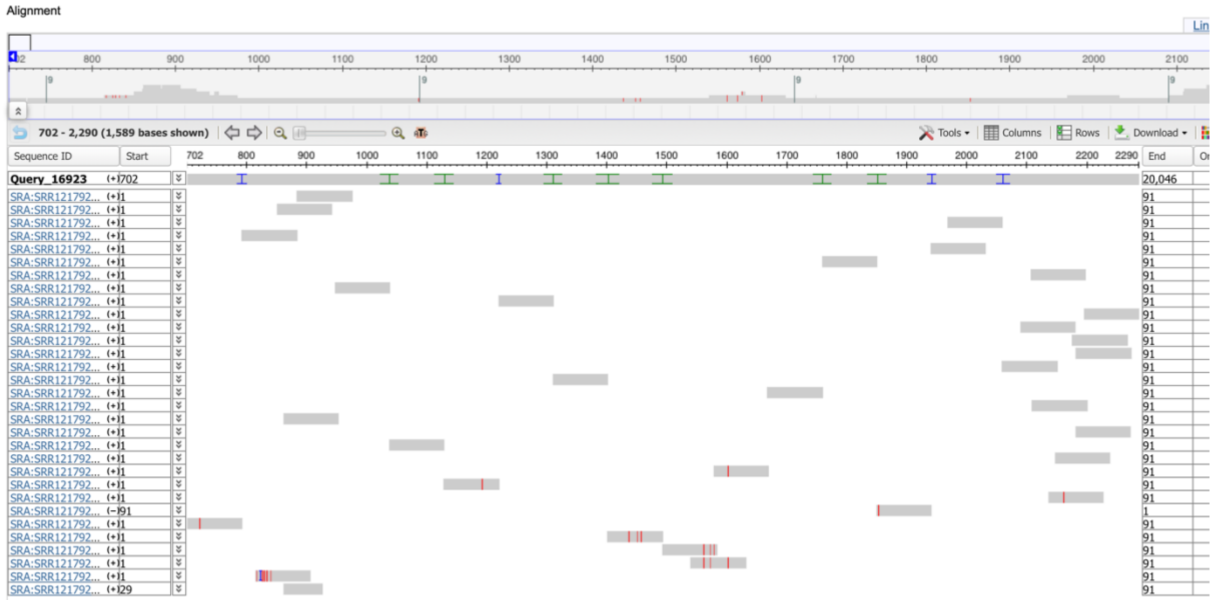

B

>IP_SRR12179258_dac
GTACGTGTGTGTGGGTGGGCTAAAAAATAGCACTCGTTAGCGCCCGCTCACTCGCTCACTCGCAGTCGTCGCCTCGCGCCGTCTCGCTCTCCAGTCG
AAGGCGAAGAAGCAGAGGCAGCGACGCCACCGTCGCCGTCGCAGCGGAGTAGCTGCCATCTCTCAATCTCTGAGTGACAATCCGTGGTACGGTACTC
GAAAACGGACACAGCTCCGGTTTCCAAAATTCCAGTACGAAAACGGTTATTCGAACTCGATTAACACAAGCGGGCAATCGGAATAAATAACTTGAAA
TATTCAGAGTATATATTTCACAGCGAAATTCAAAAAGCAAAGTGCGAGCTCTTCCTGAAGCACTTGGTCGGCGGTCTGCATACGGTTTATACCAAAC
TAAAGCGCCTTGACATCGTACCGCTCGTCTGCAATGTGGCTATCGATCAAGCCACCAGAAAGGTAAACACACAAGTATAATAAAAAAACTATGGCAA
TAACTGTTCTCCTCCTACTTTTTGGTTTTTTTTATTATTTATTTAACGAAGGAAAAAAAGAAACGCATCGTATCGATACACTTGAGAGTTTTTAGAG
CGAAAAGAATTTGTTTATTAAATTAATTTTAAACTTATATTCGGGAAAACCCCAAAAAGCCATTTAACCAAAATAGCAAAATGAATAAAATATTCCC
ATAAATCTCACCAACCGAGGGTACGAGGACATCGTCAAGCATTTGGAGCGATTGCGGGAGGAGCGAACGGATGAGCGTCAGCAGGCGATGGGTGTGG
GTGGTGCGGTCGTCGACTTAAAGAAGCTTTATATGAATTGAAAAGGGTATTTGATAATGTGTTGTTCTACACAAAAATAATAACAATGGCTTTAAGG
AAAATATTCTTTTCGATTTGCAAATTGGAAAATGAACTAGTTTGAAGTAATTTCATGTAATGTTCCAATCAAAAACTATTTTACTCAGTAATTTTTC
AATTTGCACTTTTTAAAAATTGATCCATATATCGGCGGCCACTTTGTAATCAATAATCGCAATTCATTAGGCTTACAACTACTAAAGTTTGCAATCC
GACGCGAGCGAAAGATCCGCATCCGCTATCAGCAGCAGCTTGGTGGCGGCAGCGGTGCGGTGAAGGGCAGTCCGAATGCCAACGGGAGTTCTTTACT
CCCTAAATAACTACGTATTACTTTATTTATTATATATTTCTGTTACATTAATATACTCGAATTGAAGCTAAATCGTAAGGTTCTTTGATTTATTTTA
CGATGTAGTTTTTCTAATGCTAAGGTTTTAATTTATTAACTAGCCTAGCAGGAGTGCAGTAAGTTCGTAATACTAAAAATTAGCATATAAAAATTAA
TAACTTCGCTATTATATATACACCCTATATATATACACACATTAACTTTAGTCATAACAATAGTCAAAGAACAAATAATTCCGCGAATCAGCAAACA
ACAATAACGGTTCAAATATTGTGCAACATTTCGCCGCTCGCAGTGAAAACACAGAAATTATTATAATAAATACGATTAAAATAATACAGCAGCCGAC
AACCGACAGACAAAACCAACACCAGCAACCAAGCGAAAAAG

BlastN results generated with the NCBI SRA-nucleotide-Blast tool available under the accession numbers of the SRA dataset SRR12179258, input nucleotide sequence retrieved from ensemblgenomes.org, *Drosophila melanogaster* genome assembly BDGP6.32, *dac* FBgn0005677 (NP_001014486.1). NCBI BlastN reveals 31 results viewed along the input query via NCBI MSA viewer (panel A; red mark: nucleotide mismatch), that have been manually assembled in the order of input query coverage (panel B).

4. Xenoturbella bocki

```
>XB_SRR5760181_En
TTCTTGATCTTGGCGCGCTTGTTCTGGAACCAGATCTTGATCTGGGACTCGTTGAGGC

>XB_SRR8524532_dac
TGTGGTACGTACGTACGTACGTGTGTGTGGGTG

>XB_SRR8524534_Optix
TACCTATACCTTTACCTATACCTATACCTATACC

>XB_SRR8524534_Otx
CATGATGTCTTACCTCAAACAACCCCCATACGGCATGAACGGGCTGGGCCT

>XB_SRR8524534_Msx
CTGGAGCGCAAGTTCCGCCAGAAGCAGTACCTGTCTATTGCCGAGCGCGCGCTGAGGAACGATTTTTATCTGGGGATTAAACGTCTTTGAAAAGACA
G-CCC
```

BlastN results generated with the NCBI SRA-nucleotide-Blast tool available under the accession numbers of the SRA datasets: *en* was identified in dataset SRR5760181, *dac* in SRR8524532, *Optix*, *Otx* and *Msx* in SRR8524534. Input nucleotide sequences were retrieved from ensemblgenomes.org, *Drosophila melanogaster* genome assembly BDGP6.32, accession numbers: *engrailed* FBgn0000577, *dachshund* FBgn0005677, *optix* FBgn0025360, and from ensemble.org human genome GRCh38.p13 *Otx* OTX1 and *Msx* MSX1.

Supplementary Data Set S3

shaven/PAX2

Shown are the results of EMBOSS matcher sequence comparisons between the superconserved *shaven/PAX2* CRE identified in ref. 8 and the respective *shaven/PAX2* homolog of each species. A match meeting the cutoff criteria of 60% sequence identity and 55bp length was blasted using Ensembl Blastn to identify its e-value (cutoff: 1e-1) and its position (criteria: intronic position). Each search was carried out with a) the sense and b) the antisense strand of the respective gene.

1. *Strongylocentrotus purpuratus*

Description: Pax-2a-like
Gene: LOC115918459
Transcript: XM_030979663
Product: XP_030835523

A

```
# Aligned_sequences: 2
# 1: LOC115918459_sense
# 2: sv/PAX2
# Matrix: EDNAFULL
# Gap_penalty: 16
# Extend_penalty: 4
#
# Length: 93
# Identity:      56/93 (60.2%)
# Similarity:    56/93 (60.2%)
# Gaps:           4/93 ( 4.3%)
# Score: 96
#
#
#=======================================

LOC115918459_  31081 TCCAGTGCATCGTTGAGTACGAATGGAATGG--CTGGACACAAGCTAAAT  31128
                     |.||.|||..|...||..|.||||||||.|.  |||.|.|||||.|..|.
sv/PAX2          246 TGCAATGCCCCCAGGATAAAGAATGGAAGGAGACTGAAGACAAGTTGGAG    295

LOC115918459_  31129 TTGACCAAA-CAAAAGAATGAAGT-CACAAAGCCTATGCTTTT  31169
                     ||.|..||| |||..||||..|.| ||||....||..||.|||
sv/PAX2          296 TTTATAAAATCAATGGAATTTATTGCACAGCTACTTGGCATTT    338
```

➔ **Hit:**
>LOC115918459_sense_CRE-like
TCCAGTGCATCGTTGAGTACGAATGGAATGGCTGGACACAAGCTAAATTTGACCAAACAAAAGAATGAAGTCACAAAGCCTATGCTTTT

➔ **BLASTN SEARCH** for LOC115918459_sense_CRE-like: e-value 3.6e-43

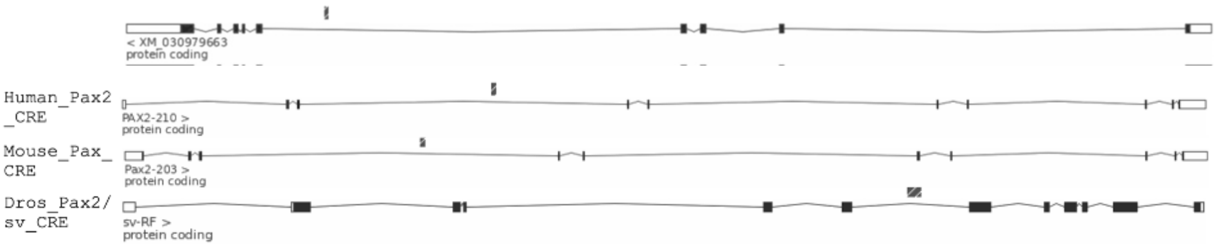

B

```
# Aligned_sequences: 2
# 1: LOC115918459_antisense
# 2: sv/PAX2
# Matrix: EDNAFULL
# Gap_penalty: 16
# Extend_penalty: 4
#
# Length: 133
# Identity:      74/133 (55.6%)
# Similarity:    74/133 (55.6%)
# Gaps:           7/133 ( 5.3%)
# Score: 98
#
#
#=======================================

LOC115918459_  27628 TTCAAAATTATGTATGAAAGAAAAATCGCATGTATACACAAT---GTACA  27674
                     |.|.|||||...|||..||.|..|....||||..|.|..|||   ||.|.
sv/PAX2           62 TCCCAAATTGCTTATTCAATAGCAGGAACATGGCTCCTGAATAAAGTCCC    111

LOC115918459_  27675 AGTCTCTATCATTTTCATATTAGTCAGGATGGTCAAACTCGTATTCTCTC  27724
                     ||..|.|||.....||..|..|||||||| ||||||||...|.||.|...
sv/PAX2          112 AGAATTTATGTGACTCGCACGAGTCAGGA-GGTCAAACAACTGTTATGGA    160

LOC115918459_  27725 GAGATATTAACA---CCAACAAGATGCTTAAAC  27754
                     |.||..||||.|   |.||.||.||..|.|.||
sv/PAX2          161 GCGAAGTTAAAAATCCAAATAAAATATTGACAC    193
```

→ below the threshold criteria

2. Saccoglossus kowalevskii

Description: PREDICTED: Pax-2-B isoform X1
NCBI Gene: 100328996
Transcript: XM_006813003.1
Product: XP_006813066.1

A

```
# Aligned_sequences: 2
# 1: XP_006813066.1_sense
# 2: sv/PAX2
# Matrix: EDNAFULL
# Gap_penalty: 16
# Extend_penalty: 4
#
# Length: 47
# Identity:      32/47 (68.1%)
# Similarity:    32/47 (68.1%)
# Gaps:           1/47 ( 2.1%)
# Score: 88
#
#
#=======================================

XP_006813066.   3543 GGAGGTCTAGTGAAGGAAGTACAAATATTCAAATTATGTTGTTTTTG   3589
                     |||||...|| |..|||||.|.|..||||.|||.||.|||.||.|||
sv/PAX2          202 GGAGGGGGAG-GTGGGAAGCAAAGGTATTTAAAATAGGTTTTTGTTG    247
```

→ below the threshold criteria

B

```
# Aligned_sequences: 2
# 1: XP_006813066.1_antisense
# 2: sv/PAX2
# Matrix: EDNAFULL
# Gap_penalty: 16
# Extend_penalty: 4
#
# Length: 40
# Identity:      29/40 (72.5%)
# Similarity:    29/40 (72.5%)
# Gaps:           1/40 ( 2.5%)
# Score: 89
#
#
#=======================================

XP_006813066.  15271 ACACGACAATTGATTCAATCTCGCAAATTGCCAAATAAAT  15310
                     |||.|.|| ||.|||||..|||.||||||||..|.|.|||
sv/PAX2           43 ACAAGTCA-TTAATTCACCCTCCCAAATTGCTTATTCAAT     81
```

→ below the threshold criteria

3. Branchiostoma lanceolatum

Gene: BL19642
Transcript: BL19642_evm1
Protein: BL19642_evm1

A

```
# Aligned_sequences: 2
# 1: BL19642_sense
# 2: sv/PAX2
# Matrix: EDNAFULL
# Gap_penalty: 16
# Extend_penalty: 4
#
# Length: 64
# Identity:      38/64 (59.4%)
# Similarity:    38/64 (59.4%)
# Gaps:           4/64 ( 6.2%)
# Score: 74
#
#
#=======================================

BL19642_sense    914 AAATCTAATAT----ACTTTACAGTCAAAGTGCATTTTGTTATGAAAGGT    959
                     ||||..|||||    |||||...|...|.|.|.|..|.|..|..||||||
sv/PAX2          177 AAATAAAATATTGACACTTTTTTGGGGAGGGGGAGGTGGGAAGCAAAGGT    226

BL19642_sense    960 ATTTTCAATCTGTT    973
                     ||||..|||..|||
sv/PAX2          227 ATTTAAAATAGGTT    240
```

→ below the threshold criteria

B

```
# Aligned_sequences: 2
# 1: BL19642_antisense
# 2: sv/PAX2
# Matrix: EDNAFULL
# Gap_penalty: 16
# Extend_penalty: 4
#
# Length: 76
# Identity:      49/76 (64.5%)
# Similarity:    49/76 (64.5%)
# Gaps:           7/76 ( 9.2%)
# Score: 101
#
#
#=======================================

BL19642_antis   1765 GGTCAAACTACAA--ATGGAGGGATGTTAAAAGATC----TTTGATATTA   1808
                     ||||||||.||..  ||||||.||.||||||| |||    |...|||||.
sv/PAX2          141 GGTCAAACAACTGTTATGGAGCGAAGTTAAAA-ATCCAAATAAAATATTG    189

BL19642_antis   1809 AACATGTTTTTGCCAGGTGCATCTGG   1834
                     |...|.||||.|..|||.|.|..|||
sv/PAX2          190 ACACTTTTTTGGGGAGGGGGAGGTGG    215
```

→ **Hit:**

>BL19642_antisense_CRE-like
GGTCAAACTACAAATGGAGGGATGTTAAAAGATCTTTGATATTAAACATGTTTTTGCCAGGTGCATCTGG

→ **BLASTN SEARCH** for BL19642_antisense_CRE-like: e-value 3.1e-32

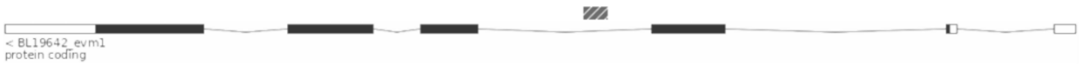

4. Ciona intestinalis

4.a Gene ENSCING00000005977 (PAX2/5/8-b)

Description: pax2/5/8-b
Gene: ENSCING00000005977
Transcript: ENSCINT00000012344.3
Product: ENSCINP00000012344

A

```
# Aligned_sequences: 2
# 1: ENSCING00000005977_sense
# 2: sv/PAX2
# Matrix: EDNAFULL
# Gap_penalty: 16
# Extend_penalty: 4
#
# Length: 105
# Identity:      64/105 (61.0%)
# Similarity:    64/105 (61.0%)
# Gaps:           9/105 ( 8.6%)
# Score: 108
#
#
#=======================================
ENSCING000000   5210 AAACCTAATTAATATAATTTATGGTTGAACTT-----ATGTTTAAAAAGG   5254
                     |||..||.||||.|||..||.|.||||.|.|.     |.|.|.||.||.|
sv/PAX2          221 AAAGGTATTTAAAATAGGTTTTTGTTGCAATGCCCCCAGGATAAAGAATG    270

ENSCING000000   5255 GAAG-ATAATTAAAAATAGT-GCA--TTATAAGAGTAAAGGAAAGCATTA   5300
                     |||| |.|.|.||.|..||| |.|  ||||||.|..||.||||...|||.
sv/PAX2          271 GAAGGAGACTGAAGACAAGTTGGAGTTTATAAAATCAATGGAATTTATTG    320

ENSCING000000   5301 CACTG   5305
                     |||.|
sv/PAX2          321 CACAG    325
```

→ **Hit:**

```
>ENSCING00000005977_sense_CRE-like
AAACCTAATTAATATAATTTATGGTTGAACTTATGTTTAAAAAGGGAAGATAATTAAAAATAGTGCATTATAAGAGTAAAGGAAAGCATTACACTG
```

→ **BLASTN SEARCH** for ENSCING00000005977_sense_CRE-like: e-value: 5e-48

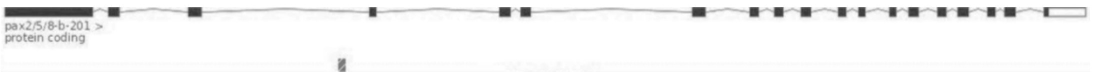

B

```
# Aligned_sequences: 2
# 1: ENSCING00000005977_antisense
# 2: sv/PAX2
# Matrix: EDNAFULL
# Gap_penalty: 16
# Extend_penalty: 4
#
# Length: 54
# Identity:      36/54 (66.7%)
# Similarity:    36/54 (66.7%)
# Gaps:           2/54 ( 3.7%)
# Score: 84
#
#
#=======================================
ENSCING000000   5875 AGGAGACTTAA-ATTAGCTGCACTTTTTAACAAAAAACCGAACATGTTGC   5923
                     ||||||||.|| |..||.||.|.|||.||| ||..||..|||..|.||||
sv/PAX2          273 AGGAGACTGAAGACAAGTTGGAGTTTATAA-AATCAATGGAATTTATTGC    321

ENSCING000000   5924 CCTG   5927
                     .|.|
sv/PAX2          322 ACAG    325
```

→ **below the threshold criteria**

4.b Gene ENSCING00000006915 (PAX2/5/8)

Description: pax258
Gene: ENSCING00000006915
Transcript: ENSCINT00000014209.3
Product: ENSCINP00000014209

A

```
# Aligned_sequences: 2
# 1: ENSCING00000006915_sense
# 2: sv/PAX2
# Matrix: EDNAFULL
# Gap_penalty: 16
# Extend_penalty: 4
#
# Length: 80
# Identity:      47/80 (58.8%)
# Similarity:    47/80 (58.8%)
# Gaps:           1/80 ( 1.2%)
# Score: 91
#
#
#=======================================

ENSCING000000    380 AGTGAAACATAAAAATATTATTTTTATAATTATTCGCTTGTTAAAAATAT    429
                     |||.|||.||..|||||..||.||.|.|.||.||.|.. |......|..|
sv/PAX2          165 AGTTAAAAATCCAAATAAAATATTGACACTTTTTGGG-GAGGGGGAGGT    213

ENSCING000000    430 TTTAAGTAAAAATTTTTAAAAAAAGTTTGT    459
                     ...|||.|||..|.|||||||.|.||||.|
sv/PAX2          214 GGGAAGCAAAGGTATTTAAAATAGGTTTTT    243
```

→ below the threshold criteria

B

```
# Aligned_sequences: 2
# 1: ENSCING00000006915_antisense
# 2: sv/PAX2
# Matrix: EDNAFULL
# Gap_penalty: 16
# Extend_penalty: 4
#
# Length: 58
# Identity:      40/58 (69.0%)
# Similarity:    40/58 (69.0%)
# Gaps:           3/58 ( 5.2%)
# Score: 104
#
#
#=======================================

ENSCING000000   4428 AGTTTTCATATTTAATTACGAACTTTTGGCAGCCCCA-CATAAATAATTG   4476
                     |.||||.||||||..|.|.|.|||||  .||||..|| ||.||.|.|||.
sv/PAX2            6 AATTTTAATATTTCTTCAGGGACTTT--CCAGCAACAGCACAAGTCATTA     53

ENSCING000000   4477 AATCACCC   4484
                     |.||||||
sv/PAX2           54 ATTCACCC     61
```

→ below the threshold criteria

5. *Lingula anatina*

Gene: g1632
Transcript: g1632.t1
Protein: g1632.t1

A

```
# Aligned_sequences: 2
# 1: g1632_sense
# 2: sv/PAX2
# Matrix: EDNAFULL
# Gap_penalty: 16
# Extend_penalty: 4
#
# Length: 99
# Identity:      60/99 (60.6%)
# Similarity:    60/99 (60.6%)
# Gaps:           4/99 ( 4.0%)
# Score: 108
#
#
#=======================================
g1632_sense    15291 GATAGAAAATATAATGAGAAAAAAGAATAAGTTGGTTTTCAAAACATTCT  15340
                     ||||.|.|||..||.||||...||||. |||||||..||.|.||.||...
sv/PAX2          260 GATAAAGAATGGAAGGAGACTGAAGAC-AAGTTGGAGTTTATAAAATCAA    308

g1632_sense    15341 TGGTAATTCATCATCAGGCATTGTAGCTTTTCCTTTTTTCTTCTCCTTT  15389
                     |||.|.||..|...|||..|.| |.||.|||..|..||.  ||||.|||
sv/PAX2          309 TGGAATTTATTGCACAGCTACT-TGGCATTTAATAATTC--TCTCATTT    354
```

→ Hit:

```
>g1632_sense_CRE-like
GATAGAAAATATAATGAGAAAAAAGAATAAGTTGGTTTTCAAAACATTCTTGGTAATTCATCATCAGGCATTGTAGCTTTTCCTTTTTTCTTCTCCT
TT
```

→ BLASTN SEARCH for g1632_sense_CRE-like: e-value: 2e-49

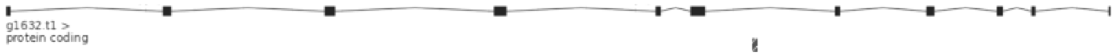

B

```
# Aligned_sequences: 2
# 1: g1632_antisense
# 2: sv/PAX2
# Matrix: EDNAFULL
# Gap_penalty: 16
# Extend_penalty: 4
#
# Length: 74
# Identity:      47/74 (63.5%)
# Similarity:    47/74 (63.5%)
# Gaps:           8/74 (10.8%)
# Score: 103
#
#
#=======================================
g1632_antisen  17815 GCAGTGAAAAATGCAGATATTTAAAAATGTTTAAAAGGACTTGGAGAGGG  17864
                     |.|||.||||||.||.|||     ||||.||.|.|.....||||.|||||
sv/PAX2          163 GAAGTTAAAAATCCAAATA-----AAATATTGACACTTTTTTGGGGAGGG    207

g1632_antisen  17865 GGAAGA---AAACAAAATAATTTA  17885
                     |||.|.   ||.||||...|||||
sv/PAX2          208 GGAGGTGGGAAGCAAAGGTATTTA    231
```

→ **Hit:**

>g1632_antisense_CRE-like
GCAGTGAAAAATGCAGATATTTAAAAATGTTTAAAAGGACTTGGAGAGGGGGAAGAAAACAAAATAATTTA

→ **BLASTN SEARCH** for g1632_antisense_CRE-like: e-value 7e-33

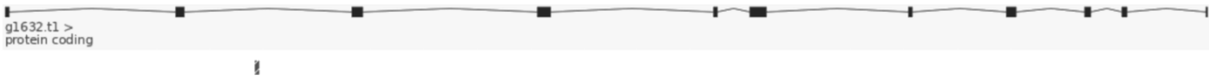

6. *Capitella teleta*

Gene: CapteG119889
Transcript: CapteT119889
Protein: CapteP119889

A

```
# Aligned_sequences: 2
# 1: CapteG119889_sense
# 2: sv/PAX2
# Matrix: EDNAFULL
# Gap_penalty: 16
# Extend_penalty: 4
#
# Length: 59
# Identity:      37/59 (62.7%)
# Similarity:    37/59 (62.7%)
# Gaps:           4/59 ( 6.8%)
# Score: 73
#
#
#=======================================
CapteG119889_   1015 GACCTTATATAATATATGCGTGTGGTTGTACA--TTGTTGGTACATTTTT   1062
                     ||..|||||.|||..|||....|..|||.|||  |..||||  |||||..
sv/PAX2          293 GAGTTTATAAAATCAATGGAATTTATTGCACAGCTACTTGG--CATTTAA    340

CapteG119889_   1063 TCGTTCTCT   1071
                     |..||||||
sv/PAX2          341 TAATTCTCT    349
```

→ Hit:

```
>CapteG119889_sense_CRE-like
GACCTTATATAATATATGCGTGTGGTTGTACATTGTTGGTACATTTTTTCGTTCTCT
```

→ BLASTN SEARCH for CapteG119889_sense_CRE-like: e-value: 9.1e-25

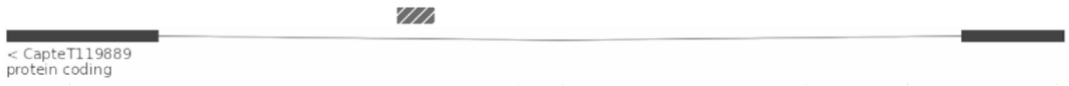

B

```
# Aligned_sequences: 2
# 1: CapteG119889_antisense
# 2: sv/PAX2
# Matrix: EDNAFULL
# Gap_penalty: 16
# Extend_penalty: 4
#
# Length: 106
# Identity:      61/106 (57.5%)
# Similarity:    61/106 (57.5%)
# Gaps:           6/106 ( 5.7%)
# Score: 77
#
#
#=======================================
CapteG119889_    473 GAACCTAAACATTTGCAAAGAGTTTGTTTTTGATTAATTGACATTAAT-T    521
                     |||.|.||....||..|||.||   |||||||.|..|.||.|...|.. |
sv/PAX2          216 GAAGCAAAGGTATTTAAAATAG---GTTTTTGTTGCAATGCCCCCAGGAT    262

CapteG119889_    522 AACGCAGTGAAACA-ATTGGGTATAAAATGGAGCATGCACAA-CAATGAC    569
                     ||.|.|..|||..| |.||...|.||..|||||..|..|.|| |||||..
sv/PAX2          263 AAAGAATGGAAGGAGACTGAAGACAAGTTGGAGTTTATAAAATCAATGGA    312

CapteG119889_    570 ATTTAT    575
                     ||||||
sv/PAX2          313 ATTTAT    318
```

→ below the threshold criteria

7. Octopus bimaculoides

Gene: Ocbimv22007526m.g
Transcript: Ocbimv22007526m
Protein: Ocbimv22007526m.p

A

```
# Aligned_sequences: 2
# 1: Ocbimv22007526m.g_sense
# 2: sv/PAX2
# Matrix: EDNAFULL
# Gap_penalty: 16
# Extend_penalty: 4
#
# Length: 42
# Identity:      32/42 (76.2%)
# Similarity:    32/42 (76.2%)
# Gaps:           3/42 ( 7.1%)
# Score: 96
#
#
#=======================================

Ocbimv2200752   8687 TATTGCTCTGC-ATTTTGCATTTAATGTAATGCTCGCATTAC   8727
                     ||||||.|.|| |.||.|||||||||  |||.|||.||||.|
sv/PAX2          316 TATTGCACAGCTACTTGGCATTTAAT--AATTCTCTCATTTC    355
```

→ below the threshold criteria

B

```
# Aligned_sequences: 2
# 1: Ocbimv22007526m.g_antisense
# 2: sv/PAX2
# Matrix: EDNAFULL
# Gap_penalty: 16
# Extend_penalty: 4
#
# Length: 192
# Identity:     105/192 (54.7%)
# Similarity:   105/192 (54.7%)
# Gaps:          14/192 ( 7.3%)
# Score: 93
#
#
#=======================================

Ocbimv2200752   3250 AAATATTCAACAAATATAGTTGACATTTACAGTGGTGAATTGTACTTCGC   3299
                     |||.||.|||..||.||| ||||||.||... |||.||...|.|..|.|.
sv/PAX2          169 AAAAATCCAAATAAAATA-TTGACACTTTTT-TGGGGAGGGGGAGGTGGG    216

Ocbimv2200752   3300 TAATATACCTATTTAAATGAGCGTCATGAT--------CGCAGCAACAGA   3341
                     .|..|.|..||||||||..||..|..||.|        |.|||.|..|..
sv/PAX2          217 AAGCAAAGGTATTTAAAATAGGTTTTTGTTGCAATGCCCCCAGGATAAAG    266

Ocbimv2200752   3342 AATCAAACTGATATATTAAGACCAAGTATTAGCGTAGTAATAGAAATTGA   3391
                     |||..||. ||.|. |.||||| ||||...||..|| |||.|..|||.||
sv/PAX2          267 AATGGAAG-GAGAC-TGAAGAC-AAGTTGGAGTTTA-TAAAATCAATGGA    312

Ocbimv2200752   3392 ATAAACTACACTTATCAATCGATTTGTAGGTTGCTCTCTCTT   3433
                     ||..|.|.|||...|...|.|..||..|...|.|||||..||
sv/PAX2          313 ATTTATTGCACAGCTACTTGGCATTTAATAATTCTCTCATTT    354
```

→ below the threshold criteria

8. *Schistosoma mansoni*

Description: Putative paired box protein pax-6
Gene: Smp_142940
Transcript: Smp_142940.1
Protein: Smp_142940.1

A

```
# Aligned_sequences: 2
# 1: Smp_142940.1_sense
# 2: sv/PAX2
# Matrix: EDNAFULL
# Gap_penalty: 16
# Extend_penalty: 4
#
# Length: 108
# Identity:      65/108 (60.2%)
# Similarity:    65/108 (60.2%)
# Gaps:          14/108 (13.0%)
# Score: 93
#
#=======================================

Smp_142940.1_   8242 CAAGATACAGAAAATAAGGATATCTATAGACTAAGTTGACTTTCATATCG   8291
                     ||.||||.||||...|||||.| ||..||||.|..|.||.|||
sv/PAX2          257 CAGGATAAAGAATGGAAGGAGA-CTGAAGACAAGTTGGAGTTT-------    298

Smp_142940.1_   8292 CATAACCTTTCTGGAATTACTATGAAC--CTAGTTTAAAAAATCAATAAT   8339
                      ||||..|...|||||||..| ||.||  |||.||...|.  |.||||||
sv/PAX2          299 -ATAAAATCAATGGAATTTAT-TGCACAGCTACTTGGCAT--TTAATAAT    344

Smp_142940.1_   8340 TATCTAAT   8347
                     |.|||.||
sv/PAX2          345 TCTCTCAT    352
```

→ **Hit:**

```
>Smp_142940.1_sense_CRE-like
CAAGATACAGAAAATAAGGATATCTATAGACTAAGTTGACTTTCATATCGCATAACCTTTCTGGAATTACTATGAACCTAGTTTAAAAAATCAATAA
TTATCTAAT
```

→ **BLASTN SEARCH** for Smp_142940.1_sense_CRE-like: e-value: 1.4e-53

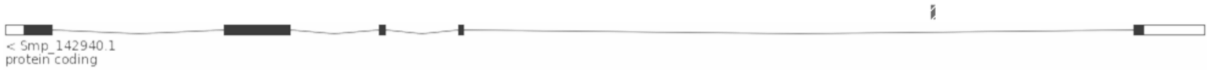

B

```
# Aligned_sequences: 2
# 1: Smp_142940.1_antisense
# 2: sv/PAX2
# Matrix: EDNAFULL
# Gap_penalty: 16
# Extend_penalty: 4
#
# Length: 131
# Identity:      76/131 (58.0%)
# Similarity:    76/131 (58.0%)
# Gaps:          11/131 ( 8.4%)
# Score: 112
#
#
#=======================================

Smp_142940.1_  28071 ACCAAATGAATTTAGAATTCAGTTTAGTTGAAAGTCCTCGAGAATTCCTT  28120
                     |.||||.|.|||||.|||....|||.||||.||..||.|.||.||
sv/PAX2          218 AGCAAAGGTATTTAAAATAGGTTTTTGTTGCAATGCCCCCAGGAT-----    262

Smp_142940.1_  28121 CTAAAAGTCGTTAGAAGA-TGA-GAAAAGGTCATGAATATTTAGTCTAAT  28168
                       |||....|..||.||| ||| ||.|||.|...|..|||..|.||.|..
sv/PAX2          263 --AAAGAATGGAAGGAGACTGAAGACAAGTTGGAGTTTATAAAATCAATG    310

Smp_142940.1_  28169 CAAT--ATTGTACAGAAAATTCCCAATTAGT  28197
                     .|||  ||||.||||..|.||..||.|||.|
sv/PAX2          311 GAATTTATTGCACAGCTACTTGGCATTTAAT    341
```

→ **below the threshold criteria**

9. *Adineta vaga*

9.a Gene GSADVT00030208001

Gene: GSADVG00030208001
Transcript: GSADVT00030208001
Protein: GSADVT00030208001

A

```
# Aligned_sequences: 2
# 1: GSADVT00030208001_sense
# 2: sv/PAX2
# Matrix: EDNAFULL
# Gap_penalty: 16
# Extend_penalty: 4
#
# Length: 157
# Identity:      87/157 (55.4%)
# Similarity:    87/157 (55.4%)
# Gaps:           9/157 ( 5.7%)
# Score: 95
#
#
#=======================================

GSADVT0003020   3262 GAATTGAAAAAAATTCCATATCAAATTTGAAGATATTACTCTTGCCAATGA   3311
                     |||.|.|||||  ||||.||.||||.|..|.|..||..|...|.....||
sv/PAX2          163 GAAGTTAAAAA--TCCAAATAAAATATTGACACTTTTTTGGGGAGGGGGA    210

GSADVT0003020   3312 TCGTGACATCAAAGCA--TTAACATCGGATATCGAACAACTGCGCCAAC-   3358
                     ....|..|.||||||..  ||||.||.||.|.|.|....|.|||.||.|.
sv/PAX2          211 GGTGGGAAGCAAAGGTATTTAAAATAGGTTTTTGTTGCAATGCCCCCAGG    260

GSADVT0003020   3359 AGAAACAATTACAAAATCATGAA---AATTTTGAATTAA-AAACTCAATT   3404
                     |.|||.|||...|..|...||||   ||.||.||.||.| |||.|||||.
sv/PAX2          261 ATAAAGAATGGAAGGAGACTGAAGACAAGTTGGAGTTTATAAAATCAATG    310

GSADVT0003020   3405 AAAACTA   3411
                     .||..||
sv/PAX2          311 GAATTTA    317
```

→ **below the threshold criteria**

B

```
# Aligned_sequences: 2
# 1: GSADVT00030208001_antisense
# 2: sv/PAX2
# Matrix: EDNAFULL
# Gap_penalty: 16
# Extend_penalty: 4
#
# Length: 32
# Identity:      26/32 (81.2%)
# Similarity:    26/32 (81.2%)
# Gaps:           1/32 ( 3.1%)
# Score: 94
#
#
#=======================================

GSADVT0003020   2890 ATTTAAAAAGCCCAAAAAAATATTTGACACAT   2921
                     |.|||||||.||.||.|||||||| |||||.|
sv/PAX2          165 AGTTAAAAATCCAAATAAAATATT-GACACTT    195
```

→ **below the threshold criteria**

9.b Gene GSADVT00000720001

Gene: GSADVG00000720001
Transcript: GSADVT00000720001
Protein: GSADVT00000720001

A

```
# Aligned_sequences: 2
# 1: GSADVT00000720001_sense
# 2: sv/PAX2
# Matrix: EDNAFULL
# Gap_penalty: 16
# Extend_penalty: 4
#
# Length: 25
# Identity:      20/25 (80.0%)
# Similarity:    20/25 (80.0%)
# Gaps:           1/25 ( 4.0%)
# Score: 68
#
#
#=======================================

GSADVT00000720   744 GTCAATCAATTGTTGATTTAGCGAA    768
                     |||||.|||.|||| ||..||||||
sv/PAX2          142 GTCAAACAACTGTT-ATGGAGCGAA    165
```

→ below the threshold criteria

B

```
# Aligned_sequences: 2
# 1: GSADVT00000720001_antisense
# 2: sv/PAX2
# Matrix: EDNAFULL
# Gap_penalty: 16
# Extend_penalty: 4
#
# Length: 40
# Identity:      26/40 (65.0%)
# Similarity:    26/40 (65.0%)
# Gaps:           0/40 ( 0.0%)
# Score: 74
#
#
#=======================================

GSADVT0000072   1524 GTTTTTTTTCTACTCTCAGGAGAATTGAGAATGGACTGAG   1563
                     ||||||.||..|.|..|...||.||..|||||||||..|||
sv/PAX2          238 GTTTTTGTTGCAATGCCCCCAGGATAAAGAATGGAAGGAG    277
```

→ below the threshold criteria

9.c Gene GSADVT00066437001

Gene: GSADVG00066437001
Transcript: GSADVT00066437001
Protein: GSADVT00066437001

A

```
# Aligned_sequences: 2
# 1: GSADVT00066437001_sense
# 2: sv/PAX2
# Matrix: EDNAFULL
# Gap_penalty: 16
# Extend_penalty: 4
#
# Length: 22
# Identity:      19/22 (86.4%)
# Similarity:    19/22 (86.4%)
# Gaps:           0/22 ( 0.0%)
# Score: 83
#
#
#=======================================

GSADVT0006643   1304 AATAGAATAATGACACTTGTTT   1325
                     ||||.||||.||||||||.|||
sv/PAX2          178 AATAAAATATTGACACTTTTTT    199
```

→ below the threshold criteria

B

```
# Aligned_sequences: 2
# 1: GSADVT00066437001_antisense
# 2: sv/PAX2
# Matrix: EDNAFULL
# Gap_penalty: 16
# Extend_penalty: 4
#
# Length: 32
# Identity:      22/32 (68.8%)
# Similarity:    22/32 (68.8%)
# Gaps:           0/32 ( 0.0%)
# Score: 70
#
#
#=======================================

GSADVT000664370   45 TAATAATCTAAATAAAACAAATATTCATCTTT     76
                     |||.||||.||||||||.|...|..|.|.|||
sv/PAX2          168 TAAAAATCCAAATAAAATATTGACACTTTTTT    199
```

→ below the threshold criteria

9.d Gene GSADVT00003964001

Gene: GSADVG00003964001
Transcript: GSADVT00003964001
Protein: GSADVT00003964001

A

```
# Aligned_sequences: 2
# 1: GSADVT00003964001_sense
# 2: sv/PAX2
# Matrix: EDNAFULL
# Gap_penalty: 16
# Extend_penalty: 4
#
# Length: 22
# Identity:      19/22 (86.4%)
# Similarity:    19/22 (86.4%)
# Gaps:           0/22 ( 0.0%)
# Score: 83
#
#
#=======================================

GSADVT0000396   1313 AATAGAATAATGACACTTGTTT   1334
                     ||||.||||.||||||||.|||
sv/PAX2          178 AATAAAATATTGACACTTTTTT    199
```

→ **below the threshold criteria**

B

```
# Aligned_sequences: 2
# 1: GSADVT00003964001_antisense
# 2: sv/PAX2
# Matrix: EDNAFULL
# Gap_penalty: 16
# Extend_penalty: 4
#
# Length: 78
# Identity:      48/78 (61.5%)
# Similarity:    48/78 (61.5%)
# Gaps:           3/78 ( 3.8%)
# Score: 84
#
#
#=======================================

GSADVT0000396   1526 CAGAAGAAAAATATTCAATTTGAA-GAAGAAAAGTTTCGTTTTATACCTT   1574
                     |||.|.|||.| ||..||...||. |||||.|||||....||||||...|
sv/PAX2          257 CAGGATAAAGA-ATGGAAGGAGACTGAAGACAAGTTGGAGTTTATAAAAT    305

GSADVT0000396   1575 CCGAGAATTTTACTTACACAACCATGTG   1602
                     |...|.|.|||| ||.||||.|.|..||
sv/PAX2          306 CAATGGAATTTA-TTGCACAGCTACTTG    332
```

→ **Hit:**

>GSADVT00003964001_antisense_CRE-like
CAGAAGAAAAATATTCAATTTGAAGAAGAAAAGTTTCGTTTTATACCTTCCGAGAATTTTACTTACACAACCATGTG

→ **BLASTN SEARCH** for GSADVT00003964001_antisense_CRE-like: e-value 1e-36

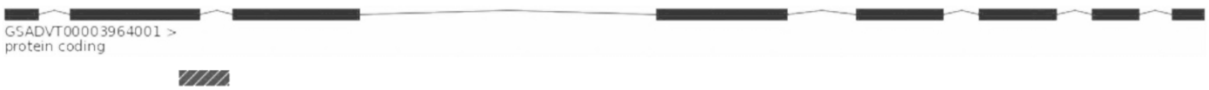

→ **does not meet criteria**: does not fully lie in intronic region

9.e Gene GSADVT00040493001

Gene: GSADVG00040493001
Transcript: GSADVT00040491001
Protein: GSADVT00040491001

A

```
# Aligned_sequences: 2
# 1: GSADVT00040493001_sense
# 2: sv/PAX2
# Matrix: EDNAFULL
# Gap_penalty: 16
# Extend_penalty: 4
#
# Length: 37
# Identity:      28/37 (75.7%)
# Similarity:    28/37 (75.7%)
# Gaps:           2/37 ( 5.4%)
# Score: 92
#
#
#=======================================

GSADVT00040493   588 AGCAACACCAAAAGTAATTAATGCAA--TTACAAATT    622
                     |||||||.||.||||.||||||.||.  |..||||||
sv/PAX2           34 AGCAACAGCACAAGTCATTAATTCACCCTCCCAAATT     70
```

→ below the threshold criteria

B

```
# Aligned_sequences: 2
# 1: GSADVT00040493001_antisense
# 2: sv/PAX2
# Matrix: EDNAFULL
# Gap_penalty: 16
# Extend_penalty: 4
#
# Length: 51
# Identity:      33/51 (64.7%)
# Similarity:    33/51 (64.7%)
# Gaps:           0/51 ( 0.0%)
# Score: 93
#
#
#=======================================

GSADVT00040493   925 TGTTAAACCTTTTTTGGGGAATAGAAAATGAAATAAAAAGAAATTGAAAA    974
                     |.||.|..||||||||||||...|.|..||..|...||||..|||.||||
sv/PAX2          185 TATTGACACTTTTTTGGGGAGGGGGAGGTGGGAAGCAAAGGTATTTAAAA    234

GSADVT00040493   975 T    975
                     |
sv/PAX2          235 T    235
```

→ below the threshold criteria

9.f Gene GSADVT00040499001

Gene: GSADVG00040499001
Transcript: GSADVT00040499001
Protein: GSADVT00040499001

A

```
# Aligned_sequences: 2
# 1: GSADVT00040499001_sense
# 2: sv/PAX2
# Matrix: EDNAFULL
# Gap_penalty: 16
# Extend_penalty: 4
#
# Length: 37
# Identity:      28/37 (75.7%)
# Similarity:    28/37 (75.7%)
# Gaps:           2/37 ( 5.4%)
# Score: 92
#
#
#=======================================

GSADVT00040499   590 AGCAACACCAAAAGTAATTAATGCAA--TTACAAATT    624
                     |||||||.||.||||.||||||.||.  |..||||||
sv/PAX2           34 AGCAACAGCACAAGTCATTAATTCACCCTCCCAAATT     70
```

→ below the threshold criteria

B

```
# Aligned_sequences: 2
# 1: GSADVT00040499001_antisense
# 2: sv/PAX2
# Matrix: EDNAFULL
# Gap_penalty: 16
# Extend_penalty: 4
#
# Length: 51
# Identity:      33/51 (64.7%)
# Similarity:    33/51 (64.7%)
# Gaps:           0/51 ( 0.0%)
# Score: 93
#
#
#=======================================

GSADVT0004049   1715 TGTTAAACCTTTTTTGGGGAATAGAAAATGAAATAAAAAGAAATTGAAAA   1764
                     |.||.|..||||||||||||...|.|..||..|...||||..|||.||||
sv/PAX2          185 TATTGACACTTTTTTGGGGAGGGGGAGGTGGGAAGCAAAGGTATTTAAAA    234

GSADVT0004049   1765 T   1765
                     |
sv/PAX2          235 T    235
```

→ below the threshold criteria

10. Hypsibius exemplaris

Gene: BV898_05868
Transcript: mrna.BV898_05868.2
Product: OQV20075.1

A

```
# Aligned_sequences: 2
# 1: OQV20075.1_sense
# 2: sv_PAX2
# Matrix: EDNAFULL
# Gap_penalty: 16
# Extend_penalty: 4
#
# Length: 140
# Identity:      80/140 (57.1%)
# Similarity:    80/140 (57.1%)
# Gaps:          11/140 ( 7.9%)
# Score: 100
#
#
#=======================================

OQV20075.1_se   2149 AGATTGATTAGTAAAAAAGGCGCTACA-GTCAAATGTATAA--TTAAATA   2195
                     |.||||.|||.|.||.| |..|..||| |.|...||.||||  |...|.|
sv_PAX2           66 AAATTGCTTATTCAATA-GCAGGAACATGGCTCCTGAATAAAGTCCCAGA    114

OQV20075.1_se   2196 ATTTGGATGACTTTT-----TAAGGTGATAAGAAAAAGGGTAAAAAA--A   2238
                     ||||...|||||...     |.|||.|.|.|.|.||..|.||...|.  |
sv_PAX2          115 ATTTATGTGACTCGCACGAGTCAGGAGGTCAAACAACTGTTATGGAGCGA    164

OQV20075.1_se   2239 AATTAAAAATTAAAAAAAAATCTAAAGAATTTTTAAAGGA   2278
                     |.||||||||..|||.|||||.|..|.|.|||||...|||
sv_PAX2          165 AGTTAAAAATCCAAATAAAATATTGACACTTTTTTGGGGA    204
```

→ below the threshold criteria

B

```
# Aligned_sequences: 2
# 1: OQV20075.1_antisense
# 2: sv_PAX2
# Matrix: EDNAFULL
# Gap_penalty: 16
# Extend_penalty: 4
#
# Length: 86
# Identity:      54/86 (62.8%)
# Similarity:    54/86 (62.8%)
# Gaps:          10/86 (11.6%)
# Score: 82
#
#
#=======================================

OQV20075.1_an   5463 GTTAAAATTCCGCTTCAACTAAACACATTGACGCGCTGTTTGTGTCAGGG   5512
                     |||||||.||     |||.|||| |.||||||.|..|.||.|.|...|||
sv_PAX2          166 GTTAAAAATC-----CAAATAAA-ATATTGACACTTTTTTGGGGAGGGGG    209

OQV20075.1_an   5513 TG-TGCGCAGCTGTG--ATTTAATGTAC-TATTTGT   5544
                     .| ||.|.|||...|  ||||||..||. |.|||||
sv_PAX2          210 AGGTGGGAAGCAAAGGTATTTAAAATAGGTTTTTGT    245
```

→ **Hit:**

```
>OQV20075.1_antisense_CRE-like
GTTAAAATTCCGCTTCAACTAAACACATTGACGCGCTGTTTGTGTCAGGGTGTGCGCAGCTGTGATTTAATGTACTATTTGT
```

→ **BLASTN SEARCH** for OQV20075.1_antisense_CRE-like: e-value 5.6e-40

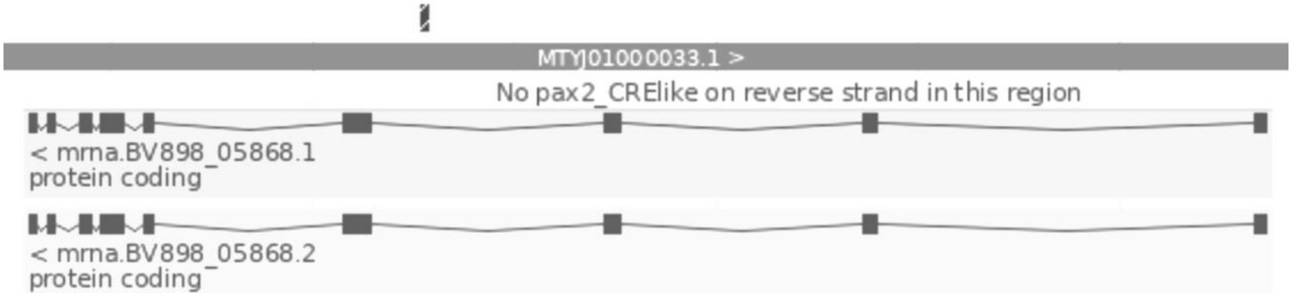

11. *Caenorhabditis elegans*

11.a Gene pax-2 (K06B9.5a.1)

Description: pax-2
Gene: WBGene00003938
Transcript: K06B9.5a.1
Product: K06B9.5a.1

A

```
# Aligned_sequences: 2
# 1: K06B9.5a.1_sense
# 2: sv/PAX2
# Matrix: EDNAFULL
# Gap_penalty: 16
# Extend_penalty: 4
#
# Length: 88
# Identity:      54/88 (61.4%)
# Similarity:    54/88 (61.4%)
# Gaps:           8/88 ( 9.1%)
# Score: 86
#
#
#=======================================

K06B9.5a.1_se   4311 AACAACTACAACAATTCCA-ATTGACTTCTGCAGCAACAGTA-AACTCAC   4358
                     |.|||.|..||.|.|||.. |..||||| |.|||||||||.| ||.|||.
sv/PAX2            3 AGCAATTTTAATATTTCTTCAGGGACTT-TCCAGCAACAGCACAAGTCAT     51

K06B9.5a.1_se   4359 TGATTTCTCGGCCG-----GCTTTTGCCATTCCAGGAA   4391
                     |.|||...|..||.     ||||.|.|.||..||||||
sv/PAX2           52 TAATTCACCCTCCCAAATTGCTTATTCAATAGCAGGAA     89
```

→ **Hit:**
>K06B9.5a.1_sense_CRE-like
AACAACTACAACAATTCCAATTGACTTCTGCAGCAACAGTAAACTCACTGATTTCTCGGCCGGCTTTTGCCATTCCAGGAA

→ **BLASTN SEARCH** for K06B9.5a.1_sense_CRE-like: e-value 2.1e-39

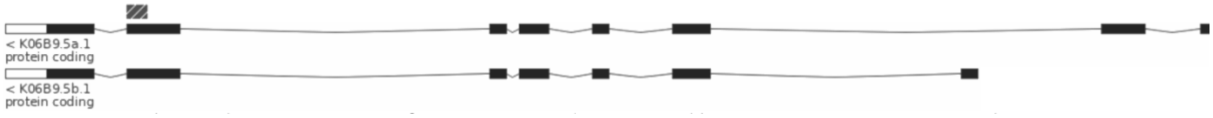

→ **does not meet criteria:** does not fully lie in intronic region

B

```
# Aligned_sequences: 2
# 1: K06B9.5a.1_antisense
# 2: sv/PAX2
# Matrix: EDNAFULL
# Gap_penalty: 16
# Extend_penalty: 4
#
# Length: 107
# Identity:      65/107 (60.7%)
# Similarity:    65/107 (60.7%)
# Gaps:          15/107 (14.0%)
# Score: 97
#
#
#=======================================

K06B9.5a.1_an   1746 GAAAATGTGTATAAAAATATTTGAAGTTCCA----CCACAGTTTAAAGAA   1791
                     |.|||.||.|.|||||....||...|||.||    ||.|||..|||||||
sv/PAX2          219 GCAAAGGTATTTAAAATAGGTTTTTGTTGCAATGCCCCCAGGATAAAGAA    268

K06B9.5a.1_an   1792 AAGGAAGGAAAATG--GAAAACTAGAAG-----AAACCCA---GAATTTT   1831
                     . |||||||.|.||  ||.||.|.|.||     |||..||   ||||||.
sv/PAX2          269 T-GGAAGGAGACTGAAGACAAGTTGGAGTTTATAAAATCAATGGAATTTA    317

K06B9.5a.1_an   1832 TTGTGCA   1838
                     |||..||
sv/PAX2          318 TTGCACA    324
```

→ **Hit:**

>K06B9.5a.1_antisense_CRE-like

GAAAATGTGTATAAAAATATTTGAAGTTCCACCACAGTTTAAAGAAAAGGAAGGAAAATGGAAAACTAGAAGAAACCCAGAATTTTTTGTGCA

→ **BLASTN SEARCH** for K06B9.5a.1_antisense_CRE-like: e-value 1.7e-46

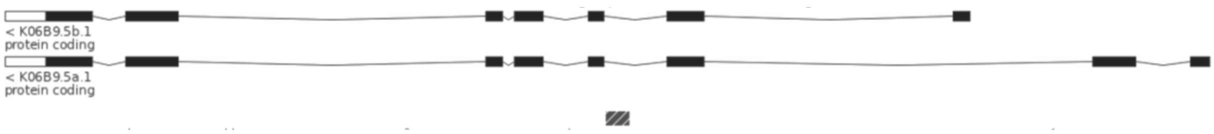

11.b Gene egl-38 (C04G2.7.1)

Description: egl-38
Gene: WBGene00001204
Transcript: C04G2.7.1
Product: C04G2.7.1

A

```
# Aligned_sequences: 2
# 1: C04G2.7.1_sense
# 2: sv/PAX2
# Matrix: EDNAFULL
# Gap_penalty: 16
# Extend_penalty: 4
#
# Length: 228
# Identity:     124/228 (54.4%)
# Similarity:   124/228 (54.4%)
# Gaps:          33/228 (14.5%)
# Score: 96
#
#
#=======================================
C04G2.7.1_sen   2879 ACTTCTGCAGCTACAGTA-AACTCACTAATGACTCCGCC-----TGCTTT   2922
                     |||| |.||||.||||.| ||.|||.||||....||.||     |||||.
sv/PAX2           27 ACTT-TCCAGCAACAGCACAAGTCATTAATTCACCCTCCCAAATTGCTTA     75

C04G2.7.1_sen   2923 CGCAATGCCTGGAAGTTAGTTATTCATATAAAATC-------TTTTTTTA   2965
                     ..||||..|.||||..|.|.|..|.| |||||.||       ||.|.|.|
sv/PAX2           76 TTCAATAGCAGGAACATGGCTCCTGA-ATAAAGTCCCAGAATTTATGTGA    124

C04G2.7.1_sen   2966 CAGAAA--AATCATGCTTTTAACTATTTACTTAAAAGTGAAATTCAGTCA   3013
                     |....|  |.|||.|...|.||..|..|..|....||.|||.||.|
sv/PAX2          125 CTCGCACGAGTCAGGAGGTCAAACAACTGTTATGGAGCGAAGTTAA----    170

C04G2.7.1_sen   3014 CAAATCCGGAGCCTAAAATAAACTATGGA---TTTTTTTGTGAGGCTCAG   3060
                      ||||||        |||||||.|||.||   ||||||.|.||||...||
sv/PAX2          171 -AAATCC--------AAATAAAATATTGACACTTTTTTGGGGAGGGGGAG    211

C04G2.7.1_sen   3061 GATTTGATTGAAGATTTTAACACTAGTT   3088
                     |.....|...|||.|.||.|.|.|||.|
sv/PAX2          212 GTGGGAAGCAAAGGTATTTAAAATAGGT    239
```

→ below the threshold criteria

B

```
# Aligned_sequences: 2
# 1: C04G2.7.1_antisense
# 2: sv/PAX2
# Matrix: EDNAFULL
# Gap_penalty: 16
# Extend_penalty: 4
#
# Length: 95
# Identity:      56/95 (58.9%)
# Similarity:    56/95 (58.9%)
# Gaps:           7/95 ( 7.4%)
# Score: 88
#
#
#=======================================
C04G2.7.1_ant   3403 CATTTTTAATGGAG---ATTTAAAAAAAGAGACCTACCAAATTGAAAATT   3449
                     ||..|.|.||||||   |.|||||||...|.|  ||..|.|||||.|.||
sv/PAX2          148 CAACTGTTATGGAGCGAAGTTAAAAATCCAAA--TAAAATATTGACACTT    195

C04G2.7.1_ant   3450 GTGATGTCCTGGGTAGGTTGGGTAAGGATAATTTCTCCAAATAGG   3494
                     .|...|....|||.||||  ||.|||.|.|..|..|..|||||||
sv/PAX2          196 TTTTGGGGAGGGGGAGGT--GGGAAGCAAAGGTATTTAAAATAGG    238
```

→ below the threshold criteria

12. Priapulus caudatus

Description: paired box protein Pax-5-like
NCBI Gene: 106819112 (LOC106819112)
Transcript: XM_014823776.1
Product: XP_014679262.1

A

```
# Aligned_sequences: 2
# 1: LOC106819112_sense
# 2: sv/PAX2
# Matrix: EDNAFULL
# Gap_penalty: 16
# Extend_penalty: 4
#
# Length: 46
# Identity:      32/46 (69.6%)
# Similarity:    32/46 (69.6%)
# Gaps:           4/46 ( 8.7%)
# Score: 80
#
#
#=======================================

LOC106819112_    9371 GCGGAATTAAAATTTCACAACGTAAAATGAAGGAAACGTTTTTGAG   9416
                      |||.|.||||||.|.||.|   |||||| |..||.||.||||||.|
sv/PAX2           161 GCGAAGTTAAAAATCCAAA---TAAAAT-ATTGACACTTTTTTGGG    202
```

→ below the threshold criteria

B

```
# Aligned_sequences: 2
# 1: LOC106819112_antisense
# 2: sv/PAX2
# Matrix: EDNAFULL
# Gap_penalty: 16
# Extend_penalty: 4
#
# Length: 23
# Identity:      20/23 (87.0%)
# Similarity:    20/23 (87.0%)
# Gaps:           1/23 ( 4.3%)
# Score: 76
#
#
#=======================================

LOC106819112_    2568 GGGGAGGGGGCAGGAGGGAAGAA   2590
                      |||||||||| |||.||||||.|
sv/PAX2           200 GGGGAGGGGG-AGGTGGGAAGCA    221
```

→ below the threshold criteria

sv/PAX2 CRE search in non-neural genes

Shown are the results of EMBOSS matcher sequence comparisons between the superconserved *shaven/PAX2* CRE identified by ref. 8 and the non-neural genes *Brachyury* (1), *GATA* (2) and *Twist* (3) for key species. A match meeting the cutoff criteria of 60% sequence identity and 55bp length was blasted using Ensembl Blastn to identify its e-value (cutoff: 1e-1) and its position (criteria: intronic position).

1. *Brachyury*

1.1 Nematostella vectensis

```
Gene-ID: 116616126

# Aligned_sequences: 2
# 1: sv_PAX2
# 2: 1015147-1021296
# Matrix: EDNAFULL
# Gap_penalty: 16
# Extend_penalty: 4
#
# Length: 281
# Identity:     151/281 (53.7%)
# Similarity:   151/281 (53.7%)
# Gaps:          24/281 ( 8.5%)
# Score: 91
#
#
#=======================================

sv_PAX2           58 ACCCTCCCAAATTGCTTATTCAATAGCAGGAACATGGCTCCTGAATAAAG    107
                     |||..|||||||  |.|.||.||..||.|.||.|  |||..||.|..|||
1015147-10212   4998 ACCTACCCAAAT--CATTTTAAAGTGCTGAAAGA--GCTTATGGACTAAG   5043

sv_PAX2          108 TCCCAGAATTTATGTGACTCGCACGAGTCAGGAGGTCAA-ACAACTGTTA    156
                     .   |.||...|.|.|..|...|.|..||..|||.|.|. |.|||| ||.
1015147-10212   5044 G---AAAAACGAGGGGCTTTATAGGTATCTAGAGCTTATCATAACT-TTC   5089

sv_PAX2          157 TGGAGCGAAGTTAAAAATCCAAATAAAATATTGACACTTTTTTGGGGAGG    206
                     ||...|..||||.||.|.  |..||..||||.|||||.|.|.....|...
1015147-10212   5090 TGTTCCCTAGTTGAATAA--ATTTACTATATCGACACGTATCGTTAGCCC   5137

sv_PAX2          207 GGGAGGTGGGAAGCA-AAGGTATTTAAAATAGGTTTTTGTTGCAATGCCC    255
                     ...||....|||||. ||..||.||.  .||||||||..||..||   ||
1015147-10212   5138 ATTAGACAAGAAGCTCAATATAGTTT--CTAGGTTTTACTTTGAA---CC   5182

sv_PAX2          256 CCAGGATAAAGAATGGAAGGAGACTGAAG--ACAAGTTGGAGTT-TATAA    302
                     |..|.|.|.|..||..||..||..|.||.  ||||....||||| |||||
1015147-10212   5183 CTTGAAAACATTATCAAACAAGCTTCAAACAACAAAAAAGAGTTGTATAA   5232

sv_PAX2          303 AATCAATGGAATT----TATTGCACAGCTAC    329
                     .||.|....||||    ||||.||..|.|||
1015147-10212   5233 CATGATGTTAATTCCACTATTTCAATGTTAC   5263
```

→ below the threshold criteria

1.2 Saccoglossus kowalevskii

```
Gene-ID: 100303461


# Aligned_sequences: 2
# 1: sv_PAX2
# 2: c349884-344800
# Matrix: EDNAFULL
# Gap_penalty: 16
# Extend_penalty: 4
#
# Length: 59
# Identity:      38/59 (64.4%)
# Similarity:    38/59 (64.4%)
# Gaps:           4/59 ( 6.8%)
# Score: 82
#
#
#=======================================

sv_PAX2          264 AAGAATGGAAGGAGACTGAAGACAAGTTGGAGTTTATAAAATCAATGGAA    313
                     |||||.||||||..|.|.|..|.||..|.|  |||.||||||.||  ||.
c349884-34480    256 AAGAACGGAAGGTAAATAATCAAAAGTCG--TTTTTAAAATTAA--GAT    301

sv_PAX2          314 TTTATTGCA    322
                     ||.|.||.|
c349884-34480    302 TTAAATGTA    310
```

→ **does not meet criteria:** does not lie in intronic region

1.3 Ciona intestinalis

```
Gene-ID: 778911

# Aligned_sequences: 2
# 1: sv_PAX2
# 2: 6264-8022
# Matrix: EDNAFULL
# Gap_penalty: 16
# Extend_penalty: 4
#
# Length: 28
# Identity:      20/28 (71.4%)
# Similarity:    20/28 (71.4%)
# Gaps:           0/28 ( 0.0%)
# Score: 68
#
#
#=======================================

sv_PAX2          171 AAATCCAAATAAAATATTGACACTTTTT    198
                     ||||...|.||||||||..||||.|.||
6264-8022       1731 AAATAACATTAAAATATGAACACATGTT   1758
```

→ **below the threshold criteria**

1.4 Lingula anatina

```
Gene-ID: 106155248

# Aligned_sequences: 2
# 1: sv_PAX2
# 2: 1426532-1435256
# Matrix: EDNAFULL
# Gap_penalty: 16
# Extend_penalty: 4
#
# Length: 44
# Identity:      31/44 (70.5%)
# Similarity:    31/44 (70.5%)
# Gaps:           3/44 ( 6.8%)
# Score: 91
#
#
#=======================================

sv_PAX2           88 AACATGGCTCCTGAATAAAG---TCCCAGAATTTATGTGACTCG    128
                     ||||..|||..||||.||.|   |...|||||||||||||.|||
1426532-14352   7024 AACAAAGCTTATGAAAAACGGATTTGTAGAATTTATGTGATTCG   7067
```

→ **below the threshold criteria**

1.5 Octopus bimaculoides

```
Gene-ID: 106883344

# Aligned_sequences: 2
# 1: sv_PAX2
# 2: 25437-45149
# Matrix: EDNAFULL
# Gap_penalty: 16
# Extend_penalty: 4
#
# Length: 86
# Identity:      53/86 (61.6%)
# Similarity:    53/86 (61.6%)
# Gaps:           4/86 ( 4.7%)
# Score: 97
#
#
#=======================================

sv_PAX2          271 GAAGGAGACTGAAGACAAGTTGGAGTTTATAAAATCAATGGAATTT-ATT    319
                     ||.|||.|.|.||.| |||.|.|||||.|||.||...|||.|...| |||
25437-45149     4581 GAGGGAAAGTAAAAA-AAGATAGAGTTAATAGAAAAGATGCAGGATGATT   4629

sv_PAX2          320 GCACAGCTACTTGGCATTTAATAATTCTCTCATTTC    355
                     |||||  |||..|||.|...|.||..|..|.|||.|
25437-45149     4630 GCACA--TACGAGGCTTGGTAGAAGGCAATGATTAC   4663
```

→ **Hit:** fully lies in intronic region

1.6 Schistosoma mansoni

```
Gene-ID: 4609

# Aligned_sequences: 2
# 1: sv_PAX2
# 2: 127735434-127742951
# Matrix: EDNAFULL
# Gap_penalty: 16
# Extend_penalty: 4
#
# Length: 138
# Identity:      81/138 (58.7%)
# Similarity:    81/138 (58.7%)
# Gaps:          13/138 ( 9.4%)
# Score: 93
#
#
#=======================================

sv_PAX2          219 GCAAAGGTATTT---AAAATAGGTTTTTGTTGCAATGCCCCCAGGATAAA    265
                     |.||||||||||   ||.||..||.||..||..|| |||.|  .||.||.
127735434-127   7306 GAAAAGGTATTTTTGAACATGTGTATTAATTATAA-GCCTC--TGAAAAC   7352

sv_PAX2          266 GAATGGAAGGAGACTGAAGACAAGTTGGAGTTTATA---AAATCAATGGA    312
                     ..||||....|..|.|||...|.|||| |.|.||||   ||||.|| |||
127735434-127   7353 CTATGGCCCAAACCAGAAATGATGTTG-ATTATATAGGTAAATGAA-GGA   7400

sv_PAX2          313 ATTTATTGCACAGCTACTTGGC--ATTTAATAATTCTC    348
                     ...||||||....|||.||..|  |||...|.|.||||
127735434-127   7401 TGCTATTGCTGTTCTAATTACCTCATTGTCTCAGTCTC   7438
```

→ below the threshold criteria

1.7 Adineta vaga

```
Gene-ID: GSADVG00063359001

# Aligned_sequences: 2
# 1: sv_PAX2
# 2: HG381423
# Matrix: EDNAFULL
# Gap_penalty: 16
# Extend_penalty: 4
#
# Length: 162
# Identity:      91/162 (56.2%)
# Similarity:    91/162 (56.2%)
# Gaps:          26/162 (16.0%)
# Score: 87
#
#
#=======================================

sv_PAX2           57 CACCCTCCCAAATTGCTTATTCAATA------GCAGGAACATGGCTCCTG    100
                     ||||.|..|||..|.||.|||||..|      |..|.|||||||.|.|..
HG381423         924 CACCTTATCAACATCCTGATTCACCAAATTATGGTGAAACATGGATGCGT    973

sv_PAX2          101 AATAAAGTC-------CCAGAATTTATGTGAC---TCGCACGAGTCAGG-    139
                     .|||.|||.       |||.|.||.|..||||   |...||.|.|||.. 
HG381423         974 GATATAGTTTCATTTTCCAAAGTTAAATTGACAAATAAAACAAATCAAAC   1023

sv_PAX2          140 AGGTCAAACAACTGTTATGGAGCGAAGTTAAAAATCCAAATAAAATA---    186
                     |||||.|     |||.|.|.|. |||.|||||.||..|||.|||||.   
HG381423        1024 AGGTCCA-----TGTCAAGTAA-GAAATTAAACATTAAAAAAAAATGATT   1067

sv_PAX2          187 TTGACACTTTTT    198
                     ||||.|||.|.|
HG381423        1068 TTGAAACTATAT   1079
```

→ below the threshold criteria

1.8 Hypsibius exemplaris

```
Gene-ID: BV898_01477

# Aligned_sequences: 2
# 1: sv_PAX2
# 2: MTYJ01000005.1
# Matrix: EDNAFULL
# Gap_penalty: 16
# Extend_penalty: 4
#
# Length: 39
# Identity:      29/39 (74.4%)
# Similarity:    29/39 (74.4%)
# Gaps:           2/39 ( 5.1%)
# Score: 81
#
#
#=======================================

sv_PAX2           26 GACTTTCCAGCA-ACAGCACAAGTCATTAATTCACCCTC     63
                     |||||||.|||| |||| |||||....|||||.||..||
MTYJ01000005.   1262 GACTTTCAAGCATACAG-ACAAGAGCATAATTAACTGTC   1299
```

→ below the threshold criteria

1.9 Priapulus caudatus

```
Gene-ID: 106811534

# Aligned_sequences: 2
# 1: sv_PAX2
# 2: 79967-89147
# Matrix: EDNAFULL
# Gap_penalty: 16
# Extend_penalty: 4
#
# Length: 39
# Identity:      27/39 (69.2%)
# Similarity:    27/39 (69.2%)
# Gaps:           0/39 ( 0.0%)
# Score: 87
#
#
#=======================================

sv_PAX2          211 GGTGGGAAGCAAAGGTATTTAAAATAGGTTTTTGTTGCA    249
                     ||..||||||.|..||.|||.|.||.|.|.|||.|||||
79967-89147     5921 GGACGGAAGCGATTGTTTTTTATATCGTTGTTTTTTGCA   5959
```

→ below the threshold criteria

2. *GATA*

2.1 Nematostella vectensis

```
Gene-ID: 5521983

# Aligned_sequences: 2
# 1: sv_PAX2
# 2: NEMVEscaffold_2
# Matrix: EDNAFULL
# Gap_penalty: 16
# Extend_penalty: 4
#
# Length: 143
# Identity:      80/143 (55.9%)
# Similarity:    80/143 (55.9%)
# Gaps:          16/143 (11.2%)
# Score: 76
#
#
#=======================================

sv_PAX2          115 ATTTATGTGACTCGCACGAGTCAGGAGGTCAAA-CAACTGTTATGGAGCG    163
                     |||..|.|||.|.||||.|..||||||..|... ||||.||||..||.|.
NEMVEscaffold   1241 ATTAGTCTGAATAGCACAAAACAGGAGCGCGCCGCAACAGTTACAGATCT   1290

sv_PAX2          164 A----AGTTAAAAATCCAAAT------AAAATATTGACACTTTTTTGGGG    203
                     |    |.||.|.|.|.||.|.      ||.|.|||.|.| ||||||....
NEMVEscaffold   1291 ACCAGACTTTAGACTTCACACGGCTCAAACAAATTTATA-TTTTTTCATT   1339

sv_PAX2          204 AGGGGGAGGTGGGAAGCAAAGGTATTTAAAATAG--GTTTTTG    244
                     |||.|.|  |.||||.....||...||..|||||  ||||.||
NEMVEscaffold   1340 AGGAGTA--TAGGAATAGCGGGCTGTTGTAATAGAAGTTTATG   1380
```

→ **below the threshold criteria**

2.2 Saccoglossus kowalevskii

```
Gene-ID: 102809317

# Aligned_sequences: 2
# 1: sv_PAX2
# 2: ACQM01128928.1
# Matrix: EDNAFULL
# Gap_penalty: 16
# Extend_penalty: 4
#
# Length: 75
# Identity:      45/75 (60.0%)
# Similarity:    45/75 (60.0%)
# Gaps:           8/75 (10.7%)
# Score: 69
#
#
#=======================================

sv_PAX2            4 GCAATTTTAATA----TTTCTTCAGGGACT--TTCCAGCAACA--GCACA     45
                     |.||.|.|||||    |.|||.||...|||  |.|||.|||||  ||||.
ACQM01128928.    739 GAAAATATAATAACCGTGTCTGCACACACTGCTACCATCAACAATGCACC    788

sv_PAX2           46 AGTCATTAATTCACCCTCCCAAATT     70
                     |..|..|...||||||...||||.|
ACQM01128928.    789 AAGCTGTGTATCACCCGATCAAAAT    813
```

→ **does not meet criteria:** does not lie in intronic region

2.3 Ciona intestinalis

Gene-ID: 779004

```
# Aligned_sequences: 2
# 1: sv_PAX2
# 2: HT001157.1
# Matrix: EDNAFULL
# Gap_penalty: 16
# Extend_penalty: 4
#
# Length: 42
# Identity:      28/42 (66.7%)
# Similarity:    28/42 (66.7%)
# Gaps:           1/42 ( 2.4%)
# Score: 72
#
#
#=======================================

sv_PAX2           23 AGGGACTTTCCAGCAACAGCACAAGTCATTAATTCACCCTCC     64
                     ||.||.||..||||||.| ||..|||.|||||...||.||.|
HT001157.1      3807 AGAGAATTAGCAGCAAAA-CAACAGTTATTAAAATACGCTAC   3847
```

→ below the threshold criteria

2.4 Lingula anatina

Gene-ID: 106172844

```
# Aligned_sequences: 2
# 1: sv_PAX2
# 2: LFEI02000418.1
# Matrix: EDNAFULL
# Gap_penalty: 16
# Extend_penalty: 4
#
# Length: 27
# Identity:      23/27 (85.2%)
# Similarity:    23/27 (85.2%)
# Gaps:           0/27 ( 0.0%)
# Score: 99
#
#
#=======================================

sv_PAX2          164 AAGTTAAAAATCCAAATAAAATATTGA    190
                     ||..|.||||||.||||||||||||||
LFEI02000418.   5945 AAACTTAAAATCGAAATAAAATATTGA   5971
```

→ below the threshold criteria

2.5 Octopus bimaculoides

```
Gene-ID: 106868722

# Aligned_sequences: 2
# 1: sv_PAX2
# 2: KQ417342.1
# Matrix: EDNAFULL
# Gap_penalty: 16
# Extend_penalty: 4
#
# Length: 100
# Identity:      59/100 (59.0%)
# Similarity:    59/100 (59.0%)
# Gaps:           4/100 ( 4.0%)
# Score: 107
#
#
#=======================================

sv_PAX2          242 TTGTTGCAATGCCCCCAGGATAAAGAATGGAAGGAGACTGAAGACAAGTT    291
                     |.|.|.|||||..|.|....|...||..|.||.|.| ||.|.|||||||.
KQ417342.1     18045 TCGCTACAATGATCTCCATTTCGGGATCGTAATGTG-CTAACGACAAGTC  18093

sv_PAX2          292 GGAGTTTATAAAATCAATGGAATTTATTGCACAGCTACTTGGCATTTAAT    341
                     .|||.|||.||..|...|   ||||.||..|.||..|.|||||.||||||
KQ417342.1     18094 TGAGATTAGAATTTTTTT---ATTTCTTCAAAAGTGATTTGGCGTTTAAT  18140
```

→ below the threshold criteria

2.6 Schistosoma mansoni

```
Gene-ID: 8354299

# Aligned_sequences: 2
# 1: sv_PAX2
# 2: SM_V7_1
# Matrix: EDNAFULL
# Gap_penalty: 16
# Extend_penalty: 4
#
# Length: 33
# Identity:      25/33 (75.8%)
# Similarity:    25/33 (75.8%)
# Gaps:           0/33 ( 0.0%)
# Score: 93
#
#
#=======================================

sv_PAX2          167 TTAAAAATCCAAATAAAATATTGACACTTTTTT    199
                     |||||||...|||.||||.|...||||||||||
SM_V7_1        38623 TTAAAAAAATAAACAAAACAGCAACACTTTTTT  38655
```

→ below the threshold criteria

2.7 Adineta vaga

```
Gene-ID: GSADVG00055891001

# Aligned_sequences: 2
# 1: sv_PAX2
# 2: HG381205
# Matrix: EDNAFULL
# Gap_penalty: 16
# Extend_penalty: 4
#
# Length: 112
# Identity:      64/112 (57.1%)
# Similarity:    64/112 (57.1%)
# Gaps:          11/112 ( 9.8%)
# Score: 80
#
#
#=======================================

sv_PAX2          239 TTTTTGTTGCAATGCCCCCAGGATAAAGAATGGAAGGAGACTGAAGACAA    288
                     ||||..||.||..|||   |.||..||.||.|.||.||||...|.||.|.
HG381205        2929 TTTTAATTCCATAGCC---AAGAAGAAAAAAGAAACGAGAAGAATGATAT   2975

sv_PAX2          289 GTTGGAGTTTATAAAATCAATGGAATTTATTGCACAGCTACTTGGCATTT    338
                     .|.     |.||.||||  |||.||..|.|||.||| ||.|.||.|..||
HG381205        2976 CTA-----TCATTAAAT--ATGCAAGCTGTTGGACA-CTTCATGCCTCTT   3017

sv_PAX2          339 AATAATTCTCTC    350
                     ..|...||||||
HG381205        3018 TCTCTCTCTCTC   3029
```

→ **below the threshold criteria**

2.8 Hypsibius exemplaris

```
Gene-ID: BV898_01252

# Aligned_sequences: 2
# 1: sv_PAX2
# 2: MTYJ01000004.1
# Matrix: EDNAFULL
# Gap_penalty: 16
# Extend_penalty: 4
#
# Length: 74
# Identity:      43/74 (58.1%)
# Similarity:    43/74 (58.1%)
# Gaps:           2/74 ( 2.7%)
# Score: 79
#
#
#=======================================

sv_PAX2          229 TTAAAATAGGTTTTTGTTGCAATGCCCCCAGGATAAAGAA--TGGAAGGA    276
                     |.|||.|.||..|.||.|||.|.|||||||..||.....|  |.||.|..
MTYJ01000004.   4881 TCAAAGTGGGCGTCTGCTGCCAGGCCCCCACTATTTCCTAATTAGAGGCT   4930

sv_PAX2          277 GACTGAAGACAAGTTGGAGTTTAT    300
                     ..||.||||.||.|....||||||
MTYJ01000004.   4931 TGCTAAAGATAATTGCCGGTTTAT   4954
```

→ **below the threshold criteria**

2.9 Priapulus caudatus

Gene-ID: 106819002

```
# Aligned_sequences: 2
# 1: sv_PAX2
# 2: KQ717601.1
# Matrix: EDNAFULL
# Gap_penalty: 16
# Extend_penalty: 4
#
# Length: 38
# Identity:      27/38 (71.1%)
# Similarity:    27/38 (71.1%)
# Gaps:           1/38 ( 2.6%)
# Score: 79
#
#
#=======================================

sv_PAX2          258 AGGATAAAGAATGGAAGGAGACTGAAGACAAGTTGGAG    295
                     |||.|||||||.|.||||.||...|||. ||||..|||
KQ717601.1      5548 AGGGTAAAGAAAGAAAGGGGAAAAAAGT-AAGTGAGAG   5584
```

→ **below the threshold criteria**

3. *Twist*

3.1 Nematostella vectensis

Gene-ID: 5514773

```
# Aligned_sequences: 2
# 1: sv_PAX2
# 2: NEMVEscaffold_53
# Matrix: EDNAFULL
# Gap_penalty: 16
# Extend_penalty: 4
#
# Length: 50
# Identity:      33/50 (66.0%)
# Similarity:    33/50 (66.0%)
# Gaps:           8/50 (16.0%)
# Score: 73
#
#
#=======================================

sv_PAX2          273 AGGAGACTGAAGACAAG----TTGGAGTTTATAAAATCAATGGAATTTAT    318
                     ||.||.|||||||..||    ||.|||.|.|.||    |||||.||||||
NEMVEscaffold   1106 AGCAGTCTGAAGATGAGCGGATTCGAGGTCAGAA----AATGGGATTTAT   1151
```

→ below the threshold criteria

3.2 Saccoglossus kowalevskii

Gene-ID: 100303576

```
# Aligned_sequences: 2
# 1: sv_PAX2
# 2: GL016116.1
# Matrix: EDNAFULL
# Gap_penalty: 16
# Extend_penalty: 4
#
# Length: 35
# Identity:      25/35 (71.4%)
# Similarity:    25/35 (71.4%)
# Gaps:           2/35 ( 5.7%)
# Score: 73
#
#
#=======================================

sv_PAX2           66 AAATTGCTTATTCAATAGCAGGAACATGGCTCCTG    100
                     ||||||.||...||||.||..|||.|||  |||||
GL016116.1      1305 AAATTGATTTGACAATGGCCAGAAAATG--TCCTG   1337
```

→ below the threshold criteria

3.3 Ciona intestinalis

```
Gene-ID: 448998

# Aligned_sequences: 2
# 1: sv_PAX2
# 2: c2028690-2027376
# Matrix: EDNAFULL
# Gap_penalty: 16
# Extend_penalty: 4
#
# Length: 153
# Identity:      88/153 (57.5%)
# Similarity:    88/153 (57.5%)
# Gaps:          15/153 ( 9.8%)
# Score: 84
#
#
#=======================================

sv_PAX2           53 AATTCACCCTCCCAAATTGCTTATTCAATAGCAGGAACATGGCTCCTGAA    102
                     |.||||.|||.||  ||||.|.|.|||..|.|||||...|. |.|||.|.
c2028690-2027    672 AGTTCAGCCTTCC--ATTGATGAATCACAATCAGGATTGTT-CACCTCAG    718

sv_PAX2          103 TAAAGTCCCAGAATTTATGTGACTCGCACGAGTCAGGAGGTCAAACAACT    152
                     |.|.|||.|.|.|...|.|..|   ||..|..|...||.||||.|.|. |
c2028690-2027    719 TTACGTCACTGGAACGAAGAAA---GCCAGTATTGTGACGTCACATAT-T    764

sv_PAX2          153 GTTATGGAGCGA----AGTTAAAAATCCAAATAAAATATTGAC--ACTTT    196
                     .||..|.|.|.|    |.||.||||||||.| |||.||| |||  |||.|
c2028690-2027    765 CTTTGGAAACAACTTCAATTCAAAATCCACA-AAACTAT-GACCAACTCT    812

sv_PAX2          197 TTT    199
                     .||
c2028690-2027    813 ATT    815
```

→ below the threshold criteria

3.4 Lingula anatina

```
Gene-ID: 106181138

# Aligned_sequences: 2
# 1: sv_PAX2
# 2: LFEI02000007.1
# Matrix: EDNAFULL
# Gap_penalty: 16
# Extend_penalty: 4
#
# Length: 38
# Identity:      27/38 (71.1%)
# Similarity:    27/38 (71.1%)
# Gaps:           0/38 ( 0.0%)
# Score: 91
#
#
#=======================================

sv_PAX2          160 AGCGAAGTTAAAAATCCAAATAAAATATTGACACTTTT    197
                     |.|.|||.||||||||..|.|||||.|.||.||..||||
LFEI02000007.   9128 ATCTAAGATAAAAATTTACATAAATTTTTAACGATTTT   9165
```

→ below the threshold criteria

3.5 Octopus bimaculoides

Gene-ID: 106871027

```
# Aligned_sequences: 2
# 1: sv_PAX2
# 2: KQ418303.1
# Matrix: EDNAFULL
# Gap_penalty: 16
# Extend_penalty: 4
#
# Length: 51
# Identity:      35/51 (68.6%)
# Similarity:    35/51 (68.6%)
# Gaps:           3/51 ( 5.9%)
# Score: 99
#
#
#=======================================

sv_PAX2          154 TTATGGAGCGAAGTTAAAAATCCAAATAAAATATTGACACTTTTTTGGGG    203
                     |.|||.|..||..||||||||.||..|||.|   |||||||||||...||
KQ418303.1      2007 TAATGTACAGATTTTAAAAATGCATTTAATA---TGACACTTTTTAATGG   2053

sv_PAX2          204 A    204
                     |
KQ418303.1      2054 A   2054
```

→ below the threshold criteria

3.6 Schistosoma mansoni

Gene-ID: 8354110

```
# Aligned_sequences: 2
# 1: sv_PAX2
# 2: c29125361-29124846
# Matrix: EDNAFULL
# Gap_penalty: 16
# Extend_penalty: 4
#
# Length: 33
# Identity:      24/33 (72.7%)
# Similarity:    24/33 (72.7%)
# Gaps:           1/33 ( 3.0%)
# Score: 72
#
#
#=======================================

sv_PAX2           88 AACATGGCTCCTGAATAAAGTCCCAGAATTTAT    120
                     |||||..||..|||||.|.||.| |||||.|||
c29125361-291    376 AACATAACTAATGAATCATGTTC-AGAATATAT    407
```

→ below the threshold criteria

3.7 Adineta vaga

```
Gene-ID: GSADVG00018249001

# Aligned_sequences: 2
# 1: sv_PAX2
# 2: HG380836
# Matrix: EDNAFULL
# Gap_penalty: 16
# Extend_penalty: 4
#
# Length: 44
# Identity:      29/44 (65.9%)
# Similarity:    29/44 (65.9%)
# Gaps:           1/44 ( 2.3%)
# Score: 73
#
#
#=======================================

sv_PAX2          266 GAATGGAAGGAGACTGAAGA-CAAGTTGGAGTTTATAAAATCAA    308
                     ||||||||||.|..||...| |||.|.|.|.|..||.||||.||
HG380836        1085 GAATGGAAGGTGCTTGGTCAACAAATGGTAATACATCAAATGAA   1128
```

→ below the threshold criteria

3.8 Hypsibius exemplaris

```
Gene-ID: BV898_04819

# Aligned_sequences: 2
# 1: sv_PAX2
# 2: MTYJ01000024.1
# Matrix: EDNAFULL
# Gap_penalty: 16
# Extend_penalty: 4
#
# Length: 33
# Identity:      25/33 (75.8%)
# Similarity:    25/33 (75.8%)
# Gaps:           2/33 ( 6.1%)
# Score: 69
#
#
#=======================================

sv_PAX2          169 AAAAATCCA-AATAAAATATTGA-CACTTTTTT    199
                     ||||||||. ||.|||||.||.| |||..||||
MTYJ01000024.   2557 AAAAATCCTCAACAAAATTTTCAACACAATTTT   2589
```

→ below the threshold criteria

3.9 Priapulus caudatus

```
Gene-ID: 106807790

# Aligned_sequences: 2
# 1: sv_PAX2
# 2: KQ715116.1
# Matrix: EDNAFULL
# Gap_penalty: 16
# Extend_penalty: 4
#
# Length: 61
# Identity:      40/61 (65.6%)
# Similarity:    40/61 (65.6%)
# Gaps:           6/61 ( 9.8%)
# Score: 68
#
#
#=======================================

sv_PAX2          195 TTTTTGGGGAGGGGGAGGTGG-GAAGCAAAGGTATTTAAAATAGGTTTTT    243
                     |||||.||.|...| ||.||| ||||.   ||..|||||.|...|||.||
KQ715116.1      4801 TTTTTCGGCAAAAG-AGTTGGTGAAGT---GGATTTTAATACTCGTTATT   4846

sv_PAX2          244 GT-TGCAATGC    253
                     || |||.||||
KQ715116.1      4847 GTATGCGATGC   4857
```

→ **does not meet criteria:** does not lie in intronic region

Supplementary Data Set S4

dachshund/DACH1

Shown are the results of EMBOSS matcher sequence comparisons between the superconserved *dachshund/DACH1* CRE identified in ref. 8 and the respective *dachshund/DACH1* homolog of each species. A match meeting the Cutoff criteria of 60% sequence identity and 55bp length was blasted using Ensembl Blastn to identify its e-value (cutoff: 1e-1) and its position (criteria: intronic position). Each search was carried out with A) the sense and B) the antisense strand of the respective gene.

1. Strongylocentrotus purpuratus

1.a Gene LOC115918252

Description: dachshund homolog 1 like
Gene: LOC115918252
Transcript: XM_030981289
Product: XP_030837149

A

```
# Aligned_sequences: 2
# 1: LOC115918252_sense
# 2: dac/DACH1
# Matrix: EDNAFULL
# Gap_penalty: 16
# Extend_penalty: 4
#
# Length: 22
# Identity:      17/22 (77.3%)
# Similarity:    17/22 (77.3%)
# Gaps:           1/22 ( 4.5%)
# Score: 53
#
#
#=======================================

LOC115918252_    142 AATTGCCAGTTTCAACATTAAT    163
                     ||||.|| |||||.|..|||||
dac/DACH1         33 AATTTCC-GTTTCGAGTTTAAT     53
```

→ below the threshold criteria

B

```
# Aligned_sequences: 2
# 1: LOC115918252_antisense
# 2: dac/DACH1
# Matrix: EDNAFULL
# Gap_penalty: 16
# Extend_penalty: 4
#
# Length: 18
# Identity:      15/18 (83.3%)
# Similarity:    15/18 (83.3%)
# Gaps:           2/18 (11.1%)
# Score: 51
#
#
#=======================================

LOC115918252_    272 TCGATATTCGATGAGTTT    289
                     ||||||  |||||.||||
dac/DACH1        100 TCGATA--CGATGCGTTT    115
```

→ **below the threshold criteria**

1.b Gene LOC586986

Description: Dachshund homolog 1, transcript variant X8
Gene: LOC586986
Transcript: XM_030980740
Product: XP_030836600

A

```
# Aligned_sequences: 2
# 1: LOC586986_sense
# 2: dac/DACH1
# Matrix: EDNAFULL
# Gap_penalty: 16
# Extend_penalty: 4
#
# Length: 137
# Identity:      75/137 (54.7%)
# Similarity:    75/137 (54.7%)
# Gaps:          13/137 ( 9.5%)
# Score: 103
#
#
#=======================================

LOC586986_sen  24437 ATTCTATTCATGTGTGGATTGCTTGAATAGATCATGTTCTTTTACAAGAA  24486
                     ||.|..|||.|.|.|...||..||.||||.||.||       .||.|.||
dac/DACH1        108 ATGCGTTTCTTTTTTTCCTTCGTTAAATAAATAAT-------AACCAAAA    150

LOC586986_sen  24487 ACAGTAAAAATGAAGTAATGGGGAGAGAACAATTATTTTTTTCTCCAGAT  24536
                     |.|..|||||..||..|.|.||..|||||.|.|||||      .|||.|.
dac/DACH1        151 AAAAAAAAAACCAAAAAGTAGGAGGAGAAAAGTTATT------GCCATAG    194

LOC586986_sen  24537 TAGCGTCTTTACAAATGTGTTTTGAAGTGTCTTGTAG  24573
                     |....|..|||.|..|||||.||.|..|.|||.||.|
dac/DACH1        195 TTTTTTTATTATACTTGTGTGTTTACCTTTCTGGTGG    231
```

→ **below the threshold criteria**

B

```
# Aligned_sequences: 2
# 1: LOC586986_antisense
# 2: dac/DACH1
# Matrix: EDNAFULL
# Gap_penalty: 16
# Extend_penalty: 4
#
# Length: 178
# Identity:     103/178 (57.9%)
# Similarity:   103/178 (57.9%)
# Gaps:          10/178 ( 5.6%)
# Score: 143
#
#
#=======================================

LOC586986_ant  26074 TAATGACCAAGTATTATTATTTCTCAAATGTTT-AATAGAAAGGATA--A  26120
                     ||||.|.|||.|..|.||...|||.|||..|.| ||..|.|..||||  |
dac/DACH1         59 TAATAAACAAATTCTTTTCGCTCTAAAAACTCTCAAGTGTATCGATACGA    108

LOC586986_ant  26121 TTCTCTTCACTTTTTTTTTCTTCTGATTATATACACGAAGGACAGAGCAA  26170
                     |.|..|||   |||||||.||||.  |||.|||.|..|....||.|..||
dac/DACH1        109 TGCGTTTC---TTTTTTTCCTTCG--TTAAATAAATAATAACCAAAAAAA    153

LOC586986_ant  26171 TGCATATTAAATAATTAAAAGGACCTAATTAAATTTCATGAGTCTGTTAA  26220
                     ...|.|...||.||.||..||||...||.|.|.|..||| |||.|.||.|
dac/DACH1        154 AAAAAAACCAAAAAGTAGGAGGAGAAAAGTTATTGCCAT-AGTTTTTTTA    202

LOC586986_ant  26221 TTCATAATTTAGTACTTTCATTACTAGT  26248
                     || |||.||..||..||.|.||.||.||
dac/DACH1        203 TT-ATACTTGTGTGTTTACCTTTCTGGT    229
```

→ below the threshold criteria

2. *Saccoglossus kowalevskii*

Description: PREDICTED: dachshund homolog 1 isoform X3
NCBI Gene: *100303544*
Transcript: XM_006824805.1
Product: XP_006824868.1

A

```
# Aligned_sequences: 2
# 1: XP_006824868.1_sense
# 2: dac/DACH1
# Matrix: EDNAFULL
# Gap_penalty: 16
# Extend_penalty: 4
#
# Length: 185
# Identity:     105/185 (56.8%)
# Similarity:   105/185 (56.8%)
# Gaps:          16/185 ( 8.6%)
# Score: 121
#
#
#=======================================
XP_006824868.  25248 GCCCTATTTTTTTTCCATGATTTATTTATTTAATATGGCTACATGTCATT  25297
                     ||||.||||...||.|..|.||.|||.||||||||...  |.||....||
dac/DACH1         29 GCCCAATTTCCGTTTCGAGTTTAATTAATTTAATAAAC--AAATTCTTTT     76

XP_006824868.  25298 TATTAGACGCGGTTTCATTGTCAGTAAAGAAAAGAAGA-TTTATTGTTTA  25346
                     ...|..|.....|.|||. ||  |||..||.|.||.|. |||.||.|||.
dac/DACH1         77 CGCTCTAAAAACTCTCAA-GT--GTATCGATACGATGCGTTTCTTTTTTT    123

XP_006824868.  25347 ------TTAAATTATTACGATAACAAAATAATAGAGAAAATCATAATAAA  25390
                           ||||||.|.||  |||||.|||.||.|.|.|||...|.||..|.
dac/DACH1        124 CCTTCGTTAAATAAATA--ATAACCAAAAAAAAAAAAAACCAAAAAGTAG    171

XP_006824868.  25391 ATGGCGTAATGATTCTGCAACATTGTGGTTTTATT  25425
                     ..||.|.||.|.|..|||  |||.||..|||||||
dac/DACH1        172 GAGGAGAAAAGTTATTGC--CATAGTTTTTTTATT    204
```

→ **below the threshold criteria**

B

```
# Aligned_sequences: 2
# 1: XP_006824868.1_antisense
# 2: dac/DACH1
# Matrix: EDNAFULL
# Gap_penalty: 16
# Extend_penalty: 4
#
# Length: 141
# Identity:      84/141 (59.6%)
# Similarity:    84/141 (59.6%)
# Gaps:          15/141 (10.6%)
# Score: 132
#
#
#=======================================
XP_006824868.  14900 GAACTTAATCAATATTTTTAA-AAATGCCTAACGGAC---AAATTC---A  14942
                     ||..|||||.|||.|..|.|| ||||.|.|..||..|   |||.||   |
dac/DACH1         45 GAGTTTAATTAATTTAATAAACAAATTCTTTTCGCTCTAAAAACTCTCAA     94

XP_006824868.  14943 TTAAATTAAAATCA-GCATTTCTGTGTTTCCATGG-------AATGCTAA  14984
                     .|..||..|.|..| ||.|||||.|.|||||.|.|       |||..|||
dac/DACH1         95 GTGTATCGATACGATGCGTTTCTTTTTTTCCTTCGTTAAATAAATAATAA    144

XP_006824868.  14985 AAAAAAAAAAGAAAAACAAGAGACAAGGAGAAAACAACTCA  15025
                     ..||||||||.||||||.|.|.|..|||||.|.|.||.|.|
dac/DACH1        145 CCAAAAAAAAAAAAAACCAAAAAGTAGGAGGAGAAAAGTTA    185
```

→ **below the threshold criteria**

3. Branchiostoma lanceolatum

3.a Gene BL03388_evm15

Gene: BL03388
Transcript: BL03388_evm15
Product: BL03388_evm15

A

```
# Aligned_sequences: 2
# 1: BL03388_sense
# 2: dac/DACH1
# Matrix: EDNAFULL
# Gap_penalty: 16
# Extend_penalty: 4
#
# Length: 90
# Identity:      54/90 (60.0%)
# Similarity:    54/90 (60.0%)
# Gaps:           0/90 ( 0.0%)
# Score: 126
#
#
#=======================================

BL03388_sense  12440 TCTTTTGGTTTGTCACATCTTCATTCAATAAAACAAAACAAAACTAAACA  12489
                     |.||||..||.||.|.||....|.|.|..||||.||||.||||..|||.|
dac/DACH1        118 TTTTTTCCTTCGTTAAATAAATAATAACCAAAAAAAAAAAAAAACCAAAAA    167

BL03388_sense  12490 AAATGGAATAAAATCTTAATTCAAAACATTTTTTAGGATA  12529
                     ..|.|.....|||..|||.|.|.|.|..|||||||..|||
dac/DACH1        168 GTAGGAGGAGAAAAGTTATTGCCATAGTTTTTTTATTATA    207
```

→ **Hit:**

```
>BL03388_sense_CRE-like
TCTTTTGGTTTGTCACATCTTCATTCAATAAAACAAAACAAAACTAAACAAAATGGAATAAAATCTTAATTCAAAACATTTTTTAGGATA
```

→ **BLASTN SEARCH** for BL03388_sense_CRE-like: e-value 5e-44

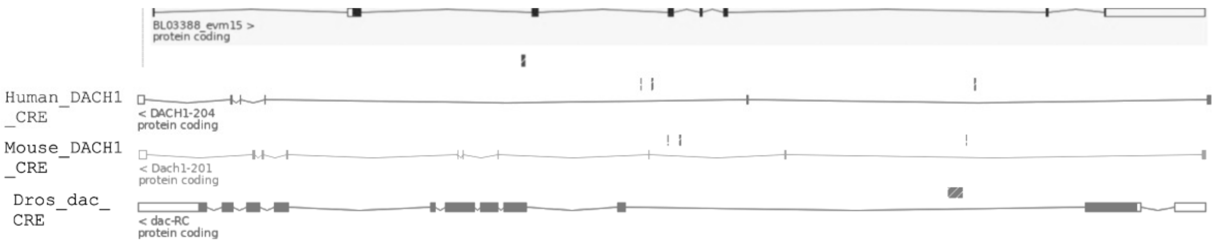

B

```
# Aligned_sequences: 2
# 1: BL03388_antisense
# 2: dac/DACH1
# Matrix: EDNAFULL
# Gap_penalty: 16
# Extend_penalty: 4
#
# Length: 58
# Identity:      37/58 (63.8%)
# Similarity:    37/58 (63.8%)
# Gaps:           0/58 ( 0.0%)
# Score: 101
#
#
#=======================================

BL03388_antis   7450 TGCATGTATTTGACCCATTTCTTTTTTAAGATCTTTAAATCTAGCACAAT   7499
                     ||.||..||..||..|.||||||||||....||.||||||..|..|.||.
dac/DACH1         96 TGTATCGATACGATGCGTTTCTTTTTTTCCTTCGTTAAATAAATAATAAC    145

BL03388_antis   7500 CAAGAAGA   7507
                     |||.||.|
dac/DACH1        146 CAAAAAAA    153
```

→ **Hit:**

>BL03388_antisense_CRE-like
TGCATGTATTTGACCCATTTCTTTTTTAAGATCTTTAAATCTAGCACAATCAAGAAGA

→ **BLASTN SEARCH** for BL03388_antisense_CRE-like: e-value 3.5e-25

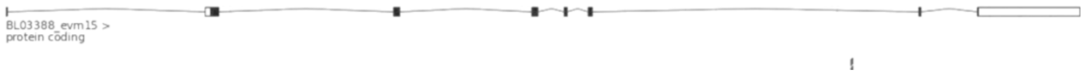

3.b Gene BL02962_evm3

Gene: BL02962
Transcript: BL02962_evm3
Product: BL02962_evm3

A

```
# Aligned_sequences: 2
# 1: BL02962_sense
# 2: dac/DACH1
# Matrix: EDNAFULL
# Gap_penalty: 16
# Extend_penalty: 4
#
# Length: 77
# Identity:      51/77 (66.2%)
# Similarity:    51/77 (66.2%)
# Gaps:           5/77 ( 6.5%)
# Score: 115
#
#
#=======================================

BL02962_sens   17527 ATGTACATAACAAATCTTATATTCACTACAAAATC-CAAGTTTCTATCGC  17575
                     ||.||...|||||||..|.|...|.|||.|||.|| |||||.  |||||.
dac/DACH1         56 ATTTAATAAACAAATTCTTTTCGCTCTAAAAACTCTCAAGTG--TATCGA    103

BL02962_sens   17576 TAAGATT--TTTATTTTTTTCTGTCAT  17600
                     ||.|||.  |||.|||||||||..||.|
dac/DACH1        104 TACGATGCGTTTCTTTTTTTCCTTCGT    130
```

→ **Hit:**

```
>BL02962_sense_CRE-like
ATGTACATAACAAATCTTATATTCACTACAAAATCCAAGTTTCTATCGCTAAGATTTTTATTTTTTTCTGTCAT
```

→ **BLASTN SEARCH** for BL02962_sense_CRE-like: e-value 1.4e-34

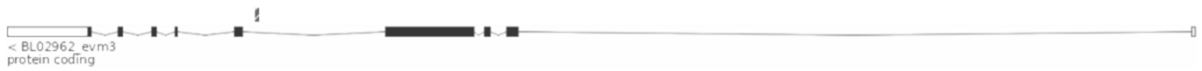

B

```
# Aligned_sequences: 2
# 1: BL02962_antisense
# 2: dac/DACH1
# Matrix: EDNAFULL
# Gap_penalty: 16
# Extend_penalty: 4
#
# Length: 56
# Identity:      40/56 (71.4%)
# Similarity:    40/56 (71.4%)
# Gaps:           1/56 ( 1.8%)
# Score: 124
#
#
#=======================================

BL02962_antis   4202 TTTATCTTGTATGTTCGAACAAATAAATAACAAACAAACAAATAAACAAA   4251
                     |||.|.||.|...||||.. ||||||||||.||.||||.|||.|||.||.
dac/DACH1        113 TTTCTTTTTTTCCTTCGTT-AAATAAATAATAACCAAAAAAAAAAAAAAC    161

BL02962_antis   4252 CAAACA   4257
                     ||||.|
dac/DACH1        162 CAAAAA    167
```

→ **Hit:**

```
>BL02962_antisense_CRE-like
TTTATCTTGTATGTTCGAACAAATAAATAACAAACAAACAAATAAACAAACAAACA
```

→ **BLASTN SEARCH** for BL02962_antisense_CRE-like: e-value 5.2e-24

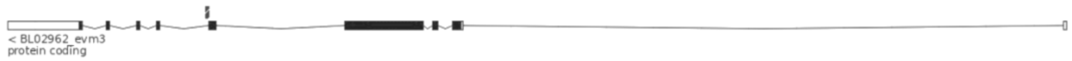

4. Ciona intestinalis

4.a Gene ENSCING00000019659

Description: novel gene
Gene: ENSCING00000019659
Transcript: ENSCINT00000037189.1
Product: ENSCINP00000034508

A

```
# Aligned_sequences: 2
# 1: ENSCING00000019659_sense
# 2: dac/DACH1
# Matrix: EDNAFULL
# Gap_penalty: 16
# Extend_penalty: 4
#
# Length: 147
# Identity:      83/147 (56.5%)
# Similarity:    83/147 (56.5%)
# Gaps:           9/147 ( 6.1%)
# Score: 111
#
#
#=======================================

ENSCING000000  15998 TTTAAATAATGTCCAGAAAAACATTAAT-TGTTTATCGGTTTTGAC--CA  16044
                     |.|||.|.|.||.||...|.|..||.. ||..|.||..||||..|  |.
dac/DACH1         80 TCTAAAAACTCTCAAGTGTATCGATACGATGCGTTTCTTTTTTTCCTTCG    129

ENSCING000000  16045 ATCAATAAA-AATTCCCAGAAAAATTAGTAACTATTTTTAACAGCTATTT  16093
                     .|.|||||| |||..|||.|||||..|..|||.|     ||.||......
dac/DACH1        130 TTAAATAAATAATAACCAAAAAAAAAAAAAAACCA-----AAAAGTAGGAG    174

ENSCING000000  16094 TTTAAAACTTTTTTTTAAAGTTTTTTTATTTATTTTTCGTTTTTAGC  16140
                     ...||||.||.||...|.||||||||||||....||..||.||||.|
dac/DACH1        175 GAGAAAAGTTATTGCCATAGTTTTTTTATTATACTTGTGTGTTTACC    221
```

→ below the threshold criteria

B

```
# Aligned_sequences: 2
# 1: ENSCING00000019659_antisense
# 2: dac/DACH1
# Matrix: EDNAFULL
# Gap_penalty: 16
# Extend_penalty: 4
#
# Length: 60
# Identity:      41/60 (68.3%)
# Similarity:    41/60 (68.3%)
# Gaps:           2/60 ( 3.3%)
# Score: 105
#
#
#=======================================

ENSCING000000   2389 TAATAAATATTGCATAAGATATGGTTTCTGCTTAAAAAACTTTCAGTTGT   2438
                     ||||.||| ||....||.|.||..|||..|||..|||||||.|||..|||
dac/DACH1         50 TAATTAAT-TTAATAAACAAATTCTTTTCGCTCTAAAAACTCTCAAGTGT     98

ENSCING000000   2439 AATCAATACG   2448
                     | ||.|||||
dac/DACH1         99 A-TCGATACG    107
```

→ **Hit:**

>ENSCING00000019659_antisense_CRE-like

TAATAAATATTGCATAAGATATGGTTTCTGCTTAAAAAACTTTCAGTTGTAATCAATACG

→ **BLASTN SEARCH** for ENSCING00000019659_antisense_CRE-like: e-value 7e-27

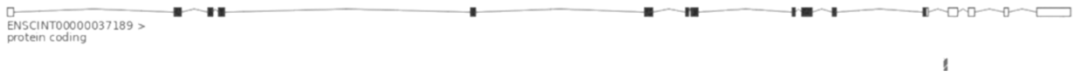

4.b Gene ENSCING00000000087

Description: novel gene
Gene: ENSCING00000000087
Transcript: ENSCINT00000000131.3
Product: ENSCINP00000000131

A

```
# Aligned_sequences: 2
# 1: ENSCING00000000087_sense
# 2: dac/DACH1
# Matrix: EDNAFULL
# Gap_penalty: 16
# Extend_penalty: 4
#
# Length: 107
# Identity:      67/107 (62.6%)
# Similarity:    67/107 (62.6%)
# Gaps:           9/107 ( 8.4%)
# Score: 115
#=======================================

ENSCING000000   1990 AATTCCCCATTCGGGTTTAATACGCCAAGTTTACA--TTCTT--CGCTTA   2035
                     ||||.||..||||.|||||||......|.|..|||  |||||  ||||..
dac/DACH1         33 AATTTCCGTTTCGAGTTTAATTAATTTAATAAACAAATTCTTTTCGCTCT     82

ENSCING000000   2036 AAAGAAAACAAGTAAGTGTATATGAAATAATGGGTTTTTATTGTACAAT   2085
                     ||   ||||....||||||||. ||.|..|||.||||.||.||.|.|...
dac/DACH1         83 AA---AAACTCTCAAGTGTATC-GATACGATGCGTTTCTTTTTTTCCTTC    128

ENSCING000000   2086 GTATAAA   2092
                     || ||||
dac/DACH1        129 GT-TAAA    134
```

→ **Hit:**

>ENSCING00000000087_sense_CRE-like
AATTCCCCATTCGGGTTTAATACGCCAAGTTTACATTCTTCGCTTAAAAGAAAACAAGTAAGTGTATATGAAATAATGGGTTTTTATTGTACAATG
TATAAA

→ **BLASTN SEARCH** for ENSCING00000000087_sense_CRE-like: e-value 4e-52

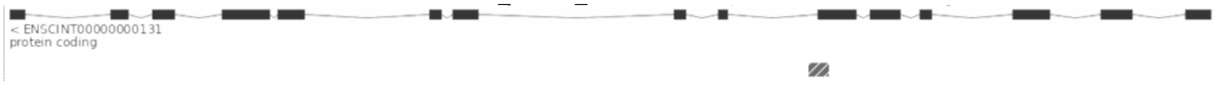

→ **does not meet criteria:** does not fully lie in intronic region

B

```
# Aligned_sequences: 2
# 1: ENSCING00000000087_antisense
# 2: dac/DACH1
# Matrix: EDNAFULL
# Gap_penalty: 16
# Extend_penalty: 4
#
# Length: 132
# Identity:      74/132 (56.1%)
# Similarity:    74/132 (56.1%)
# Gaps:           5/132 ( 3.8%)
# Score: 102
#
#
#=======================================

ENSCING000000     51 ATTTTGTTCACATCTTGTATTCGCAT--ATCATGCTCATGTCTGCAAATG     98
                     ||||..|..|||..||.|.|||||..  |..|..||||.||.|....||
dac/DACH1         56 ATTTAATAAACAAATTCTTTTCGCTCTAAAAACTCTCAAGTGTATCGAT-    104

ENSCING000000     99 ATCATATGTAATATATGTTGAATGTTTATACAAAGAATATAAGCAAAAAA    148
                     |..||..||....|.|.||...|..|||.|.|||.||||  |.|||||||
dac/DACH1        105 ACGATGCGTTTCTTTTTTTCCTTCGTTAAATAAATAATA--ACCAAAAAA    152

ENSCING000000    149 ATCAAAATCAAAATATACCAAGGCATAATGTT    180
                     |..||||.|.|||.|.....|||...||.|||
dac/DACH1        153 AAAAAAAACCAAAAAGTAGGAGGAGAAAAGTT    184
```

→ below the threshold criteria

5. *Lingula anatina*

5.a Gene g13798.t1

Gene: g13798
Transcript: g13798.t1
Product: g13798.t1
NCBI: XP_013405870.1

A

```
# Aligned_sequences: 2
# 1: g13798.t1_sense
# 2: dac/DACH1
# Matrix: EDNAFULL
# Gap_penalty: 16
# Extend_penalty: 4
#
# Length: 178
# Identity:     103/178 (57.9%)
# Similarity:   103/178 (57.9%)
# Gaps:          16/178 ( 9.0%)
# Score: 119
#
#
#=======================================

g13798.t1_sen    552 TACTTAAATTAATTGAGAGGTTGTAATATGAC-AGAAAGACT---GTGTC    597
                     ||.||||.|||||..|.|..||.|..|....| |.|||..||   ||||.
dac/DACH1         50 TAATTAATTTAATAAACAAATTCTTTTCGCTCTAAAAACTCTCAAGTGTA     99

g13798.t1_sen    598 ACAGAACGATGATTTCCTTCATTTTAATATCCTGTTGAATTTCTCATAAC    647
                     .|...||||||..||.|||  ||||....||  |||.|||...|.|||||
dac/DACH1        100 TCGATACGATGCGTTTCTT--TTTTTCCTTC--GTTAAATAAATAATAAC    145

g13798.t1_sen    648 ---AAAGAAAAGACACGAGAGAGT-GAATGCTTCTTATTATCCATGCCTT    693
                        |||.||||.|.||.|.|.||| |.|.|.......||||   ||||.|
dac/DACH1        146 CAAAAAAAAAAAAAACCAAAAAGTAGGAGGAGAAAAGTTAT---TGCCAT    192

g13798.t1_sen    694 CCTGTTTGTATTTCACATGT-TCTTAAC    720
                     ..|.|||.||||..||.||| |.||.||
dac/DACH1        193 AGTTTTTTTATTATACTTGTGTGTTTAC    220
```

→ below the threshold criteria

B

```
# Aligned_sequences: 2
# 1: g13798.t1_antisense
# 2: dac/DACH1
# Matrix: EDNAFULL
# Gap_penalty: 16
# Extend_penalty: 4
#
# Length: 69
# Identity:      43/69 (62.3%)
# Similarity:    43/69 (62.3%)
# Gaps:           1/69 ( 1.4%)
# Score: 99
#
#
#=======================================

g13798.t1_ant   7989 CATATAGTTACAGGCAACAATTAATATCCATCTTATTTTCTTTTATGTGT   8038
                     ||.|.|||...|||..|.||.|.||..||||..| ||||.|.||||...|
dac/DACH1        162 CAAAAAGTAGGAGGAGAAAAGTTATTGCCATAGT-TTTTTATTATACTT    210

g13798.t1_ant   8039 GCGTGTAAAGGTTTCTGTT   8057
                     |.||||..|..||||||.|
dac/DACH1        211 GTGTGTTTACCTTTCTGGT    229
```

→ **Hit:**

>g13798.t1_antisense_CRE-like
CATATAGTTACAGGCAACAATTAATATCCATCTTATTTTCTTTTATGTGTGCGTGTAAAGGTTTCTGTT

→ **BLASTN SEARCH** for g13798.t1_antisense_CRE-like: e-value 1e-31

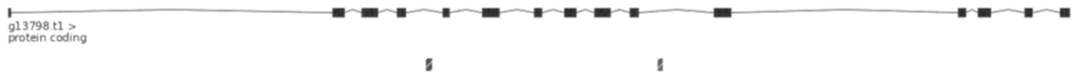

5.b Gene g33597.t1

Gene: g33597
Transcript: g33597.t1
Product: g33597.t1

A

```
# Aligned_sequences: 2
# 1: g33597.t1_sense
# 2: dac/DACH1
# Matrix: EDNAFULL
# Gap_penalty: 16
# Extend_penalty: 4
#
# Length: 62
# Identity:      39/62 (62.9%)
# Similarity:    39/62 (62.9%)
# Gaps:           1/62 ( 1.6%)
# Score: 91
#
#
#=======================================

g33597.t1_sen    887 TATAAAAGTAATAAACTGAAATAAAGAAAAGGGGAAAGATCGAAGAACAA    936
                     ||.|.||.||||||.|..|||.|||.||||....|||| |.|.||.|.||
dac/DACH1        131 TAAATAAATAATAACCAAAAAAAAAAAAAAACCAAAAAG-TAGGAGGAGAA    179

g33597.t1_sen    937 GAAAAATAGCAA    948
                     .|...||.||.|
dac/DACH1        180 AAGTTATTGCCA    191
```

→ **Hit:**

```
>g33597.t1_sense_CRE-like
TATAAAAGTAATAAACTGAAATAAAGAAAAGGGGAAAGATCGAAGAACAAGAAAAATAGCAA
```

→ **BLASTN SEARCH** for g33597.t1_sense_CRE-like: e-value 1.4e-27

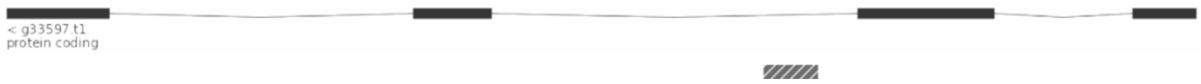

B

```
# Aligned_sequences: 2
# 1: g33597.t1_antisense
# 2: dac/DACH1
# Matrix: EDNAFULL
# Gap_penalty: 16
# Extend_penalty: 4
#
# Length: 104
# Identity:      56/104 (53.8%)
# Similarity:    56/104 (53.8%)
# Gaps:           1/104 ( 1.0%)
# Score: 76
#
#
#=======================================

g33597.t1_ant     24 TACGTGAAACTTTGGAAAAACAACTACATGATGAACAGAGAAGTAGAGGT     73
                     |.|||.|||......|.||.|||..|.|..|..|||..|.||||||..|.
dac/DACH1        126 TTCGTTAAATAAATAATAACCAAAAAAAAAAAAAAACCAAAAAGTAGGAGG    175

g33597.t1_ant     74 AAACAAATTTGTGA-ACATTTTGTTAGTTAGAAAATACAGCATACTTTTT    122
                     |.|.||.||..||. |.|.|||.||..|||.|.......|..|||.|||.
dac/DACH1        176 AGAAAAGTTATTGCCATAGTTTTTTTATTATACTTGTGTGTTTACCTTTC    225

g33597.t1_ant    123 TTGT    126
                     |.||
dac/DACH1        226 TGGT    229
```

→ **below the threshold criteria**

6. Capitella teleta

6.a Gene CapteG137512

Gene: CapteG137512
Transcript: CapteT137512
Product: CapteP137512

A

```
# Aligned_sequences: 2
# 1: CapteG137512_sense
# 2: dac/DACH1
# Matrix: EDNAFULL
# Gap_penalty: 16
# Extend_penalty: 4
#
# Length: 22
# Identity:      15/22 (68.2%)
# Similarity:    15/22 (68.2%)
# Gaps:           0/22 ( 0.0%)
# Score: 47
#
#
#=======================================

CapteG137512_    301 TAACAGGTGCAAACTGATCGCC    322
                     ||..|||.|.|||.|.||.|||
dac/DACH1        169 TAGGAGGAGAAAAGTTATTGCC    190
```

→ below the threshold criteria

B

```
# Aligned_sequences: 2
# 1: CapteG137512_antisense
# 2: dac/DACH1
# Matrix: EDNAFULL
# Gap_penalty: 16
# Extend_penalty: 4
#
# Length: 22
# Identity:      15/22 (68.2%)
# Similarity:    15/22 (68.2%)
# Gaps:           0/22 ( 0.0%)
# Score: 47
#
#
#=======================================

CapteG137512_     95 CCCTCTCAAAACACGGACTTGT    116
                     |.||||.|||||.|..|..|||
dac/DACH1         77 CGCTCTAAAAACTCTCAAGTGT     98
```

→ below the threshold criteria

6.b Gene CapteG227573

Gene: CapteG227573
Transcript: CapteT227573
Product: CapteP227573

A

```
# Aligned_sequences: 2
# 1: CapteG227573_sense
# 2: dac/DACH1
# Matrix: EDNAFULL
# Gap_penalty: 16
# Extend_penalty: 4
#
# Length: 148
# Identity:      85/148 (57.4%)
# Similarity:    85/148 (57.4%)
# Gaps:          14/148 ( 9.5%)
# Score: 89
#
#
#=======================================

CapteG227573_   2653 ACACACAAAAACTA--CGCTTGACAAGATCAAGCCTGAGTCACGTGACCT   2700
                     |.|.|||||..||.  ||||..|.||..||  .|..|.||..||..||..
dac/DACH1         61 ATAAACAAATTCTTTTCGCTCTAAAAACTC--TCAAGTGTATCGATACGA    108

CapteG227573_   2701 TGC--TACGCTTTGTCGTTGGTTGAAAAGAACAAGAGGTTACCAAAAAAA   2748
                     |||  |.|..|||.||.||.||| |||..||.||.|    ||||||||||
dac/DACH1        109 TGCGTTTCTTTTTTTCCTTCGTT-AAATAAATAATA----ACCAAAAAAA    153

CapteG227573_   2749 ATCAGACCAACAAAACA--AGCA-AACTGTTGAGTCCAGAGGCTTTGT   2793
                     |..|.|.|.|.|||..|  ||.| ||..|||....|||.||..|||.|
dac/DACH1        154 AAAAAAACCAAAAAGTAGGAGGAGAAAAGTTATTGCCATAGTTTTTTT    201
```

→ below the threshold criteria

B

```
# Aligned_sequences: 2
# 1: CapteG227573_antisense
# 2: dac/DACH1
# Matrix: EDNAFULL
# Gap_penalty: 16
# Extend_penalty: 4
#
# Length: 71
# Identity:      47/71 (66.2%)
# Similarity:    47/71 (66.2%)
# Gaps:           1/71 ( 1.4%)
# Score: 127
#
#
#=======================================

CapteG227573_   2791 AAAAAAAAAAAAAAAAAAAAAAAACGGGG-GAAAAGATGTGGAAGGATTT   2839
                     ||||||||||||||..|||||..|.|.|| ||||||.|.|.|....|.||
dac/DACH1        147 AAAAAAAAAAAAAACCAAAAAGTAGGAGGAGAAAAGTTATTGCCATAGTT    196

CapteG227573_   2840 CTTTTGACGCGCTTTTGTTTT   2860
                     .||||......|||.|||.||
dac/DACH1        197 TTTTTATTATACTTGTGTGTT    217
```

→ **Hit:**

```
>CapteG227573_antisense_CRE-like
AAAAAAAAAAAAAAAAAAAAAAAACGGGGGAAAAGATGTGGAAGGATTTCTTTTGACGCGCTTTTGTTTT
```

→ **BLASTN SEARCH** for CapteG227573_antisense_CRE-like: e-value 2.1e-32

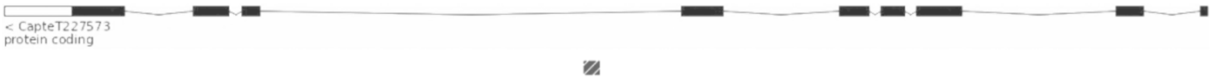

7. Octopus bimaculoides

7.a Gene Ocbimv22003564m.g

Gene: Ocbimv22003564m.g
Transcript: Ocbimv22003564m
Product: Ocbimv22003564m.p

A

```
# Aligned_sequences: 2
# 1: Ocbimv22003564m.g_sense
# 2: dac/DACH1
# Matrix: EDNAFULL
# Gap_penalty: 16
# Extend_penalty: 4
#
# Length: 24
# Identity:      17/24 (70.8%)
# Similarity:    17/24 (70.8%)
# Gaps:           0/24 ( 0.0%)
# Score: 57
#
#
#=======================================

Ocbimv2200356    504 AATTGCTAATAACCCTGAAAACAA    527
                     |||...||||||||...||||.||
dac/DACH1        133 AATAAATAATAACCAAAAAAAAAA    156
```

→ **below the threshold criteria**

B

```
# Aligned_sequences: 2
# 1: Ocbimv22003564m.g_antisense
# 2: dac/DACH1
# Matrix: EDNAFULL
# Gap_penalty: 16
# Extend_penalty: 4
#
# Length: 30
# Identity:      21/30 (70.0%)
# Similarity:    21/30 (70.0%)
# Gaps:           0/30 ( 0.0%)
# Score: 69
#
#
#=======================================

Ocbimv2200356     36 ATCAAATTCTTTGCACGACAACAACTTGCA     65
                     |.||||||||||.|.|...||.||||..||
dac/DACH1         64 AACAAATTCTTTTCGCTCTAAAAACTCTCA     93
```

→ **below the threshold criteria**

7.b Gene Ocbimv22017627m.g

Gene: Ocbimv22017627m.g
Transcript: Ocbimv22017627m
Product: Ocbimv22017627m.p

A

```
# Aligned_sequences: 2
# 1: Ocbimv22017627m.g_sense
# 2: dac/DACH1
# Matrix: EDNAFULL
# Gap_penalty: 16
# Extend_penalty: 4
#
# Length: 84
# Identity:      54/84 (64.3%)
# Similarity:    54/84 (64.3%)
# Gaps:           2/84 ( 2.4%)
# Score: 138
#
#
#=======================================

Ocbimv2201762  29383 GTTTTCTTTTTTCTTTCAAACATGAAAAACCAACAAATAAACAAAAGTAA  29432
                     ||||..|||||||.|||....|..|||.|..|||.||.|||.||||  ||
dac/DACH1        112 GTTTCTTTTTTTCCTTCGTTAAATAAATAATAACCAAAAAAAAAAA--AA    159

Ocbimv2201762  29433 ACAGAAAAAAAAAAAGAAAGAAGACAAGGCCATA  29466
                     ||..||||..|..|.||.|.|||..|..||||||
dac/DACH1        160 ACCAAAAAGTAGGAGGAGAAAAGTTATTGCCATA    193
```

→ **Hit:**

```
>Ocbimv22017627m.g_sense_CRE-like
GTTTTCTTTTTTCTTTCAAACATGAAAAACCAACAAATAAACAAAAGTAAACAGAAAAAAAAAAAGAAAGAAGACAAGGCCATA
```

→ **BLASTN SEARCH** for Ocbimv22017627m.g_sense_CRE-like: e-value 8.1e-40

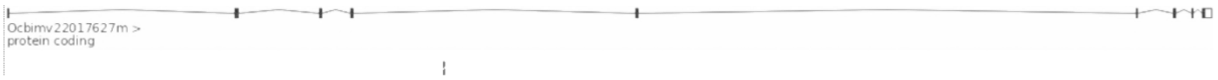

B

```
# Aligned_sequences: 2
# 1: Ocbimv22017627m.g_antisense
# 2: dac/DACH1
# Matrix: EDNAFULL
# Gap_penalty: 16
# Extend_penalty: 4
#
# Length: 67
# Identity:      46/67 (68.7%)
# Similarity:    46/67 (68.7%)
# Gaps:           1/67 ( 1.5%)
# Score: 134
#
#
#=======================================

Ocbimv2201762   6161 AAAAATAGCCGAAATAAAATAAAACAAACTTGTATATTTGAAAAACTTAT   6210
                     ||.||||.||.|||.||||.|||||.||...|||.......||||.||||
dac/DACH1        137 AATAATAACCAAAAAAAAAAAAAACCAAAAAGTAGGAGGAGAAAAGTTAT    186

Ocbimv2201762   6211 TTCAATA-TTTGTTTAT   6226
                     |.|.||| |||.|||||
dac/DACH1        187 TGCCATAGTTTTTTTAT    203
```

→ **Hit:**

>Ocbimv22017627m.g_antisense_CRE-like
AAAAATAGCCGAAATAAAATAAAACAAACTTGTATATTTGAAAAACTTATTTCAATATTTGTTTAT

→ **BLASTN SEARCH** for Ocbimv22017627m.g_antisense_CRE-like: e-value 3.2e-29

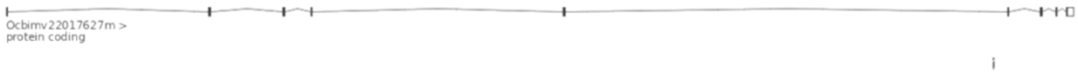

8. Schistosoma mansoni

Description: Putative dachshund homolog
Gene: **Smp_196410**
Transcript: Smp_196410.1
Product: Smp_196410.1

A

```
# Aligned_sequences: 2
# 1: Smp_196410.1_sense
# 2: dac/DACH1
# Matrix: EDNAFULL
# Gap_penalty: 16
# Extend_penalty: 4
#
# Length: 95
# Identity:      57/95 (60.0%)
# Similarity:    57/95 (60.0%)
# Gaps:           1/95 ( 1.1%)
# Score: 121
#
#
#=======================================

Smp_196410.1_  14743 GTGTTTACTTATTTTAA-ATCCATATGCATTTTTATCTAGTTACTGCAAA  14791
                     |.|||||.|||.||||| |..||.||.|.|||...||||...|||...||
Dac/DACH1         45 GAGTTTAATTAATTTAATAAACAAATTCTTTTCGCTCTAAAAACTCTCAA     94

Smp_196410.1_  14792 ACATATTTGAACGATGTATATCTCGTTGCCTTCAGCTGAATAATT  14836
                     ...|||....||||||..|.|||..||..|.|..|.|.|||||.|
Dac/DACH1         95 GTGTATCGATACGATGCGTTTCTTTTTTTCCTTCGTTAAATAAAT    139
```

→ **Hit:**

```
>Smp_196410.1_sense_CRE-like
GTGTTTACTTATTTTAAATCCATATGCATTTTTATCTAGTTACTGCAAAACATATTTGAACGATGTATATCTCGTTGCCTTCAGCTGAATAATT
```

→ **BLASTN SEARCH** for Smp_196410_sense_CRE-like: e-value 1.6e-46

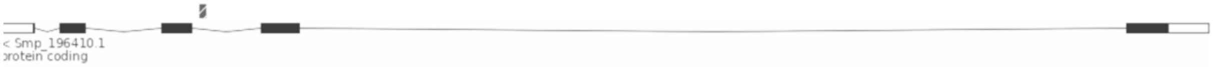

B

```
# Aligned_sequences: 2
# 1: Smp_196410.1_antisense
# 2: dac_DACH1
# Matrix: EDNAFULL
# Gap_penalty: 16
# Extend_penalty: 4
#
# Length: 187
# Identity:     108/187 (57.8%)
# Similarity:   108/187 (57.8%)
# Gaps:          13/187 ( 7.0%)
# Score: 140
#
#
#=======================================

Smp_196410.1_   7970 TTTTCTTTTCTGACAAAACCAGTTAAATCCTGTCAAAAGTATATTCACA   8019
                     |||..||...|.|..||||.|.||...|.|..||.||||    |.||.||
dac_DACH1         48 TTTAATTAATTTAATAAACAAATTCTTTTCGCTCTAAAA----ACTCTCA     93

Smp_196410.1_   8020 TTTCATATC-ATAAAATACGAATTATTTTTTTTGTTTTTACAATTTTTAC   8068
                     ..|. |||| |||..||.||. ||.|||||||..||..|..|||...||.
dac_DACH1         94 AGTG-TATCGATACGATGCGT-TTCTTTTTTTCCTTCGTTAAATAAATAA    141

Smp_196410.1_   8069 TACTGAAAATAAAATAAAACAAGTAAACTGTGAAATAATTTCAAAGAAAT   8118
                     ||...||||.||||.|||||.|. |||.|..||....|    ||||..||
dac_DACH1        142 TAACCAAAAAAAAAAAAAACCAA-AAAGTAGGAGGAGA----AAAGTTAT    186

Smp_196410.1_   8119 TTAGTCCGTTTTTATCTATTCACCTATCTGTTTACCT   8155
                     |......||||||.|.| |..||.|.|.|||||||||
dac_DACH1        187 TGCCATAGTTTTTTTAT-TATACTTGTGTGTTTACCT    222
```

→ below the threshold criteria

9. Adineta vaga

9.a Gene GSADVG00044000001

Gene: GSADVG00044000001
Transcript: GSADVT00044000001
Product: GSADVT00044000001

A

```
# Aligned_sequences: 2
# 1: GSADVG00044000001_sense
# 2: dac/DACH1
# Matrix: EDNAFULL
# Gap_penalty: 16
# Extend_penalty: 4
#
# Length: 166
# Identity:      92/166 (55.4%)
# Similarity:    92/166 (55.4%)
# Gaps:           8/166 ( 4.8%)
# Score: 104
#
#
#=======================================

GSADVG0004400   2102 AAATGAATTAACAAAAGAACAAGGTTCTCGACAAAAACT-TGAACAAGAT   2150
                     ||.|.|.||||.|||..||.....|||.|...||||||| |.||....||
dac/DACH1         51 AATTAATTTAATAAACAAATTCTTTTCGCTCTAAAAACTCTCAAGTGTAT    100

GSADVG0004400   2151 ATTCTCGAA---TTACGACGTATTCGAAGTAAAAAAAACGAAGAAATTCA   2197
                     .....|||.   ||.|....|.|.|...||.|||.|||. ||.||....|
dac/DACH1        101 CGATACGATGCGTTTCTTTTTTTCCTTCGTTAAATAAAT-AATAACCAAA    149

GSADVG0004400   2198 AATAAAAAAAACGAAGAAATTCAAATAAAAAAATCTTCTCTAATTCATTT   2247
                     ||.|||||||||.||.||.|...|..|.||||.|..|..|.|  |..|||
dac/DACH1        150 AAAAAAAAAAAACCAAAAAGTAGGAGGAGAAAAGTTATTGCCA--TAGTTT    197

GSADVG0004400   2248 TTCTAGATGTACTTAT   2263
                     ||.||. |.|||||.|
dac/DACH1        198 TTTTAT-TATACTTGT    212
```

→ below the threshold criteria

B

```
# Aligned_sequences: 2
# 1: GSADVG00044000001_antisense
# 2: dac/DACH1
# Matrix: EDNAFULL
# Gap_penalty: 16
# Extend_penalty: 4
#
# Length: 118
# Identity:      67/118 (56.8%)
# Similarity:    67/118 (56.8%)
# Gaps:           5/118 ( 4.2%)
# Score: 107
#
#
#=======================================

GSADVG0004400    298 TTCTTTTGTTAATTCATTTCGTAAATCA--ACTATAAAATATATAAAATC    345
                     |||||||.||..|||.||...|||||.|  ||.|.||||.|.|.|||...
dac/DACH1        114 TTCTTTTTTTCCTTCGTTAAATAAATAATAACCAAAAAAAAAAAAAAACCA    163

GSADVG0004400    346 AAAATCATCACATTAAATTGTGTTTTCAT---TTTACCAATTTCATTTTC    392
                     ||||..|..|....|||...|.||..|||   |||...|.|.|..||.|.
dac/DACH1        164 AAAAGTAGGAGGAGAAAAGTTATTGCCATAGTTTTTTATTATACTTGTG    213

GSADVG0004400    393 TAATTGTTTTTCTCGTAG    410
                     |..||...|||||.||.|
dac/DACH1        214 TGTTTACCTTTCTGGTGG    231
```

→ **below the threshold criteria**

9.b Gene GSADVG00042435001

Gene: GSADVG00042435001
Transcript: GSADVT00042435001
Product: GSADVT00042435001

A

```
# Aligned_sequences: 2
# 1: GSADVG00042435001_sense
# 2: dac/DACH1
# Matrix: EDNAFULL
# Gap_penalty: 16
# Extend_penalty: 4
#
# Length: 67
# Identity:      43/67 (64.2%)
# Similarity:    43/67 (64.2%)
# Gaps:           2/67 ( 3.0%)
# Score: 109
#
#
#=======================================

GSADVG0004243   1219 TTTTGCTTTNGAGACTAATTACATTAATCACCTAATATTTATTGAGATTT   1268
                     |||...||| |||..||||||..|||||.|.|.||  ||..||..|.|.|
dac/DACH1         35 TTTCCGTTTCGAGTTTAATTAATTTAATAAACAAA--TTCTTTTCGCTCT     82

GSADVG0004243   1269 AACTACTGTCACGAGTA   1285
                     ||..|||.|||.|.|||
dac/DACH1         83 AAAAACTCTCAAGTGTA     99
```

→ **Hit:**
>GSADVG00042435001_sense_CRE-like
TTTTGCTTTNGAGACTAATTACATTAATCACCTAATATTTATTGAGATTTAACTACTGTCACGAGTA

→ **BLASTN SEARCH** for GSADVG00042435001_sense_CRE-like: e-value: 5e-29

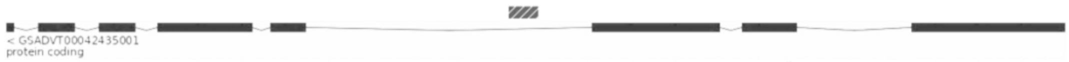

B

```
# Aligned_sequences: 2
# 1: GSADVG00042435001_antisense
# 2: dac/DACH1
# Matrix: EDNAFULL
# Gap_penalty: 16
# Extend_penalty: 4
#
# Length: 118
# Identity:      67/118 (56.8%)
# Similarity:    67/118 (56.8%)
# Gaps:           5/118 ( 4.2%)
# Score: 107
#
#
#=======================================

GSADVG0004243    288 TTCTTTTGTTAATTCATTTCGTAAATCA--ACTATAAAATATATAAAATC    335
                     |||||||.||..|||.||...|||||.|  ||.|.||||.|.|.|||...
dac/DACH1        114 TTCTTTTTTTCCTTCGTTAAATAAATAATAACCAAAAAAAAAAAAAACCA    163

GSADVG0004243    336 AAAATCATCACATCAAATTGTGTTTTCAT---TTTACCAATTTCATTTTC    382
                     ||||..|..|....|||...|.||..|||   |||...|.|.|..||.|.
dac/DACH1        164 AAAAGTAGGAGGAGAAAAGTTATTGCCATAGTTTTTTATTATACTTGTG    213

GSADVG0004243    383 TAATTGTTTTTCTCGTAG    400
                     |..||...|||||.||.|
dac/DACH1        214 TGTTTACCTTTCTGGTGG    231
```

→ **below the threshold criteria**

9.c Gene GSADVG00020231001

Gene: GSADVG00020231001
Transcript: GSADVT00020231001
Product: GSADVT00020231001

A

```
# Aligned_sequences: 2
# 1: GSADVG00020231001_sense
# 2: dac/DACH1
# Matrix: EDNAFULL
# Gap_penalty: 16
# Extend_penalty: 4
#
# Length: 110
# Identity:      61/110 (55.5%)
# Similarity:    61/110 (55.5%)
# Gaps:           5/110 ( 4.5%)
# Score: 73
#
#
#=======================================

GSADVG0002023    616 TCTTTTTCTTCATTCATTTTCGGATTAC--AAATATCCAATACCTTCAAA    663
                     |.||||.||||.||.|.|.....||.||  |||.|...||.|.|...|||
dac/DACH1        118 TTTTTTCCTTCGTTAAATAAATAATAACCAAAAAAAAAAAAAAACCAAAAA    167

GSADVG0002023    664 TCAAGATTATTTAACAATTCCATCCATAAATAATTTCTCTTTATTCAATT    713
                     ..|.||..|.  ||.|.||....|||||..|..|||.| |.||.|....|
dac/DACH1        168 GTAGGAGGAG--AAAAGTTATTGCCATAGTTTTTTTAT-TATACTTGTGT    214

GSADVG0002023    714 GTTTACCATT    723
                     |||||||.||
dac/DACH1        215 GTTTACCTTT    224
```

→ below the threshold criteria

B

```
# Aligned_sequences: 2
# 1: GSADVG00020231001_antisense
# 2: dac/DACH1
# Matrix: EDNAFULL
# Gap_penalty: 16
# Extend_penalty: 4
#
# Length: 67
# Identity:      41/67 (61.2%)
# Similarity:    41/67 (61.2%)
# Gaps:           2/67 ( 3.0%)
# Score: 89
#
#
#=======================================

GSADVG0002023    789 GAAGGAGAAAATGCATT--CATTGGTGAATTACTATCATTTGTTGAACAA    836
                     |.|||||||||...|||  |||.|.|...|||.|||..||...||...|.
dac/DACH1        171 GGAGGAGAAAAGTTATTGCCATAGTTTTTTTATTATACTTGTGTGTTTAC    220

GSADVG0002023    837 TGTTCTTGTGACATGAT    853
                     ..||||.|||.|.||||
dac/DACH1        221 CTTTCTGGTGGCTTGAT    237
```

→ **Hit:**

>GSADVG00020231001_antisense_CRE-like

GAAGGAGAAAATGCATTCATTGGTGAATTACTATCATTTGTTGAACAATGTTCTTGTGACATGAT

→ **BLASTN SEARCH** for GSADVG00020231001_antisense_CRE-like: e-value 1.2e-29

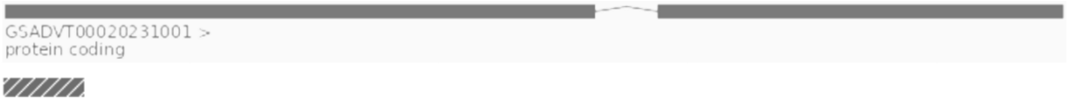

→ **does not meet criteria:** does not lie in intronic region

9.d Gene GSADVG00018241001

Gene: GSADVG00018241001
Transcript: GSADVT00018241001
Product: GSADVT00018241001

A

```
# Aligned_sequences: 2
# 1: GSADVG00018241001_sense
# 2: dac/DACH1
# Matrix: EDNAFULL
# Gap_penalty: 16
# Extend_penalty: 4
#
# Length: 110
# Identity:      61/110 (55.5%)
# Similarity:    61/110 (55.5%)
# Gaps:           5/110 ( 4.5%)
# Score: 73
#
#
#=======================================

GSADVG0001824    615 TCTTTTTCTTCATTCATTTTCGGATTAC--AAATATCCAATACCTTCAAA    662
                     |.||||.||||.||.|.|.....||.||  |||.|...||.|.|...|||
dac/DACH1        118 TTTTTTCCTTCGTTAAATAAATAATAACCAAAAAAAAAAAAAACCAAAAA    167

GSADVG0001824    663 TCAAGATTATTTAACAATTCCATCCATAAATAATTTCTCTTTATTCAATT    712
                     ..|.||..|.  ||.|.||....|||||..|..|||.| |.||.|....|
dac/DACH1        168 GTAGGAGGAG--AAAAGTTATTGCCATAGTTTTTTTAT-TATACTTGTGT    214

GSADVG0001824    713 GTTTACCATT    722
                     |||||||.||
dac/DACH1        215 GTTTACCTTT    224
```

→ below the threshold criteria

B

```
# Aligned_sequences: 2
# 1: GSADVG00018241001_antisense
# 2: dac/DACH1
# Matrix: EDNAFULL
# Gap_penalty: 16
# Extend_penalty: 4
#
# Length: 65
# Identity:      39/65 (60.0%)
# Similarity:    39/65 (60.0%)
# Gaps:           2/65 ( 3.1%)
# Score: 79
#
#
#=======================================

GSADVG0001824    793 GAAGGAGAAAATGCATT--CATTGGTGAATTACTATCATTTGTTGAACAA    840
                     |.|||||||||...|||  |||.|.|...|||.|||..||...||...|.
dac/DACH1        171 GGAGGAGAAAAGTTATTGCCATAGTTTTTTTATTATACTTGTGTGTTTAC    220

GSADVG0001824    841 TGTTCTTGTGACATG    855
                     ..||||.|||.|.||
dac/DACH1        221 CTTTCTGGTGGCTTG    235
```

→ **Hit:**
>GSADVG00018241001_antisense_CRE-like
GAAGGAGAAAATGCATTCATTGGTGAATTACTATCATTTGTTGAACAATGTTCTTGTGACATG

→ **BLASTN SEARCH** for GSADVG00018241001_antisense_CRE-like: e-value 1.8e-28

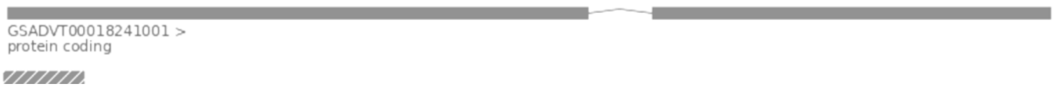

→ **does not meet criteria:** does not fully lie in intronic region

10. Hypsibius exemplaris

No *dachshund/DACH1* homolog.

11. Caenorhabditis elegans

Description: dac-1
Gene: WBGene00000895
Transcript: B0412.1a.1
Product: B0412.1a.1

A

```
# Aligned_sequences: 2
# 1: B0412.1a.1_sense
# 2: dac/DACH1
# Matrix: EDNAFULL
# Gap_penalty: 16
# Extend_penalty: 4
#
# Length: 181
# Identity:     101/181 (55.8%)
# Similarity:   101/181 (55.8%)
# Gaps:          17/181 ( 9.4%)
# Score: 101
#
#
#=======================================

B0412.1a.1_se   5148 TTGAAACATTTTAATGAATGATATCAGTTTTTTCTTACAATAAGTGAATA   5197
                     ||.||..|.||||||.||..|..||  ||||..||...||.|.....|..
dac/DACH1         48 TTTAATTAATTTAATAAACAAATTC--TTTTCGCTCTAAAAACTCTCAAG     95

B0412.1a.1_se   5198 TGTTTTGAACGGATAGGGCGAATTTTTGATTGATATAACAACAAAAAAAA   5247
                     |||.|.||...|||..|.....|||||...|....||  ||.|||.||.|
dac/DACH1         96 TGTATCGATACGATGCGTTTCTTTTTTTCCTTCGTTA--AATAAATAATA    143

B0412.1a.1_se   5248 ACTACGTAGCAAAAATAATTCAAAAAATAAGTTTTCAGGCGAAATACAAG   5297
                     ||.|...|  |||||.||..||||||.||.|     |||.||||    ||
dac/DACH1        144 ACCAAAAA--AAAAAAAAACCAAAAAGTAGG-----AGGAGAAA----AG    182

B0412.1a.1_se   5298 TTCTT-CGAGAGAGTTTTCACAAGA-TTGTG   5326
                     ||.|| |.|.||..||||.|..|.| |||||
dac/DACH1        183 TTATTGCCATAGTTTTTTTATTATACTTGTG    213
```

→ **below the threshold criteria**

B

```
# Aligned_sequences: 2
# 1: B0412.1a.1_antisense
# 2: dac/DACH1
# Matrix: EDNAFULL
# Gap_penalty: 16
# Extend_penalty: 4
#
# Length: 60
# Identity:      42/60 (70.0%)
# Similarity:    42/60 (70.0%)
# Gaps:           2/60 ( 3.3%)
# Score: 114
#
#
#=======================================

B0412.1a.1_an   3662 TTAATAAAAATAATAAAAACAAAATTGCAGTATAAAAAAGTAGGAAAA-A   3710
                     |||| |.|||||||||..|.||||....|..|..|||||||||||..| |
dac/DACH1        130 TTAA-ATAAATAATAACCAAAAAAAAAAAAAACCAAAAAGTAGGAGGAGA    178

B0412.1a.1_an   3711 AAATTTTTTG   3720
                     |||.||.|||
dac/DACH1        179 AAAGTTATTG    188
```

→ **Hit:**
>B0412.1a.1_antisense_CRE-like
TTAATAAAAATAATAAAAACAAAATTGCAGTATAAAAAAGTAGGAAAAAAAAATTTTTTG

→ **BLASTN SEARCH**
Note: zero results when blasting the sequence on Ensembl against the C. elegans genome. When including +4bp on each side, locatable via Blast and situated on an intron at e-value of 3.8e-31:

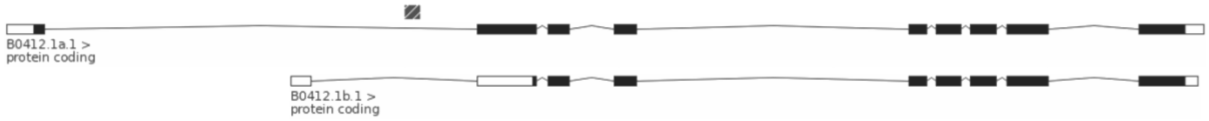

→ **Hit!**

12. Priapulus caudatus

Genome accession incomplete.

dac/DACH1 CRE search in non-neural genes

Shown are the results of EMBOSS matcher sequence comparisons between the superconserved *dachshund/DACH1* CRE identified by ref. 8 and the non-neural genes *Brachyury* (1), *GATA* (2) and *Twist* (3) for key species. A match meeting the cutoff criteria of 60% sequence identity and 55bp length was blasted using Ensembl Blastn to identify its e-value (cutoff: 1e-1) and its position (criteria: intronic position).

1. *Brachyury*

1.1 Nematostella vectensis

```
Gene-ID: 116616126

# Aligned_sequences: 2
# 1: dac_DACH1
# 2: 1015147-1021296
# Matrix: EDNAFULL
# Gap_penalty: 16
# Extend_penalty: 4
#
# Length: 183
# Identity:     103/183 (56.3%)
# Similarity:   103/183 (56.3%)
# Gaps:          19/183 (10.4%)
# Score: 123
#
#
#=======================================

dac_DACH1         42 TTCGAGTTTAATTAATTTAAT-AAACAAATTCTTTTCGCTCTAAAAACTC     90
                     ||.|.||..||..|||||||. |||.|||.|...||||..|.||..||.|
1015147-10212   5845 TTAGTGTACAAGAAATTTAAGGAAAGAAAATAAATTCGTCCAAATGACGC   5894

dac_DACH1         91 TCAA------GTGTATCGATA-CGA---TGCGTTTCTTTTTTTCCTTCGT    130
                     |.||      ||..||.|||| |||   |.||..|.|||...|..||  |
1015147-10212   5895 TTAACAAATTGTTAATAGATATCGAAAATACGAATATTTAAATGATT--T   5942

dac_DACH1        131 TAAATAAATAATAACCAAA------AAAAAAAAAAACCAAAAAGTAGGAG    174
                     ||.|.||..||||||...|      |.|||||||....|||.|.||...|
1015147-10212   5943 TACAAAAGAAATAACTCGATGATTTAGAAAAAAATTGTAAATATTATCTG   5992

dac_DACH1        175 GAGAAAAGTTATTGCCATAGTTTTTTTATTATA    207
                     ...|||...|.||..|||.|||.|||.|||.||
1015147-10212   5993 TTCAAATCATTTTTGCATTGTTCTTTGATTCTA   6025
```

→ below the threshold criteria

1.2 *Saccoglossus kowalvskii*

```
Gene-ID: 100303461

# Aligned_sequences: 2
# 1: dac_DACH1
# 2: c349884-344800
# Matrix: EDNAFULL
# Gap_penalty: 16
# Extend_penalty: 4
#
# Length: 150
# Identity:      88/150 (58.7%)
# Similarity:    88/150 (58.7%)
# Gaps:          24/150 (16.0%)
# Score: 108
#
#
#=======================================

dac_DACH1          4 TTTCGACTTCCGCCATTCGAGGCTCGCCCAATTTCCGTTTCGAGTTTAAT     53
                     ||.|||||||..||...||||  |.|..|.|||||.||||..||.|||.|
c349884-34480   3197 TTACGACTTCGCCCGAACGAG--TGGTACTATTTCAGTTTTCAGATTATT   3244

dac_DACH1         54 -TAATT-----------TAATAAACAAATTCTTTTCGCT--CTAAAAACT     89
                      |||||           |.||.||.||||.|...|.||.  |||.|||..
c349884-34480   3245 ATAATTCGACTTCGTTATGATTAAAAAATACAAGTTGCATACTACAAATG   3294

dac_DACH1         90 CT----CAAGTGTATCGATACGATGCGTTTC---TTTTTTTCCTTCGTTA    132
                     |.    |.|.|.|.||.|.|| |..||||||   |||.|||||||.||||
c349884-34480   3295 CAACTACTACTATTTCCACAC-ACCCGTTTCCTGTTTGTTTCCTTAGTTA   3343
```

→ below the threshold criteria

1.3 *Ciona intestinalis*

```
Gene-ID: 778911

# Aligned_sequences: 2
# 1: dac_DACH1
# 2: 6264-8022
# Matrix: EDNAFULL
# Gap_penalty: 16
# Extend_penalty: 4
#
# Length: 66
# Identity:      39/66 (59.1%)
# Similarity:    39/66 (59.1%)
# Gaps:           2/66 ( 3.0%)
# Score: 75
#
#
#=======================================

dac_DACH1         96 TGTATCGATACGATGCGTTTCTTTTTTTCCTTCGTTAAATAAATAATAAC    145
                     |||..||..||.|||||..|.|..||.||..|.|||...|.||  |||||
6264-8022       1690 TGTGACGTCACAATGCGAATATAATTATCGATTGTTTCGTGAA--ATAAC   1737

dac_DACH1        146 CAAAAAAAAAAAAAAC    161
                     ...||||.|..||.||
6264-8022       1738 ATTAAAATATGAACAC   1753
```

→ below the threshold criteria

1.4 Lingula anatina

```
Gene-ID: 106155248

# Aligned_sequences: 2
# 1: dac_DACH1
# 2: 1426532-1435256
# Matrix: EDNAFULL
# Gap_penalty: 16
# Extend_penalty: 4
#
# Length: 125
# Identity:      72/125 (57.6%)
# Similarity:    72/125 (57.6%)
# Gaps:           5/125 ( 4.0%)
# Score: 100
#
#
#=======================================

dac_DACH1         48 TTTAATTAATTTAATAAACAAATTCTTTTCGCTCTAAAAACTCTCAAGTG     97
                     |.||.|.|||.|||...| ||||..|.|....|.|...||||.|...|..
1426532-14352   8515 TATATTAAATATAACGTA-AAATAGTATCAAATATTGCAACTTTGTGGAT   8563

dac_DACH1         98 TATCGATACGATGCGTTTCTTTTTTTCCTTCGTTAAATAAATAATAACCA    147
                     |||.||| |||..|||.||||...||......|||..  ||||||..|||
1426532-14352   8564 TATGGAT-CGAATCGTGTCTTACATTGAAATTTTAGC--AATAATCCCCA   8610

dac_DACH1        148 AAAAAAAAAA-AAACCAAAAAGTAG    171
                     ||...|||.| |||..||||||.||
1426532-14352   8611 AAGGTAAACACAAATTAAAAAGAAG   8635
```

→ **below the threshold criteria**

1.5 Octopus bimaculoides

```
Gene-ID: 106883344

# Aligned_sequences: 2
# 1: dac_DACH1
# 2: 25437-45149
# Matrix: EDNAFULL
# Gap_penalty: 16
# Extend_penalty: 4
#
# Length: 115
# Identity:      67/115 (58.3%)
# Similarity:    67/115 (58.3%)
# Gaps:           5/115 ( 4.3%)
# Score: 107
#
#
#=======================================

dac_DACH1         88 CTCTCAAGTGTATCGATACGATGCGTTTCTTTTTTTCCT--TCGTTAAAT    135
                     |||.|.|.|||||  ||....|.|.|.|||||..|.|.|  ||..|||||
25437-45149    17099 CTCACCAATGTAT--ATCATGTTCTTGTCTTTACTCCGTGTTCTATAAAT  17146

dac_DACH1        136 AAATAATAACCAAAAAAAAAAAAAAACCAAA-AAGTAGGAGGAGAAAAGTT    184
                     ||||.||||...|..|.|...|..|||||| ||..||.|....|||.|..
25437-45149    17147 AAATTATAAATGATTACACGCAGTACCAAAGAAACAGAATATTAAAGGGA  17196

dac_DACH1        185 ATTGCCATAGTTTTT    199
                     |..|.|||.||.|||
25437-45149    17197 AAGGGCATGGTATTT  17211
```

→ **below the threshold criteria**

1.6 Schistosoma mansoni

```
Gene-ID: 4609

# Aligned_sequences: 2
# 1: dac_DACH1
# 2: 127735434-127742951
# Matrix: EDNAFULL
# Gap_penalty: 16
# Extend_penalty: 4
#
# Length: 110
# Identity:      66/110 (60.0%)
# Similarity:    66/110 (60.0%)
# Gaps:          11/110 (10.0%)
# Score: 94
#
#
#=======================================

dac_DACH1         48 TTTAATTAATTTA-ATAAACAAATTCTTTTCGCTCTAAAAACTCTCAAGT     96
                     |||||...||||. ||..|..||||.||||     ||||||.|.|.|||
127735434-127   5726 TTTAACAGATTTGTATTTAAGAATTGTTTT-----TAAAAAATTTTAAG-   5769

dac_DACH1         97 GTATCGATACGATGCGTTTCTTTTTTTCCTTCG-TTAAATAAATAATAAC    145
                       ||..|.||.|||..|.|||.|...|..|.|. ||||||..| ||||||
127735434-127   5770 --ATTTACACAATGTTTCTCTGTAAATATTGCCATTAAATGTA-AATAAC   5816

dac_DACH1        146 CAAAAAAAAA    155
                     ...||.||||
127735434-127   5817 TTTAATAAAA   5826
```

→ sequence cannot be located in Blast search

1.7 Adineta vaga

```
Gene-ID: GSADVG00063359001

# Aligned_sequences: 2
# 1: dac_DACH1
# 2: HG381423
# Matrix: EDNAFULL
# Gap_penalty: 16
# Extend_penalty: 4
#
# Length: 41
# Identity:      29/41 (70.7%)
# Similarity:    29/41 (70.7%)
# Gaps:           2/41 ( 4.9%)
# Score: 85
#
#
#=======================================

dac_DACH1        103 ATACGATGCGTTTCTTTTTTTCCTTCGTTAAATAAATAATA    143
                     |||||  ||.|.||||||.|||.||..||.|||||..||.|
HG381423         219 ATACG--GCATATCTTTTATTCTTTGTTTGAATAACGAAGA    257
```

→ below the threshold criteria

1.8 Hypsibius exemplaris

```
Gene-ID: BV898_01477

# Aligned_sequences: 2
# 1: dac_DACH1
# 2: MTYJ01000005.1
# Matrix: EDNAFULL
# Gap_penalty: 16
# Extend_penalty: 4
#
# Length: 73
# Identity:      46/73 (63.0%)
# Similarity:    46/73 (63.0%)
# Gaps:           7/73 ( 9.6%)
# Score: 110
#
#
#=======================================

dac_DACH1        161 CCAAAAAGTAGGAGGAGAAAAGTTATTGCCATAGTTTTTT-------TAT    203
                     |.|||.||||.|...|.|||..||..|..|.|||||.|||       |.|
MTYJ01000005.   2156 CTAAACAGTAAGCACAAAAATCTTCCTCTCTTAGTTATTTCCTACATTTT   2205

dac_DACH1        204 TATACTTGTGTGTTTACCTTTCT    226
                     |||..||||.|||||.|||||||
MTYJ01000005.   2206 TATGGTTGTCTGTTTGCCTTTCT   2228
```

→ **does not meet criteria:** does not lie in intronic region

1.9 Priapulus caudatus

```
Gene-ID: 106811534

# Aligned_sequences: 2
# 1: dac_DACH1
# 2: 79967-89147
# Matrix: EDNAFULL
# Gap_penalty: 16
# Extend_penalty: 4
#
# Length: 96
# Identity:      60/96 (62.5%)
# Similarity:    60/96 (62.5%)
# Gaps:           6/96 ( 6.2%)
# Score: 108
#
#
#=======================================

dac_DACH1         32 CAATTTCCGTTTCG-AGTTTAATTAATTTAATAAACAAATTCTTTTCGCT     80
                     ||.|||.|.|.||| ||...|||||||.||.||||.||..|.|.||   |
79967-89147     6286 CATTTTGCATGTCGTAGCGAAATTAATATATTAAATAATATTTATT---T   6332

dac_DACH1         81 CTAAAAACTCTCAAGTGTATCGATACGATGC-GTTTCTTTTTTTCC    125
                     .|.|||||. |.|..|.|||...||..||.| ||.|.|||||||.|
79967-89147     6333 TTTAAAACA-TGATTTATATATTTATTATACTGTATATTTTTTTGC   6377
```

→ **does not meet criteria:** does not lie in intronic region

2. *GATA*

2.1 Nematostella vectensis

```
Gene-ID: 5521983

# Aligned_sequences: 2
# 1: dac_DACH1
# 2: NEMVEscaffold_2
# Matrix: EDNAFULL
# Gap_penalty: 16
# Extend_penalty: 4
#
# Length: 48
# Identity:      35/48 (72.9%)
# Similarity:    35/48 (72.9%)
# Gaps:           1/48 ( 2.1%)
# Score: 111
#
#
#=======================================

dac_DACH1         58 TTAATAAACAAATTCTTTTCGCTCTAAAAACTCTCAAGTGTATCGATA    105
                     |.|.|.|||||.||||||.|..|.|||||||.|.|| |||.|||.|||
NEMVEscaffold   1557 TAAGTCAACAAGTTCTTTGCTTTGTAAAAACGCACA-GTGGATCAATA   1603
```

→ below the threshold criteria

2.2 Saccoglossus kowalevskii

```
Gene-ID: 102809317

# Aligned_sequences: 2
# 1: dac_DACH1
# 2: ACQM01128928.1
# Matrix: EDNAFULL
# Gap_penalty: 16
# Extend_penalty: 4
#
# Length: 35
# Identity:      24/35 (68.6%)
# Similarity:    24/35 (68.6%)
# Gaps:           0/35 ( 0.0%)
# Score: 76
#
#
#=======================================

dac_DACH1         51 AATTAATTTAATAAACAAATTCTTTTCGCTCTAAA     85
                     |||.||...||.|||.|.|||||||...|||||.|
ACQM01128928.   2067 AATAAAAGAAAGAAATATATTCTTTGTTCTCTACA   2101
```

→ below the threshold criteria

2.3 *Ciona intestinalis*

```
Gene-ID: 779004

# Aligned_sequences: 2
# 1: dac_DACH1
# 2: HT001157.1
# Matrix: EDNAFULL
# Gap_penalty: 16
# Extend_penalty: 4
#
# Length: 127
# Identity:      73/127 (57.5%)
# Similarity:    73/127 (57.5%)
# Gaps:           4/127 ( 3.1%)
# Score: 125
#
#
#=======================================

dac_DACH1         48 TTTAATTAATTTAATAAACAAATTCTTTT---CGCTCTAAAAACTCTCAA     94
                     |||.||||.|..|||....||.||||.||   |...|.|.|.|.|.|.||
HT001157.1      4043 TTTTATTATTAAAATGTTAAACTTCTGTTAAACTTACGAGACAATATAAA   4092

dac_DACH1         95 GTGTATCGATACGATGCGTTTCTTTTTTTCCTTCGTTAAATAAATAATAA    144
                     .||..|..|||..||.||.|..||..|||..||.....|||||| |.|..
HT001157.1      4093 TTGCTTTAATATTATTCGCTGTTTAATTTTTTTGAAAGAATAAA-AGTGT   4141

dac_DACH1        145 CCAAAAAAAAAAAAAAACCAAAAAGTAG    171
                     ..|.|||.|||||||.|.||||.||.|
HT001157.1      4142 TGACAAATAAAAAAAGCTAAAACGTGG   4168
```

→ below the threshold criteria

2.4 *Lingula anatina*

```
Gene-ID: 106172844

# Aligned_sequences: 2
# 1: dac_DACH1
# 2: LFEI02000418.1
# Matrix: EDNAFULL
# Gap_penalty: 16
# Extend_penalty: 4
#
# Length: 142
# Identity:      81/142 (57.0%)
# Similarity:    81/142 (57.0%)
# Gaps:          15/142 (10.6%)
# Score: 113
#
#
#=======================================

dac_DACH1         55 AATTTAATAAACAAATTCTTTTC----GCTCTAA---AAACTCTCAAGTG     97
                     |||||||.|||.|||||.||||.    |.|..||   |.|.|||..|.|.
LFEI02000418.  20147 AATTTAAAAAAAAAATTTTTTAATTAGGTTGAAGCAATAATCTAGATTT  20196

dac_DACH1         98 TATCGAT--ACGATGCGTTTCTTTTTTTCCTTCGTTAAATAAATAATAAC    145
                     |.|..||  ||||..|..|..|..||.||.||||.|..|||.|.||    
LFEI02000418.  20197 TTTTAATCGACGAGACACTCATAATTGTCTTTCGCTTGATAGAGAA----  20242

dac_DACH1        146 CAAAAAAAAAAAAAAACCAAAAAGTAGGAGGAGAAAAGTTATT    187
                       ||||..|||..||.|..|||.|..||..|..||||||.||
LFEI02000418.  20243 --AAAATTAAATGAATCTTAAAATTTGAAAAATAAAGTTTTT  20282
```

→ below the threshold criteria

2.5 Octopus bimaculoides

Gene-ID: 106868722

```
# Aligned_sequences: 2
# 1: dac_DACH1
# 2: KQ417342.1
# Matrix: EDNAFULL
# Gap_penalty: 16
# Extend_penalty: 4
#
# Length: 98
# Identity:      61/98 (62.2%)
# Similarity:    61/98 (62.2%)
# Gaps:           6/98 ( 6.1%)
# Score: 121
#
#
#=======================================

dac_DACH1         82 TAAAAACTCTC--AAGTGTA-TCGATACGAT---GCGTTTCTTTTTTTCC    125
                     |.||.|||||.  |.||||| ||..||..||   ||||.||.||.|||..
KQ417342.1     80133 TGAATACTCTTCTATGTGTAATCCTTATTATAACGCGTGTCCTTCTTTTG  80182

dac_DACH1        126 TTCGTTAAATAAATAATAACCAAAAAAAAAAAAAACCAAAAAGTAGGA    173
                     |||.||.||...|||||.|...|.|.||.||.....|||||||||.||
KQ417342.1     80183 TTCCTTTAAGTGATAATGAAATATATAATAATGCTACAAAAAGTACGA  80230
```

→ **does not meet criteria:** does not lie in intronic region

2.6 Schistosoma mansoni

Gene-ID: 8354299

```
# Aligned_sequences: 2
# 1: dac_DACH1
# 2: SM_V7_1
# Matrix: EDNAFULL
# Gap_penalty: 16
# Extend_penalty: 4
#
# Length: 127
# Identity:      73/127 (57.5%)
# Similarity:    73/127 (57.5%)
# Gaps:           2/127 ( 1.6%)
# Score: 125
#
#
#=======================================

dac_DACH1         34 ATTTCCGTTTCGAGTTTAATTAATTTAATAAACAAATTCTTTTCGCTCTA     83
                     |.||.|.|||..|.|.|||.||||.|||||| |.||||..||....|...
SM_V7_1        39929 ACTTTCATTTTAATTATAAGTAATATAATAA-CTAATTAATTATATTAAT  39977

dac_DACH1         84 AAAACTCTCAAGTGTATCGATACGATGCGTTTCTTTTTTTCCTTCGTTAA    133
                     .|||||.| |.|..|||..||..|.....||..||..|..||||...|..
SM_V7_1        39978 TAAACTAT-AGGAATATATATGTGTATATTTGGTTGATAACCTTATGTCT  40026

dac_DACH1        134 ATAAATAATAACCAAAAAAAAAAAAAA    160
                     ||.|.|||...|||||.||.||.||||
SM_V7_1        40027 ATCATTAAACGCCAAATAATAAGAAAA  40053
```

→ **below the threshold criteria**

2.7 Adineta vaga

Gene-ID: GSADVG00055891001

```
# Aligned_sequences: 2
# 1: dac_DACH1
# 2: HG381205
# Matrix: EDNAFULL
# Gap_penalty: 16
# Extend_penalty: 4
#
# Length: 136
# Identity:      80/136 (58.8%)
# Similarity:    80/136 (58.8%)
# Gaps:          16/136 (11.8%)
# Score: 104
#
#
#=======================================

dac_DACH1         98 TATCGATACGATGCGTTTCTTTTTTTCCTTCGTTAAATAAATA---ATAA    144
                     ||||.||...||||.|.|..|.|||   .||.|.||||.|||.   |.||
HG381205        1501 TATCTATGTAATGCTTGTGGTCTTT---ATCATAAAATGAATGGACACAA   1547

dac_DACH1        145 CCAAAAAAAAAAAAAACCAAAAAG-----TAGGAGGAGAAAAGTTATTGC    189
                     .|.|..|...|..|||||.|||.|     |||.|  ||||||  |||.|.
HG381205        1548 TCGACCATTGATTAAACCTAAACGACGTTTAGTA--AGAAAA--TATAGT   1593

dac_DACH1        190 CATAGTTTTTTTATTATACTTGTGTGTTTACCTTTC    225
                     ||.|.|||..||||.|| ||..|.|.|||..|||||
HG381205        1594 CAAATTTTGATTATCAT-CTATTTTCTTTTTCTTTC   1628
```

→ below the threshold criteria

2.8 Hypsibius exemplaris

Gene-ID: BV898_01252

```
# Aligned_sequences: 2
# 1: dac_DACH1
# 2: MTYJ01000004.1
# Matrix: EDNAFULL
# Gap_penalty: 16
# Extend_penalty: 4
#
# Length: 33
# Identity:      24/33 (72.7%)
# Similarity:    24/33 (72.7%)
# Gaps:           0/33 ( 0.0%)
# Score: 84
#
#
#=======================================

dac_DACH1        195 TTTTTTTATTATACTTGTGTGTTTACCTTTCTG    227
                     |.|.|.|||.||||||||.|||.||.||.|.||
MTYJ01000004.   3155 TGTCTGTATCATACTTGTCTGTCTAGCTCTATG   3187
```

→ below the threshold criteria

2.9 Priapulus caudatus

```
Gene-ID: 106819002

# Aligned_sequences: 2
# 1: dac_DACH1
# 2: KQ717601.1
# Matrix: EDNAFULL
# Gap_penalty: 16
# Extend_penalty: 4
#
# Length: 43
# Identity:      35/43 (81.4%)
# Similarity:    35/43 (81.4%)
# Gaps:           2/43 ( 4.7%)
# Score: 119
#
#
#=======================================

dac_DACH1        131 TAAATAAATAA-TAACCAAAAAAAAAAA-AAACCAAAAAGTAG    171
                     ||||||||||| |||..|||.|||.||| ||||.||.||||||
KQ717601.1     11725 TAAATAAATAAATAAATAAATAAATAAATAAACGAATAAGTAG  11767
```

→ below the threshold criteria

3. *Twist*

3.1 Nematostella vectensis

```
Gene-ID: 5514773

# Aligned_sequences: 2
# 1: dac_DACH1
# 2: NEMVEscaffold_53
# Matrix: EDNAFULL
# Gap_penalty: 16
# Extend_penalty: 4
#
# Length: 19
# Identity:      17/19 (89.5%)
# Similarity:    17/19 (89.5%)
# Gaps:           0/19 ( 0.0%)
# Score: 77
#
#
#=======================================

dac_DACH1        138 ATAATAACCAAAAAAAAAA    156
                     ||||.||||||||.|||||
NEMVEscaffold    635 ATAACAACCAAAAGAAAAA    653
```

→ below the threshold criteria

3.2 Saccoglossus kowalevskii

```
Gene-ID: 100303576

# Aligned_sequences: 2
# 1: dac_DACH1
# 2: GL016116.1
# Matrix: EDNAFULL
# Gap_penalty: 16
# Extend_penalty: 4
#
# Length: 40
# Identity:      31/40 (77.5%)
# Similarity:    31/40 (77.5%)
# Gaps:           1/40 ( 2.5%)
# Score: 107
#
#
#=======================================

dac_DACH1        131 TAAATAAATAA-TAACCAAAAAAAAAAAAAACCAAAAAGT    169
                     ||||||||||| |||..|||.|||.|||.||..|||.|||
GL016116.1      2879 TAAATAAATAAATAAATAAATAAATAAATAAATAAATAGT   2918
```

→ below the threshold criteria

3.3 Ciona intestinalis

```
Gene-ID: 448998

# Aligned_sequences: 2
# 1: dac_DACH1
# 2: c2028690-2027376
# Matrix: EDNAFULL
# Gap_penalty: 16
# Extend_penalty: 4
#
# Length: 37
# Identity:      25/37 (67.6%)
# Similarity:    25/37 (67.6%)
# Gaps:           1/37 ( 2.7%)
# Score: 65
#
#
#=======================================

dac_DACH1         41 TTTCGAGTTTAATTAATTTAATAAACAA-ATTCTTTT     76
                     |||.|..|||..|||||.|..|||||.| |||.|.||
c2028690-2027   1085 TTTTGTTTTTTTTTAATATTCTAAACCATATTATGTT   1121
```

→ **below the threshold criteria**

3.4 Lingula anatina

```
Gene-ID: 106181138

# Aligned_sequences: 2
# 1: dac_DACH1
# 2: LFEI02000007.1
# Matrix: EDNAFULL
# Gap_penalty: 16
# Extend_penalty: 4
#
# Length: 27
# Identity:      24/27 (88.9%)
# Similarity:    24/27 (88.9%)
# Gaps:           2/27 ( 7.4%)
# Score: 96
#
#
#=======================================

dac_DACH1        145 CCAAAAAAAAAAAAAACCAAAAAGTAG    171
                     |||||||||||||||  |||||.|||
LFEI02000007.  11817 CCAAAAAAAAAAAAAA--AAAAAATAG  11841
```

→ **below the threshold criteria**

3.5 Octopus bimaculoides

```
Gene-ID: 106871027

# Aligned_sequences: 2
# 1: dac_DACH1
# 2: KQ418303.1
# Matrix: EDNAFULL
# Gap_penalty: 16
# Extend_penalty: 4
#
# Length: 170
# Identity:      95/170 (55.9%)
# Similarity:    95/170 (55.9%)
# Gaps:          11/170 ( 6.5%)
# Score: 127
#
#
#=======================================

dac_DACH1         54 TAATTTAATAAACAAATTCTTT---TCGCTCTAAAA----ACTCTCAAGT     96
                     |||.|||||..|||.|||.|..   |...|.|||.|    |||.|..|.|
KQ418303.1      2002 TAAATTAATGTACAGATTTTAAAAATGCATTTAATATGACACTTTTTAAT   2051

dac_DACH1         97 GTATCGATACGATGCGTTTCTTTTTTTCC--TTCGTTAAATAAATAATAA    144
                     |.|..||||...||..|||.|||||||..  |||.|.|||||.|..||||
KQ418303.1      2052 GGACTGATAAAGTGTTTTTTTTTTTTTTTAGTTCTTGAAATATACTATAA   2101

dac_DACH1        145 CCAAAAAAAAAAAAAACCAAAAAGTAGGAGGAGAAAAGTTA--TTGCCAT    192
                     ..|.||.||.|.|.|...|.|.|.|..|...|.|.||.|.|  ||....|
KQ418303.1      2102 ATATAAGAATATATATATATATATTTTGGATACATAATTCAAGTTATTTT   2151

dac_DACH1        193 AGTTTTTTTATTATACTTGT    212
                     |||||||.|.|.||..|.||
KQ418303.1      2152 AGTTTTTATTTCATCATCGT   2171
```

→ below the threshold criteria

3.6 Schistosoma mansoni

```
Gene-ID: 8354110

# Aligned_sequences: 2
# 1: dac_DACH1
# 2: c29125361-29124846
# Matrix: EDNAFULL
# Gap_penalty: 16
# Extend_penalty: 4
#
# Length: 33
# Identity:      24/33 (72.7%)
# Similarity:    24/33 (72.7%)
# Gaps:           2/33 ( 6.1%)
# Score: 60
#
#
#=======================================

dac_DACH1         46 AGTTTAATTAATTT-AATAAA-CAAATTCTTTT     76
                     |.||||.|.|..|| ||||.| ||||||.||||
c29125361-291    178 AATTTATTGATGTTCAATAGATCAAATTTTTTT    210
```

→ below the threshold criteria

3.7 Adineta vaga

```
Gene-ID: GSADVG00018249001

# Aligned_sequences: 2
# 1: dac_DACH1
# 2: HG380836
# Matrix: EDNAFULL
# Gap_penalty: 16
# Extend_penalty: 4
#
# Length: 79
# Identity:      51/79 (64.6%)
# Similarity:    51/79 (64.6%)
# Gaps:           3/79 ( 3.8%)
# Score: 107
#
#
#=======================================

dac_DACH1         73 TTTTCGCTCTAAAAACTCTCAAGTGTATCGATACGATGCG-TTTCTTTTT    121
                     |.||||| ||.|..|.|.|||||...||..|.|.|...|| |||||||.|
HG380836        1156 TATTCGC-CTGATGAATATCAAGACAATTAACATGTGTCGATTTCTTTCT   1204

dac_DACH1        122 TTCCTTCGTTAAATAAATAATAACCAAAA    150
                     |||.|||.||..| ||.||||||..|.||
HG380836        1205 TTCTTTCTTTTTA-AACTAATAATTATAA   1232
```

→ **does not meet criteria:** does not lie in intronic region

3.8 Hypsibius exemplaris

```
Gene-ID: BV898_04819

# Aligned_sequences: 2
# 1: dac_DACH1
# 2: MTYJ01000024.1
# Matrix: EDNAFULL
# Gap_penalty: 16
# Extend_penalty: 4
#
# Length: 69
# Identity:      39/69 (56.5%)
# Similarity:    39/69 (56.5%)
# Gaps:           0/69 ( 0.0%)
# Score: 75
#
#
#=======================================

dac_DACH1        118 TTTTTTCCTTCGTTAAATAAATAATAACCAAAAAAAAAAAAAAACCAAAAA    167
                     ||.||||.|||....|.|.||.||.||.|....|.|||||....|||.||
MTYJ01000024.   2348 TTCTTTCATTCTGAGAGTCAACAAAAATCGTCGACAAAAATCCTCAACAA   2397

dac_DACH1        168 GTAGGAGGAGAAAAGTTAT    186
                     ....|..||.||||.||.|
MTYJ01000024.   2398 AATCGTCGACAAAAATTGT   2416
```

→ **below the threshold criteria**

3.9 Priapulus caudatus

```
Gene-ID: 106807790

# Aligned_sequences: 2
# 1: dac_DACH1
# 2: KQ715116.1
# Matrix: EDNAFULL
# Gap_penalty: 16
# Extend_penalty: 4
#
# Length: 47
# Identity:      32/47 (68.1%)
# Similarity:    32/47 (68.1%)
# Gaps:           1/47 ( 2.1%)
# Score: 88
#
#
#=======================================

dac_DACH1        114 TTCTTTTTTTCCTTCGTTAAATAAATAATAACCAAAAAAAAAAAAAA    160
                     |||||||.|.||.. ||||..|.|||||||||...||.|.|.|||.|
KQ715116.1      2547 TTCTTTTATGCCGA-GTTATTTCAATAATAACGTGAATATATAAATA   2592
```

→ **below the threshold criteria**

Supplementary Data Set S5

invected/ENGRAILED-2

Shown are the results of EMBOSS matcher sequence comparisons between the superconserved *invected/ENGRAILED-2* CRE identified in ref. 8 and the respective *invected/ENGRAILED-2* homolog of each species. A match meeting the Cutoff criteria of 60% sequence identity and 55bp length was blasted using Ensembl Blastn to identify its e-value (cutoff: 1e-1). Both upstream and downstream sense strand were screened.

1. *Strongylocentrotus purpuratus*

Description: engrailed-like
Gene: **LOC115929921**
Transcript: XM_031000060
Product: XP_030855920

Upstream

```
# Aligned_sequences: 2
# 1: LOC115929921_US
# 2: inv/EN2
# Matrix: EDNAFULL
# Gap_penalty: 16
# Extend_penalty: 4
#
# Length: 55
# Identity:      37/55 (67.3%)
# Similarity:    37/55 (67.3%)
# Gaps:           2/55 ( 3.6%)
# Score: 89
#
#
#=======================================

LOC115929921_   4223 ACTCGAAAGGCCCAGCGCTTCTCCA-TAACCTAATCCC-ATCACTTATAT   4270
                     |||..||||||..||.|...||..| ||..|||||||| |..||..||||
inv/EN2          177 ACTAAAAAGGCAAAGGGAGACTTAAGTATGCTAATCCCCAGGACAAATAT    226

LOC115929921_   4271 ATTTT   4275
                     |||||
inv/EN2          227 ATTTT    231
```

→ **Hit:**
>LOC115929921_US_CRE-like
ACTCGAAAGGCCCAGCGCTTCTCCATAACCTAATCCCATCACTTATATATTTT

→ **BLASTN SEARCH** for LOC115929921_US_CRE-like: e-value: 5.4e-22

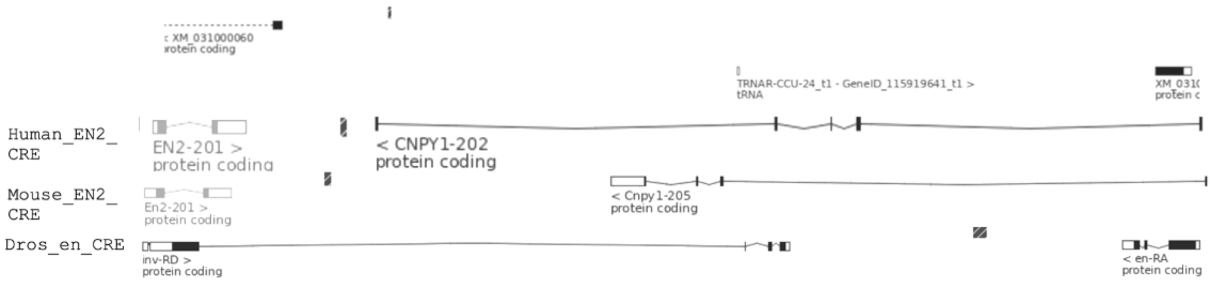

Downstream

```
# Aligned_sequences: 2
# 1: LOC115929921_DS
# 2: inv/EN2
# Matrix: EDNAFULL
# Gap_penalty: 16
# Extend_penalty: 4
#
# Length: 77
# Identity:      48/77 (62.3%)
# Similarity:    48/77 (62.3%)
# Gaps:           3/77 ( 3.9%)
# Score: 100
#
#
#=======================================

LOC115929921_  13065 ATCATTGTGCAATAATACTATTTAATCTCCTGAGA-ATTTGAAAATCATA  13113
                     |.|.|||||....||...||.||||.||..||||| ||.|.|||.....|
inv/EN2           30 AGCTTTGTGGCGGAAATATAATTAAACTGGTGAGAGATGTAAAAGATGAA     79

LOC115929921_  13114 GA--GGGGTAACATCTGAGTAATTTGA  13138
                     ||  |||.|.|||||.||.|.||||.|
inv/EN2           80 GAATGGGATGACATCAGACTGATTTCA    106
```

→ **Hit:**

>LOC115929921_DS_CRE-like
ATCATTGTGCAATAATACTATTTAATCTCCTGAGAATTTGAAAATCATAGAGGGGTAACATCTGAGTAATTTGA

→ **BLASTN SEARCH** for LOC115929921_DS_CRE-like: e-value: 2.5e-34

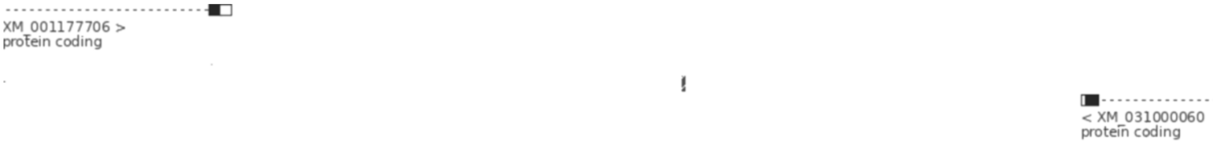

2. *Saccoglossus kowalevskii*

Description: en - engrailed
NCBI Gene: 100303466
Transcript: NM_001164900.1
Product: NP_001158372.1

Upstream

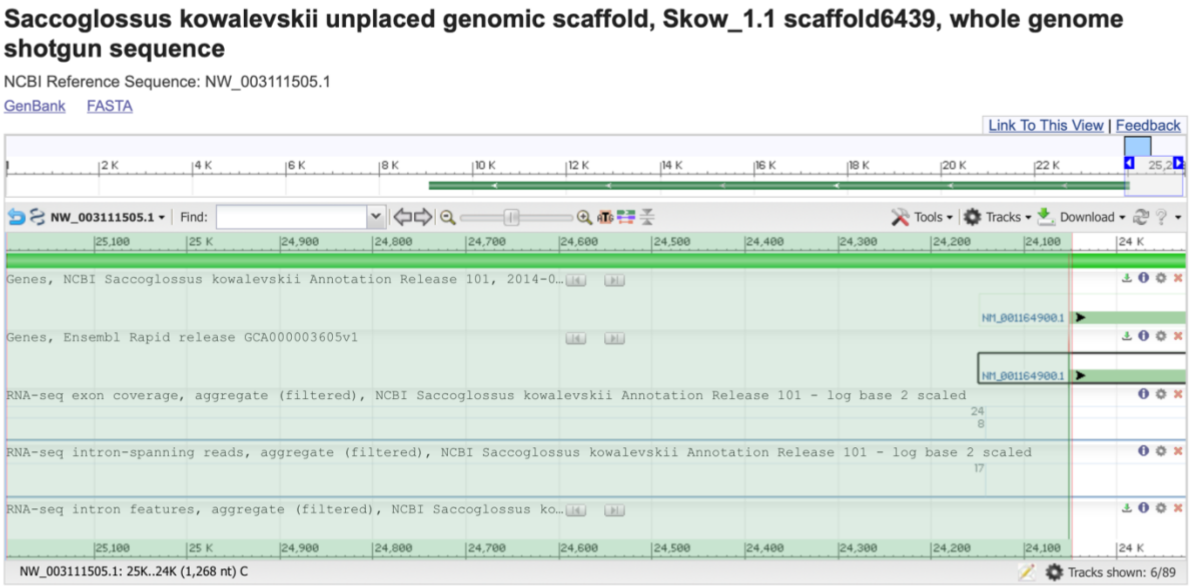


```
# Aligned_sequences: 2
# 1: 100303466_US
# 2: inv/EN2
# Matrix: EDNAFULL
# Gap_penalty: 16
# Extend_penalty: 4
#
# Length: 26
# Identity:      18/26 (69.2%)
# Similarity:    18/26 (69.2%)
# Gaps:           0/26 ( 0.0%)
# Score: 58
#
#
#=======================================

100303466_US      75 CCATTTTTATGATAGGGGGAATTGAG     100
                     ||||||||||..|||.....|.||||
inv/EN2          296 CCATTTTTATCTTAGCTATGAATGAG     321
```

→ **below the threshold criteria**

<u>Downstream</u>

Note: No neighbours annotated on NCBI, see screenshots.

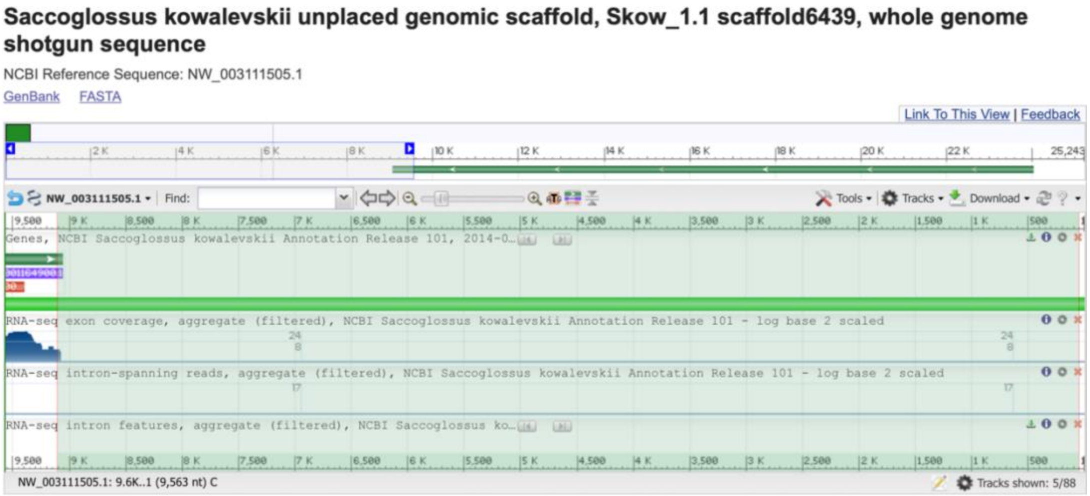


```
# Aligned_sequences: 2
# 1: 100303466_DS
# 2: inv/EN2
# Matrix: EDNAFULL
# Gap_penalty: 16
# Extend_penalty: 4
#
# Length: 42
# Identity:      28/42 (66.7%)
# Similarity:    28/42 (66.7%)
# Gaps:           0/42 ( 0.0%)
# Score: 84
#
#
#=======================================

100303466_DS     5089 AGTTAAATCTTGTTAGCCTAGCAATTTGTACTGCGCAACAGT   5130
                      |.||.|||||||||||..||..|.||..|.||||....||||
inv/EN2           227 ATTTTAATCTTGTTAGAATACAAGTTAATGCTGCAGTGCAGT    268
```

→ below the threshold criteria

3. Branchiostoma lanceolatum

Gene: **BL18701**
Transcript: BL18701_cuf2
Product: BL18701_cuf2

Upstream

```
# Aligned_sequences: 2
# 1: BL18701_US
# 2: inv/EN2
# Matrix: EDNAFULL
# Gap_penalty: 16
# Extend_penalty: 4
#
# Length: 82
# Identity:      52/82 (63.4%)
# Similarity:    52/82 (63.4%)
# Gaps:           5/82 ( 6.1%)
# Score: 92
#
#
#=======================================

BL18701_US     21151 TCCCCGGGACAAG-AGATCTTAATTGTGCGGAGCAAACATGTGTAAGAAT  21199
                     |||||.||||||. |.||.|||||..||.. ||.|.|||.|| |||...|
inv/EN2          211 TCCCCAGGACAAATATATTTTAATCTTGTT-AGAATACAAGT-TAATGCT    258

BL18701_US     21200 GA--TCCAATCGCTTGGCTTGCCCGTGCAGGA  21229
                     |.  |.||.|.|||...||||..|..||||||
inv/EN2          259 GCAGTGCAGTGGCTGAACTTGGTCAGGCAGGA    290
```

→ **Hit:**
>BL18701_US_CRE-like
TCCCCGGGACAAGAGATCTTAATTGTGCGGAGCAAACATGTGTAAGAATGATCCAATCGCTTGGCTTGCCCGTGCAGGA

→ **BLASTN SEARCH** for BL18701_US_CRE-like: e-value 1.5e-37

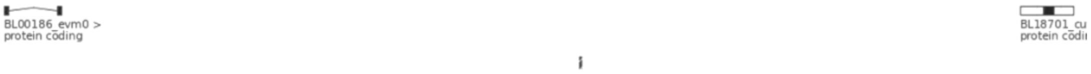

Downstream

```
# Aligned_sequences: 2
# 1: BL18701_DS
# 2: inv/EN2
# Matrix: EDNAFULL
# Gap_penalty: 16
# Extend_penalty: 4
#
# Length: 61
# Identity:      40/61 (65.6%)
# Similarity:    40/61 (65.6%)
# Gaps:           3/61 ( 4.9%)
# Score: 92
#
#
#=======================================

BL18701_DS      4639 ACACATTCATGACTAAGCTATGACTAAGTGTAATACTATTAGTTCTAAAT   4688
                     ||.||||..|..||.||||||||.|.|| ||.|..||.||.|||.||  |
inv/EN2          294 ACCCATTTTTATCTTAGCTATGAATGAG-GTGAAGCTCTTGGTTTTA--T    340

BL18701_DS      4689 TGAACATTATT   4699
                     |.|.||.||.|
inv/EN2          341 TCATCAGTAAT    351
```

→ **Hit:**

```
>BL18701_DS_CRE-like
ACACATTCATGACTAAGCTATGACTAAGTGTAATACTATTAGTTCTAAATTGAACATTATT
```

→ **BLASTN SEARCH** for BL18701_DS_CRE-like: e-value 6.1e-27

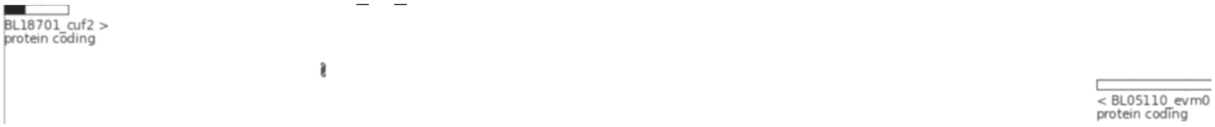

4. Ciona intestinalis

Description: en
Gene: ENSCING00000002157
Transcript: ENSCINT00000004407.3
Product: ENSCINP00000004407

Upstream

```
# Aligned_sequences: 2
# 1: ENSCING00000002157_US
# 2: inv/EN2
# Matrix: EDNAFULL
# Gap_penalty: 16
# Extend_penalty: 4
#
# Length: 35
# Identity:      24/35 (68.6%)
# Similarity:    24/35 (68.6%)
# Gaps:           0/35 ( 0.0%)
# Score: 76
#
#
#=======================================

ENSCING000000   1290 ATATTTCTTTGAATGTTATTTGATTACTACTAAAT   1324
                     |.||.|.|||.|||.||.||.||.|||.|.|.|||
inv/EN2          221 AAATATATTTTAATCTTGTTAGAATACAAGTTAAT    255
```

→ **below the threshold criteria**

Downstream

```
# Aligned_sequences: 2
# 1: ENSCING00000002157_DS
# 2: inv/EN2
# Matrix: EDNAFULL
# Gap_penalty: 16
# Extend_penalty: 4
#
# Length: 65
# Identity:      41/65 (63.1%)
# Similarity:    41/65 (63.1%)
# Gaps:           5/65 ( 7.7%)
# Score: 85
#
#
#=======================================

ENSCING000000   2900 CTGATTAGAGTACATGGTTCGAATTCTTATGTGCAGTAGTTTACCTTACG   2949
                     ||..|||||.||||.|.|   |||.||...|||||||.|.|.|.|||  |
inv/EN2          235 CTTGTTAGAATACAAGTT---AATGCTGCAGTGCAGTGGCTGAACTT--G    279

ENSCING000000   2950 AACAGACACTAGACA   2964
                     ..|||.||..|||.|
inv/EN2          280 GTCAGGCAGGAGAGA    294
```

→ **Hit:**

>ENSCING00000002157_DS_CRE-like

CTGATTAGAGTACATGGTTCGAATTCTTATGTGCAGTAGTTTACCTTACGAACAGACACTAGACA

→ **BLASTN SEARCH** for ENSCING00000002157_DS_CRE-like: e-value 8e-30

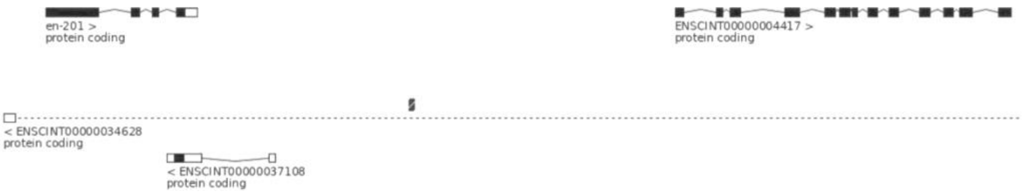

5. Lingula anatina

5.a Gene g20932

Gene: **g20932**
Transcript: g20932.t1
Product: g20932.t1

Upstream

```
# Aligned_sequences: 2
# 1: g20932_US
# 2: inv/EN2
# Matrix: EDNAFULL
# Gap_penalty: 16
# Extend_penalty: 4
#
# Length: 70
# Identity:      43/70 (61.4%)
# Similarity:    43/70 (61.4%)
# Gaps:           3/70 ( 4.3%)
# Score: 83
#
#
#=======================================

g20932_US       2989 TGCAACGCTAGG--GATTAATTTGTTTT-CCACAATGGAATGGCCAAAAG   3035
                     |||.||||.|||  |||.||..||.|.| ||||.|...||..|.||||.|
inv/EN2          143 TGCCACGCAAGGCAGATCAAAGTGCTCTGCCACCACTAAAAAGGCAAAGG    192

g20932_US       3036 AACACGTCATAATACGATTC   3055
                     .|.||.|.|..||.|.|.||
inv/EN2          193 GAGACTTAAGTATGCTAATC    212
```

→ **Hit:**
\>g20932_US_CRE-like
TGCAACGCTAGGGATTAATTTGTTTTCCACAATGGAATGGCCAAAAGAACACGTCATAATACGATTC

→ **BLASTN SEARCH** for g20932_US_CRE-like: e-value: 1.6e-30

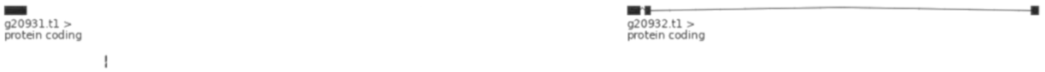

Downstream

```
# Aligned_sequences: 2
# 1: g20932_DS
# 2: inv/EN2
# Matrix: EDNAFULL
# Gap_penalty: 16
# Extend_penalty: 4
#
# Length: 54
# Identity:      35/54 (64.8%)
# Similarity:    35/54 (64.8%)
# Gaps:           0/54 ( 0.0%)
# Score: 99
#
#
#=======================================

g20932_DS      19383 AAGGATGGGATGCATTAGAGATCATACATTTGTAATAAAAGACTTTTTAA  19432
                     ||.||.|.||.||..|.|...|.||.|||..||||||||||....||||.
inv/EN2          316 AATGAGGTGAAGCTCTTGGTTTTATTCATCAGTAATAAAAGTACATTTAT    365

g20932_DS      19433 AAAA  19436
                     ||||
inv/EN2          366 AAAA    369
```

→ **below the threshold criteria**

5.b Gene g20933

Gene: **g20933**
Transcript: g20933.t1
Product: g20933.t1

Upstream

```
# Aligned_sequences: 2
# 1: g20933_US
# 2: inv/EN2
# Matrix: EDNAFULL
# Gap_penalty: 16
# Extend_penalty: 4
#
# Length: 58
# Identity:      37/58 (63.8%)
# Similarity:    37/58 (63.8%)
# Gaps:           2/58 ( 3.4%)
# Score: 89
#
#
#=======================================

g20933_US       4161 TAATCTTATTCCATGCACAGAAAAAAGCAAATCATTTTACAGAATGCATG   4210
                     ||..||.||.||..|.|||.|.|.|....||||.|.|||  ||||.||.|
inv/EN2          203 TATGCTAATCCCCAGGACAAATATATTTTAATCTTGTTA--GAATACAAG    250

g20933_US       4211 TTAAAGCT   4218
                     ||||.|||
inv/EN2          251 TTAATGCT    258
```

→ **Hit:**
```
>g20933_US_CRE-like
TAATCTTATTCCATGCACAGAAAAAAGCAAATCATTTTACAGAATGCATGTTAAAGCT
```

→ **BLASTN SEARCH** for g20933_US_CRE-like: e-value: 3e-25

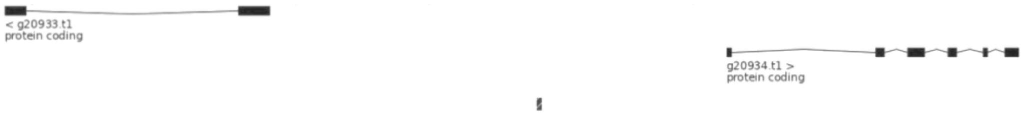

Downstream:

```
# Aligned_sequences: 2
# 1: g20933_DS
# 2: inv/EN2
# Matrix: EDNAFULL
# Gap_penalty: 16
# Extend_penalty: 4
#
# Length: 54
# Identity:      35/54 (64.8%)
# Similarity:    35/54 (64.8%)
# Gaps:           0/54 ( 0.0%)
# Score: 99
#
#
#=======================================

g20933_DS      19607 AAGGATGGGATGCATTAGAGATCATACATTTGTAATAAAAGACTTTTTAA  19656
                     ||.||.|.||.||..|.|...|.||.|||..||||||||||....||||.
inv/EN2          316 AATGAGGTGAAGCTCTTGGTTTTATTCATCAGTAATAAAAGTACATTTAT    365

g20933_DS      19657 AAAA  19660
                     ||||
inv/EN2          366 AAAA    369
```

→ **below the threshold criteria**

5.c Gene g29453

Gene: **g29453**
Transcript: g29453.t1
Product: g29453.t1

Upstream

```
# Aligned_sequences: 2
# 1: g29453_US
# 2: inv/EN2
# Matrix: EDNAFULL
# Gap_penalty: 16
# Extend_penalty: 4
#
# Length: 95
# Identity:      61/95 (64.2%)
# Similarity:    61/95 (64.2%)
# Gaps:          14/95 (14.7%)
# Score: 85
#
#
#=======================================

g29453_US       2855 TGAACATTGGGTTTTAAACAACGGAGACGCTTGCGTAATCATAGCTATCC   2904
                     ||||| ||||   |.|..||...|||||.|.|.. |.|||.||||||
inv/EN2          272 TGAAC-TTGG---TCAGGCAGGAGAGACCCATTT-TTATCTTAGCTA---    313

g29453_US       2905 AATTAATGACGTTGGAAGTTTTTAAGTTTTATGTATCAA-AATAA   2948
                       |.|||||.||  ||||.|.||. |||||||..||||. |||||
inv/EN2          314 --TGAATGAGGT--GAAGCTCTTG-GTTTTATTCATCAGTAATAA    353
```

→ **Hit:**

```
>g29453_US_CRE-like
TGAACATTGGGTTTTAAACAACGGAGACGCTTGCGTAATCATAGCTATCCAATTAATGACGTTGGAAGTTTTTAAGTTTTATGTATCAAAATAA
```

→ **BLASTN SEARCH** for g29453_US_CRE-like: e-value: 1.8e-46

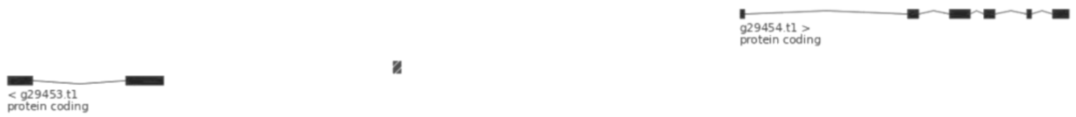

Downstream

```
# Aligned_sequences: 2
# 1: g29453_DS
# 2: inv/EN2
# Matrix: EDNAFULL
# Gap_penalty: 16
# Extend_penalty: 4
#
# Length: 54
# Identity:      36/54 (66.7%)
# Similarity:    36/54 (66.7%)
# Gaps:           1/54 ( 1.9%)
# Score: 96
#
#
#=======================================

g29453_DS       6262 AAGGATGGGATGCATTAGAGATCATACATTTGTAATAAAAG-ACTTTTTA   6310
                     ||.||.|.||.||..|.|...|.||.|||..|||||||||| ||.|||..
inv/EN2          316 AATGAGGTGAAGCTCTTGGTTTTATTCATCAGTAATAAAAGTACATTTAT    365

g29453_DS       6311 AAAA   6314
                     ||||
inv/EN2          366 AAAA    369
```

→ **below the threshold criteria**

6. Capitella teleta

Description: EN
Gene: **CapteG152545**
Transcript: CapteT152545
Product: CapteP152545

Upstream

```
# Aligned_sequences: 2
# 1: CapteG152545_US
# 2: inv/EN2
# Matrix: EDNAFULL
# Gap_penalty: 16
# Extend_penalty: 4
#
# Length: 48
# Identity:      32/48 (66.7%)
# Similarity:    32/48 (66.7%)
# Gaps:           1/48 ( 2.1%)
# Score: 84
#
#
#=======================================

CapteG152545_   7988 ATGTGCTCTTACGCCGCTAACAAGCCACTGCGCAGAATCCAAAATGCT   8035
                     |.|||||||..|.||.||||.|||.||..| |.|||.|..|..|||||
inv/EN2          162 AAGTGCTCTGCCACCACTAAAAAGGCAAAG-GGAGACTTAAGTATGCT    208
```

→ below the threshold criteria

Downstream

```
# Aligned_sequences: 2
# 1: CapteG152545_DS
# 2: inv/EN2
# Matrix: EDNAFULL
# Gap_penalty: 16
# Extend_penalty: 4
#
# Length: 66
# Identity:      41/66 (62.1%)
# Similarity:    41/66 (62.1%)
# Gaps:           3/66 ( 4.5%)
# Score: 81
#
#
#=======================================

CapteG152545_   7533 TTTTTATGTTATCTACTACC-AGGTATA--TCATGTTTATACTCGCCTCT   7579
                     |||||||.|||.|||..|.. ||||..|  ||.||.||.||.||..|..|
inv/EN2          299 TTTTTATCTTAGCTATGAATGAGGTGAAGCTCTTGGTTTTATTCATCAGT    348

CapteG152545_   7580 GTTCAAACTATAATTA   7595
                     ..|.|||.||.|.|||
inv/EN2          349 AATAAAAGTACATTTA    364
```

→ Hit:

>CapteG152545_DS_CRE-like
TTTTTATGTTATCTACTACCAGGTATATCATGTTTATACTCGCCTCTGTTCAAACTATAATTA

→ BLASTN SEARCH for CapteG152545_DS: e-value 2.8e-28

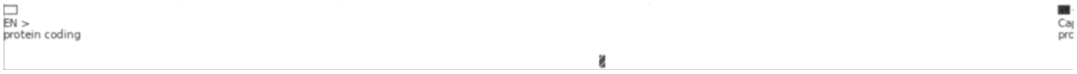

7. *Octopus bimaculoides*

7.a Gene Ocbimv22020235m.g

Gene: **Ocbimv22020235m.g**
Transcript: Ocbimv22020235m
Product: Ocbimv22020235m.p

Note: gene has no annotated neighbours on Ensembl.

Upstream

```
# Aligned_sequences: 2
# 1: Ocbimv22020235m.g_US
# 2: inv/EN2
# Matrix: EDNAFULL
# Gap_penalty: 16
# Extend_penalty: 4
#
# Length: 49
# Identity:      32/49 (65.3%)
# Similarity:    32/49 (65.3%)
# Gaps:           0/49 ( 0.0%)
# Score: 92
#
#
#=======================================

Ocbimv2202023   8607 AATATTCCAACCACCAATTCAGATATATGTAAATCGTGTGTGCGTATAA   8655
                     |.|||.|.||.|.|||...||.||||||.|.||||.|||..|..||.||
inv/EN2          201 AGTATGCTAATCCCCAGGACAAATATATTTAATCTTGTTAGAATACAA    249
```

→ **below the threshold criteria**

Downstream

```
# Aligned_sequences: 2
# 1: Ocbimv22020235m.g_DS
# 2: inv/EN2
# Matrix: EDNAFULL
# Gap_penalty: 16
# Extend_penalty: 4
#
# Length: 58
# Identity:      39/58 (67.2%)
# Similarity:    39/58 (67.2%)
# Gaps:           2/58 ( 3.4%)
# Score: 95
#
#
#=======================================

Ocbimv2202023  49004 TATACTGTAGTCTTGTATGGAACATACGTACATGATGTTGTCGCTCTTGC  49053
                     ||||.|.||.|||||| |.|||.|.|.||..|||.||..|| ||..|.||
inv/EN2          224 TATATTTTAATCTTGT-TAGAATACAAGTTAATGCTGCAGT-GCAGTGGC    271

Ocbimv2202023  49054 TATACTTG  49061
                     |..|||||
inv/EN2          272 TGAACTTG    279
```

→ **Hit:**
>Ocbimv22020235m.g_DS_CRE-like
TATACTGTAGTCTTGTATGGAACATACGTACATGATGTTGTCGCTCTTGCTATACTTG

→ **BLASTN SEARCH** for Ocbimv22020235m.g_DS_CRE-like: e-value 1.6e-24

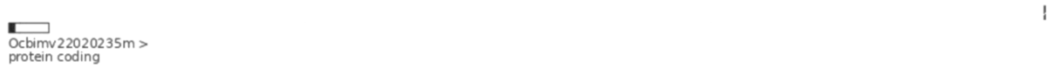

7.b Gene Ocbimv22005596m.g

Gene: **Ocbimv22005596m.g**
Transcript: Ocbimv22005596m
Product: Ocbimv22005596m.p

Upstream

```
# Aligned_sequences: 2
# 1: Ocbimv22005596m.g_US
# 2: inv/EN2
# Matrix: EDNAFULL
# Gap_penalty: 16
# Extend_penalty: 4
#
# Length: 70
# Identity:      45/70 (64.3%)
# Similarity:    45/70 (64.3%)
# Gaps:           4/70 ( 5.7%)
# Score: 89
#
#
#=======================================
Ocbimv2200559  38949 GGTGCTGGTGATGATGATGAGGAGTTA--ATGATAATGATGAT-GATGTC  38995
                     ||||||||.|.|. ||..|.|||..||  ||.|.|.||.|||. |||||.
inv/EN2           22 GGTGCTGGAGCTT-TGTGGCGGAAATATAATTAAACTGGTGAGAGATGTA     70

Ocbimv2200559  38996 GACGATGATGACGATGATGA  39015
                     .|.|||||.||....|||||
inv/EN2           71 AAAGATGAAGAATGGGATGA     90
```

→ **Hit:**
>Ocbimv22005596m.g_US_CRE-like
GGTGCTGGTGATGATGATGAGGAGTTAATGATAATGATGATGATGTCGACGATGATGACGATGATGA

→ **BLASTN SEARCH** for Ocbimv22005596m.g_US_CRE-like: 0 results -> unable to locate the hit on Ensembl!

Downstream

```
# Aligned_sequences: 2
# 1: Ocbimv22005596m.g_DS
# 2: inv/EN2
# Matrix: EDNAFULL
# Gap_penalty: 16
# Extend_penalty: 4
#
# Length: 60
# Identity:      39/60 (65.0%)
# Similarity:    39/60 (65.0%)
# Gaps:           7/60 (11.7%)
# Score: 87
#
#
#=======================================
Ocbimv2200559   3877 ACATATATATGTACATATATAAATGTACATATTTAAGTACATGCTGCAGG   3926
                     |||.||||||.|..||.|     |||....||..||||..|||||||||.
inv/EN2          219 ACAAATATATTTTAATCT-----TGTTAGAATACAAGTTAATGCTGCAGT    263

Ocbimv2200559   3927 --AGTGGCTG   3934
                       ||||||||
inv/EN2          264 GCAGTGGCTG    273
```

→ **Hit:**
>Ocbimv22005596m.g_DS_CRE-like
ACATATATATGTACATATATAAATGTACATATTTAAGTACATGCTGCAGGAGTGGCTG

→ **BLASTN SEARCH** for Ocbimv22005596m.g_DS_CRE-like: e-value 1.6e-24

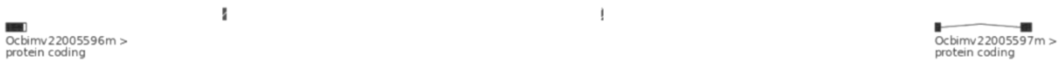

8. Schistosoma mansoni

Gene: **Smp_343800**
Transcript: Smp_343800.1
Product: Smp_343800.1

Upstream

```
# Aligned_sequences: 2
# 1: Smp_343800_US
# 2: inv_EN2
# Matrix: EDNAFULL
# Gap_penalty: 16
# Extend_penalty: 4
#
# Length: 38
# Identity:      26/38 (68.4%)
# Similarity:    26/38 (68.4%)
# Gaps:           0/38 ( 0.0%)
# Score: 82
#
#
#=======================================

Smp_343800_US   2642 GCAGCTAAGACCCATCTTGATGTTAACGGTGATCTAGG   2679
                     ||||...||||||||.||.||.|||.|..|||...|||
inv_EN2          285 GCAGGAGAGACCCATTTTTATCTTAGCTATGAATGAGG    322
```

→ below the threshold criteria

Downstream

```
# Aligned_sequences: 2
# 1: Smp_343800_DS
# 2: inv_EN2
# Matrix: EDNAFULL
# Gap_penalty: 16
# Extend_penalty: 4
#
# Length: 72
# Identity:      47/72 (65.3%)
# Similarity:    47/72 (65.3%)
# Gaps:           2/72 ( 2.8%)
# Score: 111
#
#
#=======================================

Smp_343800_DS  77710 TAGTTTAGCGGT-AGTTAGCTGCATCTCTTGTTTTTTTAAACTTATTGAT  77758
                     ||..|||||..| |.|.||.||.|.||||||.||||.|..| |.|.|.||
inv_EN2          303 TATCTTAGCTATGAATGAGGTGAAGCTCTTGGTTTTATTCA-TCAGTAAT    351

Smp_343800_DS  77759 ATAAATATATTTGTATACCGCA  77780
                     |.||.||.||||.||.|.||.|
inv_EN2          352 AAAAGTACATTTATAAAACGAA    373
```

→ Hit:

>Smp_343800_DS_CRE-like
TAGTTTAGCGGTAGTTAGCTGCATCTCTTGTTTTTTTAAACTTATTGATATAAATATATTTGTATACCGCA

→ BLASTN SEARCH for Smp_343800_DS_CRE-like: e-value: 6.7e-33

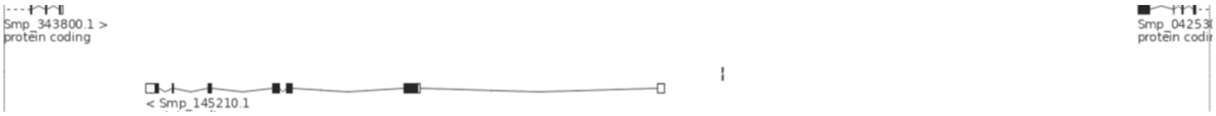

9. Adineta vaga

9. a GSADVG00002773001

Gene: **GSADVG00002773001**
Transcript: GSADVT00002773001
Product: GSADVT00002773001

Upstream

```
Aligned_sequences: 2
# 1: GSADVG00002773001_US
# 2: inv/EN2
# Matrix: EDNAFULL
# Gap_penalty: 16
# Extend_penalty: 4
#
# Length: 39
# Identity:      28/39 (71.8%)
# Similarity:    28/39 (71.8%)
# Gaps:           1/39 ( 2.6%)
# Score: 84
#
#
#=======================================

GSADVG0000277   2657 ATGATGATGACGAAGAGATAT-TATTTTAATCGAGTTAG   2694
                     |||.|.||..|.|.||.|.|| ||||||||||..|||||
inv/EN2          204 ATGCTAATCCCCAGGACAAATATATTTTAATCTTGTTAG    242
```

→ below the threshold criteria

Downstream

```
Aligned_sequences: 2
# 1: GSADVG00002773001_DS
# 2: inv/EN2
# Matrix: EDNAFULL
# Gap_penalty: 16
# Extend_penalty: 4
#
# Length: 46
# Identity:      31/46 (67.4%)
# Similarity:    31/46 (67.4%)
# Gaps:           3/46 ( 6.5%)
# Score: 71
#
#
#=======================================

GSADVG00002773   548 TAAAAAATGAGCAGGTGGAAAAATGGGAGTGACCAATCATAATGAT     593
                     |.|.|.|||...|.|..|||.||||||| ||||  ||||.|.||||
inv/EN2           60 TGAGAGATGTAAAAGATGAAGAATGGGA-TGAC--ATCAGACTGAT     102
```

→ below the threshold criteria

9.b Gene GSADVG00021246001

Gene: **GSADVG00021246001**
Transcript: GSADVT00021246001
Product: GSADVT00021246001

Upstream

```
# Aligned_sequences: 2
# 1: GSADVG00021246001_US
# 2: inv/EN2
# Matrix: EDNAFULL
# Gap_penalty: 16
# Extend_penalty: 4
#
# Length: 39
# Identity:      28/39 (71.8%)
# Similarity:    28/39 (71.8%)
# Gaps:           1/39 ( 2.6%)
# Score: 84
#
#
#=======================================

GSADVG0002124   3138 ATGATGATGACGAAGAGATAT-TATTTTAATCGAGTTAG   3175
                     |||.|.||..|.|.||.|.|| ||||||||||..|||||
inv/EN2          204 ATGCTAATCCCCAGGACAAATATATTTTAATCTTGTTAG    242
```

→ below the threshold criteria

Downstream

```
Aligned_sequences: 2
# 1: GSADVG00021246001_DS
# 2: inv/EN2
# Matrix: EDNAFULL
# Gap_penalty: 16
# Extend_penalty: 4
#
# Length: 140
# Identity:      77/140 (55.0%)
# Similarity:    77/140 (55.0%)
# Gaps:          10/140 ( 7.1%)
# Score: 97
#
#
#=======================================

GSADVG0002124   4827 TAAGAATACCATTAAGTAATGTACTCATTTGTGTGCTTTACCTGGTCTAG   4876
                     |.|||||||.|.|.|.|..||.|.|....||   |||..||.||||| ||
inv/EN2          239 TTAGAATACAAGTTAATGCTGCAGTGCAGTG---GCTGAACTTGGTC-AG    284

GSADVG0002124   4877 TAATTATAATTTTCTTTTTGTTTTAGTTTGGAAATAGTAAAAGATCTTAT   4926
                     ..|..|.|......|||||.|.||||.|..|||..||...|||.||||.
inv/EN2          285 GCAGGAGAGACCCATTTTTATCTTAGCTATGAATGAGGTGAAGCTCTTG-    333

GSADVG0002124   4927 TTATCGTGTTCTTCATCATTTTTTCAAATTTCTCTAACAA   4966
                          ||.||.|||||||.|..|..||.|...|.||..||
inv/EN2          334 -----GTTTTATTCATCAGTAATAAAAGTACATTTATAAA    368
```

→ below the threshold criteria

9.c Gene GSADVG00005228001

Gene: **GSADVG00005228001**
Transcript: GSADVT00005228001
Product: GSADVT00005228001

Upstream

```
# Aligned_sequences: 2
# 1: GSADVG00005228001_US
# 2: inv/EN2
# Matrix: EDNAFULL
# Gap_penalty: 16
# Extend_penalty: 4
#
# Length: 66
# Identity:      43/66 (65.2%)
# Similarity:    43/66 (65.2%)
# Gaps:           5/66 ( 7.6%)
# Score: 87
#
#
#=======================================

GSADVG0000522   2052 TTAGATGTGCGTGTTCATGAAGG-CGTAGTTTTACTTGTCATCAATAGTA   2100
                     ||||.|.||..||... |||||. |.|.||||||   .||||||.||.||
inv/EN2          307 TTAGCTATGAATGAGG-TGAAGCTCTTGGTTTTA---TTCATCAGTAATA    352

GSADVG0000522   2101 TATGTAGATATATATA   2116
                     .|.|||.||.||||.|
inv/EN2          353 AAAGTACATTTATAAA    368
```

→ **Hit:**

```
>GSADVG00005228001_US_CRE-like
TTAGATGTGCGTGTTCATGAAGGCGTAGTTTTACTTGTCATCAATAGTATATGTAGATATATATA
```

→ **BLASTN SEARCH** for GSADVG00005228001_US_CRE-like: e-value 1.2e-29

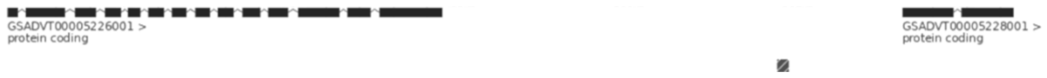

<u>Downstream</u>

```
Aligned_sequences: 2
# 1: GSADVG00005228001_DS
# 2: inv/EN2
# Matrix: EDNAFULL
# Gap_penalty: 16
# Extend_penalty: 4
#
# Length: 68
# Identity:      42/68 (61.8%)
# Similarity:    42/68 (61.8%)
# Gaps:           4/68 ( 5.9%)
# Score: 82
#
#
#=======================================

GSADVG0000522   1109 TCTTTTTTGATGTTGTTGTTGTGTACGAGATATAGCTGTAGTTCGAAG--   1156
                     |.|.||||.||.|||||.  |..|||.||.||..||||.|||.|...|
inv/EN2          224 TATATTTTAATCTTGTTA--GAATACAAGTTAATGCTGCAGTGCAGTGGC    271

GSADVG0000522   1157 TGAAGTCGATGGAGCAGG   1174
                     ||||.|.|.|...|||||
inv/EN2          272 TGAACTTGGTCAGGCAGG    289
```

→ **Hit:**

>GSADVG00005228001_DS_CRE-like
TCTTTTTTGATGTTGTTGTTGTGTACGAGATATAGCTGTAGTTCGAAGTGAAGTCGATGGAGCAGG

→ **BLASTN SEARCH** for GSADVG00005228001_DS_CRE-like: e-value 3.1e-30

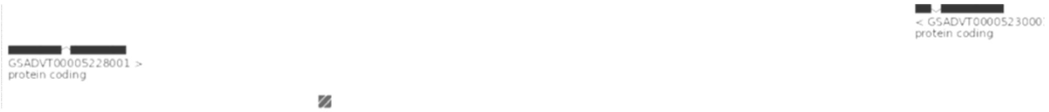

9.d Gene GSADVT00036502001

Gene: **GSADVG00036502001**
Transcript: GSADVT00036502001
Product: GSADVT00036502001

Upstream

```
Aligned_sequences: 2
# 1: GSADVG00036502001_US
# 2: inv/EN2
# Matrix: EDNAFULL
# Gap_penalty: 16
# Extend_penalty: 4
#
# Length: 63
# Identity:      41/63 (65.1%)
# Similarity:    41/63 (65.1%)
# Gaps:           5/63 ( 7.9%)
# Score: 81
#
#
#=======================================

GSADVG0003650   2003 TTAGATGTGCGTGTTCATGAAGG-CGTAGTTTTACTTGTCATCAATAGTA   2051
                     ||||.|.||..||... |||||. |.|.||||||.|   |||||.||.||
inv/EN2          307 TTAGCTATGAATGAGG-TGAAGCTCTTGGTTTTATT---CATCAGTAATA    352

GSADVG0003650   2052 TATGTATATATAT   2064
                     .|.|||.||.|||
inv/EN2          353 AAAGTACATTTAT    365
```

→ **Hit:**

```
>GSADVG00036502001_US_CRE-like
TTAGATGTGCGTGTTCATGAAGGCGTAGTTTTACTTGTCATCAATAGTATATGTATATATAT
```

→ **BLASTN SEARCH** for GSADVG00036502001_US_CRE-like: e-value: 7e-28

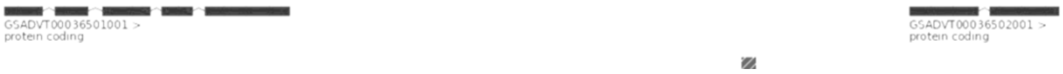

<u>Downstream</u>

```
# Aligned_sequences: 2
# 1: GSADVG00036502001_DS
# 2: inv/EN2
# Matrix: EDNAFULL
# Gap_penalty: 16
# Extend_penalty: 4
#
# Length: 70
# Identity:      42/70 (60.0%)
# Similarity:    42/70 (60.0%)
# Gaps:           4/70 ( 5.7%)
# Score: 74
#
#
#=======================================

GSADVG0003650   1080 TCTTTTTTGATGTTGTTGTTGTGTACGAGATATAACTGTAGTTCGAAG--   1127
                     |.|.||||.||.|||||  .|..|||.||.||...|||.|||.|...|
inv/EN2          224 TATATTTTAATCTTGTT--AGAATACAAGTTAATGCTGCAGTGCAGTGGC    271

GSADVG0003650   1128 TGAAGTCGATGGAGCAGGTG   1147
                     ||||.|.|.|...|||||.|
inv/EN2          272 TGAACTTGGTCAGGCAGGAG    291
```

→ **Hit:**

>GSADVG00036502001_DS_CRE-like
TCTTTTTTGATGTTGTTGTTGTGTACGAGATATAACTGTAGTTCGAAGTGAAGTCGATGGAGCAGGTG

→ **BLASTN SEARCH** for GSADVG00036502001_DS_CRE-like: e-value 2.1e-31

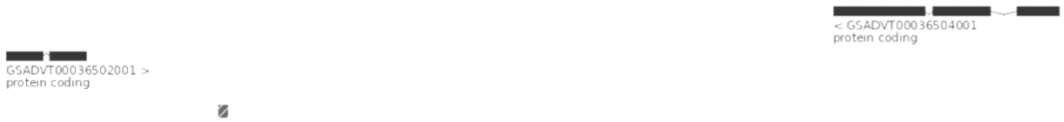

10. Hypsibius exemplaris

Gene: BV898_02358
Transcript: mrna.BV898_02358.1
Product: OQV23613.1

Upstream

```
# Aligned_sequences: 2
# 1: inv_EN2
# 2: OQV23613.1_US
# Matrix: EDNAFULL
# Gap_penalty: 16
# Extend_penalty: 4
#
# Length: 57
# Identity:      37/57 (64.9%)
# Similarity:    37/57 (64.9%)
# Gaps:           2/57 ( 3.5%)
# Score: 81
#
#
#=======================================

inv_EN2           45 ATATAATTAAACTGGTGAGAGATGTAAAAGATGAAGAATGG-GATGACAT     93
                     |.||.||.|||.||.|||.|.|||....|||| |..||||. |||.||||
OQV23613.1_US   1107 AAATTATGAAAATGCTGAAACATGATCCAGAT-AGTAATGAAGATAACAT   1155

inv_EN2           94 CAGACTG    100
                     .|.|.||
OQV23613.1_US   1156 AAAAATG   1162
```

→ Hit:

```
>OQV23613.1_US_CRE-like
AAATTATGAAAATGCTGAAACATGATCCAGATAGTAATGAAGATAACATAAAAATG
```

→ BLASTN SEARCH for OQV23613.1_US_CRE-like: e-value 1.1e-24

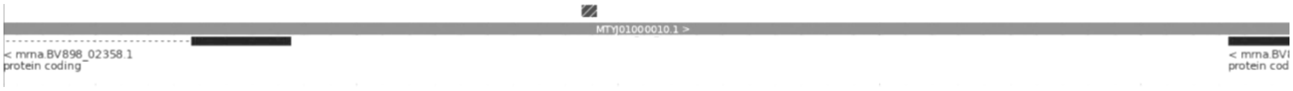

Downstream

```
# Aligned_sequences: 2
# 1: OQV23613.1_DS
# 2: inv_EN2
# Matrix: EDNAFULL
# Gap_penalty: 16
# Extend_penalty: 4
#
# Length: 41
# Identity:      25/41 (61.0%)
# Similarity:    25/41 (61.0%)
# Gaps:           0/41 ( 0.0%)
# Score: 61
#
#
#=======================================

OQV23613.1_DS      3 GCCTCACTTTATTAGCTGAACTTCGTGCCATAGACCAGACC     43
                     ||..||.|..|.|.|||||||||.||.....||...|||||
inv_EN2          256 GCTGCAGTGCAGTGGCTGAACTTGGTCAGGCAGGAGAGACC    296
```

→ below the threshold criteria

11. Caenorhabditis elegans

Description: ceh-16
Gene: **WBGene00000439**
Transcript: C13G5.1.1
Product: C13G5.1.1

Upstream

```
# Aligned_sequences: 2
# 1: C13G5.1.1_US
# 2: inv/EN2
# Matrix: EDNAFULL
# Gap_penalty: 16
# Extend_penalty: 4
#
# Length: 64
# Identity:      43/64 (67.2%)
# Similarity:    43/64 (67.2%)
# Gaps:           3/64 ( 4.7%)
# Score: 107
#
#
#=======================================

C13G5.1.1_US    3771 AAAACAAATACCAACTGATATGAGCTGTCAAAGTTGAGCAATGGGGTGCA   3820
                     ||.|.||.||  |||||.|..|||.|||.||||.|||..||||||.||..
inv/EN2           44 AATATAATTA--AACTGGTGAGAGATGTAAAAGATGAAGAATGGGATGAC     91

C13G5.1.1_US    3821 ATTGGTACTTTTTT   3834
                     ||..| |||..|||
inv/EN2           92 ATCAG-ACTGATTT    104
```

→ **Hit:**

```
>C13G5.1.1_US_CRE-like
AAAACAAATACCAACTGATATGAGCTGTCAAAGTTGAGCAATGGGGTGCAATTGGTACTTTTTT
```

→ **BLASTN SEARCH** for C13G5.1.1_US_CRE-like: e-value: 2.2e-29

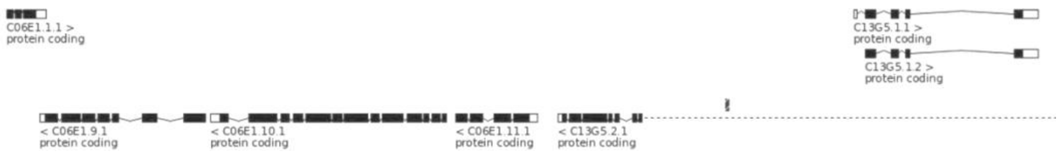

<u>Downstream</u>

```
# Aligned_sequences: 2
# 1: C13G5.1.1_DS
# 2: inv/EN2
# Matrix: EDNAFULL
# Gap_penalty: 16
# Extend_penalty: 4
#
# Length: 59
# Identity:      36/59 (61.0%)
# Similarity:    36/59 (61.0%)
# Gaps:           2/59 ( 3.4%)
# Score: 76
#
#
#=======================================

C13G5.1.1_DS    1684 AAACGGCGAGCCAAGTAGTTGGATGTGCTCTGAAAGAATAGATTTAAAGC   1733
                     |||.||.||...|||||  ||....|.|.|.|.|..||||.||||.||.|
inv/EN2          188 AAAGGGAGACTTAAGTA--TGCTAATCCCCAGGACAAATATATTTTAATC    235

C13G5.1.1_DS    1734 TTTTCCGAA   1742
                     ||.|..|||
inv/EN2          236 TTGTTAGAA    244
```

→ **Hit:**

```
>C13G5.1.1_DS_CRE-like
AAACGGCGAGCCAAGTAGTTGGATGTGCTCTGAAAGAATAGATTTAAAGCTTTTCCGAA
```

→ **BLASTN SEARCH** for C13G5.1.1_DS_CRE-like: e-value 1.9e-26

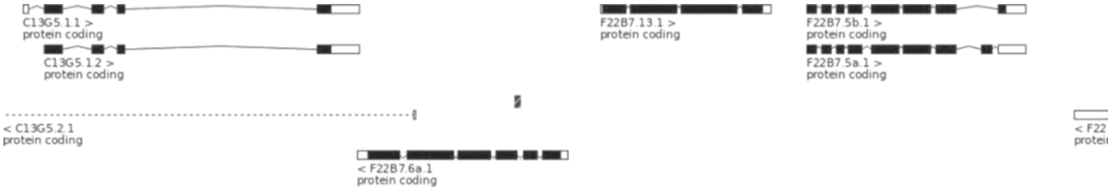

12. Priapulus caudatus

Description: engrailed-1a-like
NCBI Gene: 106809848 (LOC106809848)
Transcript: XM_014813077.1
Product: XP_014668563.1

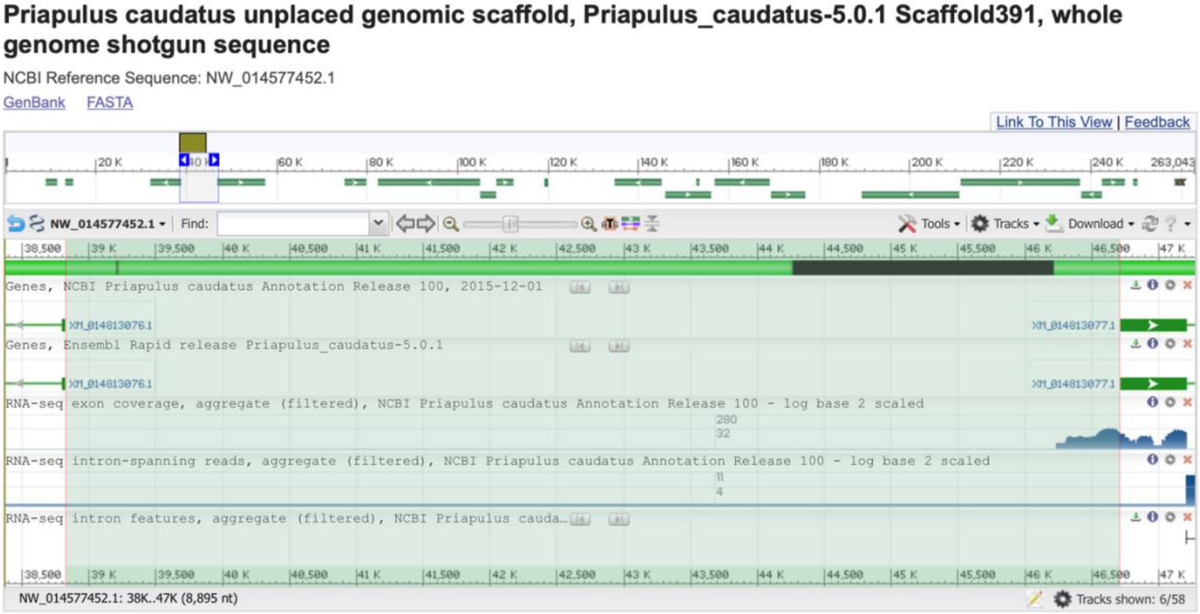


```
# Aligned_sequences: 2
# 1: 106809848(LOC106809848)_US
# 2: inv/EN2
# Matrix: EDNAFULL
# Gap_penalty: 16
# Extend_penalty: 4
#
# Length: 89
# Identity:      53/89 (59.6%)
# Similarity:    53/89 (59.6%)
# Gaps:           7/89 ( 7.9%)
# Score: 73
#
#
#=======================================

106809848(LOC     674 GAGACTTAAC-ACACAAAACACGCGAACAA-TATATAGTATTTATATGAG     721
                      |||||||||. |..|.||.|.|..|.|||| |||||..||.|..|.|.||
inv/EN2           193 GAGACTTAAGTATGCTAATCCCCAGGACAAATATATTTTAATCTTGTTAG     242

106809848(LOC     722 ATCATA---TAA--CTGCAATTATGAGTCTCACATTGCT     755
                      |..|.|   |||  |||||.|...|.|.||.|..|||.|
inv/EN2           243 AATACAAGTTAATGCTGCAGTGCAGTGGCTGAACTTGGT     281
```

→ **below the threshold criteria**

Priapulus caudatus unplaced genomic scaffold, Priapulus_caudatus-5.0.1 Scaffold391, whole genome shotgun sequence

NCBI Reference Sequence: NW_014577452.1

GenBank FASTA

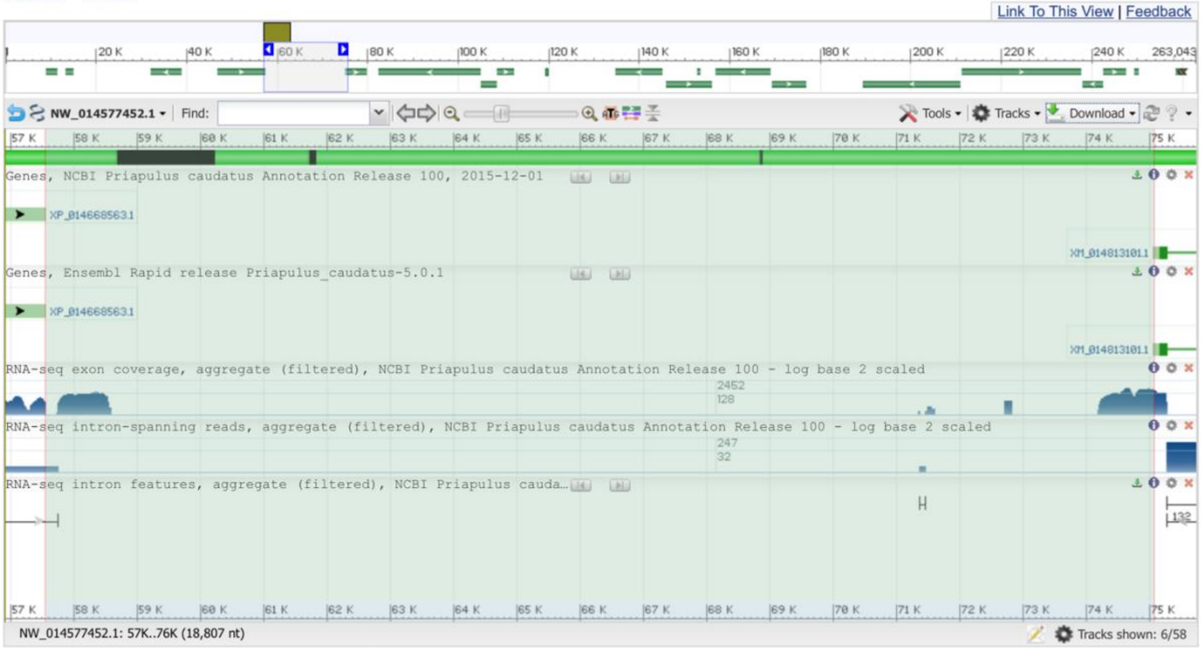


```
# Aligned_sequences: 2
# 1: 106809848(LOC106809848)_DS
# 2: inv/EN2
# Matrix: EDNAFULL
# Gap_penalty: 16
# Extend_penalty: 4
#
# Length: 153
# Identity:      86/153 (56.2%)
# Similarity:    86/153 (56.2%)
# Gaps:          12/153 ( 7.8%)
# Score: 90
#
#
#=======================================

106809848(LOC   9958 CAGCAAGGAGTTTATATTTTGTGGTGAGTAAATGCATTTAATATGGTTAG  10007
                     ||.|.|||.|.|.....|||||||.|.  ||||..|.||||..||||.||
inv/EN2           16 CAACGAGGTGCTGGAGCTTTGTGGCGG--AAATATAATTAAACTGGTGAG     63

106809848(LOC  10008 GGGTATCTCAGC--AAGAGATATATGTAATCAAATTGCTTATGTCAGTAT  10055
                     .|.|.|...||.  ||||.....|||..||||.|   ||.||.|||...|
inv/EN2           64 AGATGTAAAAGATGAAGAATGGGATGACATCAGA---CTGATTTCACACT    110

106809848(LOC  10056 AGCCTGTTTGATATTAAG---CGAGGCGCCCTAGCCT-GCAAGAGCTGCA  10101
                     .|.|.||..||||....|   |.||.||..||.|||. ||||| ||.|..
inv/EN2          111 TGGCAGTCCGATAGCTGGGCCCAAGCCGGGCTTGCCACGCAAG-GCAGAT    159

106809848(LOC  10102 CAA  10104
                     |||
inv/EN2          160 CAA    162
```

→ **below the threshold criteria**

Supplementary Data Set S6

Random Sequences

Random sequences generated with similar length for each of the three conserved DTB/MHB-specific CRE (356 bases for *sv/PAX2*; 247 bases for *dac/DACH1*; 373 bases for *inv/EN2*), with a CG:AT ratio of 40:60 to mimic the average genomic distribution of nucleotides. Using EMBOSS MATCHER, each random sequence was aligned with the respective consensus CRE sequence identified in *Drosophila*, mouse, and human to determine whether they match the threshold criteria of minimum 60% sequence identity over at least 55 base pairs with minimum $1e^{-1}$confidence level. Those random sequences with EMBOSS matcher results above threshold are highlighted in bold (for EMBOSS matcher results see Supplementary File S1, sheets 1-3).

1. random sequences for sv/PAX2

```
>p1
TGTTTGTAACCCAATAATCATCTGTCAAGTGTGCTAAGGCCATAACAAAGTTAAGAAATTCGCTAATCTTAACAGAA
TCCTTCCAGCTTTTAGAATGACGATTTTGAGATCAACTTCTGGATGAACAAAGCGTGAAGATAGAAACTGCAATAGT
TAGTTTTTTCACGAGATCCATAGACTCTCTCTTATCACTAACTGTCGTCAAATCCCCCGTAATACATCAAGTATACG
GACTTCTGACGCGCAGAAGGCACTGTAATTACTACCAAACCGGAAACACCTCTGTACCTATGGAACATGTTATATCC
TTTCGTGTCACATCTGGCTCATAATATTCAGTCTGTAGTCTGATGAGT

>p2
ATTCGGTTAACATCCTGTCATGCATGATTCTGGATCGCGCTATTATATTAAAGCACCAGTAAGTCATGTAAGAGGGG
GATGTCTATGTTCTGCTAGCGGATGAAAAGAGCTATAATAGCCTTAATATTAATAGCCTTCAATAACACCTTCTATA
GCACTCTACGTAACAAACCTGCACCTCTAATCGGACTGCTGTCAAATTCAACACATACCCTTCGTAGAATCAAGGAG
ACCACAAGCCCAGAGAATGACAAGATTTATTACATCCGGCAGCTATCTCATTGTACTATAGACCAGATCAACCGCTG
GTGCCAGGTACACAACGGTCATAGGACGATTTTAGATCGGCTCTACTA

>p3
TGTTACGACGTGAACATCAGGAGATTAGCACATTACGAACCCTGAATGACTCAGGAAATTAGACTGTATTTTAGACT
TAACCTATACGTAGGGGCGGAGGACGGCTGCGATGTCGCCTGCGTGCGATGTATGTGTATTTAGTTTCGATAATTGT
GTAGTTCCAGTTACCGATAATTTTCAATGTTCCAGTATAATCTGTCATCCGGAGACCCCGCACAGGTGATTGAATGA
CCTTAGCGAAATTTAAGTTGTTAAGGACGTACAGGATGGTGCTGAATTGATTGTATACCCTAACTGCACTAACGCAG
GTAAGGGTACTACGGATTGGCATTTTTTATCCGCTGGATAGCAGATCG

>p4
ATGATTAGTTCCTACTTTGCCATTCCGCAAGTTGCAGCCTGGTCATGTCACTGCTTAAACATTAATTATCGGGATCG
AAACTGGACGTTTGCACATTGTTGTCATGTGTTTGGCGGCGAACTAAGGGGTATACGGTTCCCGGGTGCTATTGACC
AACCCCGAGAGTAGAAACCAAATGGGGGCCTGAGGATAACTAATTAGGCATATTAAGTGTATATAAAATGCTACCCG
CTGTAAGGCATCAGTATTGCTTTACCTTGCCAACAGAGAAAGGAACCATGCTAACTTGTGCGGTTTTGATGGTTTCG
AATAGGATTTGTTTATATAGTTGTATTTAATACACTGACATCGTGGAG

>p5
GATTACAGTTTAAATTACTTGTTGTCAAAATGGGAATGAGTCAATCCCGACATTAAGAAAAAAGCTAGAGCTCTTTC
ACATTCTTAAGAGTTCAACCGATACTATGCAAAAAACCATAAAGAAGCGTAGAATTCTGCGGTCCAATGTGCATTCA
GACAGTACTACCAGGCAAGATGTGACACATAGTCGAAGAGCTATCTCGTATTAGAATCAATTTCTTGGGAATTTTAT
```

AAATTTTGGGTTCTACACATATCCAACTAGTTATTTCAACTAACTTTTATAAACAAATATGCTTTTAATGTTCATAA
GCATTAAATGCGAGTAGGGCTTCGCTTTTGATAACGAATGCGATCAAT

>p6
TGCGGCACCCTTCCATGCCTGATGATCAATAAACCTCGTCAAGTAACTCTATGTGCCGTGATAACGTAAACACACAA
CAGGGAATTAGAGTCGAATAAGCACTTAGTTCTAACGCCAAAATGAGAGTGCACAAACCTAGACTGGGCTTCATGTT
TTAGCGCAGGGGTGGCATCTATTATCAATAGCTCTACGTGTACCAACATACTAATGTCGGATTAATTTGACGAGTCG
CTAAGGTAGGGGACGGGTTTTGAAGATAACCTGGGACTATGAAGGGATGAACAATGACCAACGGCAGGCAGCAGCGA
GTTATGTGATTATGCATTTTTAATCTTTCTTTCGGGAATCTCGCTCCT

>p7
GTGAAGACTCTTTGCACTCCAAGGGGGGAGTGACGAACAATTATACAAAGGGTTGTTCCGGTTATTAACGTATCATA
TTCACATAACCCAGTGACGTTGTGCTCAGGTCTATAAATTATAACACAGCTAGGAACCTCAGCTCGGTATTCTACAA
ATAACCCACCGAGGATACCGCAAACTTGCGCTGAACCAGCGTCCCCATAACGTCACAGACATTAAGCTCGCACAAGA
TCGATGCGCAATGCTTGGAAGGTGGGTGAAAGAAGGGAATTCTTTTTGCACCGGAATCATCTACGATGATCACCCCT
GTAACGTTAATCCGAGGCTGCTGCTTGCGTAGATACCCTCTTTTGAAT

>p8
TTCATCATTCTTGCTATATTTGGCTTCATGGTGCGTAAATAGATTGAGTTATAACCGTGTGGTAATAAGCCCCTTTT
AATACAGCCTTTGAGAGTACGCTATCATCGCAGAATAACCCCAAACTCATTCATTGATATCTGTCTAGATGTGACGC
TCGATTGAGCAAATGGCAGAAATAATAGAAGCAAAAGATGTAAATATACCGGTCTCCCTTAACATCGGCATCTCTTT
AGAGACAGAAATACAAGCTATTAGCTTGAGTTCCAACGTGGCATGTTTTAAGAGTACCTAAGACACTTCACAGTCCG
TCGTTTTGCACAGCATATCCGAGTTCGTGGAAGGTCCATATCTAATAG

>p9
GTTGTTAGATTATGAGTTGTATGGTCGATACCGAGACAACCACCATAAAACGTCTAAATGCGGCAACACGCAGTGTG
CACTCTTAAATAATAAAAGTATCTTTCTTTCTGCAGCCCGCATTCCTCGAGTGTGTGAAGTACATATAGTCACCCAA
TTCTTTAATGCCTATCTCCCTATGCTTCTGTTTTGTAGCCCACGTTGATAGAAATGAAGTCCGGATACTACCAGAGA
TAAATACTTCCGTGCGTACCCTGGACCCCACCGCTAAGTACTAACCACTCGTTCGGTGCTTTTACTCGTGAGTCAAG
AGTCTCACGATAGTTTCTATCGTTCGGTATAAGCTTACCCTTTTAATA

>p10
CCGTAACCCTAAAGACGGAAGCCGTGGGCCAGCTCATAGGCGTGAGCTAATGTGATAGCGCGCATCCTTATCTACTT
TCAGCCTCAACATCCGCGTAACAGTGTGCTCATTATGTACAAGAAAAATTCATAAAAAACAAAATAAAGATTAAAGA
ATTCGTATGTGATCCAACATGACAAGGCGGTACGAGGTTAAGTGAAATCAAGTTTGCTAGGTTATCGTCAATGAATG
AAATCGGTGATGTCTAGCTGCGTTTTTAGACACTTTCAGACCTGTCCCTGCTTAAACTAGTATAATTTGTTTTTTCA
CTGTGATAAAAGTCGGCATCCGCACAGCTATAGGGACGTGTATTTCTC

>p11
TGAGTATGAGTCTTTTATCGTCTTATGGTCACCAGAATGTTGCTCTGGTCTTTCCTGCCCCTCGGGCGTGAAGAGTT
GTATAATTAGGGGCGTTTAAAAGCCCGAAAGCGAACCCCTTAAGCATCTTTATGAGATTTGAGTTAGCTGCTTTTTC
AAGGCGAGAATCTAGGACTTGTCAACGATGTATGCTCAGCCACTGTCATCCGGGGGGAAACGCTAGTAGGGCAAGCA
ATTACTACAACGGTACTCGCTACGATTGAAGTTGTAGAATTTTAATTCATCGTTATATTGTTACTTATAAAAATGTC
CAGGTAGCATCTGCGGAAAATTGACGGGTGAAAAAGTTCAAACGGATC

>p12
CCTTGTCCTGTCGAGGTCGGATAATGGCCAAGCTTGAACCTAAGACAATTGAGGGTCCGATTCGACAGTATAAAAGA
AAATAATTCCCTGGCTGCCAGGCAAGTGCATTTGGAGAACCAACAAAATCACTAATATTTCGTAAGTCTGACTAGCA
GAATTAGTTCTTCATCATGTACGTATCAGCAACTAAGAGGATTGATTATATATCCTGATATCCAACTAATAAACCGG
AAGCAAATCTCTTGGTTCGTCTTCTAAATAATGAATGAGTCAAGAATAGTAAGTCAATTATCATGTCCTTAACCGGC
ACAAAAAAGTACCGACTCATAGCATCAGTACAATCATCAAGTGAATTT

>p13
ATCCGATTGACAAGAAGAGAGGTATGTAACTATAAGTAATTTTCTTCCAGTTCACACAATTTCAGTATACTAGATCA
ACATCTATCTTGAGCAAATGCATACAATTCGTAATCGGCCTAAAAATGACCTACACATAGTTGTTATGAATGAAAAC
TAGGCCAACGGTCAAACTGTAGATCATTAATATACATTATTATGCGCCAGTAGTAAGTGTATCACTGAAAACCAGAT

CATCTATTATGAGGATGGTTCATGGGAAAATTCTTGGAGAGTATCGGCTACTCCTCGCGGCTACAAATATGTACTTG
CTTTGTTCACATCTTTTCGTCGGTTGGACTATAATCTAATAATTGTAA

>p14
CGCTCTAAGTTAGCTGAAAAGTTAGTATGAAATTACATGCTCTTCTTCAATTGCGCTTACATAAATAGACACATCAT
TACATGGGGTGGTCAGCCTATATCAATCAGGAAATTATGGAATTTTTCCGGCATTTCCGCACATGTCGGTGTCTGGC
ATCAAAAAAGAGCGGCATAAATTCTCTGAGATACGAATCCAATAAAATACATACCATGTGCCTTGAGTCTACTTATC
ATCGTAGATCATTGTACTAGATACATCCTCCTTAGCAGTCAAATCAAACTGTAACAGGTGGGACACATGCATTATTT
AAAAACGATCTTATAAAGGATATCATCTATAGATGTAAGATAACCTAG

>p15
CTAGGATAGCATGAATCAACGTTTTGCCTCAGGTGTGCTACACTTGTAGAGCATCAATTACATGCTGGTATATGAGG
CGAGTTACGGCCCGGGGGATATTCCTTTAGAACAACCGCGTCATCCTATTTGAGCTGGCTCGCCAGGTGCATCAGCT
CCACGTCAAGGTTGGAATTACATTTGATGATATAGATCTGGCGTAGGACAAAGCGCGCCCTGGTAACATATTCTAGA
ACGTACGTAATATCTGTATTCAGAGATATGGCTCTATTTTTATAATTCGTTCTCTGTCTATGTTCTTACGAACTAAA
TTGGATCTTACCACAAATGAAGCCTGTAACTCGGCATTTTTATGTTTC

>p16
TTGGGACATCTAGTTGTCTAGGTCGTATTAACCTTTTTTTTCTAACTATGATCCATACTGAGCAGTATGGTATGGCT
ACCGGTGCATTGTATGATAGGCACAAGATTAGCCCTACGAAGTACGTGGCGCGTTTTAACGAACACTTTTCATTTAC
CTAAAATAATTGATGTTTGGTGTATGCCTTCTATGCAGTTATAAAAACACTGGTCAGACTCTCGCTGTTTCCGCTAG
CGTTAGTTGGATCATCGCAGTTACACATATTTAACCTGCGAGAAGTAGCATGAGTAGTTCCAACTAGATGGCTGGGA
GTATACTAAGCATATTAAAATAGCGCATAAACCAAATTGCCTAACGCG

>p17
TCCTTCTCAGTAGTCCCCAATCGAATTTCTCGATCTGTCTCGTGACTTCCTGTGTCATTCTTAACCTTACACAGTCT
ATGACTTGCCTCATAACATTCTACATTTTGTTTAACCACATTCACAGACTGTTACGAAACCAGTTATTCCAATAACG
CCTCTATTGTACGTGGTATTGCAGATATGAGTTATCTCCGACTTATAACTTCACGATATCGCGCTACGATCAACCCT
CAAATTTTTGCATTTTGTCGTGTGAAGTTTGAAGGCATTACACTGCTCTTACACCGGACGATATTTACGGGCAAATG
AATTCTTTCGCTCTTTTGCGCCGAATTACAATTCGGAGTTGATACAGA

>p18
CCACTCAAAACGTTTTGGTAAATGAGGAAAAAGTCCGAACCAATGGCTATGACAGACCAGAGACCAACTAACCCCGG
GTAAGTCGGATAACTGAAAAGTGACGTTATCCGTTAGGTTCTTCGTGCTTTTTAAGGAAATCGCACGCGGGCAAGTC
TGCGTTTGTTGAAACATGTCTATCTTATCATCATGGCTCGCTGAATTTGTTGTCCCTTTGCCCAAACACTGAATGGA
GACGACATGCATCTAACATCCTAACCTTTTACTTATTTGTAATAATTGGGCATAGGAACCTACCGACGAGAAACTTA
CCGTTGCAAGAGACAACTACCTTTCTATGTAGATTGTATCAACAGGAA

>p19
ATTCAACGTGTAATTGTTATGCACACGTTTATTCCCCCATGGGTTTAGGAACCTTCGCTATAGTGTCGAAACTCAAA
TTGAATATGAATTGCCTATTGTCAGAGCGCTAAATAACCGTTTAAGTTCTATTCGTTTCTGTGCAGGTACGTTCTCC
GAATGTTCTGGTATACTGAGTTAGCTTGAATCATTTTACGTAGGTGATTTAAGGGATTGAGATCTTTCTAGCCTGTC
AATTCGAACGGGACAATTATTGTTCTGACGGACAAAATTAGTGTTATTTCGAGCATCGCCCAATTACTATTACATTT
AATAGAGTTTACGTCATACCAACCTAGGAACCCGGAATAAAGCATAAT

>p20
GCACACGGTTTCAATTGATACTATGAACCTTGGTTTTGTATCGGAGGTTTCCGGAGACTAATTTCGGACGGTGATGC
TCTACGACCAACATGTAATAGTGCAAACGAACCACTGATCCTCAAACACTTAATCAAATCTTAATCATTTTTGGTGA
CATAGCAATTGTATTAGGCCGCGCATCAAGCAAACAGCGTATATACTGATGAAAATTAATCTATGATTCGTGTGCAG
TTTATTAGAGATCACAAACAACACTGATCTTGGGAATATACATGCGTGTCAAGCATACTTTATTAGAGTATCCACGA
CTAATGACACGCGTTGATTCATTGTCGCCGCCCCTCAGACTATCATAG

>p21
CTACGCATGCCCACTATTCGGATGTTGAAATTCCTCGGACGATACTAGAAGTTAATGGGACGTACTAACATCCACAG
GAGCGTACTCACTAATAGTTTCTGCGTACGTTAAACGAACGTCGTTAGTAATTACGGATAAAATGTGCGCAACTGCT
CACGTGGAACTCTTTAAGGCTGTTGCCCTATTATGCATGCATGATGTATATTGAGCGAGAGTTTCTCCATAAGAGTA

TGGATAAAAAACACTTTTATATACTAGGTTTGTGTTTCTTGCTTTATGAGACGCAGACTGGTGGACCCAAATCCATT
GGCTGCTTTGGCAAACTATTGATTCAAGGCTCGGTTATTTTACTGCTT

>p22
GCTAGTTATTGCGTCTACACTATTATCTCGTACCCCCACCCCTAGTATTTCCAATGACCACTCAAAACACACATTGT
TTACAAAGTCAACCGTTTGCGCAAATGGTTCTGTCTCGTACGAGTGTACTTTCCCAGTATATAAGCTTAGATGTTAG
AAGGTCGAAACCTCAGCAACATCACTCGATGCGATTAATTGGGTATTGATGATCTAGTCACCTTTTACATTAGCCTA
CTTTCGTAAGTCTGAGAGCGGCCATTAGGGTAAATGTAATCTTCAGGAATATAGAATACTTATTGTTTCGTTTCTCT
TATGCGAAATCTCCAAAAACACCATAAATGCAATATACCGTGTCCAGG

>p23
AGAAATTGACATTTGACTAGATCCGTTGTCCACGAGGTATATCTTTCAGCTCCCATAGTTATTTACCAAGAGAAAAT
AGGGAGTCGGCTAGATGGGTCACGCCTACGCTTGGTAGTCCATTCATTATTATCGTCTTCATACCCTCAGTTCAAGT
TTGAGCTTCGAGCTGCATCGGTTAGAAAGAGAATTTGCGTGCAAGCCCTTAGTTACTGTCGTCTATACGGTCAGAAC
TATCCCATGTGGTGAGCATTCTAATGGGAATGTGCGAATGCCTTTGATCTTTCGCACGCCTTCACACTCTTGATGGG
GACGAAGACTAACTATCAACTCTTCAAATAACCCGGCCCACACTGAGG

>p24
TGCAAGGTCTATGCGTAGGTCCCAGTCGTGAATACTTCCAACTTATTGATATTTCTCGTCCTTCTAAGATAGGCCTG
AGTAACTAATTAATGGCAATGGGCAATACTCTATAATGGTGTATGTCATTAAGATCGCTCGCACTGTTAGATAAACG
CTTAGAGGGGGATAACATGGCGGTACTCACACTCACTATCCTGTACCTGTAGTGTTCTCAATTAATCGAAGTCCGTA
TTGACTTGTTTAAAAGTGGCTGGGCCAAAATATTAGCCTATCCCACATAGGTCTCCCCTCATAGACAGGGTTAGATT
CGACGACAAATATGTCGCGATATGCTTCCATTAGAACTGATTGTGGAT

>p25
AAAAACATAGAACAGTTGTGTGTTGGGCTCTGGTTGCTAAAGGACTCATTTTACGCGTAAAGCATTCCTCCGTAGGC
AAACACGCCGATTCACACGCCGTTAGGATTAAGTTCCCCGTAGACGCGAAAATTAAAACAGAGAATTCCAACGACTT
CAACCGCCCTTTTTAACGGCCAGGAGAGTTATTATCAACTAATAAGACTCGGTGGCACTTGAGTAATATTACGGAAT
GGTCTCTAGTACCGGAGAGCAGATAGTTGTTACTCACCCAAAGCTCGCTACAGTATAAGCACCAACTGAGCAGCAAT
GGGATATCCTCGCTTAGCCGGATGATAGTTCAATGTTGGTATAATTGA

>p26
GTAATAATTGAACCCGGGCCACTTATGGGCAGGTGTTCTAATATGCCCTCAACCATATGTCGTTATCCCTCGGTCGT
ACTGACATTTATTATTAAAGTGAGTCAACCGATACCCAAGCTCGCAATACTCTTCTTATCGTCTTCAATCCTGGGCG
TCGCACAAACAACGTATTAAGTCCGTAACATTTTTAGCTCGTCACGGTGTCGTCTAGCTTATAATAATACAGTAATC
AAACAGCGCATGACCAGAGTACAGTATAGCTAGTAATAAGCGTTGCTGTCGCGAAGGTCCTTTACAACAGCGCTCGT
TGATGTATCACGTGATGTAGTGCGCGAAATTATACACTTGATGATCTC

>p27
TAAGAAATGCATGTGTATCATCTTTCATATCCAGTTTTTATGTGTGACTCTTTTGAGTCTAGCTGAGAGCCAACATA
CATAATAGTTAACATTTCAAGACGGTAGGGAAAGTAATTCAGGGTAAAGTAGCTACTGAGTTTATTTGAAGCTCATG
GGAACAAAGGCGTGTACACCTAGTTAAACACCCTATTCATTAGTATCCAGACTATCTGCTTCGCCTCAATATTTTTT
TTTGTGATCCCCAACACCACTATTTCAACTAGTATAACGATCAAGATATACAGAAGCTTACTTTGCTGTGCTAATGG
CCGAAGTGGATTTAGACTATGGATCAGAGGGGTAAAACTTGCTAAATT

>p28
CCGTCCAACATACGTAATCGACTTCTCTCGACAATGGGTTAGAGAGATAAGGTCATATCATTCGATAAGTATAGGGG
GATTGTCCTTAGTATATACTTAGTTAGTTTTGTTTCATGTGGTTCAGCACATATAAAGCTGCGGGATAGAAGTAAAC
ACCTAATGTCTCGCGGGACGGTCTGATTCAATATGACAATAATCTTGACATACGTAACAAGGTTTAAAAGCCATCCA
TCATTTAACATTACAAATGACAATAGTATATCGCTTAAATAAAGGTCTTGCAATGCAATGTTCCAGCATAAGTTTGA
TCTAAATGTTCAGTAGTAGCATATTTGTTGCACGTAGACATGAAGTGT

>p29
CGACCAAAACACACGTTATAAAAATAAATGTACACGGCGGGGCAGGCCGCCACAGCTTTTGTTTCGCCTAGTTACTT
CATGAACCTCGAGCAAGCACCGGGTTCCAAAAAATGATCGCTTAGTTGAATAAGGCTGACTTTTTAGTTCAAACTAG
TACTATAACTGTCGATCTAAAGACAGGAACCTGTGTAGGTTGAACTGAGAATTTTTATTGTCACTCAGGTATCAGGC

CATTTGTATAGAAACACCCTCAAAATGCGTACGTCAAGCTTTATTTAGTAAACGATGTCCCGAGCCAAAAAATGTTA TTCCTATCGTTGATCATTTACACATACCTGATAATGCCACGAATCAAT

>p30
TGGTCATATTCCAAAAGAGCAATATATTAAGAACGGTAGAATTCAACCACATATAGTTCGGCACCAGTGTTATGAAT
TTTTAGTTAGGACACAGTGAGTTTGAACGTTCAGAGACCGGTGTAATCCTAACCTTTCGTAATACGGCATTCATAGA
AACACACGCCAGCATTGCGTTTTGTAAAACGTCTTGAGGATTTATAGGTATTAGAGCACACCAGACACTTTTCCTCA
ACTCTACAAGCCACAGAGGCAAGGAGTCTTAGGGTATCTATAGACTTGCTTTGTGGGCTTTTCGGTAATGCGGAGGG
TCTTTTTGAGCGGGGATTATAAAAGTGAACGTAACTCATCGGTTATTA

>p31
AGTGTTTCCTCCCTGACGACCTTAGGTCATATATTAGTTCTTTAATACATTCTAAAGCTTCGAGAGAAACGCAATGT
GTAGCGGAAATTTAATCCGAACGGGCTGGTCAGGGCCACTCTAAGTCGTGTTTGGTGCGCGTACACTGTAGGCCGCA
AGCAAGTTTCCAGTTCGATTAGACTAACATTCACGACAGCGACTATACCCATGACGTAAACACATTGATGGGAAAGT
GAAAGAGGCGTGGCGTAGCCATTTCTGACATGTAACCCGGTGCGCGGCAAGACTTGGTTGCAAATCCCAACCCTGAC
TTCTTAAGGTAATCTTGATTCAAAAGCTTGTAGTGTTCACTGTGGCAT

>p32
AATGGTATGAGCAAGTTGTTTCTAGCCGGAACTAGTTGTCCTAAGAAAATAGTTAGCACTTAGGTAGTATTTTATAG
GCAAGCGAATCGGTTTGATACCGTACTTTAGGAACGTTGGCATTTCCAGTGGGCCATGGATTGCTACCAACATCCTA
TGCGATAAGGTGATAGACCAAGCAGTCAGCTTGGCAGGAGTGAATCACTTAGGCGCTCTAATGATACATGGGTTTTT
CACATATAATATACAATTGAATAGGTGCCTTGGCATGTAATCTATATGCTTCAACACTGACAAAAGTTGGTTCTGTG
GTATATCTTAATAATAACAGCAGACAGAACAAGTCTATCGAAACTTGT

>p33
ACCCATACCTTATATCTCGTTCAGATATCTGTATACCTGGAGCGGCACAACCTCTCTAAAGGAGACGTTTATCATTC
GTTGCTCTACAGACGCAACGGCTGTAAGTGGTACTCAAACGCTAAGAGTTAATGACAGCTCGGTTTGATGATGTCGG
TATAGTATCAGAATCTACAGAAAACAGTTAAGAGAATTGCAGGACTACCATACGGCCATGCTAGAGCGGAAATCTTT
TACAATTTGATCTAAAGGTTGATAAGATAGACCTAGTTATTAAGGATCTAGTTACTTAACCATGGTTCCACAGTGCT
CCACTGTGCGCGATAAATTATAGTTAGTTTCAAAGCGACTCGTAAACG

>p34
AACTCTGAAATATTAATAGATTTGCCCTTGAAATGTTTTCGCAAATGCGGTGTTTCTTTCTCACTAAAGCATTCATC
TATGTTTTGTACGGCTTACTATATAGTAATCGGCTGTCCGCATTTGTCTAGTGAACGTGGATATCACGTACTTTTAT
TCTCGTTCTGGCAAACCATTCTATTTCACAACGCTGATATGATGACAACGTCGCTGTGTTACATAAACTGTGAGACC
ACAGGGTATGCGTCAAAGTAAGGTTGAATCATCGTTAAGAATATCTGCCCGACATTTCTTTATGTGCCTTTTACACG
GGTTCTACGCAACGAATTCGGATACATAAATTTATGAGACGCCATTTA

>p35
GAAGTAGCTGGACGAGCATGGTTAGACCTAATAAAACGGTCTACTGTTTACTACGTATCTGCGCGAATCTAGACTTA
ATACAGTTGAGCGGTGGGCGTATGTGAACAATAGTCTAGAAAGCTCGATAAGTTATTACAGGCAAAGGCAAGGTGAT
GTGATAAGCTATTCTAGCTTTTTATACCTAAAATTACAATACCAGACATAAATAACATTCGAAGACAAATTACAGAA
CGCATAAGGTAATTTATATCGGTATCAATCTTTTTATGAAATACCAACTTGTGTCTGAAAGTGTAGATCGGCATAGA
CTTAAAATTAGATATTGGTGTTGTAATTCGGCTTACTCGAGATATTCA

>p36
GGTAGTACGTGCGTATTCACCAACAGTTGAATCCCTTTAATCTGTACGACATTTTTCACAAAACGCTAGCACTTAGA
ACCCAGGACGAAGCTGAATCCAACGTATTCGTTTCGGGGACGTATTTTTACCAAAAAAAAAATTTCTCATATTCGAT
TCCTTCTTACCTCGCATACTATTACTTACACTTATTTAAATAGATCGCACAAACCAACAGTTAAGATTAGTATTGGG
GTAAGAGGTGTTTGCGTTACCGATTTAGATCCAACTTACCACCTCTTCTTGAATGAAACGCTACATTACTTTTTCTC
AATTAACCTGAATGGAAATGAACAATCGTTAAATATGTAATGCATGTT

>p37
CGACGAAGATTTGCAACATTGGCGGCGCTATTGTTTCTTCATTCTAACCTTCTGCCCAGATCTTACTTCAATATTTA
AAAGACCAGCCCTAGTCCGTGAACATCTCCAAATAAAAACGGTTCGCAGATTCGTTTCTGGAGGAAGTCGTCCGTTA
ATCAATAGATGTTGGGTGTGCCCTGGCTAAAAGCATATAGGAGTTCGCCGATCTTAACACTGGAACTGTTATGTTAG

TGGTTACGCACCAGATATACAATTGTATTTGAAATTAAATTACCTAAAATTCGTAACCTAACGTTAGTAAGTTTATT
GTGTCGTTGGGAACTTTCTATATGTAGAGTCACTTTTAGTGCAAAGAA

>p38
TGTCTACGATTTTCTTCCAGACCACATTGGACGCTAGGTACCTGATGATTAAAGCATCGCAGCTCAAGCCGGACCAA
GTGCGGAAGTGACTTACGACACCTTTCTGTGGTAGTTATTTACGCAACCAAGAGATCGTGACCCTAACTAGTAAGGT
ACAAAGATAATTCTATTTGTTGTTTACCATATGTCTCGTGTCTTAGCCCTAGCGTTTTCTTTTTGAACGAGCGGGTT
CAGGATTAACTGTATCCCTTTGCGACTTCATTTTGGATAGGCTACCCAAATATATAAACGCGTACTTCACAATCGAT
TGGTCAAACAATAGATACGTCTTTATTTGTGATTGGTTAGTTACGCGC

>p39
CTTTAACCAACTTAACTTTAGACGCCTAGAGTATGAGTTTCTTAACCTGTCACTATATGGTAACGTTCACCTATCTA
CTCCCTCTTTGCCTCTACAACTCGGGTGGAAGTGTAGCATGTTAGTTCTGGGATGATAATAACCTGCAAAACAATAA
GAAGCTTTTTCGTTGTTATGTAAACCACAGTGATTTTAGATATCAAGCTATATTTCAGGAAATATAACAAAGGCATG
TCCGCATCAACCCGTAGGAAGTAGTCAGGCATTGTATACGCCCACATTGTGTCAAAAAAGCTACTTTGAATCTTCTG
TAGAAAATTCTGATGTAACCGTGTAACGACAAAAGAATCCACCGCGAG

>p40
CCAGCGCAATGTCCTATAAACATAATCTGGCCCGAGCAGTCACGAGACTCATCAGGACGTACAAAAACCTCCGAATA
GTACAACCAAATCGCAAAATTTAAAAGCGTGCTGTCGAAGCACTCATAGTCTTAAATTTATGTAGGGGACGAAAGCA
TCTTTTCTTGGTCCTTCACGTACGCAGTTCTATGTTTAAATGGACATATAATCGTTCAACTTCGCATATTACACGAA
AAATTTCAGATTTTTTACGCCAAGCACAAAATAATATGTGGGGCCAACCTTTTGATTGAGAAAAATCAAGGTTGGCA
CGTGTGAGAACTACAAACAAAGATGTAAACCCGTGCGGATGCCGAGCC

>p41
AGGTACATGCGATGTACTACATACGGCAGTAAGGTTTATAGACATATCCAATCTACAAATACCCTCCTAGTTAGTGC
AGCGTTCATAAATTACTTGTGTCGGGCTTTCAAATTCAGCGAGCTACCTAAACTGTCGCGTAATCTGTGTTCAGCAG
CACGTAACTGTCAAAGATTTTCCGTTCGCCTTCTCTCGCTACGTCTGGCCCCAGTACTCCTCGAGTAAGTACTGGTA
TGGCGTGGAGTCAATAGGGAATGGTAATACTAAATTTGATGTAACTGAGATCCAAAGAGCTACAGGTCCCATATTAT
TTGATTGATCAAATTTTAGTTGATGCAACTATCAGAAATAGGAAAGAG

>p42
CAATCGATCATATCGTGGTAGTATTATAAATTTTAGGTTTTAATACATGTGGAGGACCATGGACTTCCCAAATTGCA
TTAGCGCTTGGGACAAACGTGTCCCTTTTTTTCTTTGAAGAATACGAATTTCCCTTTTCGAAGCGAAGTGGCAGTTTA
TATTTTACTCTTCCTTCATTCCGGGGAATAGACCCAAGACTAAGACGAGTGGACTTCCGAATCTTATGCTCCTTTCG
TGAAAGGCGCTTACGGGTACCAGTATACGTTCTCCAGTTTTTTAGATATGTCATCGACATGTTGGTCAATTCAGTTT
ATACCCGCTGTGGTGGATGTTCGTAATTTCATGACCCATTATACTATA

>p43
TACGCGGGAGTCATCATATTCGAGTGTAAGTCTAATGGAAGAGGGTATCAATGCGGCGTAGCTTCTTGTTATATCTA
CTAGGTGCATGGTTTTCCTAGTCCCGGTCACCCTGTCGGACTTGCTGAGAGTAAGCATGTTGAAGGTGTTATACTAC
TGATCTGTATATTGTGTTGGATAATGTATTAGAACTTCCAGTAGGCGCCTATCCGATACCAACTAGAGAAATTCAAG
CAGACGTGAAGTACCCTTTAGTGTTGTCCTTAGTCAATGTTACATATCTTTGTGCAATCTAAATTTCGCACATGATC
CAGGTCAAGCGTAATGGTTTATGTCCGAATATAATTAACCTTAGCAAA

>p44
TGAACCTGCCACAATCGGGGATGCCTGCGTTGGTCTATCTTGCGTCTTTTTTAAGAATTGGTTGCAAAGTGGCTACT
ATAGTTCTCGAGCGTAATTACAGTAAGTTCAAATTATTACGTACTCTCATGCGATGCGGAAAAATGCTGAGTCATTT
CCTATTCTCTGAGAATGGGGGCTTATATATTTTCAAACATGGTATCCAACACTATTTAGTAACGGTTTCATGCAGTT
TTATTCCTCGATAACGTGATCATCAACTGTATTTGACGACCGGAGCAGTCTATTCATCCTCGCAGTGTGACCGATGA
TACGCACGACTAAGGTAAAGAAGATAGCACATAGATAGGACTTTAAAC

>p45
ACCTATCTGCTCTCCGGAGGCGGAGGTTCATATTAAATAAGCTGCTCTGTATCTCCCAAATAATCTTGACCTAGGCT
CGTGGTCTAGCTTAGCGGCAATCCTTTGTGTTTAGCAAACCAGGTACATGCTTCCAAGGACTGCTGGCCACCACATA
TGCCAAAGGTAGGGTACACTGCTGATTGCTCCCTTATAGCAGTGGATAATAGTACTTTGGGTAACAGGGATAGATTT

TCCGAACATTAAACGGTCGTTGTCGCTCTGAGGTTCACATAGATCTACAAGCTTCTTCGTTCTACCCGGTTGTATAT
CTATAAGCAAAAGAGAGAATTGAATTGTTCCTCCAGTAGCTTCGTGCT

>p46
TGTATGGTCTAAATGTAATAAAGAAATGTTATCGGAGCATGGATTTTCCTAAGCCCGTATCTAAAGGATCACAGTAT
GAACAGTTTGTATATAATATAGAGGTCAGGATATTAAGTGTACCGGGACAGTTTGGATAAATGGACCGTTCATAATA
TAAATTGCCCTCCTTTTTCTGGCGGACTGGAGTAAAAGGCAAAGCCATGATAAGTCTTGAGGAAGCAGTTGCTTTCG
TTCTGTTATATATCTAAGGAATCCAATAAGGCTTGCTTAAATTGTAACTTTGGAATGTCTATTCTCCGGACGTTATG
AATTCTTCAAGAGCCTCGTATGGGTGGAATTTAAAAGACTAAGTTATG

>p47
TCCCTTGATTATAGAGCGCACATATCTGAGGTAAGAGGCGAATTTAGTTCGCCGACGTAAGTGTGCACAGTTCAACT
GTAATTACCCCTAAGTGCCAACGAGAGGGTAAGAGCTAGCACGCTGCGGAATCGTATCTAATAACAACCCACAGCAT
ATATGGGTCTACTGGTGCGTTCCGCTTGATGCGACTTCCCGTCTATCTAACAGAATACTTTGATTTTTTGATCTGTT
CGTTGTGGCCTCTTAAAGCTATCCTAATTGAAGTTCTATTTTTACCAGCTGGGGTTGATTATCACAACTTTTGGTTT
TATTAAATGTAGTGCATGTCTCTTCGAAGGTGCATGAATGTCTAGAAT

>p48
GACATGTGGTGAGGTTAATCCAATCAGTATCCGTGAATGTCACACGACACACTCAGTTGCGAAGATTCTGTGTCGCC
CATACGATAGCTCGATGGTGAGTCCTGCTATTAAGGGAGAATTTAATAGGACCCAATCCTAAATATGGGGAGGACTG
TACTGGATACTCAAGTAATAACCAAAGTTGTTGTGTAGGAATTGAAGAATAGTCTCTTAAAGCATCTGTACCGGTCC
TCTAGAGAGATCTCCAACGAACACGTAATTTCGGGAACACGATGGTGATGGAGATGAGAGTAGTCGTGCAGTCTACT
GTTAACCCTTAGACGATTTCTTTTTTTCCAAAAGTCGTTAATCGAGGT

>p49
AAGGGTCTATAGTCAGCGGGTTACTCTTTCTTTCCTAGTACCTTGTAACTGTCACCTGGAACCATAAAACAATTCAA
ATGTACGCCAAAGCTCTCCAGATGAGATACCAGACGAAGTTATTAAGTCCAGCTCTTGTTGCTTTATTTCTTAGTTA
GCACGAGTCCAGAACCGAAAAATGCGAATTCTGTTTCCACCACTCCGAATACAAGCTCGTAAGGAGCGGCGCGTTGG
TCGCGGTGAACCTGTGTACATCGATTAGAGATTTAGTTAACGTTAGAATGGTACTTGAACCCCATTATAACATCTAG
TATCGGGTCGTGATGTTGTAAAACGTGCCACATAATTAACTTTGCACC

>p50
ACAAATAGTATTAGTTTTTTATTTCCTTTGAAACTGTCTCCAGTCTTTAACTATCTGACGCAAATATGCTGTAGGCT
ATCATTGAACTAATCGATCTACCGAAAAAGGTTCGTTTTCAGTTAAGTTCCGATTGTTTCGGGAGAGGGTTTTAGCT
TAGGTTACTATAATTGCTCCATAAGATGGACATACAGTCAAAAGCCATAAAACAATCAGATAAGGTATAGACATATT
AGGTTCTGTCTTCGCTGCTCTATATCTCCTTTAAAGGCACATGTTTCATGGTTACACCCAGGATCAATAAGCGCGGG
CCCGGTTCGTTTTTCCATCGTTTTCTTGAAGGGCTATCCTTACCCGAG

2. random sequences for dac/DACH1

>d1
AACTTAAAGTATGGGGAACTTAAAAGATAGTCGAATACCGAGACTCATACTGGGCTCTTCCGTAGGGCAACAATCCT
GAGGTCATCTGAGATACAGATAATTCTAGGTACACAAATGTATTTAACCGCGTCCTCGGTAACATGTTTTACGCCTT
TTAAGCGAGATATCAGAATATACGATTCATGTCCGTAAAACTCTCACACGTATTTAGTAGACCCGAGGCGCCAGATG
CTGACTCAAAGATCAT

>d2
CCCGTCAAAGGCGTAGCTTTGGGGCCCTTAATAGACAACTGATAAAATAGGTAGTAACTACTCCAAGCTTTTGATCG
AGCCATTCGCTTCAACGTAGAGGGAATTTCGATCTAGTCTGTACGAGATATTCACTAATAGCTCTCAATAAACGTGT
TGGATGGGCACAGCGCCTGTCAATTGTACATTTAACATACAAAATCGATTGTACGCTCGACTACAGCTCTTGTTATC
AACAGCCTAAAACTTT

>d3
ATTGAATATTTAATATACGGGAAGATGTGGCCGTTTTTGACAATATTTTAAGCATCATGCGTATCGTGCTTACAAAC
CATAGAGGCATAGTAAATATTCCCACAAAACAAAAGACCGGGCGCAATCTGTACTAAAGACAGGAGATGGACAGAAC
AGTCACATGTTAACTGAGAATTCTTTGGCTAACGTTCGGTCAATTTCGCCATGGCCTTAGTATACTTTTTGAAATTC
ACGTTTCATTTACATC

>d4
GGCTATCTCAGAGCATTGTTACTAGCAGTGGACAGCAACTCTCGAATTACCAACCGCTCTATACCTGAATCGGAGTG
CTTCTTACTATATTACCTATGCATGATCTAGATACGTATCGATTGTCCCTTAAAACACATTTTGCTCTGTGAGGACA
AAGCCACGTTCGACTGCGCTTCCCTTCTAAGCCTAATTTCGAGAGTGTATCACATCATATCCCCTCTGTGATCTAAA
AGACGAAAGTCTCTGG

>d5
CAGTTTAGAGATAAGGATGATTGAATTTGCAGGGTCCTTATGGTATAGGGATGAGAGTGATTCACTACCAGATGCAA
AGTGGGAGATTACACATAGCATTGAAGCCCTCAGTCATAAGACGATCGGTTCTGCTGGCTTGATCCGGCCGAAGCTG
AGATGATTCGGCTCTTTGCCAACGATTGATCAACTGAGAAAGTCTTACACGAGTGGCTACCCGACTCAACAGCTTCG
TATTAGGTGTCTCGCC

>d6
AGGTGGTAAGTGGGCGGTATGCTCAGAATTTTAAGTATTATATTTCTCTCAATAGACTAAGCTGCAACCACGGTACT
TCATCGTCTAGCGAGTAGTACCAAGAACAATACTGCATACTTTGCTGTTGTACCTCAAAATCAGCTTATCGACTTTT
ATCGGATAAATGCCAACCGGTACGTTGAATATCACTGTTACCTTTTATTGTCTCGTTAATGATTCTCCTCCAGGCAT
AGTCTTCCACATACTA

>d7
TTTAGCCGGCAGGAAGTTGCCTAGTCTCACATTGTAATCTATTAGTAACTTACTTTTAGGATCAGCGGCAATAAATA
CGAATGAACATCAATACCATTATTTTTTTAATGCCCATGTAATAGAACAATGGACCTTCCTCTTAGTTACTACGGT
CGTTTAATCGGCACCTTGGGAAGTTTGATCCACGCCTATTACTAAGGTAATTAACACCCACAAGTGGAATCAGCTTC
GGCCTCACTAATTACG

>d8
GTAAATTTTAAACAATAGACGAGCTGAATGGGGAAATTCTGAGCTCGTGAAGCAAAAGTCGTATGTATTCCTTAGCT
TAGTCTACACTCCGATTTATCATGTACTATATCCCCGATTCTGCAGTTTCCGAAGGGTTTGAATTTATGTTACTATA
TCGTCTCCTGATGCCCTGCCAATGGCCCTAGTAACAATAAAGAGGCCGTTGCACCCATGGTTCGGCATTTATTTGGA
CCTTACATCACGGCTT

>d9
GATATACGCCATTTAAATGTTTATCAACGAGCCTGTAAATTAGCTGTTGAATGTTAAAACAATGTTCGGCTGGACTA
TCGTTACAACGAAACCTTTAAATTCCTCAAACTACGTATATTCTTCGGTAGAAAGGTACATTCCTTATTATAACACT
ATATCGGAGATCGCTCCGAGAAGGATCGTAACATTATTTCTTAGAATCATGTAAGAATTGTAAAAATCCGACTTATC
ATGCGTAGCTATACTG

>d10
ATATGGAGTTGAAACAGATTTCTGTCCTAGTTATGAGACTCCCGTACAGCTTAGTTTTTCCAAAGGCAAAGTTCTGC
CGACTTTATAGACTGCTAAAGGGATTGGCGGGTTTCTATGCTTTAAGAGAGAATGCGCTTTTCCCTCACTATACATT
TGAGAACTAATATTATAGTAGTACCTTAATCCCACAATTATATTAGTAATACGATAGCTGAGTAAGCACGATGTTCA
AGTAACACATGCCCCA

>d11
GGCAATGCTGCTTTAACACATGTATTAAAAAGTGAGAGTGGAAGTCTAAACCAAATTAAATACTTTAACACCTTGAA
TATTGTAGTCCCATCGTCATACTGGCTCAATCATGTAAGGTACGGATTTTATCATGGACGCGCACGGGAGCATGTGT
TCCGCATTTATTGTTTAAATGGCTAGATATCTTGACGGTTTCCACGATATACAGAGCGAGGGCGATAGCTGGAATAT
ACACATGGACTAACAG

>d12
CCTCAAGACAACGTCCCAACTTACCTCTTCAATTGGTACCCCCTAACGCCTTTGAGCCCGAATAAAAATTGACTTCT
TCTCACCTATAACTGCTCACGAACAAAAACGAAGAATGAGCCATACGATCCGACAGATTCCACAAAAAAATGATGAA
GTATGAAAAGATTCAGTAGTTTTATGGACATTAAATGTACAAACAGATCCATTTAATATTAGTAGATCAGTGATAAT
GGATGTATAAGTCAAA

>d13
ATGTACAGTATGGTAGCACGCAAACATACACTGTGCAACATTCTTCGTTATAACACAGACAGTCTCTACACATTACG
GTCTGATGTTGGCATATCATCTACCTGATATTCGGCAATCCCAGTTTAAGTAATCTTTTCTAAATTTGCACAGAATC
AATTTACGCCAATTAAAGCGACGAGTATGATACCTCAGAGAACATCGAAGCTCAGGGTCGATAAATATCTCCTTCGA
GTAATTCGTTCGTTGA

>d14
GCCCTTCAACTCATGCTGTTAAGAGAAAGGGGTAACACAGCGTATATACGCATTTTTCAGGTATATTAGATCCGTCT
TGGGCCCGCGAAAAGAATTTTGCATGTGTTGTATGAGTTTTGTTTACAGAATACCAAGATAAGAATGCTTTGCCAAG
GCATTATCTTAATGACCGATTAGGTAATATCGTTCCTTAAAATTATGGTGCCTGAAATAAAATTGCTACCACGGATT
ATTGATCCGGTATTGC

>d15
GGCTTTCGGATTACCGTGGGGTTTAGTGATCACTAGTATGCGCACAGAATATTTTTACGTCCGTAGCATTACCCGAC
ATATTACATTAACCATGGACGACACTGAGGCACGGGTGGCTAATAGGGATATCAGGCGACTATTGATCTTGATGCAC
CGCGAGCTTAAGTATTTTCCTAAGAAATTCTTGTTGGACTACCTACACAGGGAGTACCTCATGTTGTTTTCGGATTA
AGATCTCATGCCGCGT

>d16
TCTAGAGTGGACCTATACGCCTTGTGCATGCTATACACCCAATAAATCCTATTTTCATAATCTTCCTATTAAGCTTT
GCAGTTTTATAGAGCGGGTGCATACAACAGTACTTGTAGTACACCCAACCACAGAGAGCTTGCCAAAAAGGGACTTA
CGATACCCCACATAACATGAGCCTATAACATTTTAGATGTTTTACAATGAAGGCGTCTAGGATTGCTGTATAAGACG
CCATAAATTCCAGTTA

>d17
ATGCAACGTAGCCAGAGGATTGCCCTTTTATACATAACGGGTCCTATTTTATGAATTCAGGAGCAGGCGGGTCCCTG
ACAGATGCTGACCTCGTGTATGTGAATTGAAACTATTGCTGGCGAGGGAGATTACACATGGACTAACTGAGTTCATA
TTCCAATCTTCGGCTTATTAACCGCGGATTGATTCGTAGCGGCGCGCCTACAGAACGAGCTATATGGATTTAGGAAT
TTTTTGACCGGCGTCC

>d18
CCTGCTCAATTCGGAGCTAAAGCGTGGCGCGTATAAGAACGAATAACACTTTCCTAAAACTAGAGACGCATGCGAAA
GCAACCAGAGTTTTAACTAAGCTGAAGTAAGGATCATTACCTCAGCAAGTTTATTGTGAGTTGGCTCCTTGAGGTTT
ACAGCCCACATCGGGTTGTTCGCGGGCGGGCCTACTCTGACTTTTCAAGGACGTAATTATATTTCTCATTGATTACG
TAGAACTCGATTAGAC

>d19
ATAAAGTGCTTGGAAATATGTAATTTTCATGTGGTGTATCCATAACTAACCTTACATGATATATGTTCTCCGACCGT
TACGAAGAACATGCCTCCGGGGTAATGCACGGGGCAGCTTTTCTCATATCTACAATGCGTAAATATGGGTAGGGTCG
ACATTCGCTCGGGATCTAGGCTAGCCTAATATTACGGATAAGGTTAAATATCGCTTAAGCTTGAAATCAATCCTTCT
CGGTGCGTGATATCAA

>d20
ACCAAATCTCCAATGTCTTACTCTTGCAGTCATTCCTATTGTAGGTCATGATGAACACAACCCAAACGCGGCTCGTA
TTATGGTTGACCGATAGGCATGTCACATACATAGATATACCAACTCATCCTTGTTGTATCATCGCAACTACGTCGCT
TATGTACACGTTTCCGCTCTTAGCGACAATCTGAGGTCTGCGGTTTGCATCAGAAGTGTCGTCCGTAAGGAACGCTA
ACGGAAGGCTACAAAC

>d21
TTGTCACGGATTGCTTTGAAGTTACTATGATTATACTCTCTATACAATGGTCTACTAGGCAGGCCTAGCGTTGATGA
CCTTGTTGGCCATCAGCCCTAATCCCACCATTCTCAACTAGTTTCGTTACTGTACTTCAGTAAAGGTTTTCCCATAG
TCACACTTAAAACCGTTCATTTATATACCCGGCTAAAACTAATGCACTTCAGGGGGTCCGCCTGAGACTTGGTTGTC
CGGTGACGACGTGATT

>d22
AGGACCTAGTAAGGGTCTCTTTTCGCTTGTTCGCAAGGTAGGAGAAGAGAAGTAACTAAAATTCATATATAAGAACC
GCATACAAAAGAGAATACCATTTCTTCTAACATATTAGACATTTAGGGACTAATACAAATGACAGTGCTAGTTCCGC
CGTTAGGAGTCTATATTCTTGGCACAGATTCTTGCACTAGTTTCGCGGCAAGATCACGAATTGGATTGATACGTGGC
TTGACATTTTGATACA

>d23
ACTGAGAATGGCGGCCTACATTTCATCCGGATTTATACGCTGTCGTCTTATTACTTTGAACCCTCATTCACTCGCTG
TCATACAGGGTATACATCGTGTGTGTTCTTGGGCCAAAGTTAGTTCGAGAGAAAAGTCAGCATTCCAAAGTATCATT
GATTTTCCCTTAGAGAACATTTAGTGCTGTTCCGCCAGATCTCATCTGGCAATAGGACCCATGCACATTCCGTACAA
CAATGGACCATTAACA

>d24
TAATATGAGTAAGCAACAATCTACTCCTGGACTATTACGCGATATTCGTTTCGTCAACTGGGTTTAAGATCATGAAT
TATATATACGTATTGGACACCTCAAAGAACGAAACGAAGTGTCTCTTAGTACCACGTCGCAGATTTAAGGACAACGA
AACGAGTTAGGAGGCGGTCTGGCATTCATATTATGACCTAGAAACTTGTTTAGATTCTCGCTTAAATGGCTGCAACC
TTATCGAATCGTATTC

>d25
ACTTACCGATTATCTGAAAACGAATTATGAATCGCTAATGTTTATGAGTATTACGCGCTAGGCGAAGTGTGTCACTT
CTATCCTTTATTATCTTTAGAGGCCTAAGATCGTACTCTGTTAAATAATAGGTAGACTCCCTGTTATGATCCTAAAA
GGTGGAGGGCAATCTGGCCTTTGATCCTACTACACATAGTAGTTGGTGTGCTCGACTCTTGAAAAACTGTTCACTGC
CCTAAATTACCTGCCA

>d26
TAATCTACTCAGGATCTCTCAATGCGTGTCCATATTTGCAAGGTTCCGTGGCGTCTGTGCGTACGTTAACTACAATT
ACGTAGGTCGTACCAACGACAAGAACCAGAAACCAAGAATCATTACTCATGCTGACTAGAATCAAGCAGATGGGAGG
CGTATCACTCTGCCTATTTATTGGGTGACATTGAGTTTTGTAAAACCTAAGGGTTTGTGCAAAGAAATTTATAGATA
ATATAGAACACGCGAA

>d27
AATGGGATGAAGGCAGGGTTCGGGTGACAACGAGCTTGCGATCTATTGCATAACTTCGCGCAACGCAGTACTCAGAG
AGGATTAAACTAACGATAATGTTACATAAATGTATACTTAAACGGCAGATCTAGGAAGAAACATTCTGTAAGGCGTT
TTTCCAGAAATGAGGTTATGACTTTGGCTAACCACCACGGGATAACGGAGTTGACGATACTTGTACGGCCAATCTTT
CACAACTTACAAACTG

>d28
CGCGAATGGTCGTCCAGTAAACTCAAAAACAATAAGGATTAGGCCGACCCCAGAAATAGAACTCGGTTTAAACACGT
CAGTAATCATGAGATACATTTTTACTCTCCGTGACTAGCGTCATCACTCAGGCATGGTACCTTAACATTGTTACAGC

TTGCCCATGTTGCAACAGAATTGTCTATTCTTAGGACACAACAGTACGGAGCCGTTTTTGGAAGTATGGCTATACGA
TCGCACCCTAGTAGAC

>d29
TGAATGACTCTAAATAAGAAAACCCTTATACTGTAGTTGTTGGATTCTACCCTGCTAATACAAGGCGAAGGTGTATA
GTCATAGTCCCGTAGTACTATCATACATACAGCTCTTGACCAATGATGGAAACTGGATCTACAGAGCCTAGCAACAA
TAACCATAAATTAATGACAGTGTTCGGGAACGAATTTAAACTCTTGTTCCATTTAGAACTTGTATTTCAGCCTCAAG
GAAATTTCCTTGCTGT

>d30
TATGATCAGTTAGAAATAAAATAGTAAACCGAGTCGGTGTGTAACCTGGATATCAGGAATATGGAATTTCGCATATT
ATAATGATAGTGCAAAATATCGACATTATTGCAGTAAGTTAACTTTTTGAGTGTGACCGTTACATGGCTATGTAACT
ATTCCAAATAATTTTCCCGCAGCTAGACAAAAAGCCAATGGGTAACGTAACTACAATAAACACAAGTTTGGTCTTTT
CTCTTAAGTGAACGTT

>d31
TCTGATCTGCATACTACTACACTAGTGAGCCTAATGAGCAATTCTGCTGGAGGACTCCCTGTCGGGAAATCTCACAA
ACGCCTTTAAATTAGCTTGTACTTACCAGCATAATCGCCCGTAAGATAACCTAGTACTCGTCCGCCATTATATCGCT
CAGCTATAATCTTTTAATTATGCCTTAATATGTGACTTGGCTAATAGGGTTCTACATTGTTCCCTAGATCCTTTTGA
TAACCAAAAGGTAGCA

>d32
CGTCTTTCATCATTTATAGTAAGAGTATACTGACAAGGGAAGTGGAAGCCAACATACCGGTACACGACTTTGGGAGC
GCAGTGGAAAAATCCAATCGCCCCGTTTTTCACGTCAAAGAGACCCCTGAATTCAATGCGTGAGAATAAAACAATCA
GGAAAAGTTAGCGTACAATGTGTATCCTATAATCGCACCTGTGGTTCTCCAGTTCTAAGACCAGTGCCTTACTAAGG
CTCGAGGTGTGAAAAT

>d33
CCTCAAACTTTCGGTCACGCTTGTTGATTCGGCTGCAGCAACTTACTTGAGACTACGCACAAGTAGCTGATCTTTTT
TATAGTTAACACAATGCATTTATCCGTACAAAAGGCTTTAAACTTTCCCAAAAGACTGCTGACTTATTATTGTCCAG
GTATTTTGACGATAGCCGGCATAAGTCCACACGAGATACTCCGCCATTCAACTTATTCAATGTCCTGTCAACAATTG
GATACGGACTTTGCAT

>d34
TAAGCAATACTTATAACGCAGAAGCCTTACAACAAAATTAGGCGTGGCTGAGCAAGGATAACCACTACGCCTTAAGA
GGCGGCCTTAGGGAATAATGTCAAAATCATATCGGAGCTCGTAAGGGGGTAATATGCTGATTATATGCCTAAACATA
AAACATAACGCACACTCCTATTGATGTGGGAACTTTTGACCCTATACGATACGCCGCGTGGCTGGCCAGGTAACAAC
TCTTACTGCACGGCAA

>d35
TTAAACACATGGTATTCAAAAGCAGAGTGATGGTATCTTTATAGTGAGTTTGCGAATCCATGATCACGAAATAGATC
GACACTTAAGATCGTCATAACTAATTATAGTAATCTTATACTTACTCATAAGGTAATTGATTGCCGTGCATGATAGT
GTGACATTTGTAAGTTGATCATGAACGTACCAAAAGCCGTATTTAAGCTTAACCAATACCACAAATGGTTTCGCAGA
TCTAACGTTCCCGTGA

>d36
CGGTTTTAAGGATGTACTCTAACCACGCTTGTCCACCTCGGACGAAGAAAAACGACGAAGCCATCCGTCCAGAAACA
TAATTATCTGCTAGAACAACGATTATACAGTCCGATAATAGAAGGGAAGTAACTATCCTCATAGTAAAGGGGAGTTC
GTAGTGGTTATGAATTATGGCTGAGATCTGGGTATTGGGAATAACTAAGTCAATCTCACGATACTTAGAATGACTAG
ATCGGACTGTTGCTGC

>d37
CGATCACAGTGTAGGTTTAGAGCGTATCTACCTTGTGAGGAGATTTCCTCATTTATAGCGCTACGGTTTTTACCCGA
GATGCCTTCACCATATCAATAAGGATTCATCAAGCGCCTCATATTCTATAGTTTAAAATACTTCTAGCGACAAATAG
GAAAGGGGTTAGATGTATCAACTGTTATACCTATCTGTAACTGCTTTCATAACCCTAAATCCCGTAATTGGCGCCCT
AGATCGCGCCTTACTG

>d38
TTGGTTACACGAGTATTGCATAAAGGCTGAGAGAAAACTTCCTATCTGGAGTTTTTGCCCTTTGTAATCGGATAGTG
GACAAACGATGCATTTTACTTTTGTTCGGTTTATCCTCAGCGCCACGATACTAATAATGCTACTCAGAATTCAAAAG
CAGGAACACGTCCCACGCTCTATAACGGTTTGCCTAGACGTCAGCCTCGTTGTGATGTTTTCATAGCTAACGAACGT
AGTATTTCTTTTAGAT

>d39
TCATGTGATGTTACTTATCACTCTGATCAGGTTGATCACGCGAGCCCAATTAAAGCGGGACTAGAAGGTTTTTTTGA
TGATCATACAACAGAGTCCTTTAAAAACAGTAACTAGGAAAGGAATATTTTGTTATTGGGGTCATGTGTCCCAAAGA
TCACCTAGTACAAGGTTCTTACAACTAGGCGAATCATCTTTATTTATGTCGGGCGAGTCTTTGTAATTTTTCTCCCT
TTCAAGGGCATAAAGC

>d40
ACTGTGGATTGACTTAGAAATCGTAGAGCAGGCAAAAATAGTCTTACACTACAATTAAATCAGGAACATGATCGATG
GTGCCATAAAATAATAAAGTTGTTCCAGCTTTAGACATGACGCAAACGCTTAGATGCTAGCAACGTAGACTAAAAGG
TCATGGATGGTCGTTTGTAAACCTGCGGTAGATATAATAGTCGGAGGTCGAATACGCATACTTTTTATCGATTTATG
TCCTTCTTTTGGCCTT

>d41
ATCAAGTAAAAGTTCAAGATGTTAGGAATTTGGACAAAGAAGACTTACGAACCGAAGGTTTAATCGCCACCTTACCT
GTCTTAGTACAGACTGCTTTTGTGGACGACTGTCATACTTGGCATATCTTGAATGTTGTCATATCCACTATATATTC
GGCGCTAGACAGGTCTAGTTAGCTGGAGAGTAGTTTCACTATCATATTGTAAACGAATCAAAACAGAGCTCGCCATG
AGCAACGTTCTCTAAA

>d42
GGTGGTAGTCTGAAGGCTCAACCTAATGAGCATCTGACTGTATTGGGGATTAAAGTCAGCTCGGTTCGTAATTATGC
GAGGCCAATGAGTGAGTTTACTTTTGTATCATGCGTCATTCTCCAAAAGGCGTTCCATGTGACTAACTTCTAATCTC
GTAGACGGGTAAAATTCACAGGTTAAGTGATGGAGTGCCAATATAACATAGCAAACCTCAACGTGATTACCCCCCTA
GTAATCTAACAACGGA

>d43
TGGCCCTGCTCTTGGTGAAATCTTTGACCGGTACAGAGTTAAAAAGTCCGTATTCGCCATAACTTAGTGGCCTGGTT
CTAGAGTACGGTAGTATTTTGAAAGTAGTAAGTACCTCTGCTATTGCATTTGAACGCACAGTCACAAAAATTGCTAG
AATACTGTCAAAGGTATTGCGGTCGCTTCATTATTCGCTACAGCTCCGCTGAAGAGAACTCTGACGTCAATAAGGTA
CTTTAACGCTATCGCA

>d44
AGTCATCATATAAACAGTAGTTGTGGTGGCCGTTCTTTATTTTCCCGAGGCACTTGTACCGAGTACTTTTGTTTCGA
TCCATTCTAGGTGACTCCTAATTGGAAAATTAACCCTGAGCAGATCATAGGATGAATACTTTTACTTCAGTAATCGT
TGAATCAGGACAGGAGAGCTGGACTTTGTCTAAACTACTATACTCTATTACTTGTGAGTTCGATAACTTATATACTG
TCGGCTAGTAACACCA

>d45
GTCATCTAACTGCGTATCCCAAGTCAAAATAGGAAAATGTAAGACACTTGCTTTCGCTACTAATTTTGTTACAGCTT
GTTGTATACTCGGAACTCTCTATATCTTGGAGGGTTCACACTTCTTTTTAACTGCTTGATTTTGTGAGCCTGATTAA
AGTTCTACTTGAGACTGACAACTTATAGTTGGATTGATGCCCGCGATGTAACGTCATGTTTTGGTTCCGAAGTATCT
AACATTAAGTCAAACA

>d46
TCTATGTCCTAGTGTGCAGTAGCATCGTACGCTACTCTTGCCTATTACGCGTTACTTTGAATCCAGTTATTACGTGA
TAGATTCTAGTCGGAATCATTCTATAGCGCTTTTGTTTCCCAGTCTCTACCTACTTGGAAATCGTCTGTGCTGTGAA
ACATCTTATCTACAATAGAAAGAGAATTCAACTGTGAAAACAAAATACCTCCTCATATAGAAAGGACAGACTAACTT
CATAAGACTAGAACGA

>d47
TAAGTGCCAAAATTTTACCCTAATCACATTGAGTTTATATGGAAACATCGCGGCTTCGCATAGGTATTATATGCGCG
GAGGAGGTCTAATACGAAAAGTTAAGCATAGCCCAGTTCTGGTCTACGACGCTTTTAACCACCATCAAGTATGACGT

TCGTTAATTACTCCATCTAGAGTTAAGTGTCGTTGTCTAAGGTCCTTGATATTTGATAGGACTTTGCTTAAGTACGA
AATGCCAAGTGGATAT

>d48
GCACCCCTTCCCTTTAATTGTCTTAGTACTTGAGTTTTGGAACCAGCAAAATATAAAACCCCATTGGTAGTACAATA
TGTTTGGCAAACTACTCTTGAAGGTAACCGATTCTTAACTGTGTATTGAGTACGCCGCAGCTCCACTACAGTGACGC
GCGTCAGACACTTGGATACTAACTATACGTTACTGGTTTATAAACTAGGGAGCTTTAAACATGATAAGTACCACTGT
ACTCGATCAAAAATAA

>d49
CCCATCACGCTTTATTGATCCTTAATCCCAGTCTGGGTATAATACTGAAAAACTCCGTAAGCGGATCTAAACAGCCA
ACTGTTATAGTGAGGGTCCTAACTAAACGTCAGCTCTCGCTCCATGTCTAGCGTATAAACGTCAATCTTTAGCGTTC
AAACAACCTTTTTTATAGGTTGGCGCCCTGAATGCGAGGGAATGCGGAGCGAATGCTGACAATGTATACGACTACAC
TTCGTCTAGTCGTCAC

>d50
AACGTAGCTTTTGACTAACAGGTAACTACAGTATTTCAGTTTAGATTTCAATATCCGGTGGTGAGTAGAATGCTTAA
GAGCTATATCATATGGCAATGGAATTCCCTTGATGAAGATAGATAGCCATCTCTAATCCTAGTGGATATATGATCGA
AGGATGGTAAACCCCTCAAAGCATTATAAGAAATACCAGTCATGACAGCATCGTATTTCTGTGCAGAGTAGTAAGAT
AGGCATCGTACATTTC

3. random sequences for inv/EN-2

>e1
ACTTCATATATCGTACAATCTACCCGGATGAGTATGCCTTGTAGCACTCTCAAACTTAGGACATAAATGATTAATAG
TTAGACCTACCATAAATGAAAGCGCATCCAGTGGTCGTAGAATGGTATAGTATAACCTTCCAAGACTTACAACGAGT
GTTGATCGCACGATTACCGTAAACTATGTTTAAGTATTTCATCAAGAGCTCTTATCATAGTGAACTTTTCGTCTGTA
AACACTCCTTTATTTGGCGTCCAGTAAAAGTATTCGAAATCGATTTTACTTCGTCATCGGTGGAGTGATGGCAATGA
ATCTGTACTTATGGTGACCAACAATGAGGAGTAGATACAACTATCTGGTCATTCATGAATTTCTT

>e2
TGCATAGTTACGCTCGCAGGCCTATGGTAAGACATCCGTATCGAAAAGCACTAAATGTTGCTCCATCTGCAGATTGG
TCAGAAAAGTTCAATTCTCAGGGCCTAGAATTATGCTACTTACAAGCCTAGAATATATTTATCTACTATTCTGCACC
ACCCAGTCGTAAGAAGAAGTCTTTGTGAACACTTACAATCTATGCTTGGACGCTGCTTGCTGTTATTTGCGCGTCAA
CGGCGTTATCAAGTATCGACTGAGCCTGTAGGCACTATAAACATCACAATCTACATGAGGATTCCAAAGTTTATACA
AACTGGTCGTCTACACTACGAAGTAGTCAAAGGGAATATGATGTAACTTACGTCAATATAGGAAT

>e3
GATATGACGAGTGTGTGACTTGGGATTGCTATACCCAGTGGGATGGTCCTATTTCTCTAATGAGCCATCGCTTAACG
ATGCACCTTAATGAGATTTCTATACAGAATCCCATTTTCATCTATGCTATAGGTTAAAACGACCGATGATTTGTAAC
ACGGTCACCTAGTAATGCAGGATCACACTTAGGTGCATTTCTAGTGTCGTGAATGTAAAACGCGGCTCTAAACTGTT
TTTAAATGAATTTATCTGTGCAACGATGCGGCTATTCTTCCTAGAATGATCCAGAAGATGACCACCACGTGGAACGG
TTATCGACAGCGCTCGTGGCCATACCACAAGATCCGGATAGCCGCGTCCTACATACTACGAAGTT

>e4
AACATAGAACCAGTGACGGATGTATCATTGAAATTTCTCCCAATTAATAAGATATTGCAAGTCCTTATTCTTCTCAA
GATCTTTCGCAGTATCCCTACGTATATGAGGAACCGGCTTGCTTCGCAAATCCTTATTTAATGTAATCTCCCCCCAT
TCATTCCCGTGGGTACAATTTGATTACGCAACGTTTACAATCGTTGGCATCATGGATTAACCTCACATACAGAGTTA
AACCCGTGCCATACAGTTACTCACGCTGTTTTTTGAATTGAGAATCTTTCAATCGTCTCATTGAGAAGATTACTAGC
TTATACAGCTAGTATGCTTCCAATTGAGACAACAGTGTGCCTCACTATTAGGCTAACTCATGTGG

>e5
CTTTCTGCGTAAAGATTAGGCACTATAGCATATGGACATGAGACGTGAATTCCGAGCATCTTCTTTCGTAATATTCT
GGTCTTGCATCTTAAGAAAATTAGCTTTACATGAAGGTGCACAGATGGTTGCAACCGTCCGTGGTCCTTTAATAAGT
GTGGAGAAGCAAGAGTAGCGTATATTGTATGGTGTCAACACTAAACTTCCAAGACGCCTTCTATATAAGATGCATAG
CGTTATGCCCCCCATCTCGTTCGAGGCTTTAAACCAACATTCCAGATGGTTCTATAATGCTATTTCTCGCCTTGAAT
TCCATTGTTTGTCAGTTGAGATTGGGATAAATTAGCGGGTTATATGAAAAAGGGTTCATAGACCG

>e6
TTGTACTCTTGTATAGACTTAAAACCTGGGTACAACCTACCCATGTGATCCCTGGGAAGTGTGGCTATCTACAGTTA
TATATGATAGTAACGCAAGTACAGTCGTACATTTGTTCAGAAATTTTATGTTTTACAGGCGCAACATTTGCTTGTTC
GTAATAAAGATTTATGTCGCTCTAACGAAACCAGTTTCTCCCTATACTCATTATAAAGCAGCTTTGACCATAGCGTT
TTAACATTGCAATAGAATGTTGCAGAATAAATTCTAATAGATGACCCCAATAAGTGGTAGTAATTACGCAAGAGTTT
AATCTTCAGTTTCACGGCACATACAAATGATTTTTGACTTGTTGCGTTTGGAATGGACTTTAAGG

>e7
CCGCCTACGTCGCTCTATTAAACGGCACACAATAGATTCGACAGTGGCGCAGAATAAATACAACACTCATTTACTGC
ACACAAATAATCTACCACGATTAGTTCATCTGAGAAGTTATTTCTGCCAATCTAAGACTACCGCGAGATCGGTCGCC
CGCCACGAGTACTAGTTAGCCGAAGTGGGGATACTTCGGGCTTCTACTCGTCAAAACTGTGCATCCCGCAGCTGTTT
TTCACTTTAGAGTGTCGAGAAATGAATCCACCTAATTGCTAAATCCTTTTCAACTACTACAGTTTCTCCGCAGGATA
AATTTACATGAAATCACGTCATAGTACCCAAGATAACGCGGATCGCGTACTCTATGACTGTAGAG

>e8
AAAGCGGAGTCGTTGCAAAGTTAAGATATGTTAACTCCCGAAAAAGAGGAAACGTTTCTTTGGGTAATTCCGCCGCA
GTAACGCCTATATCACGTCGGCCGGTAATAGCTCGTGTTAAAATCATAGATAGCTGAATTCTAGTGTTGGTTGAAGA
CCCTTACTGTCGACAATTCGCTATCTTTGAGTCCGACAGAATCGATGTAAAAAAACGAAGATATTCAAGGGAGGCCG

GTATGTCCCGCATGGTTGTACAGGATTGGATAGAACTGGTGAGTTACCCTGTTATATAGGGCAACAGATGAGGCACA
CTCTTTACATTAATAACTCATAACTACCAGCAGCTTGTACAACTGTTTAAAGATCGCATATGCCT

>e9
GGCAGCTTTACTGGCCCAGTTACAATCATCAGGAGTCTACGGAAACCCAGCAGAGTTAGACACATATGATGCACTCT
GCGGTGCGATTGGGCAGTTCGGCTATCTCTGAATTATCGTACCCTTTGTCCGGTCACATAACCCAAAATATGCCGCC
GGACATCCGAGTTTAATTTGTTCATGGACCAGCCGTCGGATTGTGAGACCCAAATATGGTTTAATCCACTATCCTAA
TATGATTTAATAGGAGGCTATTAAGATAGAAACTCAGGACTGATGCAGTCGCACTAAGTAACTCACATCTGGGTTCA
TTGGCTTCACTATGCGTTACCACCTCTTATAATCACCGAGTGTTAACACTCCTAGCATATACACA

>e10
ATAAGTATGAAGCGGGTGATATACAAGGACCACAGTTGATTCGTCTATTGGAGTGTTTAATGGGGCTACTTCGAACA
CAAATTAGCTTACCGGCATGAGCTTCATAATTTTCAGAGAATATCCTAAAAAAAACTATGTCCTTAATTAAAATCCC
AAACACCAATGAGCGGCCAAGTCACTGGCTGTGCAGAATCTCCTAACACTCGGACTTAGCTAATACGTTCCAACGCC
ATGCGGTTCGGAAGCCTGTACTCCAGCGTATCCAAAGTATAAGCATCGTTTGGTTAATCGACACATTATTTATTTAT
AGAATCGTTATAAGTCGTTTTGTTCATTAAGTCCTAATAAGATCGGCCGGGGTTATCGTGGATGT

>e11
GTACGATTCACGACTATATCAGGCCCCGCATTGTTCAACATACTCCATTTGTTTGAAGTGTTGACTAGAGCGGAAAA
GCCTGGTAATTTAACGCGCAATTATTGCACAAACCTAGCATAATGCATTGTACATTTAATGTGTGTGAAGCAATGAA
TAATAGGCGGTTTTCCAATCATGGATATCAGCATCCGTACAACTGCTTTCGGTCTCACCTACGGACGACTTTGAGCC
GAGCCCGTGTCTGCTATGACGCCAGGAGGATCAGGCCTATTACTAGCTAAATAATGTTAAGTATCATCAGCCTGCTA
TCTATGTCTTATGGTTATAGCCTTACGTATATATATTGCAAACCATGTGTTGACCAAAGTGTTAA

>e12
GAAGTCAACATTGCCTCTTTTCACAGATCTTCTAACTCATACTGTGAAGATGAGACGTAACGTTGCATTAGGGACAA
TAAAATCCTATCGCTTTATGTTTAAAGACCTAGGGTAGCAATTAAAACTATAAAGTACCGTACCATGCATAATATTA
TGTGCAACGATTGTAAGATATCGTAAGGATACACGCTAGCATTAGTGCTATGTATCATCACTGCACACCGACGATTT
ATTAGTGGCAATTCTACCATCGTCAATCAAAACTACATGTACTGTCCTGACTAGGTAAAAAATGTAACTTAGCCGGT
CACGGGGGGCCCGATGGTGATAAGAGTTAAGTAGCTATATCGTAATATATTACCAGGTCATGCCA

>e13
GTCATGAGAAATATTCGTGTTTTTATAATGGCGCATTCTCCATTCAGTACAAGCTCATTAGGTAGTACTAGTATCTT
AGGTTCGAGACGGAGTCGAGTTCGTACGGACAACATATTAATAAGGTTGTGATTCTCGTTGAAGTATTTCATGGAAG
CGAGATAGATTGCCAGCAATTACGGTGTATCTTTCATAATAGTGCCGGTATTTACTGTTCTGAGAACAATATATTGG
GCACAAGCAATTTGCCGATACTGGACTCACGCAAAGCGCTTCCTGAGTCTTTTTTACGAGACCTACCACGTAACATA
GCGTCATCCTGATACGATTATAGAATTGCACGAATTAACCTCCACACATCAATGAAAAAATCTCT

>e14
CAGACAAAATGCTTATTGACAGTATAGCAGACCCTCTGAGGGTGTTAGCTAGCTTCATTAGTACAATTGCTTGCGGA
AAATTGATGTACCCTTTATAACTACGTGCGAAGGATGTAATTATTTGATGTCGCTCAGTCTGTAGCGGGATGACATT
GTGTATACATGTCATAGGATACTACTCTAGATATTAAAAAAGCTAAACTTCCTAAAGCTGTACCTTTGCTATTCCGG
GATGGTTCACTAGAGATGAAATGTCATTTAGACTAGGTTAGAGGTCACCTGTAGTTAAGTTGCTGGCATACTATGTC
AGGCAATTCTTGGTGAATGTGCTTAGCGCCTATGACGAAACCCTTCTAGACTCGATACGATTGAA

>e15
TAATTAAAAGGAAACGCCGGAAAAGTAACACAGTGCGAACTTGGGTAATGTGTTTACAAGTCTTTAACGTGGGTTAG
TATTAAATATCTGTGTCTGTAAGTGGGGATTTCCGGGCTAGTTATAGGATTTTTATTGCCGTCCAAGTGAAAAAAGA
CACGAGACCATCTAACAGCTTGACGAATACAAATTTTATGGCCGAGTTTCTAAAGTTCCGAGGGATCATACTTGTCG
GCCAAAAAAAGAGGTGGGAGCAATTGATTATGTAGGTTATATAAAGATTTAAACCAAGATCATAATTATCTACACAA
TCATAAGAGGCAACATATTACGATCATAGTTTGCTATACATATTACGTTGATAAGTAACGATGAA

>e16
TCTTTTCTTACAAATTGGAGCCACCACAACTGCATGTCGGGACAAATAATATATTAGTGTAAGTAGGGCTATAAGCC
AGATCTAACATGTAGGGTGTAATTTAATCGCGTTACTCCGCTAGGTTATAATACAGACTAATTAATTTTTGTTCGCC
CGTCTTCTATCCATAATTGGAATCAGTATACTTCAAATCCCCTTACTTGATATCTGCCGAGTGGAGTTTTAACAATG

TTGATTCTTGATGACGGCAGCCGGTCGCTGATATAACGATTAATTGCACAGCCGATAAGCTATGTCACCAATAACCT
AGAGATGTGAGCATAAAAATGACTTTAAAACCAGCGAGGCTGTCTGGTACTTGCCGAATAAAGAT

>e17
GCCTACTACGACGCCATACGTTCCGAATAATTCGTAGTAAATTAAGCCATATTTCTGTCGAGAATTTTTAAATGTGC
CGTATATGTGCACATCGTTTACGAGATGGAGTTTCTACATGGGATAATTCAACTATGGTTATTGCTTTTGATGCGAA
CACCTAAGTCCTGATATTCGAATCAGGTGGAGTTAGAACCGATCCTATACGAAGGTATTCTAGGAGGTTGAAGGGGA
CTAGTGAGAGAAAATTGACAGTGCTGGTCAATGAAAACCACATCAGTCCATCAAGATAATCTTCTCCCAAAACTTGG
TGCTTTACAACTTCCCTTTCGACCTTTAAAAATAGGGCTGGATGAATGGGAGATAGATGCGTAGA

>e18
TATTGAGCCACTATATGACATGGAAAATTTAACGACGTGTTATTACTGTTAACTATATTAACTCACAGTCCACTCTG
ACGATTGATTAAGATCAGATCGAGTTATGTCAAGTAGTGAAGAGAGAACGCAGGGAGTATAAAAATATAAAGGGAAG
CCAGTATTGAACAATATGGTTGTAGGATAGATCTCAAGTATAAGGGGGTAAGAACTAGTGGGGGCTGCCAAATCTCG
TCATTCCTAATTAATTTACATGCACTGTCCTAAATGTGCATAGTTTTGCCTGTTGGTAGAATACGCTCGCTCGTAAT
GAAGATTTATAAATCTAGCGAAGATCCTCAACCCCTGAAACTGTTTAATTTTATGAAAGGGTTCT

>e19
**ATCCGCTGGGCGCCGACCGCAACAGAGTGATTAAATAACTACTTAAGAATCTAACAACATATTCGTACTGGCGATGC
GAGTGGGTTAGCCAAATCTTTTGCCTAAAAGATTAAATCTAATTTAGTATTTCTTTTCCAATTTTCCAGTCAAAGTG
CTTTTCACGCCGCAGGATAAATTGTTCACTGGCCTTATGATAGTGGTGATAGCGATTGAGAAATAGAACACTACTAC
TTACGATTCTCTCTGGACGCATACCGGATTAGCAACACTCGATTCAATACTCAATCATCTAGATAGCCTGTCAAGGG
TATTCATATAATCAGAAATACATAAGATTCGTTAGTTTCGAGAAGGATGTCGGGTGGGAATTTCC**

>e20
AATAACATAACCTTGCATTAATGTTACGTTGACATTTAGGTAAGAGACAGTGCGTCTTCAGAAACTTTCAAATCCGG
GCTCACTCTCTTGACACTGTTGAACATTTAATGAAAAGATTCAACTGAAATTATTTGGCACTAAGCCGAAAAGCCGT
CTGTTGCCATCAATCTCATCGAATACCATAAGTAAATTGTACAATCATAACGATTTTTTACGCAATAGTGTCGTTCG
GTAGTCGTATAGTGCACTCGCCTGGAACCACCGAGAAATGCAGCAGAATCTGGTTTCACCTAGCCAAATAGTTTTGA
CGAAGAGCACACTACTGGTTGGCCGTTTCTTTTATCATAAGATCGAACGAACCTTGAGTTTTAAA

>e21
GGTCAAATACACCAGCGACAGTTATTTTGAGAGAGCGTCGGCTTAATGACTCACATTTTTATGGCTCGACTGTCGCA
TGAACTCCCGGGTAGAGCTAGAAGGCACTCTAACCTCATAGCTTGACAAACTACAAACATCTATGGCAATTGGTCTA
TCTTCAAGTCCACGAGAACTAGTTCCGCTTTAAATAAGACAGTCGGGGAGCATCACCCCAACAAGAGCATTTAGACG
TTGCATTTCTTTCAGCAAAAGTTTTTATCTGTTTAGAGTTTTCCCAGAATTTGTAATCGATGAGCAAAAATGATATT
GCCAACCCTTCAGCCAGGAGGCGTAGCCAACAAACCTTCGGCCGACCGCCACCTGGCGGAAATTC

>e22
**AAATCGCGCATGAATATTCTATGGCAGTTTTAGTGATATGATAGAATCTCTGAAATGCGCACGATTAGCTAAAAGCA
CATTACACATAAATCTACAATGACCCGCCCAATCTCTATCGTCAAACCAATTTCGCCACCCGACAAGGTCGGCCCAG
TGACAAGAGAATAGGATAAGACGTTTTGATAAGCGGCCATCCTGAAAAGGTAACATGGCGTAAGCGCCAAAGACAAC
GACAAAAGAAGTATATATAAAACCCGCTAGCTAAGTGGTCAGCGTGTAGGTAGTAGATCTCAGCATCTGGTATAAAC
GTCTGATTCGTAAATTGATAGCCTTCATGATCCATAAGCGTATCTCCGTCGACTTTAGTAATGAA**

>e23
TTTGGAGATGAGAGAAGGGACACAAAAAACGCACACATATAAAATCATCTTTTCTATTGTTAATTTATCGAGACATT
CTTCATAGAGATTCTTTCATGTTTCCTACAAGGGGATGTTTGTCCGACTCCCGAGAGGCGGATTGAGGTATATGAAA
CCCACATAGGCAGAGTTGTAGTTACTCACTTAGCCATCCACATATTGGTTATGATTTTTAAAGAGTCCACCCCTTTT
CGCACCTTCTGGTTCTGGGTTCTATCTATAATACATATTGGGCAAGAAAAGTAACATATCAGGTCCTACATTAAGTC
GCAAATTAATGCTTCCAGTTTGCTACGATTGCTTTAAATGATAGTCCTACCTTGATCTGAGATGA

>e24
CAGGAACGGGTACACATGCAGACGTTTCCCCATTTTTTAGATGCGGCCACGGGGAACTAAGTAGACGATTTGTTTAG
TGTCAGTATCGAAGGATGTTCTATTATCATTCAAGTATGCAGAGAAGGCACTGTCTTTCGTCATTCGTGTCTTCCAC
ATAAGATTATGCCATCTTACATCTTAGTGTCTGAGTCGACGAATGAACTACGTGGACAGCGATATTCGTTTTCAGTC

TGGTAATTGACGCGATGGGATGAATCTTACCAGCCCTACTATGAGCAACTGTGTGGGCCCGAGGCCCAGCTGTAATT
TTTATAGGATATTATGAGGGTCAGACATTTCTCCTATTCTATGGTCTCGACACATTATCTCTTTG

>e25
TTGATGGAAAACTGTGACACAGTCTTTGCGAATTGAATAGGGTCCTATACAACCGGACCCAAACTTTAGTATAATGC
CATGTTCAGATTAATAAGATCACCACCTTAGGCTGAGAAGGTTCATGGGTGAAAAGTAAAGCATTGTGGTATGTAAC
TCGAGTCCTATTTAATCGGTCCTTTGAGCTTCAGTCTGCTGTATGTAACATTTCATCTTCCATTCAGAAGAAATTTG
GTTTTTTTTCGTGACTTTATCCCGAAAGGTTGTATCTCGACCATATGCTGGATATTATACACAATCACGGGTTCCCA
TACACAAAATACAAAGCGACTGAGTTATAGTAGGATCGTAGAACTCCAGGCGGGACTGGCTTATC

>e26
TTTTAGGTATATATCATACTCCACAATCAAAGCTGTGATCTCCTGTTAACTTGTGTGTAGGGAACTATACTTTTCTG
ACTAAATGCGAGGTTTAGTGTCGTCGACTTTAGTAGGCCGGAAGCATACTGTAATAAATAGTCTCACAATTAAGACT
TTGCGCTCAAGCCTATAGCACGGCCAGAAACACATAGTGACTAACGTATCATAGGTGTCAAAGCACAACGTATGGGT
TTATTTTTTGACAAGTTCCAGGTGTCAAGACGATGTAAAGATTTCTCGTCCGTTCGCTCTGACATTGCTGAACGAGC
AACCTACACGTCTTGATCCATTATCAATCTTACGTACTACACACAGTGCGGTTGAGAACTTGCAC

>e27
TCATCCTGCCTAACGTTACGTAGCATAGCGCTTCAGCAGTATTTAACACACTCTGGTACAGCGGGTTAAATCTCCAT
ATTCTGCAATACAACTTTAATTAACGTATTTGTTACAAAGTAGCTTGTTTCGTGACTATGTAATACTATATGCTGGG
TCCAACGCGTTACTGATTTACTAAAACACGAAACGTAACTAATCTGCTTTGGCGCTTGTTTGTTTGAGTTAAAATAC
TCGTGAAACAAAGAATACCTAACTATATGTATGCGGATGCGTGGTAACAAACTAGGCTCGGTGCAGAGTCTGCGTGG
ACAAGCATATTCTCTCTAAGCAGTCAAGTCACTAGCATTGATTGTAGCAAGTACGTGCGAAGGGT

>e28
GCTTCCTCAGAAGGTATCGGGATTCGTTAATGAACCCCCATGGCGTGTAAATTTTAGGAAAATACTGAATTTTGTGA
ATACCGTCACATCCGTGATTCAAACCGTTAAACATGGCTATAATCACAAAAAAGCAATTGAATCATTCGTGAGCTGG
ATAATCACCTGTAATTATACAAACCGAAATGGTGCCTCATCTTTGTTTGCCACCCTTGGCCTCAAAATAATAAGCTA
GTTCTAGTGATCAAAGCACTACACCCTTGACTGATGAAAGGGCATAGCCGTTTTTTATAATAATTATACACGTCGTA
CAATTTAACATCTGGATCACATTGGCGAAACCGGTCTTAGCCCCCGCGTTTAGGTTGCCAATCCA

>e29
CACACAAATTAAACTTGAAGATCTTAGGTCTTAACAAGCGCCATTGAACATGTCGCCCATACACGGGCCCTCATCTA
TATCCCCTCTTTAGGAACGTAAAAGAGTTTAGGCGGGTAATCATAGAATGATTTGCCAATCGAGATAGTCTGATACA
GTAAGCGCATAAAACCTCTGTCTGTTTTTAGGCCATAACACTATAACTGACTAGTGGATTCAATTGCACATTACGCA
AGAACAAACTATCGGTACCGGGGTGCCTTACCTCAGACTAATGAAAAGACACTTCCTCATTAGTTTACCAACATTTC
TCCGACATCCTTGAGCGAGCTCGTGTATTCAAATCGGCAAGAACCTATGAAAATGCGACAGGTCG

>e30
CTCCCATTTTTTTTTGTCTGTAAAAGTTTCTGGCATACGTTTCTGCGGCCAATCAAAAGAGTTGTGAGTCGTTTGACT
GTGGCTTCTATGAGCACCCCCCTTCGCGAACTACGAATCGATTGTTACAAGTTCCCTGTAAACGTGAATACGTACTA
GGCATGTTTGCACCTACGCATTCTATAGCGGCTGTACACATACTGGCACCGCGACGTAGACCACGACTGAGACATAG
ACATTTTGTAATATTACTGACTGAATCTTTCTAGGAGTATGGCTACTCAACCCGCCCTCTAGCTAATAGTTTTCCAT
CGGAGATATACATTTTGCTTCAATGTATTAACATTATTTTCCGATGAATAAGGAGTTCATGCTGA

>e31
GGTAATATAGTACCATCCTCGTCTACTTCTACTCTTCTGCTCACGATTTTATTCACATCATTCATCCATGGAATACG
TCTCAATCTTGAAGCTATCCTGACCCAATAACATAAGTTATGCCGAAGCCTTGACACGCATCGGCTAAGATATGCTC
CATAAAGTTTCTATAATACTAATAGCTAACTTATCAGGAAACTGACAACTACTAGCCGAGCGTTCCTACGCTCTAAG
GGATGGTATTGCGATTTCTACTAGCAAACATAGTAATATAATGGATTTCAGGACTTCTCTTCGTCCACTAAACAGAT
AATATATCCTCTTTAGACGTCGTGGATTGTCCGGGGAAGCTGTTAACGGTGTTCAGTTTATCAAA

>e32
CTTGTACGATCCCGCGCAGAAGCTGCTCGGCCGGATGTTTATAAATTGATAAAGCGACTTATTTAGTGGATTATGTA
AGAACTAACCCATCCCCCGTATATGTATTAACCCTTGTCACTCTTAACCAAACCCTTAAAAGGAGCCATGTTATTGT
GGTGTTGGCCAGAGTATATATATCAGGGGAAAAAGGTTTTTTCCGAAAACCATGGTGTGGGTGACGGCTTGTCTCGC

GAAGTTAAACCTAGTTCAAAATCGTTCATTAAAGTCGGTAGACCTCCTCGTCTAATTCAAGCTTTTTCACTAGATGC
GCTTAGTTATCACATACACTAGATGTCTCCATACTTACCAATGCTACTTTGGGATCGTCGATAGC

>e33
CAGCCAAAGGAATGTAGTAACCCTGAACTTTTTTGATTTCATATTATGTGCTGCACTTGGTCTGTCACGGCTCATTC
CCTTCAATTACAGGTGACTGCCGGATGGATAGGCACATTTTGAGAATCTCTCTAAATGTATGGACTAATAGCGTTTT
TCCGGTCTTTGACCTAAATTTCCCGTAGTAAATTTTTATCAAGTGAGTTTAAGGCTAAACCATTACGCCCGTGTATT
CAAACGCTTGATGAACCGCGGTGTTGTTCCAGTCAAATTGGAAGTGCGTGTCCTGGTAAGGTTAGAGCTTGACTTTG
GCTTACTGCGTATATTATTTGAGTCCTTAAATCATAAATGCCGATCCCTTAATAACTTTTTTACA

>e34
TAAGAACCCATTAACTTCACTTTAAGGTAGATATTCATATATTTCGGCTAGCACGTCCAGCCGGCCAATTCTCAGGT
TATCTCTTTTAGGTAAATTAACCCGTCTAGTCTTATTAAGATAAACTGTATAAAAAAGGATGCCCATTACAGTGGCT
TAAAAACGACTAGCAAGATGAAAACTCTATATGGTTAAAGAATACCGGAGGATTACACTCAGGCAGTCAATAACGGC
GAAACAAAGATCGGCAGTGAAAATCAATGATCCTAGTCTCTTTAATTTTTCCCATTGCAACCTAAGGACTTCCCACT
TGCGAACAAGTTCGGATAACGTGCTTTCAACCAACAGCTCTCGTTCGGAATCTCCTGGGGCAAAA

>e35
AAGTCTATCCCCTATAGCAAACCCCAGGGCTATACCCAAATCCGTGTAGATCGGAATAACTCGAACCATTAGCGGTC
ACTGCCTTAGGATCAATCTCAGCTGAATCGATCCATCCATTATGTAGAAATTTATAAGGCTTCATAATGAAATTGAA
TTATCCGTTAAAATCCGATTGGGTTTCGTCCGTTTTGTCGAAAAAGGAACCATGGTTTAAAAATACTGCCTAGTACC
CGTAAAGTATACCTTAGTTGGCGCTTTTTGGCGAGGCATATAATCGTCGAGTCAAAAGCTCCGTCACAGTATCATCG
TGACAATCATCTTAATTCGGTACGCGTATGGTTTGGTACTTAGAGACCGCATTTAGGGAGTGCTA

>e36
ACCGATACTCTGGCGCTAATTTTTAAAGTAAAAAGTATTGCGCTGGTGATCAATAAACTCTTAAGCAAAAATAGAAC
TGACTACTGAGTTTTACAGCGACGCAACTAAGTTAAGGAAACTATCGGAGAAAAATAGGAGATGTATCTAGTTTTGT
ATTATATAAATATTGACTTTCTTAATTAGCGTTCTTCGGCACTTTAATGGACGCAATTTAGACTGTTACGGCACCGC
GAAATCCCTGTCTGAATTAAGTCACGCAATTACTGAAGTCTCGTTTCTAGTTATGGTGGAGCGTTTCTTAACGTATC
TTAACCGTGTATATACTTAATTTTACCTCACGACCGTTATTTGAAGTCATATTCTCTTGTACCCG

>e37
AAACCATAAAATAACGGGTTGAATCAAACAGAATTGTTCCCTTGGCTTGTTCTAAGACAGCATTGCCATGGGGCTAT
TATGGATGACCAGGAGCGAGGGAGTTCTAAAAGACCATTCCTAAAAATGGATAAGTTTGATTCGAGGGTTGTATGAA
ATACTTAAGGGATAATCTCCACCTGAGGTATAGCATAAGAGGCAAATATCCGTATTTGTTGTTAATATGTCAGCGAT
TGTTCGTTATATACATAGGGTAAGAAGCTAAATAGTCAAATCGTTAGGTTTGATGCTAAAAGAACTACTTACATGTT
AGTTGGCCGGCCATGTAGATACGCTAAAATAGAACAAGTCGGTATTAAAAAAAGTATTCTGAACT

>e38
ACTTAGCCTAGTATGTGCCCGAAAGAATACGGGATGGTTTTGTGTATGTTTACTCGCATCTACGTAATGCTTGCGCT
GTAAGGTCAAAACTTTTTGAAGCAGTTTATTAGTGGGTCTAACTTTCGAGTAAGGAAACATCAAAGCCTTCTAATTC
TGGCTGTAATCCCTGTGTTGGAGTCACAAACCTAGAACTTTATATATACCTTAATATGCACCTTTTGGTGCACAATA
TTTACCTACATACGTATTGTAGCTTACCGGATTGCTGCTTTTCACATTCGAAAATAAGAAACCCCCAACCTTTAGGC
TAGTGTGTATGTCCTCACTAAAAATTCACCATTAAATATCGATTCGGGTTCTACATGGAATTTTC

>e39
GATATGGATGCCAATCTAGTTTGATCCACTAGTCATGAGCGCCGTTCAGGGCATACGAGGCCATCTAAAAGTCACCC
TATATCATACTGGCATCATCACTTCTAACAATTAATTCTTTGTCAATTTCATCTTACTTGGAATGAGATAGTTAGCG
CGCCGACTTAATCTTGAATCCGTTAAAAAGATATGGTTTTATGGTCTACTCTATTAATGCGAAGACTAACAGTTATT
AACGTCCTGAATCCGAGGGTGCCGGGTAAAAGTACATCCCTCGGATAGTCCTCTTCTCCGACGGCTTATGAAAAATC
CATCTATAAAGCAACGACGCATAATTCTCATCCCTCTTGGGTGAGTACAACGCAAGTCCTTAAAC

>e40
CGTCAAACGTTTGCAAAGACTTATTATTTAATCGTTAAGGCCCTATAGAACTACTATCCTTTCCAAGGCTAGTCTTA
TATCGCTGAACGCCATTTCATTTACATCCGATATTTAAATATCAAGTACTTGCGCATTACGACGACGATAGAATGTA
CTGAATAAACCTAATTATGTGTAACTTCAAATTATCTCGTGATATGAATATGGATACTACTCTGCTACATTTCGCTT

GCACTGTTCTGTGGCTACGATCTAAATGATCATGTCTCCCTCCGTGAACCAGAGTGAAGGCCTTAGCATTAGAACTG
GAGTTCGGTGAAACTGTAAGTAACGCAGTGATTATTGACTTATCATCAAATTTCTTCCGGCGCGT

>e41
GGAAGGAGAATAAAAATATGGTTGGCGGCATAAATTCTCACAATATAGGTTGCTAGTCATTGCCTTCTTATTTCCAT
ATTTAGGTCCCACTCGCCAGGTTAATATGAAAGGTATATGAAATTTACTAAGGAAAGCCTTTACATTACAATCCTAG
TATTATCATCGTTGAATAGTGGCGTTCACCGGGTCTATTAAAATCGGCCAGAGACCGGAAATAATTAGACACCTCAC
TGTTGTATATATCTTCTTTACTAGCCTAAATTCAGCGTTGCTGAATACAATACCTTCATAATCAGAAATGTATTATG
TAGCGAAAGCCAAACTCCAATAGTTACAAGGACTTTAACCAGACTGCCTATGCTTTACTGAGATG

>e42
TCAAACGGGTCCAGAGGCAAGGCACAGTGAGTATTTTCTCACTAACTCACGTGTTCAACATGAACTAGGTTATCTAC
ATCATCAGTATTTTTCTGTCACCTCTAAAGTGAGGGTTGTCGATCTCATCCTACGTTACCCCAACCACAGCATTAAA
GTACAAAATAACTAAGCATCATTATTTGTTAATGACCAAAGATATCGATCTGCCAGTACTGGGAGTTAGGTTTTTGG
AAGTCGTTTATTAAACAACGTTGAAAGTTTCTTCAGAAATGCGCTGACTTAAATCGCCGCATTTGAGGTAAAAACTA
TAGAACTTTGAAATCAAAACTGGATCATTCCTAATTCGCTAACATCAGAATGGGTAGCTGAGTAC

>e43
ATTCGACCCCGTTATATAAAACATATCTCACCGCCAGCACGCTAAAAAGTAACCCAGACATTAACGTTTCCACACGT
CACACCGCGTATAATTACAAATTATTCCACAGAAGAGAGTAGTACAACTAGTTCCCAACCCCGAACTTAGCATCTCA
ACAGTACACAGAAAACACCAGACTAAGATATCTTCATCATGTATCTACGCTTCGCTTCTGAGGGATGATATTAGCGA
TTGAGCTTACAGATTTTTTGCTTATGCAGACCCTAGAAAAAACAAATCAAGAACGTTGATTTAACATGGACGAATTA
ACGTCATCCCGTGCATTAGGTTATGCAAGCACGGATATCCATTGTAATAAAAAATGAGGGCAATT

>e44
TATTTCATTAGACTTCTGAAATGGCATTTCTTATTGGTCAGCATAGAAATTCCTGGATTTTCCAGAACTATATAAGA
GTTCTCGAACAAGCCGGTTAATTACTGTATGATGAACACCTGATACGTTTCTCCCGACTGGTTTCCCTGTCGCAATA
TTTAGCAAATAATACGAGGCAGTATATAGCGCAGGGTTTATAGGAGCGGACGCAGAAATAATGTGTCTTTTGTTCAG
ACGTATGTTTAAGGGAGACAGAGTTCTTCTGAGCTTCCGTTAAAGGCTCATAGGAGAGCTCCGCAAGGAAACCCATC
TAATCACCCCTCCTCACATTTACTGGTAACCAAGTTACTTCAGTATATAGGACCCGCAAGGGGGA

>e45
CCGCCATTTAGCCACTAGAGCCCATTTAAGTTAGGACACGGCTGCATTGTAGTTCAGAGTCGTTAGGTGGTTTAAAG
AAGCCTTTATCTGTAATTAACTCATCCAAGGTAATCGAACAATTCCCCAATAATTGTCTCGGTTTCCGAGGTCCGAA
ATTCCGAATTTAATTTGCCCGCAGATGTTTCTTGACAACACACCCAACGAATTACTGAGACCTGGTCGTCTTAATAC
CAAATTAGACCATTCAATTATTAATGACACAGGTATCCCGACATCAGCAGGCTCATATCCATCATATTAGCAAGTCG
TGAGAATTATCAAGGTCTGTTAAAAGGTTGAGTATCAAAAAAACTTATATGTTCATGATTCGTGT

>e46
GTAGCTAGAGCGTTCCTAGCCAGCGATATATGTGTTAATAGCTTAGGATTCTGCAGGGATCTTTATTTAGGGTCGCT
TTTCGCTGTCCGTGGCGACAATCCCCTCGGTTTACTAATACAGGTGTATAATCTTACCTACACCATGTTTTCCTTCG
CAATACATAAGAAACCCCCAACGTAGCTGCTGTAACGTTGTTGCGACACAGAGCTTGGGCCCGTAGATTTTCGTATA
GAAGGGACGAGCTTGTACCAGATGTTTCCAAAGCGATCGATCTTCAGGTAAAGGTCGTATTCATAATCAGCGTGATA
GAGTCGAATGTTATTACACAACGGAAGGGTGCCACCGGATGATGGCTGCCTAATGGAAGGAGAGA

>e47
AAGCCGTTAACCAAAAACGAACAGATCACAGAATGTTGAGCAGTTTTGGCTTGGTACTATATGGCTTAACCAAAGTG
TCCCTTACTGCAGCTACTGACCGACATTTATTATAGGGCGATGTACAATTTATCAGAGGACACCACGTTAAGCAAAT
AATCGATAGATCACGATTCTTGTCCTGCCAGTTACACCCGGCTAATATACGGTTATAGTTGTTCTCTCGCGGCTGCA
CCATCGCACGAGCGTCAAATGTAATAAAACCGTGTCAAATATTGGTCGTTTTCTCACCAGGCACAACGCGTGACCAA
TCTAGACGCAATTATTGCCGATAAGATTTCGACTAATGAAGATTACTTCAAGGTACATAGTAAGC

>e48
AACCCGAGGCGCTTACAACAAAAGAGAAATGCACACTGCTTAATAACTTTTTCCTTGTACTGCATGCCCGACAAACA
AACCACCTTTTAGTCCAAACAAGAACAATTGTACCATTATCCATGTGGGCTCTCAGCTGGTAGAGAATTGTGTAAAC
ACATAAAGGGTACAGACTTCACCCATACACTTTTCGAAAAACTCGAACCTATTTTTTAGTTAGGGTAACCGCGTGGA

AGGGGAGGTCACATAATTGTTACATAATGATCTGATCTTTACGCATACATAGAAATAAAGTTTGGATTATAGGTCGA
GGATTATCACACAGTCATCATCCGGGATGTTTGTGAACACAGTTGGACAGACATGTTTTATTCTA

>e49
CGTAAAAATTGGGTTTCCAGTGACAGATGGAAACCCTATGTATTCATGTACCCTTCGGGGCCAAACATTTTCAACGG
ATATCAAAGTCACTTTTAGGAGATTAGCCGATTCGAGTTGAATCCGTTCTTTGCACAGGTAGTCAACTCGACTTGCT
TCTCGGACGTAATTTAGACTAGTGTCTTCATAATTAGATGTCCTAGGATTGATGTTATGCGTAACAGATTGGGCGGT
AGCTCAGGTAGTAAAAGAAATTTAAATGATAATTAGCGCTTCATCTAGGTGAAAGGATGACGATAGTATATGATCAA
ATTCCTATCGTGTTGTGGATAGAACACGCGTAGCAACATGTTTTAGGAGTGATAGAACCAAATAA

>e50
AACTGCGACGAGGAATAGGAGTTACATTGAAACTGTCGTTCCGGGATCAGTAGGTAATCGCAACAACTCACATAGAA
TTAACTCGGTCTGTAGTATAATTACTCATCCTACTTACATTCCATTTTATCGAATTGTAAATATTCCGTTATCCTGG
TTCGTATGAAATTCTCTTTCTAGATGGGATACTACACACTCGGCATGTGTCGGAGTGGCTGTCAGTTTCCAGTAAAT
TACTACGTCTGACTCTTATTAATTTATGATTAAACAGGTTACATTGCGAGTTATTATTACTGCTAGATACTTATCTC
ATGCATCAGAAAACGACAAGCACAGAAGTTTCATAGAGACTTTGTATTAATATGTAGTGTCATCG

Supplementary Excel Files:

Data Excel File S1: Random hits.

Data Excel File S2: Homology Index.

Data Excel File S3: Predicted Transcription Factor Binding Sites.
